# Supplementary material for: Genome-wide investigation highlights global and local pleiotropy linking neurodevelopmental disorders to acquired hearing problems
Source: Psychol Med. Author manuscript; Available in PMC 2026 May 8. (PMC12885347; doi:10.1017/S0033291726103201)
Supplement: Supplemental Material [file NIHMS2171397-supplement-Supplemental_Material.pdf]

Supplemental Table 1. Observed-scale SNP-based heritability (SNP-h2) and inflation statistics (linkage disequilibrium score regression, LDSC intercept) of the traits investigated. SE: standard error.

Phenotype	SNP-h2 (SE)	SNP-h2 Z-score	LDSC Intercept
ASD	0.422 (0.050)	8.44	0.969
ADHD	0.087 (0.004)	21.6	1.026
TS	0.361 (0.042)	8.60	1.014
HP	0.054 (0.003)	18.1	1.017

Supplemental Table 2. Genetic Correlations among hearing problems (HP), autism spectrum disorder (ASD), attention-hyperactivity disorder (ADHD), and Tourette syndrome (TS).

Phenotype 1	Phenotype 2	Genetic Correlation (rg)	Standard Error (SE)	P-value
ASD	HP	0.217	0.040	6.9E-08
ADHD	HP	0.218	0.028	4.1E-15
TS	HP	0.071	0.051	1.7E-01
ASD	ADHD	0.436	0.048	7.8E-20
ASD	TS	0.230	0.045	3.2E-07
TS	ADHD	0.129	0.061	3.4E-02

Supplemental Table 3. HP polygenic overlap with attention-hyperactivity disorder (ADHD), autism spectrum disorder (ASD), and Tourette syndrome (TS).

Only statistically meanigful MiXer models (AIC>0) were reported.

Traits	Fraction shared	Concordant fraction within shared variants	Best vs min AIC
HP-ADHD	0.34 (0.06)	0.74 (0.05)	0.81

Supplemental Table 4. Genome-wide significant associations repored in the GWAS catalog within hg19 chr12:116,197,676-117,091,843 (hg38 chr12:115,759,870-116,654,038).

Variant	P Value	Mapped Genes	Reported Trait	Study Accession
rs7969182	1.00E-133	MED13L	Height	GCST90245848
rs117774463	9.00E-36	MED13L	Bone mineral density mean	GCST90321120
rs708835	2.00E-31	LINC02463	Height	GCST90245848
rs11068058	1.00E-30	LINC00173,LINC02457	Height	GCST90245848
rs10507271	6.00E-29	MED13L	Height	GCST90245848
rs7970014,rs7977515,rs9989045	5.00E-27	LINC02463; LINC02463; LINC02463	Core binding factor acute myeloid leukemia	GCST008413
rs60109929	9.00E-20	MIR4472-2,MED13L	Monocyte count	GCST90002340
rs551567271	1.00E-18	MED13L	GLIPR1 protein levels	GCST90469357
rs11067763	2.00E-18	LINC02463,RN7SL865P	Diastolic blood pressure	GCST002631
rs17498459	4.00E-18	MED13L	Height	GCST90245844
rs11067762	5.00E-18	LINC02463,RN7SL865P	Diastolic blood pressure x smoking status (ever vs never) interaction (2df test)	GCST006190
rs57465027	5.00E-18	MED13L	Height (baseline)	GCST90565843
rs11067762	7.00E-18	LINC02463,RN7SL865P	Diastolic blood pressure x smoking status (current vs non-current) interaction (2df test)	GCST006193
rs73204034	1.00E-17	MIR4472-2,MED13L	Monocyte count	GCST90002344
rs11067773	3.00E-17	RN7SL865P,LINC02463	Atrial fibrillation	GCST90624412
rs61937364	5.00E-17	MED13L	Cutaneous melanoma or hair colour	GCST010302
rs17498550	8.00E-17	MED13L	Heel bone mineral density	GCST006979
rs60109929	4.00E-16	MIR4472-2,MED13L	Monocyte percentage of white cells	GCST90002394
rs73200232	5.00E-16	MED13L	Standing height (UKB data field 50)	GCST90468178
rs7970014,rs7977515,rs9989045	6.00E-16	LINC02463; LINC02463; LINC02463	Core binding factor acute myeloid leukemia	GCST008413
rs11067763	6.00E-16	LINC02463,RN7SL865P	Systolic blood pressure	GCST002630
rs61928421	9.00E-16	RN7SL865P,LINC02463	Atrial fibrillation	GCST90624411
rs73204034	1.00E-15	MIR4472-2,MED13L	Monocyte count (UKB data field 30130)	GCST90468090
rs17498550	1.00E-15	MED13L	Heel bone mineral density	GCST006433
rs60109929	2.00E-15	MIR4472-2,MED13L	Monocyte count	GCST90002393
rs73200241	3.00E-15	MED13L	Height	GCST90435412
rs61928421	4.00E-15	RN7SL865P,LINC02463	Heart rate response to recovery post exercise (40 sec)	GCST005849
rs11067763	4.00E-15	LINC02463,RN7SL865P	Diastolic blood pressure	GCST90310295
rs11067763	4.00E-15	LINC02463,RN7SL865P	Diastolic blood pressure	GCST90278625
rs2392971	7.00E-15	MED13L,MIR4472-2	Height	GCST007841
rs11067762	1.00E-14	LINC02463,RN7SL865P	Systolic blood pressure x smoking status (ever vs never) interaction (2df test)	GCST006192
rs11067762	1.00E-14	LINC02463,RN7SL865P	Systolic blood pressure x smoking status (current vs non-current) interaction (2df test)	GCST006195
rs112761681	2.00E-14	MED13L	Cutaneous melanoma (MTAG)	GCST90103971
rs3858652	3.00E-14	LINC02463,RN7SL865P	Height	GCST90245844
rs11616067	3.00E-14	MED13L,LINC02463	Height	GCST90245845
rs60109929	4.00E-14	MIR4472-2,MED13L	Monocyte percentage (UKB data field 30190)	GCST90468091
rs61928421	5.00E-14	RN7SL865P,LINC02463	Heart rate response to recovery post exercise (50 sec)	GCST005848
rs10850679	7.00E-14	LINC03088,MAP1LC3B2	Height	GCST90245848
rs11067763	4.00E-13	LINC02463,RN7SL865P	Diastolic blood pressure (MTAG)	GCST90449057
rs373112267	1.00E-12	LINC02463,MED13L	Musculoskeletal symptoms referable to limbs (PheCode 771)	GCST90480576
rs17498550	2.00E-12	MED13L	Heel bone mineral density	GCST007066
rs187309577	2.00E-12	MED13L	Enterococcus abundance in stool	GCST90032364
rs778269542	2.00E-12	RN7SL865P,LINC02463	Pulse rate (UKB data field 102)	GCST90468177
rs7962636	2.00E-12	MED13L	Weight (UKB data field 21002)	GCST90468183
rs61928421	2.00E-12	RN7SL865P,LINC02463	Atrial fibrillation	GCST90559230
rs60109929	3.00E-12	MIR4472-2,MED13L	Monocyte count	GCST90018967
rs73204034	4.00E-12	MIR4472-2,MED13L	Monocyte percentage of white cells	GCST004609
rs7962636	6.00E-12	MED13L	Body surface area	GCST90245995
rs61928421	7.00E-12	RN7SL865P,LINC02463	Heart rate response to recovery post exercise (20 sec)	GCST005847
rs73204034	1.00E-11	MIR4472-2,MED13L	Monocyte count	GCST004625
rs74550975	1.00E-11	MED13L,MIR4472-2	Vertex-wise cortical surface area	GCST90095130
rs56295994	1.00E-11	MED13L	Pulse pressure	GCST90310296
rs61928421	2.00E-11	RN7SL865P,LINC02463	Heart rate response to recovery post exercise (30 sec)	GCST005850
rs75938105	2.00E-11	LINC02463	Insulin-like growth factor 1 levels	GCST90019511
rs9651899	3.00E-11	LINC02463,RN7SL865P	Adolescent idiopathic scoliosis	GCST006287
rs11067773	3.00E-11	RN7SL865P,LINC02463	Heart rate response to recovery post exercise	GCST005788
rs11067763	4.00E-11	LINC02463,RN7SL865P	Diastolic blood pressure	GCST90435414
rs74550975	4.00E-11	MED13L,MIR4472-2	Cortical surface area	GCST90091060
rs71469748	4.00E-11	MED13L	Smoking initiation	GCST90243968
rs11067763	4.00E-11	LINC02463,RN7SL865P	systolic blood pressure (SBP, mean, inv-normal transformed)	GCST90480706
rs61928421	5.00E-11	RN7SL865P,LINC02463	Heart rate response to recovery post exercise (10 sec)	GCST005846
rs71469748	5.00E-11	MED13L	Smoking initiation	GCST90243985
rs11067762	5.00E-11	LINC02463,RN7SL865P	Mean arterial pressure	GCST90239619
rs143507801	6.00E-11	MED13L	Enterococcus faecalis abundance in stool	GCST90032363
rs13097571,rs780313	6.00E-11	RN7SL553P - MTARC2P1 x LINC02463	Total PHF-tau (SNP x SNP interaction)	GCST010340
rs11615689	9.00E-11	MED13L	Pulse pressure	GCST006629
rs17427318	1.00E-10	MED13L,MIR4472-2	Lymphocyte count	GCST90002320
rs11068059	2.00E-10	LINC00173,LINC02457	Blond vs. brown/black hair color	GCST006988
rs11067762	2.00E-10	LINC02463,RN7SL865P	Diastolic blood pressure	GCST90239616
rs11067762	3.00E-10	LINC02463,RN7SL865P	Mean arterial pressure (BMI adjusted)	GCST90335169
rs118171107	3.00E-10	RN7SL865P,LINC02463	Height (baseline)	GCST90565843
rs17427318	4.00E-10	MED13L,MIR4472-2	Lymphocyte count	GCST90002316
rs802122	4.00E-10	RN7SL865P,LINC02463	Heart rate variability (root mean square of successive differences)	GCST90281263
rs11067763	5.00E-10	LINC02463,RN7SL865P	Systolic blood pressure	GCST90310294
rs7315837	6.00E-10	MED13L,MIR4472-2	Retinal arteriolar tortuosity	GCST90270397
rs10850564	6.00E-10	RN7SL865P,LINC02463	Diastolic blood pressure	GCST90132904
rs11067763	6.00E-10	LINC02463,RN7SL865P	Systolic blood pressure	GCST90278642
rs76130193	8.00E-10	MED13L	Educational attainment	GCST90105038
rs16870983,rs10774860	8.00E-10	IRX2-DT - LINC01377 x MIR4472-2 - LINC02457	Total PHF-tau (SNP x SNP interaction)	GCST010340
rs113469387	9.00E-10	MED13L	Cutaneous malignant melanoma	GCST010304
rs74550975	9.00E-10	MED13L,MIR4472-2	Vertex-wise sulcal depth	GCST90095129
rs12372279	9.00E-10	LINC02457	Body mass index	GCST90255621
rs3858652	9.00E-10	LINC02463,RN7SL865P	Whole body fat free mass (UKB data field 23101)	GCST90428120
rs11067762	1.00E-09	LINC02463,RN7SL865P	Diastolic blood pressure (BMI adjusted)	GCST90335165
rs7962636	2.00E-09	MED13L	Educational attainment (MTAG)	GCST006571
rs7962636	2.00E-09	MED13L	Body mass index	GCST90255621
rs697883	2.00E-09	LINC02463	Whole body fat free mass (UKB data field 23101)	GCST90428120
rs11067763	3.00E-09	LINC02463,RN7SL865P	Diastolic blood pressure	GCST007094
rs2707183	3.00E-09	LINC02457	Metabolic syndrome	GCST90444487
rs61939692	4.00E-09	MED13L	Blond vs. brown/black hair color	GCST006988
rs61937394	4.00E-09	MED13L,MIR4472-2	Ascending aorta diameter	GCST90267390
rs2392971	5.00E-09	MED13L,MIR4472-2	Physical function (baseline)	GCST90565837
rs802122	5.00E-09	RN7SL865P,LINC02463	Heart rate variability (corrected root mean square of successive differences)	GCST90281264
rs61936936	6.00E-09	LINC02463,MED13L	Body size at age 10	GCST010989
rs10507271	6.00E-09	MED13L	Physical function (baseline)	GCST90565837
rs2603812	6.00E-09	MED13L	Height (baseline)	GCST90565843
rs73204034	7.00E-09	MIR4472-2,MED13L	Granulocyte percentage of myeloid white cells	GCST004608
rs4301303,rs10444487	7.00E-09	LRFN2 - UNC5CL x MED13L	Age-related hearing impairment (SNP x SNP interaction)	GCST002487
rs146151364	8.00E-09	LINC02463,MED13L	Hypertrophy of breast (Gynecomastia) (PheCode 612.2)	GCST90651443
rs117356123	9.00E-09	MED13L	Protein quantitative trait loci (liver)	GCST011427
rs150024806	9.00E-09	MED13L	Protein quantitative trait loci (liver)	GCST011427
rs10850587,rs10904323	9.00E-09	LINC02463 - MED13L x LINC00705 - AKR1E2	Total PHF-tau (SNP x SNP interaction)	GCST010340
rs74643199	1.00E-08	MIR4472-2,MED13L	Ease of getting up in the morning	GCST007986
rs113469387	1.00E-08	MED13L	Nevus count or cutaneous melanoma	GCST010303

rs113281820	1.00E-08	MIR4472-2,MED13L	Crohn's disease	GCST90446793
rs11067773	1.00E-08	RN7SL865P,LINC02463	PR interval	GCST007045
rs7958254	1.00E-08	LINC02463,MED13L	CAG-632 abundance in stool	GCST90032294
rs61937385	1.00E-08	MED13L	Educational attainment	GCST90105038
rs78349600	1.00E-08	MAP1LC3B2,LINC03088	Gut microbiome abundance (class Clostridium sensu stricto sp. (at 1 year) x Winter Birth (Dec-Feb) interaction	GCST90568890
rs61937387	1.00E-08	MED13L	Whole body fat free mass (UKB data field 23101)	GCST90428120
rs11067904	2.00E-08	MED13L	Height	GCST008839
rs58984824	2.00E-08	MED13L,MIR4472-2	Reaction time	GCST006268
rs17427318	2.00E-08	MED13L,MIR4472-2	Lymphocyte count	GCST90018962
rs58984824	2.00E-08	MED13L,MIR4472-2	Reaction time	GCST90011294
rs61937394	2.00E-08	MED13L,MIR4472-2	Ascending thoracic aortic diameter	GCST90094400
rs7962636	2.00E-08	MED13L	Body mass index	GCST90301650
rs73196098	3.00E-08	MED13L	Systolic blood pressure	GCST007087
rs2575286	3.00E-08	LINC02463	Appendicular lean mass	GCST009576
rs73200209	3.00E-08	MED13L	Total body bone mineral density	GCST005348
rs7308437	3.00E-08	MED13L	Height	GCST90018739
rs118171107	3.00E-08	RN7SL865P,LINC02463	Physical function (baseline)	GCST90565837
rs697881	3.00E-08	LINC02463	Height (baseline)	GCST90565843
rs11085234,rs697862	3.00E-08	RNU6-2 - CNN2 x LINC02463	Total PHF-tau (SNP x SNP interaction)	GCST010340
rs12423578	4.00E-08	MED13L	Height (standard GWA)	GCST90267284
rs73400807	4.00E-08	MIR4472-2,MED13L	Educational attainment	GCST90105038

Supplemental Table 5. Genome-wide significant associations reported in the GWAS catalog within hg19 chr6:149,278,799-150,635,315 (hg38 chr6:148,957,662-150,314,179).

Variant	P Value	Mapped Genes	Reported Trait	Study Accession
rs9371533	4.00E-286	RAET1E,RAET1E-AS1	Blood protein levels	GCST006585
rs76968226	4.00E-238	ULBP1	ULBP2 protein levels	GCST90471008
rs34337297	3.00E-161	LATS1	Serum levels of protein LRP11	GCST90090134
rs117243801	2.00E-112	PCMT1	LRP11 protein levels	GCST90469796
rs6918234	1.00E-106	ULBP3,PHB1P1	NKG2D ligand 3 levels	GCST90425447
rs4343924	1.00E-104	ULBP2	ULBP2 protein levels	GCST90471008
rs3039620	3.00E-100	PCMT1	Low-density lipoprotein receptor-related protein 11 levels	GCST90179352
rs10872645	2.00E-86	NUP43	Blood protein levels	GCST006585
rs4870170	7.00E-81	BTF3P10,RAET1K	NKG2D ligand 2 levels	GCST90060011
rs555648964	1.00E-79	RAET1E,RAET1E-AS1	LRP11 protein levels	GCST90469796
rs6557221	5.00E-79	RAET1K,BTF3P10	NKG2D ligand 2 (analyte X22583.47) levels	GCST90424475
rs57215159	5.00E-72	LATS1	Retinal thickness (pixel 62_94)	GCST90527943
rs76004317	5.00E-60	PPIL4	LRP11 protein levels	GCST90469796
rs6557222	2.00E-59	BTF3P10,RAET1K	Retinoic acid early transcript 1L protein (analyte X10589.7) levels	GCST90421169
rs142987810	4.00E-58	LRP11	LRP11 protein levels	GCST90469796
rs150467898	4.00E-56	LATS1	LRP11 protein levels	GCST90469796
rs117984853	4.00E-55	UST,TAB2	Atrial fibrillation	GCST90624412
rs2153252	1.00E-54	UST	Height	GCST90245848
rs117984853	1.00E-53	UST,TAB2	Atrial fibrillation	GCST90624411
rs9765961	6.00E-51	RPS18P9	Low-density lipoprotein receptor-related protein 11 levels	GCST90179352
rs12194150	1.00E-50	ULBP1,ULBP2	ULBP2 protein levels	GCST90471008
rs6557221	2.00E-50	RAET1K,BTF3P10	Retinoic acid early transcript 1L protein (analyte X20526.3) levels	GCST90423773
rs9498290	1.00E-47	TAB2	LRP11 protein levels	GCST90469796
rs117984853	2.00E-47	UST,TAB2	Atrial fibrillation	GCST90559230
rs78360446	2.00E-46	KATNA1,LATS1	LRP11 protein levels	GCST90469796
rs73604783	2.00E-42	GINM1	Low-density lipoprotein receptor-related protein 11 levels	GCST90248262
rs116968353	2.00E-41	PPP1R14C,ULBP3	ULBP2 protein levels	GCST90471008
rs61730071	8.00E-38	RAET1L	Retinoic acid early transcript 1L protein level in Chronic kidney disease with hypertension and no diabetes (20526_3)	GCST90235450
rs116912868	8.00E-38	PCMT1	Serum levels of protein LRP11	GCST90090134
rs144950678	1.00E-36	RAET1G,RAET1E-AS1	LRP11 protein levels	GCST90469796
rs573904049	3.00E-34	LATS1,KATNA1	LRP11 protein levels	GCST90469796
rs558980931	3.00E-33	GINM1,PPIL4	ULBP2 protein levels	GCST90471008
rs34245267	3.00E-33	UST,TAB2	LRP11 protein levels	GCST90469796
rs564778845	9.00E-33	RAET1K,RAET1L	ULBP2 protein levels	GCST90471008
rs35161649	4.00E-32	PCMT1	Low-density lipoprotein receptor-related protein 11 levels	GCST90248262
rs7752089	2.00E-31	LRP11	Brain morphology (MOSTest)	GCST90239729
rs9688867	5.00E-31	PCMT1	Low-density lipoprotein receptor-related protein 11 level in Chronic kidney disease with hypertension and no diabetes (8330_1)	GCST90238893
rs9479808	2.00E-30	PCMT1	Adenine levels	GCST90245098
rs777771162	2.00E-29	LATS1	GLIPR1 protein levels	GCST90469357
rs112146772	3.00E-29	LRP11	LRP11 protein levels	GCST90469796
rs544441045	4.00E-29	ULBP2,RAET1G	ULBP2 protein levels	GCST90471008
rs4870015	4.00E-29	PCMT1	Metabolite levels (S-adenosylhomocysteine (SAH); ___adenosylhomocysteine; S_Ado_homocysteine)	GCST90300219
rs9383665	4.00E-28	PPP1R14C,ULBP3	LRP11 protein levels	GCST90469796
rs55763431	8.00E-28	PCMT1	Low-density lipoprotein receptor-related protein 11 level in Chronic kidney disease with hypertension and no diabetes (6713_4)	GCST90238384
rs9688867	2.00E-26	PCMT1	S-adenosylhomocysteine (SAH) levels	GCST90200345
rs4394226	3.00E-26	RAET1G,ULBP2	ULBP2 protein levels	GCST90471008
rs9373596	5.00E-26	GINM1,PPIL4	Low-density lipoprotein receptor-related protein 11 level in Chronic kidney disease with hypertension and no diabetes (15472_16)	GCST90234391
rs117578942	5.00E-26	LATS1	LRP11 protein levels	GCST90469796
rs674919	5.00E-26	TAB2,ZC3H12D	Height	GCST90245848
rs237002	5.00E-25	ZC3H12D,TAB2	ULBP2 protein levels	GCST90471008
rs117208965	5.00E-25	LRP11	LRP11 protein levels	GCST90469796
rs143105482	7.00E-25	BTF3P10,RAET1K	ULBP2 protein levels	GCST90471008
rs117984853	1.00E-24	UST,TAB2	Atrial fibrillation	GCST006414
rs11441223	2.00E-24	KATNA1	Retinal thickness (functional principal component 5)	GCST90455725
rs12523793	4.00E-24	LATS1,KATNA1	Subcortical volume (MOSTest)	GCST010702
rs77261077	4.00E-24	KATNA1	LRP11 protein levels	GCST90469796
rs12183587	6.00E-24	RAET1M	Alopecia areata	GCST004866
rs117984853	1.00E-23	UST,TAB2	Atrial fibrillation (MTAG)	GCST90449054
rs117984853	1.00E-23	UST,TAB2	Atrial fibrillation (MTAG)	GCST90132229
rs13195599	1.00E-23	RNU4-7P,PPP1R14C	Height	GCST90245848
rs1889471	4.00E-23	RAET1E-AS1	Low-density lipoprotein receptor-related protein 11 (analyte X15472.16) levels	GCST90422684
rs117718395	3.00E-22	ULBP2,ULBP1	ULBP2 protein levels	GCST90471008
rs144413146	4.00E-22	LATS1	LRP11 protein levels	GCST90469796
rs547269472	4.00E-22	ULBP2	LRP11 protein levels	GCST90469796
rs1475756	6.00E-22	LRP11	LRP11 protein levels	GCST90469796
rs12526675	7.00E-22	NUP43	Brain morphology (MOSTest)	GCST010703
rs75672805	9.00E-22	PCMT1	LRP11 protein levels	GCST90469796
rs11155675	1.00E-21	NUP43	LATS1 protein levels	GCST90469740
rs567051977	2.00E-21	RAET1E-AS1,RAET1E	LRP11 protein levels	GCST90469796
rs2095375	1.00E-20	PCMT1	Serum metabolite levels	GCST012021
rs2095375	1.00E-20	PCMT1	Serum metabolite levels	GCST012020
rs184540490	2.00E-20	BTF3P10,RAET1K	ULBP2 protein levels	GCST90471008
rs141033986	2.00E-20	KATNA1	LRP11 protein levels	GCST90469796
rs7773289	7.00E-20	PPP1R14C	ULBP2 protein levels	GCST90471008
rs9505982	1.00E-19	KATNA1	S-adenosylhomocysteine (SAH) levels	GCST90139559
rs138397082	2.00E-19	GINM1	LRP11 protein levels	GCST90469796
rs14314	3.00E-19	LRP11	Macular thickness	GCST006976
rs9479482	4.00E-19	PHB1P1,RAET1M	Alopecia areata	GCST000719
rs186994920	1.00E-18	KATNA1	ULBP2 protein levels	GCST90471008
rs4336467	1.00E-18	UST	Peak expiratory flow	GCST90244095
rs869109015	3.00E-18	LRP11	5-methylthioadenosine (mta) levels	GCST90139562
rs394563	1.00E-17	ZC3H12D	Dupuytren's disease	GCST004858
rs191469589	1.00E-17	ULBP3,PPP1R14C	ULBP2 protein levels	GCST90471008
rs9498383	1.00E-17	KATNA1	Left hippocampal volume	GCST90267908
rs117984853	3.00E-17	UST,TAB2	Atrial fibrillation	GCST006061
rs9285521	3.00E-17	GINM1	Plasma 5-methylthioadenosine (MTA) levels in chronic kidney disease	GCST90264694
rs55945045	3.00E-17	LATS1	LRP11 protein levels	GCST90469796
rs10872653	4.00E-17	PCMT1	Urine S-adenosylhomocysteine (SAH) levels in chronic kidney disease	GCST90265919
rs183113893	6.00E-17	PPP1R14C,ULBP3	ULBP2 protein levels	GCST90471008
rs14314	7.00E-17	LRP11	Vertex-wise sulcal depth	GCST90095129
rs78811127	7.00E-17	UST,TAB2	Atrial fibrillation	GCST90204201
rs117984853	8.00E-17	UST,TAB2	Atrial fibrillation	GCST006061
rs117938827	8.00E-17	GINM1	LRP11 protein levels	GCST90469796
rs187211175	9.00E-17	LATS1	LRP11 protein levels	GCST90469796
rs366905	1.00E-16	TAB2,ZC3H12D	Dupuytren's disease	GCST90301252
rs117984853	2.00E-16	UST,TAB2	Atrial fibrillation/atrial flutter	GCST90018796
rs2500513	2.00E-16	UST	LRP11 protein levels	GCST90469796
rs1889473	4.00E-16	LRP11	Cerebrospinal fluid 5-methylthioadenosine (MTA) levels	GCST90318216
rs14314	4.00E-16	LRP11	Plasma S-adenosylhomocysteine (SAH) levels in chronic kidney disease	GCST90265918
rs11758260	8.00E-16	KATNA1	LRP11 protein levels	GCST90469796
rs9498383	8.00E-16	KATNA1	Right hippocampal volume	GCST90267930
rs144586141	2.00E-15	PCMT1	LRP11 protein levels	GCST90469796
rs727979	3.00E-15	TAB2	Non-albumin protein levels	GCST90019515
rs181773431	5.00E-15	RAET1E-AS1,RAET1G	ULBP2 protein levels	GCST90471008
rs60171724	5.00E-15	PCMT1	Left subiculum volume (head)	GCST90267915
rs146529055	6.00E-15	RAET1G	LRP11 protein levels	GCST90469796
rs560556494	7.00E-15	PHB1P1,RAET1M	ULBP2 protein levels	GCST90471008
rs14314	7.00E-15	LRP11	Left hippocampal volume (body)	GCST90267904
rs4897101	9.00E-15	TAB2	Total protein levels (UKB data field 30860)	GCST90468105
rs14314	1.00E-14	LRP11	Left subiculum volume (body)	GCST90267914

rs201827278	2.00E-14	PCMT1	LRP11 protein levels	GCST90469796
rs60171724	2.00E-14	PCMT1	Right subiculum volume (head)	GCST90267937
rs537262812	2.00E-14	PCMT1	Low-density lipoprotein receptor-related protein 11 levels	GCST90248262
rs1889471	3.00E-14	RAET1E-AS1	Low-density lipoprotein receptor-related protein 11 (analyte X8330.1) levels	GCST90427338
rs6906384	5.00E-14	TAB2	Dupuytren's disease	GCST90297847
rs1125	8.00E-14	LATS1	Vertical cup-disc ratio	GCST90129627
rs558914963	1.00E-13	TAB2	ULBP2 protein levels	GCST90471008
rs366905	1.00E-13	TAB2,ZC3H12D	Contracture of palmar fascia [Dupuytren's disease] (PheCode 728.71)	GCST90480521
rs9377205	2.00E-13	TAB2	Serum total protein levels	GCST90018976
rs3949414	2.00E-13	UST	Peak expiratory flow (UKB data field 3064)	GCST90468176
rs12193052	2.00E-13	TAB2	LRP11 protein levels	GCST90469796
rs145162715	2.00E-13	GINM1	LRP11 protein levels	GCST90469796
rs727979	3.00E-13	TAB2	Serum total protein levels	GCST90019522
rs391875	5.00E-13	ZC3H12D	Eosinophill percentage (UKB data field 30210)	GCST90468069
rs539700294	5.00E-13	PCMT1	Vertex-wise cortical surface area	GCST90095130
rs181620867	9.00E-13	RAET1L	ULBP2 protein levels	GCST90471008
rs7746208	9.00E-13	UST,TAB2	Height	GCST90245848
rs75337203	1.00E-12	PPP1R14C,RNU4-7P	ULBP2 protein levels	GCST90471008
rs181134994	1.00E-12	TAB2	LRP11 protein levels	GCST90469796
rs28740897	1.00E-12	PCMT1	Right subiculum volume (body)	GCST90267936
rs142525075	2.00E-12	UST	ULBP2 protein levels	GCST90471008
rs111779138	2.00E-12	LATS1	LRP11 protein levels	GCST90469796
rs9383881	2.00E-12	RAET1E-AS1	LRP11 protein levels	GCST90469796
rs12174588	2.00E-12	PCMT1	Right hippocampal volume (tail)	GCST90267929
rs12176034	3.00E-12	PCMT1	Cerebrospinal fluid adenine levels	GCST90318284
rs11752529	3.00E-12	RAET1L,RAET1M	Basal cell carcinoma	GCST90328134
rs9485372	4.00E-12	TAB2	Breast cancer	GCST001420
rs11752228	4.00E-12	PPP1R14C	LRP11 protein levels	GCST90469796
rs35037735	4.00E-12	RAET1M,RAET1L	LRP11 protein levels	GCST90469796
rs4869780	5.00E-12	BTF3P10,RAET1K	Aspartate aminotransferase levels	GCST90244010
rs10457078	5.00E-12	RAET1K,BTF3P10	LRP11 protein levels	GCST90469796
rs9689694	6.00E-12	PCMT1	Left hippocampal volume (head)	GCST90267906
rs193040741	7.00E-12	ULBP2,ULBP1	ULBP2 protein levels	GCST90471008
rs9689694	7.00E-12	PCMT1	Hippocampal subfield right CA1 volume (head)	GCST90267917
rs11752529	7.00E-12	RAET1L,RAET1M	Basal cell carcinoma	GCST90328132
rs9485372	8.00E-12	TAB2	Breast cancer	GCST90090980
rs185861332	8.00E-12	RPS18P9	LRP11 protein levels	GCST90469796
rs9688452	1.00E-11	LATS1	Brainstem global volume Whole-brainstem	GCST90002768
rs4380763	1.00E-11	LATS1	Subcortical volume (min-P)	GCST010698
rs11753049	1.00E-11	PPP1R14C	ULBP2 protein levels	GCST90471008
rs4039600	1.00E-11	GINM1	Adenine levels	GCST90140274
rs391875	2.00E-11	ZC3H12D	Eosinophil percentage of white cells	GCST90002382
rs9322194	2.00E-11	KATNA1	Brainstem volume	GCST009697
rs140924397	2.00E-11	UST,TAB2	Exophthalmos (PheCode 242.3)	GCST90479865
rs17669546	3.00E-11	UST	Height	GCST007841
rs12662494	3.00E-11	PHB1P1,ULBP3	SH2 domain-containing adapter protein D levels	GCST90424770
rs539700294	3.00E-11	PCMT1	Cortical surface area	GCST90091060
rs570063769	3.00E-11	LRP11	Unspecified diffuse connective tissue disease (PheCode 709.7)	GCST90480488
rs4421206	4.00E-11	LATS1	Brain morphology (min-P)	GCST010699
rs202138115	5.00E-11	LATS1,KATNA1	Brain region volumes	GCST009518
rs1853665	5.00E-11	ULBP1,BTF3P10	Neurological blood protein biomarker levels	GCST008478
rs190421361	5.00E-11	TAB2	Low-density lipoprotein receptor-related protein 11 levels	GCST90179352
rs28740897	5.00E-11	PCMT1	Hippocampal left GC-ML-DG volume (body)	GCST90267901
rs431978	6.00E-11	ZC3H12D	Eosinophil counts	GCST007065
rs28740897	6.00E-11	PCMT1	Hippocampal subfield left CA4 volume (body)	GCST90267898
rs9689694	6.00E-11	PCMT1	Right hippocampal volume (head)	GCST90267928
rs114623501	7.00E-11	ZC3H12D	Eosinophil counts	GCST90002298
rs9322189	8.00E-11	GINM1	Hand grip strength (baseline)	GCST90565845
rs12178835	1.00E-10	PCMT1	Brain shape (segment 116)	GCST90012995
rs7758080	1.00E-10	TAB2,TAB2-AS1	Breast cancer	GCST90018799
rs10872649	1.00E-10	PCMT1	Right hippocampal volume (body)	GCST90267926
rs431362	2.00E-10	ZC3H12D	Asthma	GCST010043
rs9688452	2.00E-10	LATS1	aseg lh volume VentralDC	GCST90002627
rs9689694	2.00E-10	PCMT1	Hippocampal subfield left CA1 volume (head)	GCST90267895
rs12174588	2.00E-10	PCMT1	Left hippocampal volume (tail)	GCST90267907
rs80147756	3.00E-10	NUP43	HippSubfield lh volume subiculum-head	GCST90002671
rs9371505	3.00E-10	LRP11	Low-density lipoprotein receptor-related protein 11 (analyte X6713.4) levels	GCST90426812
rs4870050	3.00E-10	LRP11	Hand grip strength (baseline)	GCST90565845
rs1564602	4.00E-10	UST	Atrial fibrillation (MTAG)	GCST90449054
rs1999670,rs9383	6.00E-10	R14C; PPP1R14C; ULBP3 - PPP1R14C; ULB	Core binding factor acute myeloid leukemia	GCST008413
rs1562468327	6.00E-10	TAB2,ZC3H12D	Aromatic antiseizure medication-induced Stevens-Johnson syndrome or toxic epidermal necrolysis in HLA-B*15:02 non-carriers	GCST90103804
rs1001305	6.00E-10	ZC3H12D,TAB2	Peak expiratory flow	GCST90244095
rs1999670,rs9383	7.00E-10	R14C; PPP1R14C; ULBP3 - PPP1R14C; ULB	Core binding factor acute myeloid leukemia	GCST008413
rs4870163	7.00E-10	ULBP1	Platelet count	GCST90018969
rs7758080	8.00E-10	TAB2,TAB2-AS1	Crohn's disease	GCST90446792
rs6901019	8.00E-10	UST,TAB2	Acetabular diameter	GCST90448906
rs391875	1.00E-09	ZC3H12D	Eosinophil counts	GCST90002381
rs9383843	1.00E-09	PCMT1,LRP11	Migraine	GCST90102553
rs4869780	1.00E-09	BTF3P10,RAET1K	Platelet count	GCST90018749
rs58189451	1.00E-09	KATNA1	Drinks per week	GCST90243989
rs2184370	1.00E-09	LRP11	Hypertrophic cardiomyopathy (MTAG)	GCST90435257
rs58014361	1.00E-09	PCMT1	Volume of left basal nucleus	GCST90474877
rs9689694	1.00E-09	PCMT1	Volume of left basal nucleus	GCST90474876
rs7748981	1.00E-09	TAB2	Height	GCST90245848
rs17054300	2.00E-09	PHB1P1,ULBP3	Blood protein levels	GCST006585
rs11155671	2.00E-09	KATNA1,LATS1	Testicular germ cell tumor	GCST004635
rs11155662	2.00E-09	RPS18P9,GINM1	Vertex-wise cortical thickness	GCST90095131
rs28740897	2.00E-09	PCMT1	Left presubiculum volume (head)	GCST90267913
rs11155675	3.00E-09	NUP43	Brain region volumes	GCST009518
rs9373594	3.00E-09	PPIL4	Rheumatoid arthritis	GCST002318
rs35409891	3.00E-09	TAB2	Breast cancer	GCST004988
rs768512669	3.00E-09	ZC3H12D	Asthma	GCST010042
rs4869780	3.00E-09	BTF3P10,RAET1K	Aspartate aminotransferase levels	GCST90018724
rs14314	3.00E-09	LRP11	Volume of left cortico-amygdaloid transition area	GCST90474879
rs14314	3.00E-09	LRP11	Volume of left cortico-amygdaloid transition area	GCST90474880
rs10872649	3.00E-09	PCMT1	Hippocampal subfield right CA4 volume (body)	GCST90267920
rs28740897	3.00E-09	PCMT1	Left molecular layer hippocampal volume (head)	GCST90267910
rs1320575	5.00E-09	TAB2,UST	Atrial fibrillation (MTAG)	GCST90449054
rs1475756	5.00E-09	LRP11	Heel bone mineral density x serum urate levels interaction	GCST012489
rs4869780	6.00E-09	BTF3P10,RAET1K	Aspartate aminotransferase levels	GCST90018944
rs17054300	7.00E-09	PHB1P1,ULBP3	Blood protein levels	GCST006585
rs7758080	7.00E-09	TAB2,TAB2-AS1	Crohn's disease	GCST003044
rs6570952	7.00E-09	TAB2,UST	Electrocardiogram morphology (amplitude at temporal datapoints)	GCST010796
rs9688809	7.00E-09	PCMT1	Right molecular layer hippocampal volume (head)	GCST90267932
rs35989414	9.00E-09	LATS1	C-reactive protein	GCST90018950
rs12205199	1.00E-08	RAET1M,RAET1L	Basal cell carcinoma	GCST008871
rs7746208	1.00E-08	UST,TAB2	Vertex-wise sulcal depth	GCST90095129
rs9322194	1.00E-08	KATNA1	Regional cortical thickness (parahippocampal)	GCST90399890
rs75353579	1.00E-08	RAET1G	Heel bone mineral density x serum urate levels interaction	GCST012489
rs2342764	1.00E-08	LRP11	Cortical thickness	GCST90091061
rs2744427	2.00E-08	TAB2	Coronary artery disease	GCST010866
rs200741605	2.00E-08	TAB2	Hip circumference adjusted for BMI	GCST90020028
rs12523793	2.00E-08	LATS1,KATNA1	C-reactive protein levels (MTAG)	GCST90179146

rs552736307	2.00E-08	ZC3H12D,TAB2	Triglyceride levels	GCST90528079
rs7747354	2.00E-08	ZC3H12D	Peak expiratory flow	GCST90270085
rs1934534	2.00E-08	LATS1	Pain (pleiotropy)	GCST90104572
rs9478311,rs4623	2.00E-08	ULBP1 x PPIAP48 - CDH11	Total PHF-tau (SNP x SNP interaction)	GCST010340
rs10428766	3.00E-08	PHB1P1,ULBP3	Eosinophil counts	GCST007065
rs912558,rs69417	3.00E-08	ULBP3 x CDYL	Total PHF-tau (SNP x SNP interaction)	GCST010340
rs6570952	3.00E-08	TAB2,UST	Electrocardiogram morphology (amplitude at temporal datapoints)	GCST010796
rs200741605	3.00E-08	TAB2	Hip index	GCST90020026
rs12523793	3.00E-08	LATS1,KATNA1	C-reactive protein levels	GCST90029070
rs6922684	3.00E-08	ULBP3,PPP1R14C	Gut microbiome abundance (class Bacteroides sp. 8 (at 1 year) x Household furry pet cat (3 Months) interaction	GCST90568729
rs10872649	3.00E-08	PCMT1	Hippocampal right GC-ML-DG volume (body)	GCST90267923
rs2500535	4.00E-08	UST	Response to antidepressants	GCST000643
rs9404024	4.00E-08	TAB2	Neutrophils and lymphocytes in blood (confirmatory factor analysis Factor 35)	GCST90309369
rs4897110	4.00E-08	TAB2	Hip shape mode 2	GCST90482705
rs58014361	4.00E-08	PCMT1	Volume of left paralaminar nucleus	GCST90474895
rs58014361	4.00E-08	PCMT1	Volume of left whole amygdala	GCST90474898
rs11968921	4.00E-08	PCMT1,LRP11	Mean volume of bilateral amygdala	GCST90474900
rs56103941	4.00E-08	PCMT1,LRP11	Right presubiculum volume (head)	GCST90267935
rs12178835	4.00E-08	PCMT1	Left presubiculum volume (body)	GCST90267912
rs9969044	4.00E-08	RAET1E-AS1,RAET1E	Height	GCST90245848

Supplemental Table 6. Genome-wide significant associations reported in the GWAS catalog within hg19 chr2:9,960,671-10,611,654 (hg38 chr2:9,820,542-10,471,528).

Variant	P Value	Mapped Genes	Reported Trait	Study Accession
rs4669524	2.00E-278	CYS1,KLF11	Height	GCST90245848
rs4669524	6.00E-234	CYS1,KLF11	Height	GCST90245843
rs375285744	2.00E-115	HPCAL1	HPCAL1 protein levels	GCST90469471
rs3755259	1.00E-103	HPCAL1	CASP3/HPCAL1 protein level ratio	GCST90313633
rs3755259	1.00E-98	HPCAL1	HPCAL1/NUDC protein level ratio	GCST90315074
rs3755259	2.00E-96	HPCAL1	HPCAL1/PTPN6 protein level ratio	GCST90315077
rs3755259	1.00E-94	HPCAL1	HPCAL1/PLA2G4A protein level ratio	GCST90315075
rs375285744	5.00E-93	HPCAL1	Hippocalcin-like protein 1 levels	GCST90247923
rs3755259	6.00E-84	HPCAL1	DFFA/HPCAL1 protein level ratio	GCST90314458
rs3755259	2.00E-76	HPCAL1	DNAJA2/HPCAL1 protein level ratio	GCST90314503
rs3755259	2.00E-76	HPCAL1	CDKN2D/HPCAL1 protein level ratio	GCST90313986
rs3755259	5.00E-76	HPCAL1	GP6/HPCAL1 protein level ratio	GCST90314963
rs10169634	6.00E-68	HPCAL1	HPCAL1/MPIG6B protein level ratio	GCST90315073
rs3755259	4.00E-67	HPCAL1	HPCAL1/LAT protein level ratio	GCST90315072
rs12990115	2.00E-66	CYS1,KLF11	Height	GCST90662911
rs3755259	3.00E-66	HPCAL1	CHMP1A/HPCAL1 protein level ratio	GCST90314049
rs3755259	4.00E-61	HPCAL1	HPCAL1/PMVK protein level ratio	GCST90315076
rs3755259	9.00E-56	HPCAL1	HPCAL1/S100A4 protein level ratio	GCST90315078
rs7597744	1.00E-53	RN7SL66P,RRM2	Height	GCST90245848
rs7556858	2.00E-52	RRM2,RN7SL66P	Height	GCST90245848
rs10165255	1.00E-45	CYS1	Height	GCST007841
rs3755259	2.00E-42	HPCAL1	HPCAL1/SERPINB9 protein level ratio	GCST90315079
rs575837367	4.00E-37	HPCAL1	Calcium levels (UKB data field 30680)	GCST90468065
rs3755259	1.00E-35	HPCAL1	DAPP1/HPCAL1 protein level ratio	GCST90314386
rs56003766	4.00E-35	HPCAL1	HPCAL1 protein levels	GCST90469471
rs10165255	1.00E-34	CYS1	Height (baseline)	GCST90565843
rs12990115	2.00E-34	CYS1,KLF11	Height	GCST90435412
rs6717452	3.00E-31	CYS1	Height	GCST90018959
rs6721191	4.00E-30	KLF11	Height	GCST90245844
rs3885668	1.00E-29	KLF11,GRHL1	Body size or adipose distribution (multivariate analysis)	GCST90624105
rs4482455	1.00E-29	GRHL1	CA6 protein levels	GCST90468515
rs575837367	7.00E-29	HPCAL1	Calcium levels	GCST90018951
rs112107359	4.00E-25	HPCAL1	Bone mineral density mean	GCST90321120
rs1861230	8.00E-22	HPCAL1	HPCAL1 protein levels	GCST90469471
rs6721191	1.00E-21	KLF11	Height (maximum, inv-normal transformed)	GCST90479634
rs6721191	3.00E-21	KLF11	height (mean, inv-normal transformed)	GCST90479635
rs73913932	8.00E-20	GRHL1	Prostate cancer	GCST90274713
rs34153848	1.00E-19	TAF1B	SCGB3A2 protein levels	GCST90470543
rs10171807	4.00E-19	CYS1	Physical function (baseline)	GCST90565837
rs10184715	2.00E-18	RNU4-73P,TAF1B	Calcium levels (UKB data field 30680)	GCST90468065
rs6721191	6.00E-18	KLF11	height (minimum, inv-normal transformed)	GCST90479636
rs891499	2.00E-16	GRHL1	DNER protein levels	GCST90469019
rs559072575	2.00E-16	GRHL1,KLF11	GLIPR1 protein levels	GCST90469357
rs73913932	3.00E-16	GRHL1	Prostate cancer	GCST90274714
rs13029882	4.00E-16	GRHL1,KLF11	Calcium levels (UKB data field 30680)	GCST90468065
rs6717452	2.00E-15	CYS1	Body size (confirmatory factor analysis Factor 21)	GCST90309355
rs6721191	2.00E-15	KLF11	Height	GCST90245845
rs56242692	4.00E-15	CYS1	Atrial fibrillation	GCST90624411
rs115866594	9.00E-15	RRM2,RN7SL66P	Height	GCST90435412
rs6745337	1.00E-14	RRM2	Height	GCST90245848
rs56242692	2.00E-14	CYS1	Atrial fibrillation	GCST90624412
rs4669522	2.00E-14	KLF11	Heel bone mineral density	GCST006433
rs4396680	5.00E-14	GRHL1,KLF11	Atrial fibrillation	GCST90559230
rs4669522	8.00E-14	KLF11	Heel bone mineral density	GCST006979
rs139539100	1.00E-13	HPCAL1	Femur bone mineral density x serum urate levels interaction	GCST012490
rs115866594	1.00E-13	RRM2,RN7SL66P	Height (baseline)	GCST90565843
rs6721191	1.00E-13	KLF11	What is your height? (cm, inv-normal transformed)	GCST90479637
rs115866594	2.00E-13	RRM2,RN7SL66P	Height	GCST007841
rs2463463	2.00E-13	ODC1,HPCAL1	Height	GCST90245848
rs58248202	3.00E-13	HPCAL1,ODC1	GLIPR1 protein levels	GCST90469357
rs15380	7.00E-13	CYS1	Heel bone mineral density	GCST007066
rs3791758	8.00E-13	GRHL1	Urea levels (UKB data field 30670)	GCST90468108
rs7632	2.00E-12	KLF11	Serum urate levels	GCST90455669
rs10165255	4.00E-12	CYS1	Gout	GCST90455677
rs41264179	4.00E-12	GRHL1	HEPACAM2 protein levels	GCST90469445
rs62127532	5.00E-12	KLF11	Red blood cell count	GCST90002363
rs62127532	1.00E-11	KLF11	Red blood cell count	GCST90002367
rs6432055	1.00E-11	CYS1,KLF11	Serum urate levels	GCST90319904
rs145647824	1.00E-11	HPCAL1	GLIPR1 protein levels	GCST90469357
rs3791760	4.00E-11	GRHL1	Blood urea nitrogen (BUN, mean, inv-norm transformed)	GCST90479525
rs6758955,rs8183625	6.00E-11	HPCAL1 x EFCAB8	Diffuse plaques (SNP x SNP interaction)	GCST010341
rs57909992	9.00E-11	GRHL1,KLF11	Calcium levels	GCST90019500
rs139215025	9.00E-11	RN7SL66P,RRM2	Lung function (FEV1/FVC)	GCST006482
rs3791760	1.00E-10	GRHL1	Blood urea nitrogen levels	GCST008062
rs7558744	1.00E-10	RRM2	Body mass index	GCST90662912
rs3791760	2.00E-10	GRHL1	Blood urea nitrogen levels	GCST90018948
rs112040334	2.00E-10	KLF11	QT interval	GCST90179153
rs3885668	3.00E-10	KLF11,GRHL1	Mean spheric corpuscular volume	GCST90002397
rs6741117	3.00E-10	GRHL1	Basal cell carcinoma	GCST90328134
rs1437919,rs10978043	3.00E-10	CPS1 - RPS27P10 x PTPRD	Monoclonal gammopathy of undetermined significance	GCST006917

rs6433971,rs16959241	3.00E-10	Y_RNA - FMNL2 x GAS7	Total PHF-tau (SNP x SNP interaction)	GCST010340
rs113577745	4.00E-10	GRHL1	Breast cancer	GCST004988
rs4396680	4.00E-10	GRHL1,KLF11	Neuroticism	GCST006476
rs6432053	4.00E-10	KLF11	Red blood cell count	GCST90662905
rs1437919,rs7851513	4.00E-10	CPS1 - RPS27P10 x PTPRD	Monoclonal gammopathy of undetermined significance	GCST006917
rs113577745	5.00E-10	GRHL1	Breast cancer	GCST90090980
rs116430522	6.00E-10	HPCAL1	Femur bone mineral density x serum urate levels interaction	GCST012490
rs73913932	6.00E-10	GRHL1	Prostate cancer	GCST011049
rs75921737	6.00E-10	HPCAL1	Bone mineral density variability	GCST90321121
rs2430420	7.00E-10	HPCAL1,ODC1	Hippocalcin-like protein 1 levels	GCST90247923
rs112040334	7.00E-10	KLF11	QT interval	GCST90179154
rs10165255	1.00E-09	CYS1	Red blood cell count	GCST007069
rs11674455	1.00E-09	KLF11,GRHL1	Height (baseline)	GCST90565843
rs6726950	1.00E-09	GRHL1	Basal cell carcinoma	GCST90328132
rs6432053	2.00E-09	KLF11	Red blood cell count	GCST90018971
rs10165255	3.00E-09	CYS1	Gout	GCST90455676
rs546196413	3.00E-09	TAF1B	Bone mineral density variability	GCST90321121
rs4396680	4.00E-09	GRHL1,KLF11	Neurociticism	GCST006940
rs6717452	4.00E-09	CYS1	Forced expiratory volume (baseline)	GCST90565844
rs4396680	5.00E-09	GRHL1,KLF11	Neuroticism	GCST007084
rs287982	6.00E-09	RNU4-73P,TAF1B	Nonsyndromic cleft lip with cleft palate	GCST004166
rs11686949	6.00E-09	HPCAL1	Platelet distribution width	GCST90002401
rs56277911	7.00E-09	RRM2,RN7SL66P	Body surface area	GCST90245995
rs56277911	8.00E-09	RRM2,RN7SL66P	Menarche (age at onset)	GCST007078
rs6432054	8.00E-09	KLF11	Serum urate levels	GCST90319906
rs70948864	9.00E-09	GRHL1,KLF11	Mean corpuscular volume	GCST90018966
rs4396680	1.00E-08	GRHL1,KLF11	Feeling worry	GCST006950
rs6432054	1.00E-08	KLF11	Waist circumference adjusted for body mass index	GCST009867
rs4396680	1.00E-08	GRHL1,KLF11	Height (standard GWA)	GCST90267284
rs2303914	2.00E-08	TAF1B	Serum urate levels	GCST90455669
rs114034700	2.00E-08	TAF1B,GRHL1	Bone mineral density variability	GCST90321121
rs145793974	2.00E-08	KLF11	Height	GCST90278637
rs887959	2.00E-08	RN7SL66P,RRM2	Gut microbial network clusters (BlueViolet (at 3 months) x Homesmokers interaction	GCST90569276
rs6721191	2.00E-08	KLF11	Height	GCST90245846
rs11902236	3.00E-08	GRHL1	Prostate cancer	GCST001942
rs12692398	3.00E-08	CYS1	Chronic bronchitis and chronic obstructive pulmonary disease	GCST002625
rs6713406	3.00E-08	KLF11,GRHL1	DNA methylation variation (age effect)	GCST006660
rs145793974	3.00E-08	KLF11	Cystatin C levels in bottom 99% of individuals by creatinine levels	GCST90566734
rs73913932	4.00E-08	GRHL1	Type 2 diabetes or prostate cancer (pleiotropy)	GCST011320
rs6432054	4.00E-08	KLF11	Subcortical volume (MOSTest)	GCST010702
rs4396680	4.00E-08	GRHL1,KLF11	Neuroticism general factor	GCST90428866
rs57909992	4.00E-08	GRHL1,KLF11	Blood urea nitrogen levels	GCST90278623
rs12539892,rs42833	4.00E-08	PER3P1 - U3 x LINC01122	Total PHF-tau (SNP x SNP interaction)	GCST010340

Supplemental Table 7. Genome-wide significant associations reported in the GWAS catalog within hg19 chr4:79,880,102-81,206,182 (hg38 chr4:78,958,947-80,285,028).

Variant	P Value	Mapped Genes	Reported Trait	Study Accession
rs116128373	1.00E-300	LINC01088,NAA11	Bone mineral density mean	GCST90321120
rs17004869	6.00E-236	FGF5	FGF5 protein levels	GCST90469226
rs36077257	9.00E-226	FGF5,PRDM8	FGF5 protein levels	GCST90469226
rs13125101	7.00E-165	PRDM8,FGF5	Diastolic blood pressure	GCST90310295
rs13149993	8.00E-163	PRDM8,FGF5	Pulse pressure x alcohol consumption interaction (2df test)	GCST006168
rs13125101	1.00E-152	PRDM8,FGF5	Systolic blood pressure	GCST90310294
rs79059900	2.00E-148	ANTXR2,RPSAP39	FGF5 protein levels	GCST90469226
rs13125101	8.00E-139	PRDM8,FGF5	Diastolic blood pressure (MTAG)	GCST90449057
rs12509595	8.00E-133	PRDM8,FGF5	Systolic blood pressure (MTAG)	GCST90449056
rs10857147	4.00E-121	PRDM8,FGF5	Hypertension	GCST90244037
rs16998073	6.00E-115	PRDM8,FGF5	Diastolic blood pressure	GCST90244036
rs13125101	3.00E-114	PRDM8,FGF5	Systolic blood pressure	GCST007087
rs16998073	5.00E-111	PRDM8,FGF5	Systolic blood pressure	GCST90244038
rs33989035	1.00E-110	FGF5,PRDM8	FGF5 protein levels	GCST90469226
rs12509595	1.00E-101	PRDM8,FGF5	Diastolic blood pressure	GCST90132904
rs16998073	3.00E-96	PRDM8,FGF5	Mean arterial pressure (BMI adjusted)	GCST90335169
rs16998073	2.00E-93	PRDM8,FGF5	Diastolic blood pressure (BMI adjusted)	GCST90335165
rs13125101	1.00E-92	PRDM8,FGF5	High blood pressure / hypertension	GCST90475214
rs12509595	9.00E-91	PRDM8,FGF5	Systolic blood pressure	GCST90435415
rs12509595	1.00E-88	PRDM8,FGF5	Hypertension	GCST90468135
rs35662177	3.00E-87	FGF5	FGF5 protein levels	GCST90469226
rs12509595	2.00E-86	PRDM8,FGF5	Systolic blood pressure	GCST90132903
rs34783620	2.00E-85	FGF5	FGF5 protein levels	GCST90469226
rs16998073	9.00E-85	PRDM8,FGF5	Mean arterial pressure (BMI unadjusted)	GCST90335168
rs16998073	6.00E-84	PRDM8,FGF5	Diastolic blood pressure (BMI unadjusted)	GCST90335164
rs12509595	2.00E-83	PRDM8,FGF5	Essential hypertension (PheCode 401.1)	GCST90475922
rs12509595	2.00E-82	PRDM8,FGF5	Medication use for hypertension (number of purchases)	GCST90250905
rs12509595	3.00E-82	PRDM8,FGF5	Takes medication for high blood pressure?	GCST90475216
rs13125101	1.00E-80	PRDM8,FGF5	Cardiovascular disease	GCST007072
rs1458038	1.00E-80	PRDM8,FGF5	Systolic blood pressure (cigarette smoking interaction)	GCST006188
rs12509595	3.00E-80	PRDM8,FGF5	Hypertension (PheCode 401)	GCST90475919
rs1458038	7.00E-80	PRDM8,FGF5	Diastolic blood pressure (cigarette smoking interaction)	GCST006187
rs12509595	4.00E-79	PRDM8,FGF5	Diastolic blood pressure	GCST90435414
rs12509595	4.00E-76	PRDM8,FGF5	Systolic blood pressure (BMI adjusted)	GCST90335163
rs12509595	6.00E-75	PRDM8,FGF5	Systolic blood pressure	GCST90018972
rs12509595	2.00E-74	PRDM8,FGF5	Medication use (agents acting on the renin-angiotensin system)	GCST90018988
rs10857147	2.00E-74	PRDM8,FGF5	Mean arterial pressure	GCST90018963
rs12509595	2.00E-73	PRDM8,FGF5	Systolic blood pressure x alcohol consumption interaction (2df test)	GCST006434
rs13125101	4.00E-70	PRDM8,FGF5	Medication use (agents acting on the renin-angiotensin system)	GCST007930
rs148192485	7.00E-68	FGF5,PRDM8	FGF5 protein levels	GCST90469226
rs12509595	5.00E-67	PRDM8,FGF5	Systolic blood pressure (BMI unadjusted)	GCST90335162
rs12509595	3.00E-65	PRDM8,FGF5	Diastolic blood pressure x alcohol consumption interaction (2df test)	GCST006166
rs13125101	3.00E-65	PRDM8,FGF5	diastolic blood pressure (DBP, mean, inv-normal transformed)	GCST90475255
rs10857147	3.00E-64	PRDM8,FGF5	Systolic blood pressure	GCST007267
rs16998073	1.00E-61	PRDM8,FGF5	creatinine (mean, inv-norm transformed)	GCST90475229
rs12509595	2.00E-61	PRDM8,FGF5	Diastolic blood pressure	GCST007094
rs16998073	2.00E-61	PRDM8,FGF5	Systolic blood pressure	GCST90278642
rs16998073	9.00E-60	PRDM8,FGF5	Diastolic blood pressure	GCST90018952
rs16998073	1.00E-59	PRDM8,FGF5	Diastolic blood pressure	GCST007094
rs1458038	1.00E-59	PRDM8,FGF5	Diastolic blood pressure	GCST90301698
rs12509595	3.00E-59	PRDM8,FGF5	Diastolic blood pressure	GCST90278625
rs12509595	5.00E-58	PRDM8,FGF5	diastolic blood pressure (DBP, maximum, inv-normal transformed)	GCST90475252
rs16998073	6.00E-58	PRDM8,FGF5	creatinine (minimum, inv-norm transformed)	GCST90475232
rs13125101	2.00E-57	PRDM8,FGF5	High blood pressure / hypertension	GCST90479553
rs62300379	5.00E-57	FGF5,PRDM8	FGF5 protein levels	GCST90469226
rs139246187I	3.00E-55	ANTXR2	Lymphocyte count	GCST90002316
rs12509595	5.00E-55	PRDM8,FGF5	Essential hypertension (PheCode 401.1)	GCST90480125
rs12509595	9.00E-55	PRDM8,FGF5	Systolic blood pressure	GCST007099
rs13125101	1.00E-53	PRDM8,FGF5	Hypertension (PheCode 401)	GCST90480128
rs16998073	2.00E-53	PRDM8,FGF5	Systolic blood pressure	GCST007099
rs13125101	2.00E-53	PRDM8,FGF5	estimated glomerular filtration rate (eGFR, mean, inv-norm transformed)	GCST90475282
rs114100453	4.00E-53	PRDM8,FGF5	FGF5 protein levels	GCST90469226
rs11099097	6.00E-53	PRDM8,FGF5	Systolic blood pressure (UKB data field 4080)	GCST90468179
rs7681907	6.00E-51	FGF5	Male-pattern baldness	GCST007020
rs16998073	2.00E-50	PRDM8,FGF5	Hypertension (PheCode 401)	GCST90651143
rs9996178	4.00E-50	LINC02469,PCAT4	Anthrax toxin receptor 2 levels	GCST90246517
rs13125101	6.00E-50	PRDM8,FGF5	Takes medication for high blood pressure?	GCST90479552
rs13125101	7.00E-50	PRDM8,FGF5	Pulse pressure	GCST90310296
rs3796604	1.00E-49	FGF5	Balding type 1	GCST007038
rs13125101	5.00E-49	PRDM8,FGF5	Hypertension (confirmatory factor analysis Factor 12)	GCST90309346
rs13125101	8.00E-49	PRDM8,FGF5	diastolic blood pressure (DBP, mean, inv-normal transformed)	GCST90479581
rs11099097	2.00E-48	PRDM8,FGF5	Hypertension	GCST90446531
rs59133384	4.00E-48	PRDM8,FGF5	FGF5 protein levels	GCST90469226
rs11099097	5.00E-48	PRDM8,FGF5	Systolic blood pressure	GCST90301695

rs16998073	6.00E-48	PRDM8,FGF5	creatinine (maximum, inv-norm transformed)	GCST90475226
rs62300384	2.00E-47	FGF5,PRDM8	FGF5 protein levels	GCST90469226
rs16998073	2.00E-47	PRDM8,FGF5	Essential hypertension (PheCode 401.1)	GCST90651145
rs13125101	2.00E-47	PRDM8,FGF5	estimated glomerular filtration rate (eGFR, maximum, inv-norm transformed)	GCST90475280
rs4596195	3.00E-47	ANTXR2	Lymphocyte count	GCST90002388
rs10027407	3.00E-47	ANTXR2	Lymphocyte count (UKB data field 30120)	GCST90468082
rs16998073	4.00E-47	PRDM8,FGF5	Mean arterial pressure	GCST90239619
rs12509595	7.00E-47	PRDM8,FGF5	diastolic blood pressure (DBP, maximum, inv-normal transformed)	GCST90479580
rs11099097	2.00E-46	PRDM8,FGF5	Systolic blood pressure x alcohol consumption interaction (2df test)	GCST006434
rs12509595	5.00E-45	PRDM8,FGF5	Red blood cell count	GCST90002367
rs16998073	1.00E-44	PRDM8,FGF5	Mean arterial pressure	GCST007706
rs16998073	2.00E-44	PRDM8,FGF5	Systolic blood pressure	GCST90239615
rs11098925	5.00E-44	PCAT4	Anthrax toxin receptor 2 levels	GCST90246517
rs16998073	6.00E-43	PRDM8,FGF5	Diastolic blood pressure	GCST90239616
rs150172324	8.00E-43	PRDM8,PRDM8-AS1	FGF5 protein levels	GCST90469226
rs12509595	1.00E-42	PRDM8,FGF5	Medication use (calcium channel blockers)	GCST90018987
rs1458038	2.00E-42	PRDM8,FGF5	estimated glomerular filtration rate (eGFR, minimum, inv-norm transformed)	GCST90475285
rs11099097	6.00E-42	PRDM8,FGF5	Diastolic blood pressure (UKB data field 4079)	GCST90468163
rs12509595	1.00E-41	PRDM8,FGF5	systolic blood pressure (SBP, mean, inv-normal transformed)	GCST90476403
rs12509595	5.00E-41	PRDM8,FGF5	Estimated glomerular filtration rate (creatinine)	GCST90100220
rs16998073	6.00E-41	PRDM8,FGF5	Systolic blood pressure	GCST007703
rs12509595	1.00E-39	PRDM8,FGF5	potassium (mean, inv-norm transformed)	GCST90476307
rs28645467	3.00E-39	ANTXR2	lymphocyte (absolute count, maximum, inv-norm transformed)	GCST90475423
rs12509595	4.00E-39	PRDM8,FGF5	systolic blood pressure (SBP, maximum, inv-normal transformed)	GCST90476400
rs16998073	5.00E-39	PRDM8,FGF5	Red blood cell count	GCST90002363
rs10012656	1.00E-38	ANTXR2	Lymphocyte count	GCST90018962
rs10857147	2.00E-38	PRDM8,FGF5	Estimated glomerular filtration rate (creatinine)	GCST90103633
rs16998073	3.00E-38	PRDM8,FGF5	Diastolic blood pressure	GCST007704
rs13125101	3.00E-38	PRDM8,FGF5	Atrial fibrillation	GCST90624411
rs12509595	2.00E-37	PRDM8,FGF5	Estimated glomerular filtration rate	GCST008058
rs10857147	7.00E-37	PRDM8,FGF5	Medication use (calcium channel blockers)	GCST007929
rs4690134	3.00E-36	ANTXR2	lymphocyte (absolute count, maximum, inv-norm transformed)	GCST90479663
rs10857147	3.00E-36	PRDM8,FGF5	creatinine (minimum, inv-norm transformed)	GCST90479563
rs3796606	7.00E-36	FGF5	Height	GCST90245848
rs16998073	1.00E-35	PRDM8,FGF5	Hypertensive heart disease (PheCode 401.21)	GCST90651158
rs10857147	2.00E-35	PRDM8,FGF5	creatinine (mean, inv-norm transformed)	GCST90479562
rs10857147	3.00E-35	PRDM8,FGF5	Hypertensive heart and/or renal disease (PheCode 401.2)	GCST90651159
rs11099098	4.00E-35	FGF5,PRDM8	Atrial fibrillation	GCST90624412
rs3796605	6.00E-35	FGF5	FGF5 protein levels	GCST90469226
rs72658365	1.00E-34	FGF5	Male-pattern baldness	GCST007020
rs12509595	1.00E-34	PRDM8,FGF5	potassium (mean, inv-norm transformed)	GCST90480654
rs4610280	2.00E-34	ANTXR2	lymphocyte (absolute count, mean, inv-norm transformed)	GCST90479664
rs10027415	3.00E-34	ANTXR2	Lymphocyte count	GCST90002320
rs1458038	4.00E-34	PRDM8,FGF5	Diastolic blood pressure	GCST90301700
rs16998073	8.00E-34	PRDM8,FGF5	FGF5 protein levels	GCST90277792
rs13125101	1.00E-33	PRDM8,FGF5	Mean arterial pressure x alcohol consumption interaction (2df test)	GCST006167
rs10857147	1.00E-33	PRDM8,FGF5	Pulse pressure	GCST90018970
rs7658439	4.00E-33	PRDM8,FGF5	FGF5 protein levels	GCST90469226
rs12509595	7.00E-33	PRDM8,FGF5	systolic blood pressure (SBP, mean, inv-normal transformed)	GCST90480706
rs10857147	8.00E-33	PRDM8,FGF5	Mean arterial pressure	GCST007706
rs34383234	9.00E-33	FGF5	FGF5 protein levels	GCST90469226
rs1458038	1.00E-32	PRDM8,FGF5	Hematocrit	GCST90002304
rs10857147	1.00E-32	PRDM8,FGF5	Medication use (diuretics)	GCST007928
rs13125101	3.00E-32	PRDM8,FGF5	Hematocrit	GCST90002308
rs13125101	6.00E-32	PRDM8,FGF5	Hemoglobin concentration	GCST90002310
rs10857147	8.00E-32	PRDM8,FGF5	Mean arterial pressure	GCST90018743
rs10857147	9.00E-32	PRDM8,FGF5	Systolic blood pressure	GCST007703
rs537655243	9.00E-32	FGF5,PRDM8	FGF5 protein levels	GCST90469226
rs13125101	2.00E-31	PRDM8,FGF5	Hemoglobin concentration	GCST90002314
rs10857147	2.00E-31	PRDM8,FGF5	Systolic blood pressure	GCST90018752
rs28645467	2.00E-31	ANTXR2	lymphocyte (absolute count, mean, inv-norm transformed)	GCST90475426
rs545692945	4.00E-31	FGF5,PRDM8	FGF5 protein levels	GCST90469226
rs10857147	5.00E-31	PRDM8,FGF5	Medication use (beta blocking agents)	GCST90018986
rs16998073	2.00E-30	PRDM8,FGF5	Hypertension	GCST009685
rs12509595	3.00E-30	PRDM8,FGF5	systolic blood pressure (SBP, maximum, inv-normal transformed)	GCST90480705
rs1458046	8.00E-30	FGF5	Hair color	GCST007082
rs10857147	9.00E-30	PRDM8,FGF5	estimated glomerular filtration rate (eGFR, mean, inv-norm transformed)	GCST90479599
rs16998073	3.00E-29	PRDM8,FGF5	Hypertension	GCST007707
rs11099097	4.00E-29	PRDM8,FGF5	Systolic blood pressure	GCST90301696
rs1458038	4.00E-29	PRDM8,FGF5	Diastolic blood pressure	GCST90301699
rs1458038	9.00E-29	PRDM8,FGF5	Estimated glomerular filtration rate	GCST008059
rs10857147	1.00E-28	PRDM8,FGF5	estimated glomerular filtration rate (eGFR, maximum, inv-norm transformed)	GCST90479598
rs1458038	4.00E-28	PRDM8,FGF5	creatinine (maximum, inv-norm transformed)	GCST90479561
rs12510805	5.00E-28	ANTXR2	Lymphocyte percentage of white cells	GCST90002389
rs11099098	8.00E-28	FGF5,PRDM8	Mean arterial pressure	GCST007706
rs10857147	2.00E-27	PRDM8,FGF5	Mean arterial pressure	GCST006010
rs11099098	4.00E-27	FGF5,PRDM8	Systolic blood pressure	GCST007703

rs1458038	4.00E-27	PRDM8,FGF5	Mean arterial pressure	GCST007706
rs7680591	1.00E-26	FGF5	Male-pattern baldness	GCST006661
rs76451811	1.00E-26	PRDM8-AS1,RPSAP39	FGF5 protein levels	GCST90469226
rs10857147	2.00E-26	PRDM8,FGF5	Diastolic blood pressure	GCST007704
rs10857147	2.00E-26	PRDM8,FGF5	Serum creatinine levels	GCST90018979
rs13125101	2.00E-26	PRDM8,FGF5	Medication use (beta blocking agents)	GCST007927
rs1458038	3.00E-26	PRDM8,FGF5	Systolic blood pressure	GCST007703
rs16998073	3.00E-26	PRDM8,FGF5	Hypertension	GCST90239614
rs16998073	6.00E-26	PRDM8,FGF5	Biological age (BioAge)	GCST90104396
rs1458038	7.00E-26	PRDM8,FGF5	Estimated glomerular filtration rate (creatinine)	GCST90103634
rs10857147	1.00E-25	PRDM8,FGF5	Systolic blood pressure	GCST005979
rs11946402	1.00E-25	ANTXR2	Lymphocyte percentage (UKB data field 30180)	GCST90468083
rs13125101	2.00E-25	PRDM8,FGF5	Red blood cell count	GCST007069
rs13125101	2.00E-25	PRDM8,FGF5	Red blood cell count	GCST90002403
rs10857147	2.00E-25	PRDM8,FGF5	Diastolic blood pressure	GCST90018732
rs13125101	2.00E-25	PRDM8,FGF5	Red blood cell erythrocyte count (UKB data field 30010)	GCST90468098
rs28645467	2.00E-25	ANTXR2	lymphocyte (fraction, maximum, inv-norm transformed)	GCST90475432
rs13125101	2.00E-25	PRDM8,FGF5	Blood urea nitrogen (BUN, mean, inv-norm transformed)	GCST90475165
rs7680591	5.00E-25	FGF5	Male-pattern baldness	GCST005116
rs16998073	8.00E-25	PRDM8,FGF5	Hypertension	GCST011141
rs1458038	8.00E-25	PRDM8,FGF5	estimated glomerular filtration rate (eGFR, minimum, inv-norm transformed)	GCST90479600
rs1458038	9.00E-25	PRDM8,FGF5	Diastolic blood pressure	GCST001228
rs12509595	9.00E-25	PRDM8,FGF5	Pulse pressure (BMI adjusted)	GCST90335167
rs12509595	1.00E-24	PRDM8,FGF5	Medication use (diuretics)	GCST90018985
rs12509595	4.00E-24	PRDM8,FGF5	Diastolic blood pressure	GCST007098
rs12509595	4.00E-24	PRDM8,FGF5	potassium (minimum, inv-norm transformed)	GCST90476310
rs1458038	5.00E-24	PRDM8,FGF5	Systolic blood pressure	GCST006259
rs16998073	5.00E-24	PRDM8,FGF5	ryptic phenotype that captures autosomal dominant polycystic kidney disease severit	GCST90101829
rs16998073	9.00E-24	PRDM8,FGF5	Diastolic blood pressure	GCST007098
rs10857147	9.00E-24	PRDM8,FGF5	Red blood cell count	GCST90018971
rs10857147	2.00E-23	PRDM8,FGF5	Hypertension	GCST007707
rs1458038	2.00E-23	PRDM8,FGF5	Systolic blood pressure	GCST001227
rs10857147	2.00E-23	PRDM8,FGF5	Pulse pressure	GCST90132905
rs13125101	2.00E-23	PRDM8,FGF5	potassium (minimum, inv-norm transformed)	GCST90480655
rs16998073	3.00E-23	PRDM8,FGF5	Diastolic blood pressure	GCST006020
rs62298617	3.00E-23	ANTXR2	Lymphocyte count	GCST90085815
rs56164535	4.00E-23	FGF5,PRDM8	Estimated glomerular filtration rate (creatinine, cystatin c)	GCST90428446
rs12509595	6.00E-23	PRDM8,FGF5	Pulse pressure (BMI unadjusted)	GCST90335166
rs12504282	7.00E-23	ANTXR2	Lymphocyte count	GCST004627
rs1458038	7.00E-23	PRDM8,FGF5	Diastolic blood pressure	GCST006258
rs7683390	7.00E-23	FGF5	Diastolic blood pressure (MTAG)	GCST90449057
rs1902859	2.00E-22	PRDM8,FGF5	Systolic blood pressure	GCST002630
rs10857147	2.00E-22	PRDM8,FGF5	Diastolic blood pressure	GCST005978
rs11099098	3.00E-22	FGF5,PRDM8	Diastolic blood pressure	GCST007704
rs1458038	3.00E-22	PRDM8,FGF5	Hypertension	GCST011141
rs28464573	4.00E-22	ANTXR2	lymphocyte (fraction, maximum, inv-norm transformed)	GCST90479666
rs9783424,rs10022	4.00E-22	PPP1R12A x LINC02431 - MIR6082	Total PHF-tau (SNP x SNP interaction)	GCST010340
rs16998073	6.00E-22	PRDM8,FGF5	Fibroblast growth factor 5 levels	GCST90000469
rs1458038	9.00E-22	PRDM8,FGF5	Diastolic blood pressure	GCST007704
rs11099097	9.00E-22	PRDM8,FGF5	Systolic blood pressure	GCST90301697
rs12509595	1.00E-21	PRDM8,FGF5	Potassium levels	GCST006031
rs16998073	1.00E-21	PRDM8,FGF5	Diastolic blood pressure	GCST000394
rs11099098	2.00E-21	FGF5,PRDM8	Hypertension	GCST007707
rs11099098	2.00E-21	FGF5,PRDM8	Hematocrit	GCST90002383
rs28643281	5.00E-21	FGF5,PRDM8	FGF5 protein levels	GCST90469226
rs10857147	6.00E-21	PRDM8,FGF5	Serum uric acid levels	GCST90018977
rs13149993	1.00E-20	PRDM8,FGF5	Mean arterial pressure	GCST007706
rs16998073	1.00E-20	PRDM8,FGF5	Other hypertensive complications (PheCode 401.3)	GCST90651186
rs12509595	1.00E-20	PRDM8,FGF5	Atrial fibrillation	GCST90559230
rs56164535	1.00E-20	FGF5,PRDM8	Estimated glomerular filtration rate (creatinine)	GCST90428447
rs1458038	2.00E-20	PRDM8,FGF5	Hypertension	GCST007707
rs12509595	2.00E-20	PRDM8,FGF5	Estimated glomerular filtration rate	GCST008747
rs13149993	3.00E-20	PRDM8,FGF5	Systolic blood pressure	GCST007703
rs10857147	3.00E-20	PRDM8,FGF5	Hemoglobin	GCST90002384
rs1902859	4.00E-20	PRDM8,FGF5	Diastolic blood pressure	GCST002631
rs16998073	5.00E-20	PRDM8,FGF5	Creatinine levels (UKB data field 30700)	GCST90468067
rs13125101	6.00E-20	PRDM8,FGF5	chloride (mean, inv-norm transformed)	GCST90479540
rs1902859	8.00E-20	PRDM8,FGF5	Mean arterial pressure	GCST007706
rs13125101	9.00E-20	PRDM8,FGF5	Blood urea nitrogen (BUN, minimum, inv-norm transformed)	GCST90475168
rs1902859	1.00E-19	PRDM8,FGF5	Systolic blood pressure	GCST007703
rs12510805	1.00E-19	ANTXR2	Neutrophils and lymphocytes in blood (confirmatory factor analysis Factor 35)	GCST90309369
rs12510805	1.00E-19	ANTXR2	lymphocyte (absolute count, minimum, inv-norm transformed)	GCST90479665
rs12509595	1.00E-19	PRDM8,FGF5	chloride (mean, inv-norm transformed)	GCST90475194
rs12509595	2.00E-19	PRDM8,FGF5	Systolic blood pressure	GCST007095
rs181409212	2.00E-19	FGF5	FGF5 protein levels	GCST90469226
rs16998073	4.00E-19	PRDM8,FGF5	Systolic blood pressure	GCST007095
rs56190605	4.00E-19	FGF5,PRDM8	Systolic blood pressure (MTAG)	GCST90449056

rs11946205	5.00E-19	ANTXR2	Neutrophil percentage of white cells	GCST90002399
rs16998073	6.00E-19	PRDM8,FGF5	Coronary artery disease	GCST010866
rs16998073	6.00E-19	PRDM8,FGF5	Preeclampsia or other maternal hypertension (maternal genotype effect)	GCST90269904
rs1458046	8.00E-19	FGF5	Blond vs. brown/black hair color	GCST006988
rs10857147	1.00E-18	PRDM8,FGF5	Serum urate levels	GCST90319904
rs11099098	2.00E-18	FGF5,PRDM8	Haemoglobin concentration (UKB data field 30020)	GCST90468074
rs13125101	2.00E-18	PRDM8,FGF5	potassium (maximum, inv-norm transformed)	GCST90480653
rs4355335	3.00E-18	ANTXR2	Platelet-to-lymphocyte ratio	GCST90056184
rs12645809	3.00E-18	ANTXR2	Height	GCST90245848
rs1902859	8.00E-18	PRDM8,FGF5	Hypertension	GCST002627
rs11946205	9.00E-18	ANTXR2	Neutrophill percentage (UKB data field 30200)	GCST90468093
rs7662551	1.00E-17	LINC00989	Spherical equivalent or myopia (age of diagnosis)	GCST006291
rs565853247	2.00E-17	PRDM8,FGF5	Diastolic blood pressure	GCST007268
rs16998073	2.00E-17	PRDM8,FGF5	Diastolic blood pressure	GCST007098
rs12509595	2.00E-17	PRDM8,FGF5	Diastolic blood pressure	GCST007098
rs13149993	2.00E-17	PRDM8,FGF5	Mean arterial pressure	GCST003275
rs28645467	2.00E-17	ANTXR2	neutrophil (fraction, minimum, inv-norm transformed)	GCST90475541
rs6842105	3.00E-17	PCAT4,ANTXR2	Spherical equivalent	GCST010378
rs10857147	4.00E-17	PRDM8,FGF5	Serum creatinine levels	GCST90018759
rs16998073	4.00E-17	PRDM8,FGF5	hemoglobin (maximum, inv-norm transformed)	GCST90475372
rs1458038	8.00E-17	PRDM8,FGF5	Diastolic blood pressure	GCST004777
rs11099098	8.00E-17	FGF5,PRDM8	chloride (maximum, inv-norm transformed)	GCST90479539
rs1458038	8.00E-17	PRDM8,FGF5	Blood urea nitrogen (BUN, maximum, inv-norm transformed)	GCST90475162
rs1458038	9.00E-17	PRDM8,FGF5	Mean arterial pressure x depressive symptoms interaction (2df test)	GCST90093273
rs1458038	1.00E-16	PRDM8,FGF5	Systolic blood pressure	GCST004776
rs13149993	2.00E-16	PRDM8,FGF5	Diastolic blood pressure	GCST007704
rs10857147	2.00E-16	PRDM8,FGF5	Creatinine levels	GCST005985
rs10857147	2.00E-16	PRDM8,FGF5	Creatinine levels in bottom 99% of individuals by creatinine levels	GCST90566733
rs12509595	3.00E-16	PRDM8,FGF5	Pulse pressure	GCST007096
rs11099097	3.00E-16	PRDM8,FGF5	Mean arterial pressure x alcohol consumption (light vs heavy) interaction (2df test)	GCST006172
rs56164535	3.00E-16	FGF5,PRDM8	Estimated glomerular filtration rate (cystatin c)	GCST90428448
rs10857147	3.00E-16	PRDM8,FGF5	red blood cell count (RBC, maximum, inv-norm transformed)	GCST90476345
rs11099098	4.00E-16	FGF5,PRDM8	Blood urea nitrogen (BUN, mean, inv-norm transformed)	GCST90479525
rs12509595	5.00E-16	PRDM8,FGF5	Estimated glomerular filtration rate	GCST007344
rs11098774	6.00E-16	LINC00989	Height	GCST90245848
rs1458038	7.00E-16	PRDM8,FGF5	LDL cholesterol levels	GCST010245
rs10857147	7.00E-16	PRDM8,FGF5	Estimated glomerular filtration rate	GCST008747
rs28464573	8.00E-16	ANTXR2	neutrophil (fraction, minimum, inv-norm transformed)	GCST90479715
rs16998073	8.00E-16	PRDM8,FGF5	hematocrit (maximum, inv-norm transformed)	GCST90475338
rs16998073	9.00E-16	PRDM8,FGF5	Mean arterial pressure	GCST006231
rs10857147	9.00E-16	PRDM8,FGF5	Hemoglobin	GCST90018957
rs1902859	1.00E-15	PRDM8,FGF5	Diastolic blood pressure	GCST007704
rs16998073	1.00E-15	PRDM8,FGF5	Pulse pressure	GCST007096
rs10857147	1.00E-15	PRDM8,FGF5	Hematocrit	GCST90018960
rs139446091	1.00E-15	PRDM8,FGF5	FGF5 protein levels	GCST90469226
rs13125101	1.00E-15	PRDM8,FGF5	Bicarbonate (maximum, inv-norm transformed)	GCST90479518
rs13125101	1.00E-15	PRDM8,FGF5	Bicarbonate (mean, inv-norm transformed)	GCST90479519
rs16998073	1.00E-15	PRDM8,FGF5	Preeclampsia (maternal genotype effect)	GCST90269903
rs10857147	2.00E-15	PRDM8,FGF5	Urate levels	GCST008972
rs16998073	2.00E-15	PRDM8,FGF5	Preeclampsia or eclampsia	GCST90271316
rs16998073	2.00E-15	PRDM8,FGF5	Bicarbonate (mean, inv-norm transformed)	GCST90475148
rs13149993	3.00E-15	PRDM8,FGF5	Hypertension	GCST007707
rs13129838	3.00E-15	LINC00989	Myopia	GCST003997
rs16998073	3.00E-15	PRDM8,FGF5	Mean arterial pressure x alcohol consumption (light vs heavy) interaction (2df test)	GCST006172
rs10024534	3.00E-15	PCAT4	Histone-lysine N-methyltransferase EHMT2 levels	GCST90247415
rs11099097	4.00E-15	PRDM8,FGF5	LDL cholesterol	GCST90018961
rs11099098	4.00E-15	FGF5,PRDM8	Atrial fibrillation	GCST90204201
rs66968950	4.00E-15	ANTXR2	Educational attainment	GCST90105038
rs13125101	4.00E-15	PRDM8,FGF5	Chronic renal failure [CKD] (PheCode 585.3)	GCST90476123
rs16998073	5.00E-15	PRDM8,FGF5	Serum urate levels	GCST90455669
rs11099098	6.00E-15	FGF5,PRDM8	Atrial fibrillation and flutter (PheCode 427.2)	GCST90475967
rs9783424,rs12648	6.00E-15	PPP1R12A x RNU1-89P - TERF1P3	Total PHF-tau (SNP x SNP interaction)	GCST010340
rs77331798	7.00E-15	ANTXR2	Resistance to COVID-19 infection (Exposed negative vs positive)	GCST90255358
rs11099097	8.00E-15	PRDM8,FGF5	Diastolic blood pressure x alcohol consumption (light vs heavy) interaction (2df test)	GCST006169
rs12509595	8.00E-15	PRDM8,FGF5	Bicarbonate (maximum, inv-norm transformed)	GCST90475145
rs12509595	9.00E-15	PRDM8,FGF5	Pulse pressure	GCST007705
rs17004869	1.00E-14	FGF5	Mean arterial pressure	GCST007706
rs10857147	1.00E-14	PRDM8,FGF5	Coronary artery disease	GCST005194
rs10857147	1.00E-14	PRDM8,FGF5	Coronary artery disease	GCST005195
rs10857147	1.00E-14	PRDM8,FGF5	Coronary artery disease	GCST005196
rs11099097	1.00E-14	PRDM8,FGF5	Systolic blood pressure x alcohol consumption (light vs heavy) interaction (2df test)	GCST006170
rs28645467	1.00E-14	ANTXR2	lymphocyte (fraction, mean, inv-norm transformed)	GCST90479667
rs10857147	2.00E-14	PRDM8,FGF5	Pulse pressure	GCST007269
rs139246187	2.00E-14	ANTXR2	Lymphocyte percentage of white cells	GCST004632
rs12509595	2.00E-14	PRDM8,FGF5	Glomerular filtration rate	GCST005984
rs16998073	2.00E-14	PRDM8,FGF5	Diastolic blood pressure x alcohol consumption (light vs heavy) interaction (2df test)	GCST006169
rs1458038	2.00E-14	PRDM8,FGF5	Diastolic blood pressure	GCST003273

rs1458038	3.00E-14	PRDM8,FGF5	Blood pressure	GCST001236
rs1458038	3.00E-14	PRDM8,FGF5	hemoglobin (mean, inv-norm transformed)	GCST90475376
rs1902859	4.00E-14	PRDM8,FGF5	Hypertension	GCST007707
rs6534834	4.00E-14	PRDM8,FGF5	Diastolic blood pressure	GCST90132904
rs16998073	4.00E-14	PRDM8,FGF5	ICD10 O13: Gestational hypertension	GCST90454232
rs34805068	4.00E-14	ANTXR2	Height	GCST90245848
rs12509595	5.00E-14	PRDM8,FGF5	Chronic kidney disease	GCST008064
rs10857147	5.00E-14	PRDM8,FGF5	Serum uric acid levels	GCST90018757
rs10857147	5.00E-14	PRDM8,FGF5	Coronary artery disease	GCST90132314
rs11099098	6.00E-14	FGF5,PRDM8	Renal failure (PheCode 585)	GCST90476118
rs140082972	7.00E-14	GK2,LINC00989	CXCL10 levels	GCST90274886
rs11099098	7.00E-14	FGF5,PRDM8	Chronic Kidney Disease, Stage III (PheCode 585.33)	GCST90476128
rs12509595	1.00E-13	PRDM8,FGF5	Estimated glomerular filtration rate	GCST007876
rs11722683	1.00E-13	ANTXR2	Neutrophil-to-lymphocyte ratio	GCST90056182
rs78740643	1.00E-13	ANTXR2	Resistance to COVID-19 infection (Exposed negative vs positive)	GCST90255358
rs34207047	1.00E-13	LINC01088,NAA11	Depression	GCST90319327
rs12509595	1.00E-13	PRDM8,FGF5	chloride (minimum, inv-norm transformed)	GCST90475197
rs1458038	2.00E-13	PRDM8,FGF5	Gout	GCST90455676
rs1458038	2.00E-13	PRDM8,FGF5	Gout	GCST90455677
rs10857147	2.00E-13	PRDM8,FGF5	BMI and adiposity (confirmatory factor analysis Factor 7)	GCST90309342
rs11099098	2.00E-13	FGF5,PRDM8	Systolic blood pressure x depressive symptoms interaction (2df test)	GCST90093264
rs11726478	2.00E-13	LINC02469	FGF5 protein levels	GCST90469226
rs10857147	2.00E-13	PRDM8,FGF5	Blood urea nitrogen (BUN, minimum, inv-norm transformed)	GCST90479526
rs11098964	2.00E-13	ANTXR2	white blood cell count (WBC, maximum, inv-norm transformed)	GCST90476451
rs10027407	3.00E-13	ANTXR2	White blood cell count	GCST90002374
rs10050061	3.00E-13	ANTXR2	White blood cell count	GCST90002378
rs35851183	3.00E-13	LINC02469,PCAT4	Body mass index	GCST90018947
rs35757217	3.00E-13	FGF5	FGF5 protein levels	GCST90469226
rs6848130	3.00E-13	FGF5,PRDM8	Systolic blood pressure (standard GWA)	GCST90267304
rs12642606	3.00E-13	NAA11,LINC01088	Schizophrenia	GCST90503210
rs10857147	5.00E-13	PRDM8,FGF5	Pulse pressure	GCST007705
rs16998073	5.00E-13	PRDM8,FGF5	Systolic blood pressure x alcohol consumption (light vs heavy) interaction (2df test)	GCST006170
rs62299775	5.00E-13	PCAT4,LINC02469	CXCL10 levels	GCST90274886
rs17004869	6.00E-13	FGF5	Diastolic blood pressure	GCST007704
rs1458038	6.00E-13	PRDM8,FGF5	Apolipoprotein B levels	GCST010243
rs10857147	6.00E-13	PRDM8,FGF5	Coronary artery disease or factor XI levels (pleiotropy)	GCST90129545
rs141610938	6.00E-13	FGF5,PRDM8	Male-pattern baldness	GCST006661
rs11098964	6.00E-13	ANTXR2	Lymphocyte-to-monocyte ratio	GCST90056181
rs16998073	6.00E-13	PRDM8,FGF5	red blood cell count (RBC, mean, inv-norm transformed)	GCST90476349
rs11099098	7.00E-13	FGF5,PRDM8	heart rate (HR, minimum, inv-normal transformed)	GCST90480667
rs17004869	8.00E-13	FGF5	Systolic blood pressure	GCST007703
rs1078234	8.00E-13	LINC01088	FGF5 protein levels	GCST90469226
rs12509595	9.00E-13	PRDM8,FGF5	Systolic blood pressure	GCST007095
rs10857147	9.00E-13	PRDM8,FGF5	Hypopotassemia (PheCode 276.14)	GCST90479944
rs16998073	1.00E-12	PRDM8,FGF5	Systolic blood pressure	GCST007095
rs11736348	1.00E-12	LINC00989	Spherical equivalent	GCST010378
rs79059900	1.00E-12	ANTXR2,RPSAP39	Systolic blood pressure (MTAG)	GCST90449056
rs750021449	1.00E-12	ANTXR2	FGF5 protein levels	GCST90469226
rs10857147	1.00E-12	PRDM8,FGF5	chloride (minimum, inv-norm transformed)	GCST90479541
rs1458038	1.00E-12	PRDM8,FGF5	Biological Father: High blood pressure	GCST90479573
rs1458038	1.00E-12	PRDM8,FGF5	hematocrit (mean, inv-norm transformed)	GCST90475341
rs16998073	2.00E-12	PRDM8,FGF5	Pulse pressure	GCST007705
rs7747	2.00E-12	ANTXR2	Spherical equivalent or myopia (age of diagnosis)	GCST006291
rs12509595	2.00E-12	PRDM8,FGF5	Pulse pressure	GCST90018750
rs16998073	2.00E-12	PRDM8,FGF5	Myocardial infarction	GCST90018877
rs79059900	2.00E-12	ANTXR2,RPSAP39	Diastolic blood pressure (MTAG)	GCST90449057
rs1994190	2.00E-12	LINC01088,NAA11	Drinks per week	GCST90243984
rs7662551	3.00E-12	LINC00989	Myopia (age of diagnosis)	GCST006290
rs36034102	3.00E-12	FGF5	Hypertension	GCST011141
rs16998073	3.00E-12	PRDM8,FGF5	Creatinine levels	GCST90239628
rs13125101	3.00E-12	PRDM8,FGF5	systolic blood pressure (SBP, mean, inv-normal transformed)	GCST90476402
rs371069425	4.00E-12	ANTXR2	White blood cell count	GCST90018978
rs77676437	5.00E-12	ANTXR2	Myopia (age of diagnosis)	GCST006290
rs16998073	5.00E-12	PRDM8,FGF5	Pulse pressure	GCST90239618
rs10857147	5.00E-12	PRDM8,FGF5	Cardiac dysrhythmias (PheCode 427)	GCST90475963
rs16998073	5.00E-12	PRDM8,FGF5	Chronic renal failure [CKD] (PheCode 585.3)	GCST90480379
rs7674623	6.00E-12	PCAT4	Trunk fat mass (UKB data field 23128)	GCST90468180
rs11437847	6.00E-12	FGF5	ICD10 I63: Cerebral infarction	GCST90246098
rs143753691	6.00E-12	LINC02469,LINC00989	Bone mineral density mean	GCST90321120
rs12499307	7.00E-12	ANTXR2	White blood cell count	GCST007070
rs17004869	7.00E-12	FGF5	Hypertension	GCST007707
rs7655441	7.00E-12	ANTXR2	Neutrophil percentage of white cells	GCST004633
rs10857147	7.00E-12	PRDM8,FGF5	Serum urate levels	GCST90319905
rs13125101	7.00E-12	PRDM8,FGF5	diastolic blood pressure (DBP, mean, inv-normal transformed)	GCST90475254
rs11098964	7.00E-12	ANTXR2	white blood cell count (WBC, mean, inv-norm transformed)	GCST90480724
rs10857147	8.00E-12	PRDM8,FGF5	Coronary artery disease or factor VII levels (pleiotropy)	GCST90129542
rs28464573	8.00E-12	ANTXR2	neutrophil (fraction, mean, inv-norm transformed)	GCST90479714

rs13125101	8.00E-12	PRDM8,FGF5	Atrial fibrillation (PheCode 427.21)	GCST90480168
rs13149993	9.00E-12	PRDM8,FGF5	Mean arterial pressure	GCST003275
rs12509595	9.00E-12	PRDM8,FGF5	Medication use (agents acting on the renin-angiotensin system)	GCST90018768
rs11099098	9.00E-12	FGF5,PRDM8	hemoglobin (maximum, inv-norm transformed)	GCST90479638
rs1458038	1.00E-11	PRDM8,FGF5	Direct low density lipoprotein levels (UKB data field 30780)	GCST90468080
rs12509595	1.00E-11	PRDM8,FGF5	Medication use (calcium channel blockers)	GCST90018767
rs72649373	1.00E-11	LINC02469	Body fat percentage (UKB data field 23099)	GCST90468160
rs10857147	1.00E-11	PRDM8,FGF5	Serum uric acid levels	GCST007725
rs1458038	1.00E-11	PRDM8,FGF5	Blood urea nitrogen (BUN, maximum, inv-norm transformed)	GCST90479524
rs1139638	1.00E-11	ANTXR2	Myopia (PheCode 367.1)	GCST90480074
rs13125101	1.00E-11	PRDM8,FGF5	Atrial fibrillation and flutter (PheCode 427.2)	GCST90480170
rs11099098	2.00E-11	FGF5,PRDM8	Pulse pressure	GCST007705
rs11099098	2.00E-11	FGF5,PRDM8	Blood pressure	GCST002497
rs10857147	2.00E-11	PRDM8,FGF5	Coronary artery disease or fibrinogen levels (pleiotropy)	GCST90129546
rs10857147	2.00E-11	PRDM8,FGF5	Coronary artery disease or von Willebrand factor levels (pleiotropy)	GCST90129544
rs10857147	2.00E-11	PRDM8,FGF5	Coronary artery disease or factor VIII levels (pleiotropy)	GCST90129543
rs36077257	2.00E-11	FGF5,PRDM8	Diastolic blood pressure (MTAG)	GCST90449057
rs113087495	2.00E-11	PRDM8,FGF5	Systolic blood pressure (MTAG)	GCST90449056
rs1484145	2.00E-11	NAA11,LINC01088	Major depressive disorder	GCST90020227
rs16998073	2.00E-11	PRDM8,FGF5	Gestational hypertension	GCST90271317
rs16998073	2.00E-11	PRDM8,FGF5	Serum urate levels	GCST90319906
rs10857147	2.00E-11	PRDM8,FGF5	Creatinine levels	GCST90278624
rs12510805	2.00E-11	ANTXR2	neutrophil (fraction, mean, inv-norm transformed)	GCST90475538
rs7016645,rs1498(	2.00E-11	STMN2 x ANKRD50 - FAT4	Neurofibrillary tangles (SNP x SNP interaction)	GCST010343
rs10857147	3.00E-11	PRDM8,FGF5	Total cholesterol levels	GCST90018974
rs6534293	3.00E-11	LINC01088,NAA11	Educational attainment	GCST90105038
rs1994190	4.00E-11	LINC01088,NAA11	Drinks per week	GCST90243989
rs11098965	4.00E-11	ANTXR2	Body fat percentage (adjusted for testosterone and SHBG)	GCST90432180
rs115058055	4.00E-11	LINC01088	Meniere's disease (PheCode 386.1)	GCST90480110
rs6534834	5.00E-11	PRDM8,FGF5	Systolic blood pressure	GCST90132903
rs4333130	5.00E-11	ANTXR2	Body mass index	GCST90255621
rs1458038	6.00E-11	PRDM8,FGF5	Pulse pressure	GCST007705
rs12642606	6.00E-11	NAA11,LINC01088	Subjective well-being (MTAG)	GCST005325
rs12509595	6.00E-11	PRDM8,FGF5	Mean arterial pressure x alcohol consumption interaction (2df test)	GCST006167
rs35851183	6.00E-11	LINC02469,PCAT4	Body mass index	GCST90301650
rs77495688	6.00E-11	LINC00989	CXCL10 levels	GCST90274886
rs11724750	7.00E-11	PCAT4,ANTXR2	Body mass index	GCST009871
rs10857147	1.00E-10	PRDM8,FGF5	Red blood cell count	GCST004601
rs11437847	1.00E-10	FGF5	Hemoglobin levels	GCST010083
rs35851183	1.00E-10	LINC02469,PCAT4	Body mass index	GCST007039
rs1458038	1.00E-10	PRDM8,FGF5	Atrial fibrillation/atrial flutter	GCST90018796
rs113087495	1.00E-10	PRDM8,FGF5	Diastolic blood pressure (MTAG)	GCST90449057
rs56164535	1.00E-10	FGF5,PRDM8	QT interval	GCST90179153
rs16998073	1.00E-10	PRDM8,FGF5	ICD10 O14: Pre-eclampsia	GCST90454233
rs2310325,rs1361(	1.00E-10	LINC02497 x MINAR1 - TFDP1P3	Total PHF-tau (SNP x SNP interaction)	GCST010340
rs10857147	2.00E-10	PRDM8,FGF5	Myocardial infarction	GCST011365
rs16998073	2.00E-10	PRDM8,FGF5	Hypertension	GCST90086157
rs10857147	2.00E-10	PRDM8,FGF5	Urate levels	GCST008973
rs10027407	2.00E-10	ANTXR2	Resistance to COVID-19 infection (Exposed negative vs positive)	GCST90255358
rs10857147	3.00E-10	PRDM8,FGF5	Serum uric acid levels	GCST005983
rs12509595	3.00E-10	PRDM8,FGF5	Blood urea nitrogen levels	GCST008062
rs12509595	3.00E-10	PRDM8,FGF5	Ischemic stroke	GCST90018864
rs184765639	3.00E-10	LINC02469,LINC00989	Perceptual rivalry (voluntary modulation strength)	GCST007178
rs56164535	3.00E-10	FGF5,PRDM8	QT interval	GCST90179154
rs7685593	4.00E-10	ANTXR2	Age-related eyesight deterioration (confirmatory factor analysis Factor 27)	GCST90309361
rs138066321	4.00E-10	ANTXR2	Rheumatoid arthritis	GCST90132222
rs10010217	4.00E-10	ANTXR2,PCAT4	Age at onset of walking	GCST90651078
rs2903657	4.00E-10	PRDM8,FGF5	ICD10 I10: Essential (primary) hypertension	GCST90246081
rs3796606	4.00E-10	FGF5	Body mass index	GCST90255621
rs6847975	5.00E-10	ANTXR2,PCAT4	Body fat percentage	GCST007064
rs16998073	5.00E-10	PRDM8,FGF5	Diastolic blood pressure	GCST008029
rs3796606	6.00E-10	FGF5	Monobrow	GCST003996
rs13104584	6.00E-10	PCAT4,ANTXR2	Adult body size	GCST010988
rs10857147	6.00E-10	PRDM8,FGF5	Blood urea nitrogen levels	GCST90018728
rs17465728	6.00E-10	PRDM8,FGF5	Diastolic blood pressure (MTAG)	GCST90449057
rs1542636	6.00E-10	NAA11,LINC01088	hyperactivity disorder or Anorexia nervosa or Major depressive disorder or Obsesive-c	GCST90624575
rs16998073	6.00E-10	PRDM8,FGF5	Uric acid levels (1KG-JEWEL7K)	GCST90444429
rs7686601	6.00E-10	FGF5,PRDM8	Systolic blood pressure	GCST90310304
rs7654647	7.00E-10	PCAT4,ANTXR2	Predicted visceral adipose tissue	GCST008744
rs10857147	7.00E-10	PRDM8,FGF5	Pulse pressure	GCST006009
rs16998073	7.00E-10	PRDM8,FGF5	Estimated glomerular filtration rate in non-diabetics	GCST008745
rs10857147	7.00E-10	PRDM8,FGF5	Medication use (HMG CoA reductase inhibitors)	GCST90018989
rs76275007	7.00E-10	ANTXR2	Resistance to COVID-19 infection (Exposed negative vs positive)	GCST90255358
rs6842571	7.00E-10	ANTXR2,RPSAP39	Height	GCST90245848
rs13125101	8.00E-10	PRDM8,FGF5	Diastolic blood pressure	GCST008029
rs1458038	9.00E-10	PRDM8,FGF5	Systolic blood pressure	GCST007237
rs28454198	9.00E-10	NAA11,LINC01088	Subjective well-being	GCST005328

rs6534834	9.00E-10	PRDM8,FGF5	FGF5 protein levels	GCST90277792
rs11099098	9.00E-10	FGF5,PRDM8	Diastolic blood pressure	GCST008029
rs1678311,rs12365	9.00E-10	LINC02517 - ACOX3 x TENM4	Diffuse plaques (SNP x SNP interaction)	GCST010341
rs17004869	1.00E-09	FGF5	Systolic blood pressure	GCST004279
rs35851183	1.00E-09	LINC02469,PCAT4	Body mass index	GCST009004
rs1458038	1.00E-09	PRDM8,FGF5	Diastolic blood pressure	GCST003273
rs16998073	1.00E-09	PRDM8,FGF5	Red blood cell count	GCST90018751
rs36077257	1.00E-09	FGF5,PRDM8	Systolic blood pressure (MTAG)	GCST90449056
rs1458038	1.00E-09	PRDM8,FGF5	Diastolic blood pressure	GCST008029
rs1458038	1.00E-09	PRDM8,FGF5	Atrial fibrillation (MTAG)	GCST90132229
rs28454198	1.00E-09	NAA11,LINC01088	Schizophrenia	GCST90128471
rs72649373	2.00E-09	LINC02469	Body mass index	GCST009871
rs12509595	2.00E-09	PRDM8,FGF5	Hematocrit	GCST004604
rs1458038	2.00E-09	PRDM8,FGF5	Atrial fibrillation	GCST006414
rs572381265	2.00E-09	ANTXR2	White blood cell count	GCST90002407
rs1038425	2.00E-09	NAA11,LINC01088	Major depressive disorder (broad)	GCST90328131
rs62298617	2.00E-09	ANTXR2	Gout	GCST90455676
rs10010217	2.00E-09	ANTXR2,PCAT4	Body mass index	GCST90446645
rs13125101	2.00E-09	PRDM8,FGF5	Medication use (HMG CoA reductase inhibitors)	GCST007931
rs11724750	2.00E-09	PCAT4,ANTXR2	Body mass index (MTAG)	GCST90179150
rs1458038	2.00E-09	PRDM8,FGF5	LDL (standard GWA)	GCST90267272
rs1132460	2.00E-09	PCAT4	Insomnia	GCST90131901
rs72860756	2.00E-09	LINC00989,GK2	t microbial network clusters (Pink (at 1 year) x Any Breastfeeding (3 Months) interacti	GCST90569458
rs10857147	2.00E-09	PRDM8,FGF5	Myocardial infarction	GCST90432175
rs11099098	3.00E-09	FGF5,PRDM8	Urate levels	GCST010637
rs10857147	3.00E-09	PRDM8,FGF5	Blood urea nitrogen levels	GCST90018948
rs115050282	3.00E-09	GK2,NAA11	Protein quantitative trait loci (liver)	GCST011427
rs58383713	3.00E-09	PCAT4	Hand grip strength	GCST005830
rs9998799	3.00E-09	LINC01088,NAA11	Schizophrenia	GCST011768
rs1458038	3.00E-09	PRDM8,FGF5	Atrial fibrillation (MTAG)	GCST90449054
rs16998073	3.00E-09	PRDM8,FGF5	Hypertension	GCST90086092
rs12504282	3.00E-09	ANTXR2	Reactive lymphocyte count	GCST90281252
rs36034102	4.00E-09	FGF5	PR interval	GCST010321
rs1484144	4.00E-09	NAA11,LINC01088	autism spectrum disorder, bipolar disorder, major depression, obsessive-compulsive d	GCST009600
rs11099097	4.00E-09	PRDM8,FGF5	Coronary artery disease	GCST010479
rs13125101	4.00E-09	PRDM8,FGF5	Systolic blood pressure	GCST008044
rs35851183	4.00E-09	LINC02469,PCAT4	BMI (standard GWA)	GCST90267268
rs727312	4.00E-09	NAA11,LINC01088	Resistance to COVID-19 infection (Exposed negative vs positive)	GCST90255358
rs11931537	4.00E-09	PCAT4,LINC02469	Insomnia	GCST90131901
rs10002178	4.00E-09	LINC02469	Height	GCST90245848
rs36034102	5.00E-09	FGF5	PR interval	GCST010320
rs113323321	5.00E-09	ANTXR2	Longitudinal brain connectivity change (global efficiency)	GCST009728
rs16998073	5.00E-09	PRDM8,FGF5	Stable angina pectoris	GCST90018915
rs10857147	6.00E-09	PRDM8,FGF5	ion, percutaneous transluminal coronary angioplasty, coronary artery bypass grafting	GCST004787
rs36034102	6.00E-09	FGF5	Descending aorta maximum area	GCST90137442
rs11099098	6.00E-09	FGF5,PRDM8	Systolic blood pressure	GCST008044
rs17003752	6.00E-09	NAA11,GK2	Smoking status (standard GWA)	GCST90267302
rs199788196	6.00E-09	NAA11,LINC01088	Resistance to COVID-19 infection (Exposed negative vs positive)	GCST90255358
rs35954793	6.00E-09	FGF5	Hypertension complicating pregnancy	GCST90245819
rs12509595	7.00E-09	PRDM8,FGF5	Red blood cell count	GCST90278641
rs12509595	8.00E-09	PRDM8,FGF5	Medication use (antithrombotic agents)	GCST90018982
rs1902859	9.00E-09	PRDM8,FGF5	Pulse pressure	GCST007705
rs12509595	9.00E-09	PRDM8,FGF5	Estimated glomerular filtration rate	GCST008060
rs10857147	9.00E-09	PRDM8,FGF5	Estimated glomerular filtration rate (cystatin c)	GCST90103635
rs12509595	9.00E-09	PRDM8,FGF5	Heart failure	GCST90455657
rs16998073	9.00E-09	PRDM8,FGF5	Preeclampsia or small for gestational age infant (maternal genotype effect)	GCST90269905
rs60661769	9.00E-09	ANTXR2,PCAT4	Whole body fat mass (UKB data field 23100)	GCST90428121
rs13149993	1.00E-08	PRDM8,FGF5	Pulse pressure	GCST007705
rs1458038	1.00E-08	PRDM8,FGF5	Preeclampsia (maternal genotype effect)	GCST90013478
rs9307551	1.00E-08	LINC00989	Refractive error	GCST001858
rs35851183	1.00E-08	LINC02469,PCAT4	Weight	GCST90018949
rs115050282	1.00E-08	GK2,NAA11	Protein quantitative trait loci (liver)	GCST011427
rs12509595	1.00E-08	PRDM8,FGF5	Ischemic stroke	GCST90018644
rs36034102	1.00E-08	FGF5	Angina pectoris	GCST90018793
rs6848123	1.00E-08	NAA11,LINC01088	Schizophrenia	GCST90053836
rs17003752	1.00E-08	NAA11,GK2	Lifetime smoking	GCST90100569
rs1458038	1.00E-08	PRDM8,FGF5	Systolic blood pressure	GCST008044
rs16998073	1.00E-08	PRDM8,FGF5	Systolic blood pressure	GCST008044
rs10857147	1.00E-08	PRDM8,FGF5	Urate levels	GCST008972
rs36034102	1.00E-08	FGF5	Diastolic blood pressure x depressive symptoms interaction (2df test)	GCST90093267
rs2867610	1.00E-08	LINC01088,NAA11	Depression	GCST90239706
rs16998073	1.00E-08	PRDM8,FGF5	Hemoglobin concentration	GCST90258653
rs7674623	1.00E-08	PCAT4	Whole body fat mass (UKB data field 23100)	GCST90428121
rs12208017,rs4560	1.00E-08	PHIP - HMGN3 x RNA5SP173 - NDUFB5P1	Neurofibrillary tangles (SNP x SNP interaction)	GCST010343
rs17003752	2.00E-08	NAA11,GK2	Smoking status	GCST007085
rs12642606	2.00E-08	NAA11,LINC01088	Depressive symptoms (MTAG)	GCST005323
rs35487709	2.00E-08	LINC00989	Spherical equivalent	GCST006289

rs12509595	2.00E-08	PRDM8,FGF5	Blood urea nitrogen levels	GCST005986
rs12509595	2.00E-08	PRDM8,FGF5	Red blood cell count	GCST005996
rs11098964	2.00E-08	ANTXR2	es (ankylosing spondylitis, Crohn's disease, psoriasis, primary sclerosing cholangitis, u	GCST005537
rs1484144	2.00E-08	NAA11,LINC01088	Attention deficit hyperactivity disorder (MTAG)	GCST012093
rs36034102	2.00E-08	FGF5	Descending aorta maximum area (MTAG)	GCST90137451
rs7685593	2.00E-08	ANTXR2	Myopia	GCST90134549
rs10857147	2.00E-08	PRDM8,FGF5	Urate levels	GCST008971
rs11099098	2.00E-08	FGF5,PRDM8	Cardioembolic stroke (MTAG)	GCST90132228
rs58981705	2.00E-08	FGF5,PRDM8	ut microbial network clusters (Tan (at 3 months) x Summer Birth (Jun-Aug) interactio	GCST90569246
rs10019300	2.00E-08	ANTXR2	CAMSIS occupational score (MTAG)	GCST90492678
rs76486815	2.00E-08	LINC00989	ndance (class Clostridium sensu stricto sp. 17 (at 1 year) x Exclusive Breastfeeding (6	GCST90569029
rs1484140	2.00E-08	NAA11	Subjective well-being	GCST90128422
rs141026327	2.00E-08	LINC02469,PCAT4	Color vision defects (Tritan)	GCST90301671
rs66968950	3.00E-08	ANTXR2	Eosinophil counts	GCST007065
rs1458038	3.00E-08	PRDM8,FGF5	Diastolic blood pressure	GCST003273
rs76202297	3.00E-08	LINC01088	Height	GCST90105052
rs76101924	3.00E-08	LINC01088	Protein quantitative trait loci (liver)	GCST011427
rs10857147	3.00E-08	PRDM8,FGF5	Coronary artery disease	GCST007990
rs10857147	3.00E-08	PRDM8,FGF5	Medication use (antithrombotic agents)	GCST90018762
rs9992915	3.00E-08	ANTXR2	Educational attainment (MTAG)	GCST006571
rs117010230	3.00E-08	LINC00989	Alzheimer's disease	GCST90624093
rs12509595	3.00E-08	PRDM8,FGF5	Left ventricular end systole anterospetal wall thickness	GCST90278513
rs9783424,rs19031	3.00E-08	PPP1R12A x LINC01179	Total PHF-tau (SNP x SNP interaction)	GCST010340
rs28459916	4.00E-08	GK2,LINC00989	Smoking status (ever vs never smokers)	GCST007327
rs56326811	4.00E-08	GK2,NAA11	DNA methylation variation (age effect)	GCST006660
rs6816922	4.00E-08	LINC01088,NAA11	Chronotype	GCST007576
rs16998073	4.00E-08	PRDM8,FGF5	Ascending thoracic aortic diameter	GCST90094400
rs72649373	4.00E-08	LINC02469	Whole body fat mass (UKB data field 23100)	GCST90428121
rs16998073	4.00E-08	FGF5,PRDM8	Ascending thoracic aortic diameter	GCST90094400

Supplemental Table 8. Genome-wide significant associations reported in the GWAS catalog within hg19 chr9:37,687,113-38,822,977 (hg38 chr9:37,687,115-38,822,980).

Variant	P Value	Mapped Genes	Reported Trait	Study Accession
rs4878795	3.00E-125	ALDH1B1,U8	IGFBPL1 protein levels	GCST90469533
rs139527453	5.00E-76	VN1R48P,GAS2L1P1	IGFBPL1 protein levels	GCST90469533
rs10758481	1.00E-60	U8,ALDH1B1	platelet count (mean, inv-norm transformed)	GCST90476299
rs2228093	2.00E-54	ALDH1B1	Daily alcohol intake in rs671 GA genotype	GCST90319893
rs10758481	4.00E-54	U8,ALDH1B1	Platelet count	GCST90002361
rs10758481	1.00E-50	U8,ALDH1B1	Platelet count	GCST90002357
rs7846941	1.00E-50	ALDH1B1	IGFBPL1 protein levels	GCST90469533
rs2228093	4.00E-50	ALDH1B1	Daily alcohol intake x rs671 interaction (2df)	GCST90319895
rs2228093	1.00E-49	ALDH1B1	Alcohol drinking status x rs671 interaction (2df)	GCST90319896
rs3043	2.00E-47	ALDH1B1	Daily alcohol intake in rs671 GA genotype	GCST90319893
rs10758481	2.00E-47	U8,ALDH1B1	platelet count (minimum, inv-norm transformed)	GCST90476302
rs10973797	1.00E-46	IGFBPL1,ARMC8P1	Insulin-like growth factor-binding protein-like 1 levels	GCST90179323
rs3853284	9.00E-46	ANKRD18A	IGFBPL1 protein levels	GCST90469533
rs12238372	6.00E-41	IGFBPL1	Insulin-like growth factor-binding protein-like 1 levels	GCST90427086
rs10758481	4.00E-39	U8,ALDH1B1	platelet count (maximum, inv-norm transformed)	GCST90476296
rs12343705	2.00E-38	ALDH1B1,U8	Platelet count (UKB data field 30080)	GCST90468095
rs10758481	3.00E-35	U8,ALDH1B1	Platelet crit (UKB data field 30090)	GCST90468096
rs10973700	1.00E-34	ALDH1B1,U8	Plateletcrit	GCST90002400
rs10758481	3.00E-34	U8,ALDH1B1	Platelet count	GCST90002402
rs927631	2.00E-33	EXOSC3	Height	GCST90245848
rs10973772	6.00E-33	U8,ALDH1B1	Insulin-like growth factor-binding protein-like 1 levels	GCST90179323
rs10758481	3.00E-32	U8,ALDH1B1	Platelet count	GCST90018969
rs1974262	2.00E-31	ANKRD18A	IGFBPL1 protein levels	GCST90469533
rs111359624	2.00E-30	TCEA1P3,GAS2L1P1	IGFBPL1 protein levels	GCST90469533
rs80045011	3.00E-30	ARMC8P1	IGFBPL1 protein levels	GCST90469533
rs117810662	7.00E-30	IGFBPL1,ARMC8P1	IGFBPL1 protein levels	GCST90469533
rs78392476	9.00E-30	ALDH1B1,U8	IGFBPL1 protein levels	GCST90469533
rs117895195	8.00E-28	ARMC8P1,TCEA1P3	IGFBPL1 protein levels	GCST90469533
rs2585666	1.00E-27	U8,ALDH1B1	platelet count (mean, inv-norm transformed)	GCST90480651
rs2585666	2.00E-26	U8,ALDH1B1	platelet count (minimum, inv-norm transformed)	GCST90480652
rs7851774	3.00E-25	SHB	CEACAM19 protein levels	GCST90468691
rs11446839I	9.00E-24	ALDH1B1,U8	Lymphocyte count	GCST90002316
rs542662582	7.00E-23	IGFBPL1	IGFBPL1 protein levels	GCST90469533
rs10973892	1.00E-22	ANKRD18A	IGFBPL1 protein levels	GCST90469533
rs12003607,rs2165776,rs	3.00E-22	U8 - ALDH1B1; U8 - ALDH1B1; U8 - ALDH1B1; U8 - ALDH1B1; U8 - ALDH1B1; U8 - ALDH1B1	Core binding factor acute myeloid leukemia	GCST008413
rs7861055	5.00E-22	ALDH1B1,U8	Lymphocyte count	GCST90002320
rs10758481	7.00E-21	U8,ALDH1B1	platelet count (maximum, inv-norm transformed)	GCST90480650
rs11388692	2.00E-20	IGFBPL1,ARMC8P1	Insulin-like growth factor-binding protein-like 1 levels	GCST90179323
rs12343705	8.00E-20	ALDH1B1,U8	Lymphocyte count	GCST90002388
rs184935473	2.00E-19	U8,ALDH1B1	IGFBPL1 protein levels	GCST90469533
rs4878776	2.00E-19	U8,ALDH1B1	IGFBPL1 protein levels	GCST90469533
rs3043	3.00E-19	ALDH1B1	Alcohol consumption (drinkers vs non-drinkers)	GCST009799
rs12003607,rs2165776,rs	4.00E-19	U8 - ALDH1B1; U8 - ALDH1B1; U8 - ALDH1B1; U8 - ALDH1B1; U8 - ALDH1B1; U8 - ALDH1B1	Core binding factor acute myeloid leukemia	GCST008413
rs575731091	4.00E-19	U8,ALDH1B1	IGFBPL1 protein levels	GCST90469533
rs533460447	9.00E-18	ALDH1B1,U8	IGFBPL1 protein levels	GCST90469533
rs187522824	1.00E-17	SHB	IGFBPL1 protein levels	GCST90469533
rs4878737	3.00E-17	SHB	Male-pattern baldness	GCST007020
rs10814706,rs2996368	6.00E-17	FAM220BP - ANKRD18A; VN1R48P - FAM220BP	Core binding factor acute myeloid leukemia	GCST008413
rs12554114	2.00E-16	ALDH1B1	GLIPR1 protein levels	GCST90469357
rs11446839	3.00E-16	ALDH1B1,U8	Plateletcrit	GCST004607
rs193245362	2.00E-15	U8,ALDH1B1	GLIPR1 protein levels	GCST90469357
rs11446839	3.00E-15	ALDH1B1,U8	Platelet count	GCST004603
rs554003018	3.00E-15	FAM201A,YWHABP1	IGFBPL1 protein levels	GCST90469533
rs7861857,rs1327848	3.00E-15	FAM240B - VN2R3P x CELF2-DT - ORMDL1P1	Total PHF-tau (SNP x SNP interaction)	GCST010340
rs62537833	2.00E-14	RNU6-765P,YWHABP1	IGFBPL1 protein levels	GCST90469533
rs76084197	4.00E-14	IGFBPL1	IGFBPL1 protein levels	GCST90469533
rs10758481	5.00E-14	U8,ALDH1B1	Monocyte count	GCST90002340
rs10814706,rs2996368	7.00E-14	FAM220BP - ANKRD18A; VN1R48P - FAM220BP	Core binding factor acute myeloid leukemia	GCST008413
rs2585668	7.00E-14	ALDH1B1,U8	Lymphocyte count	GCST90018962
rs566759320	7.00E-14	ANKRD18A	IGFBPL1 protein levels	GCST90469533
rs7861055	1.00E-13	ALDH1B1,U8	Lymphocyte count (UKB data field 30120)	GCST90468082
rs776022	1.00E-13	SHB	IGFBPL1 protein levels	GCST90469533
rs10758475,rs2581745	6.00E-13	SHB - U8 x GPM6A	Total PHF-tau (SNP x SNP interaction)	GCST010340
rs12554087	8.00E-13	FRMPD1	GRHPR protein levels	GCST90469400
rs10973700	1.00E-12	ALDH1B1,U8	White blood cell count	GCST90002374
rs12555457	2.00E-12	ALDH1B1	Aldehyde dehydrogenase, mitochondrial levels	GCST90246482
rs10758481	6.00E-12	U8,ALDH1B1	Monocyte count	GCST90002344
rs12235833	6.00E-12	U8,ALDH1B1	IGFBPL1 protein levels	GCST90469533
rs570859455	8.00E-12	VN1R48P,FAM220BP	Biological Grandparent (paternal): Liver Condition	GCST90479735
rs10125515	1.00E-11	ALDH1B1,U8	White blood cell count	GCST90002378
rs544312545	1.00E-11	SHB	Dermatitis due to solar radiation (PheCode 938)	GCST90480626
rs72733221	2.00E-11	ALDH1B1,U8	Smoking initiation	GCST90243968
rs551339002	3.00E-11	ALDH1B1,U8	Swelling of limb (PheCode 771.1)	GCST90480574
rs56268063	4.00E-11	SHB	Lung function (FEV1/FVC)	GCST007080
rs10758481	4.00E-11	U8,ALDH1B1	Monocyte count	GCST90002393
rs3849927	6.00E-11	SHB	Hair color	GCST007082
rs72733235	6.00E-11	ALDH1B1,U8	Smoking initiation	GCST90243985
rs7861055	7.00E-11	ALDH1B1,U8	Platelet count during second trimester of pregnancy	GCST90302225
rs1928243	8.00E-11	SHB	Balding type 1	GCST007038
rs7861857,rs1264474	1.00E-10	FAM240B - VN2R3P x RN7SL563P - GRHL2-DT	Total PHF-tau (SNP x SNP interaction)	GCST010340
rs7861857,rs4523894	3.00E-10	FAM240B - VN2R3P x ADAMTSL3	Total PHF-tau (SNP x SNP interaction)	GCST010340
rs77181999	4.00E-10	SHB	Protein quantitative trait loci (liver)	GCST011427
rs12343705	5.00E-10	ALDH1B1,U8	White blood cell count	GCST90002407
rs2810748	1.00E-09	ALDH1B1,U8	Platelet count during third trimester of pregnancy	GCST90302226
rs7861055	1.00E-09	ALDH1B1,U8	Platelet count during first trimester of pregnancy	GCST90302224
rs10973953	1.00E-09	FAM201A,YWHABP1	Oral microbiota abundance (Kingella) in oral squamous cell carcinoma	GCST90101805
rs10758481	1.00E-09	U8,ALDH1B1	Platelet count	GCST90278640
rs11330143	2.00E-09	EXOSC3,DCAF10	Reticulocyte count	GCST90002405
rs12550953	2.00E-09	SHB,U8	Pulse pressure	GCST90132905
rs10973748	2.00E-09	ALDH1B1,U8	Longevity (100 years and older)	GCST012419
rs2810747	2.00E-09	ALDH1B1,U8	Platelet count	GCST90239624
rs341473,rs7191157	2.00E-09	U8 - ALDH1B1 x CDYL2	Neuritic plaques (SNP x SNP interaction)	GCST010342
rs73449401	3.00E-09	DCAF10	High light scatter reticulocyte count	GCST90002385
rs10758481	3.00E-09	U8,ALDH1B1	Platelet count	GCST90018749
rs1928244	4.00E-09	SHB	Systolic blood pressure	GCST007087
rs62536879	4.00E-09	U8,SHB	Systolic blood pressure	GCST90018972
rs78591290	4.00E-09	TRMT10B,FRMPD1	Lung function (forced vital capacity)	GCST90244093
rs72733235	5.00E-09	ALDH1B1,U8	Smoking status (ever vs never smokers)	GCST007327
rs10758481	5.00E-09	U8,ALDH1B1	White blood cell count	GCST90018978
rs2585668	6.00E-09	ALDH1B1,U8	Neutrophil percentage of white cells	GCST90002399
rs7047650	6.00E-09	U8,SHB	Pulse pressure	GCST90310296
rs78797545	6.00E-09	U8,SHB	COVID-19 vaccination	GCST90255613
rs4878743,rs3093135	6.00E-09	SHB x CYP4F2	Total PHF-tau (SNP x SNP interaction)	GCST010340
rs10169138,rs2417670	6.00E-09	CDC42EP3-AS1, PIRAT1 x SLC25A6P5 - LINC01505	Neurofibrillary tangles (SNP x SNP interaction)	GCST010343
rs7019055	7.00E-09	U8,SHB	Pulse pressure	GCST007096
rs10732358	7.00E-09	SHB	Atrial fibrillation	GCST90624411
rs7847267	8.00E-09	FRMPD1	Resistance to COVID-19 infection (Exposed negative vs positive)	GCST90255358
rs72733235	1.00E-08	ALDH1B1,U8	Smoking status	GCST007085
rs56209921	1.00E-08	ALDH1B1,U8	Smoking status (ever vs never smokers)	GCST010018
rs4878737	1.00E-08	SHB	acne vulgaris	GCST90092000
rs78688365	1.00E-08	ARMC8P1	Revision surgery due to aseptic loosening after total joint arthroplasty	GCST90102256
rs1203948313	1.00E-08	RNU6-765P,FAM240B	Alzheimer's disease or family history of Alzheimer's disease	GCST90624094
rs116954858	1.00E-08	ARMC8P1,IGFBPL1	Bone mineral density variability	GCST90321121
rs7861857,rs2682754	1.00E-08	FAM240B - VN2R3P x SUDS3 - LINC02460	Total PHF-tau (SNP x SNP interaction)	GCST010340
rs7861857,rs2682754	1.00E-08	FAM240B - VN2R3P x SUDS3 - LINC02460	Neurofibrillary tangles (SNP x SNP interaction)	GCST010343
rs7030685	2.00E-08	ALDH1B1,U8	Azorhizobium abundance in stool	GCST90032197
rs72719497	2.00E-08	RNU6-765P,FAM240B	Gut microbial network clusters (Salmon (at 1 year) x Vaginal Birth interaction	GCST90569412
rs188613153	2.00E-08	FAM220BP,VN1R48P	Hearing difficulty	GCST90044775
rs62541320	2.00E-08	SHB	RBC levels of LPI18:1	GCST90267504
rs10973987,rs10007750	2.00E-08	FAM240B x PPARGC1A	Total PHF-tau (SNP x SNP interaction)	GCST010340
rs17684675,rs10973494	2.00E-08	CRBN - SUMF1 x FRMPD1	Total PHF-tau (SNP x SNP interaction)	GCST010340
rs10758475,rs6506073	2.00E-08	SHB - U8 x MYOM1	Total PHF-tau (SNP x SNP interaction)	GCST010340
rs10973700	3.00E-08	ALDH1B1,U8	White blood cell count	GCST007070
rs4570248	3.00E-08	U8,SHB	Vertex-wise sulcal depth	GCST90095129
rs3808862	3.00E-08	U8,SHB	Chronic obstructive pulmonary disease liability (machine learning-based score)	GCST90244098
rs75635242	3.00E-08	YWHABP1,RNU6-765P	Gut microbial network clusters (Salmon (at 1 year) x Household furry pet dog (1 Year) interaction	GCST90569451
rs7875054	3.00E-08	YWHABP1,RNU6-765P	Gut microbial network clusters (Salmon (at 1 year) x Any Breastfeeding (3 Months) interaction	GCST90569450
rs78622592	4.00E-08	SHB,U8	Pulse pressure	GCST90018970
rs10283803	4.00E-08	SHB	Educational attainment	GCST90105038

Supplemental Table 9. Genome-wide significant associations reported in the GWAS catalog within hg19 chr17:27,344,402-29,783,141 (hg38 17:29,017,383-31,456,123)

Variant	P Value	Mapped Genes	Reported Trait	Study Accession
rs3760318	1.00E-300	ADAP2	Height	GCST90245843
rs140040369	1.00E-300	TEFM,ATAD5	Bone mineral density mean	GCST90321120
rs61749868	1.00E-300	RNF135	Bone mineral density mean	GCST90321120
rs6505211	1.00E-300	CRLF3	Height	GCST90245848
rs3760318	1.00E-300	ADAP2	Height	GCST90245848
rs17767418	1.00E-299	GOSR1	Height	GCST90245848
rs9891920	2.00E-298	TAOK1	Mean platelet volume	GCST004599
rs8068104	2.00E-288	RNU4-34P,RPL35AP35	mean platelet volume (MPV, minimum, inv-norm transformed)	GCST90479709
rs56709487	2.00E-268	TAOK1	Platelet count (UKB data field 30080)	GCST90468095
rs6505216	3.00E-253	ATAD5	What is your height? (cm, inv-normal transformed)	GCST90475368
rs116524302	8.00E-247	CRLF3	BLMH protein levels	GCST90468447
rs137881719	3.00E-243	BLMH	BLMH protein levels	GCST90468447
rs9900280	2.00E-239	TAOK1	Platelet count	GCST90002361
rs6505129	2.00E-235	TAOK1	Platelet count	GCST90002357
rs6505216	5.00E-225	ATAD5	Height	GCST90435412
rs6505216	3.00E-223	ATAD5	Standing height (UKB data field 50)	GCST90468178
rs7223535	1.00E-222	ATAD5	Height	GCST007841
rs542939	2.00E-213	ABHD15,ABHD15-AS1	Height	GCST90245848
rs8065958	8.00E-187	TAOK1	Platelet count	GCST90018969
rs6505216	9.00E-187	ATAD5	Height (baseline)	GCST90565843
rs191010498	1.00E-173	TAOK1	Mean platelet thrombocyte volume (UKB data field 30100)	GCST90468087
rs11656408	6.00E-169	BLMH,TMIGD1	GPA33 protein levels	GCST90469385
rs11656408	6.00E-155	BLMH,TMIGD1	EPCAM protein levels	GCST90469126
rs73268099	1.00E-152	BLMH	Bleomycin hydrolase levels	GCST90179234
rs3110496	1.00E-141	GIT1,ABHD15-AS1,ANKRD13B	Height	GCST90245848
rs7209300	2.00E-139	TAOK1	Mean platelet volume	GCST004599
rs6505216	4.00E-122	ATAD5	Height	GCST90018959
rs11654523	1.00E-121	ABHD15-AS1,SSH2	Height	GCST90245848
rs35763383	5.00E-118	TAOK1	Platelet count	GCST90056183
rs7209032	4.00E-108	RN7SL316P,LRRC37BP1	Height	GCST90245848
rs551321261	4.00E-105	NUFIP2	Bone mineral density mean	GCST90321120
rs574333826	4.00E-105	NUFIP2	Bone mineral density mean	GCST90321120
rs6505216	2.00E-101	ATAD5	Appendicular lean mass	GCST90000025
rs76947600	4.00E-100	ATAD5	height (mean, inv-normal transformed)	GCST90479635
rs2320954	8.00E-97	TAOK1	Platelet side fluorescence	GCST90281193
rs11651858	2.00E-95	ATAD5	Height	GCST90245848
rs6505216	3.00E-95	ATAD5	Height (maximum, inv-normal transformed)	GCST90479634
rs76947600	6.00E-95	ATAD5	height (minimum, inv-normal transformed)	GCST90479636
rs12938714	2.00E-92	TMIGD1	SLC51B protein levels	GCST90470664
rs602056	2.00E-90	TAOK1	Platelet large cell ratio	GCST90281201
rs3760318	1.00E-85	ADAP2	Height	GCST90245844
rs2946741	6.00E-85	TAOK1	Platelet forward scatter	GCST90281194
rs9911167	2.00E-83	ANKRD13B,ABHD15-AS1	Vertex-wise sulcal depth	GCST90095129
rs12945042	1.00E-82	SLC6A4,SNORD63	TSPAN8 protein levels	GCST90470980
rs35184780	1.00E-80	ABHD15-AS1,ANKRD13B	Mean platelet thrombocyte volume (UKB data field 30100)	GCST90468087
rs6505216	1.00E-75	ATAD5	What is your height? (cm, inv-normal transformed)	GCST90479637
rs6505216	7.00E-73	ATAD5	Whole body water mass (UKB data field 23102)	GCST90468184
rs8080300	2.00E-72	TMIGD1,BLMH	S100A14 protein levels	GCST90470515
rs8068104	4.00E-72	RNU4-34P,RPL35AP35	platelet count (mean, inv-norm transformed)	GCST90480651
rs9890032	2.00E-71	ATAD5	Anthropometric traits	GCST007485
rs61542570	3.00E-70	RPL35AP35,RNU4-34P	Platelet count	GCST004603
rs2617881	8.00E-70	SSH2	Height	GCST90245848
rs542939	2.00E-69	ABHD15,ABHD15-AS1	Standing height (UKB data field 50)	GCST90468178
rs35763383	1.00E-66	TAOK1	Platelet count	GCST90002402
rs8068104	1.00E-66	RNU4-34P,RPL35AP35	platelet count (minimum, inv-norm transformed)	GCST90480652
rs602056	2.00E-65	TAOK1	Immature platelet fraction	GCST90281200
rs8068104	1.00E-64	RNU4-34P,RPL35AP35	mean platelet volume (MPV, mean, inv-norm transformed)	GCST90475519
rs6505216	2.00E-64	ATAD5	Physical function (baseline)	GCST90565837
rs6505216	3.00E-64	ATAD5	Basal metabolic rate (UKB data field 23105)	GCST90468159
rs7208859	7.00E-63	ADAP2	Mean corpuscular volume	GCST90002338
rs542939	3.00E-60	ABHD15,ABHD15-AS1	Height	GCST90435412
rs2874402	5.00E-60	TAOK1	Platelet-to-lymphocyte ratio	GCST90056184
rs2729450	1.00E-59	SSH2	Monocyte count	GCST90002340
rs191010498	3.00E-59	TAOK1	Mean platelet volume	GCST90002395
rs7210288	7.00E-59	SSH2	Monocyte count	GCST90002344
rs8068104	8.00E-59	RNU4-34P,RPL35AP35	mean platelet volume (MPV, mean, inv-norm transformed)	GCST90475518
rs7208859	1.00E-58	ADAP2	Mean corpuscular volume	GCST90002334
rs79894787	1.00E-58	NUFIP2,RPL35AP35	Mean platelet thrombocyte volume (UKB data field 30100)	GCST90468087
rs35893904	9.00E-58	TAOK1	BLMH protein levels	GCST90468447
rs8068104	2.00E-57	RNU4-34P,RPL35AP35	mean platelet volume (MPV, minimum, inv-norm transformed)	GCST90475522

rs542939	1.00E-56	ABHD15,ABHD15-AS1	Height (baseline)	GCST90565843
rs8068104	3.00E-56	RNU4-34P,RPL35AP35	mean platelet volume (MPV, maximum, inv-norm transformed)	GCST90475515
rs602056	7.00E-56	TAOK1	Platelet side scatter	GCST90281192
rs602056	5.00E-55	TAOK1	Immature platelet count	GCST90281199
rs2244592	6.00E-55	SSH2	Lung function (FEV1/FVC)	GCST007080
rs602056	4.00E-54	TAOK1	High fluorescence immature platelet fraction	GCST90281198
rs555695475	7.00E-54	RNU4-34P,TAOK1	Platelet large cell ratio	GCST90281201
rs880749	1.00E-53	SSH2	Monocyte count	GCST90002393
rs3097118	2.00E-53	ABHD15-AS1,SSH2	Vertex-wise cortical thickness	GCST90095131
rs190114804	4.00E-53	RN7SL316P,LRRC37BP1	BLMH protein levels	GCST90468447
rs112707384	6.00E-52	KRT17P3,TBC1D29P	Height (baseline)	GCST90565843
rs8068104	9.00E-51	RNU4-34P,RPL35AP35	platelet count (maximum, inv-norm transformed)	GCST90480650
rs3794806	1.00E-50	BLMH	F3 protein levels	GCST90469170
rs8068104	6.00E-49	RNU4-34P,RPL35AP35	mean platelet volume (MPV, minimum, inv-norm transformed)	GCST90475521
rs542939	3.00E-48	ABHD15,ABHD15-AS1	Height	GCST90245844
rs3110494	4.00E-48	ABHD15-AS1,SSH2	Vertex-wise cortical surface area	GCST90095130
rs6505216	5.00E-48	ATAD5	Whole body fat free mass (UKB data field 23101)	GCST90428120
rs17885309	1.00E-47	NF1	OMG protein levels	GCST90470124
rs7208859	4.00E-47	ADAP2	Mean corpuscular volume (UKB data field 30040)	GCST90468086
rs7208859	2.00E-46	ADAP2	Mean corpuscular haemoglobin (UKB data field 30050)	GCST90468084
rs1808923	2.00E-46	ABHD15-AS1,SSH2	Lung function (FEV1/FVC)	GCST90244094
rs10468576	3.00E-46	TAOK1	Blood cell traits latent factor 5 (platelet)	GCST90559247
rs9890032	8.00E-46	ATAD5	Anthropometric traits (multi-trait analysis)	GCST007490
rs7208859	2.00E-45	ADAP2	Mean corpuscular hemoglobin	GCST007068
rs56357032	6.00E-45	NSRP1	Height	GCST007841
rs7208859	2.00E-43	ADAP2	Mean corpuscular volume	GCST90002392
rs117870634	2.00E-42	ADAP2	Height (baseline)	GCST90565843
rs2058284	3.00E-42	RNU6-990P,CPD	BLMH protein levels	GCST90468447
rs2244592	5.00E-42	SSH2	Lung function (FEV1/FVC)	GCST007431
rs35914520	5.00E-42	TAOK1	Platelet forward scatter distribution width	GCST90281197
rs3760318	3.00E-41	ADAP2	Height	GCST002647
rs189598890	7.00E-41	BLMH	BLMH protein levels	GCST90468447
rs2244592	2.00E-40	SSH2	FEV1 FVC ratio Z score (UKB data field 20258)	GCST90468165
rs3760456	3.00E-40	ABHD15-AS1,CORO6	Heel bone mineral density	GCST006433
rs7208859	3.00E-40	ADAP2	mean corpuscular hemoglobin (MCH, minimum, inv-norm transformed)	GCST90475450
rs12945042	5.00E-40	SLC6A4,SNORD63	CXADR protein levels	GCST90468922
rs9900684	2.00E-39	TAOK1	Mean platelet volume	GCST90002395
rs7222766	4.00E-39	RNU4-34P,RPL35AP35	CPXM1/HBEGF protein level ratio	GCST90314219
rs10712682	5.00E-39	RNU4-34P,RPL35AP35	Blood cell traits latent factor 5 (platelet)	GCST90559247
rs3110494	6.00E-39	ABHD15-AS1,SSH2	Brain morphology (MOSTest)	GCST90239729
rs7222766	2.00E-38	RNU4-34P,RPL35AP35	HBEGF/PDGFA protein level ratio	GCST90315033
rs8076739	5.00E-38	RNU4-34P,RPL35AP35	Mean platelet volume	GCST001335
rs183121783	6.00E-38	ADAP2	BLMH protein levels	GCST90468447
rs17767418	2.00E-37	GOSR1	Height	GCST90245844
rs3760456	3.00E-37	ABHD15-AS1,CORO6	Heel bone mineral density	GCST007066
rs2259855	3.00E-37	SSH2	White blood cell count	GCST90002374
rs149237084	3.00E-37	KRT17P3,SMURF2P1	Height (baseline)	GCST90565843
rs7222766	1.00E-36	RNU4-34P,RPL35AP35	CCN2/HBEGF protein level ratio	GCST90313713
rs76422200	2.00E-36	SLC6A4,SNORD63	Height (baseline)	GCST90565843
rs7208859	4.00E-36	ADAP2	Mean corpuscular volume	GCST90018966
rs6505239	8.00E-36	RAB11FIP4	BLMH protein levels	GCST90468447
rs146534500	1.00E-35	BLMH	BLMH protein levels	GCST90468447
rs7210152	2.00E-35	SSH2	White blood cell count	GCST90002378
rs56357032	4.00E-35	NSRP1	Height (baseline)	GCST90565843
rs7208859	8.00E-35	ADAP2	mean corpuscular volume (MCV, mean, inv-norm transformed)	GCST90475470
rs6505216	9.00E-35	ATAD5	Weight (mean, inv-normal transformed)	GCST90480727
rs551929459	1.00E-34	ATAD5	Mean corpuscular hemoglobin	GCST90002390
rs7223535	1.00E-34	ATAD5	Waist circumference adjusted for body mass index	GCST009867
rs72823964	2.00E-34	SSH2	Height (baseline)	GCST90565843
rs7210152	6.00E-34	SSH2	Monocyte count (UKB data field 30130)	GCST90468090
rs117654588	1.00E-33	NF1	BLMH protein levels	GCST90468447
rs7223535	4.00E-33	ATAD5	Multi-trait sex score	GCST90270116
rs6505216	4.00E-33	ATAD5	Weight (maximum, inv-normal transformed)	GCST90480726
rs6505216	2.00E-32	ATAD5	Weight (minimum, inv-normal transformed)	GCST90480728
rs2008998	4.00E-32	MIR4733HG	Total cholesterol levels	GCST90239673
rs4795583	6.00E-32	NF1	Height (baseline)	GCST90565843
rs72809854	6.00E-32	TBC1D29P,ALOX12P1	Height (baseline)	GCST90565843
rs76515799	6.00E-32	SSH2	White blood cell count	GCST90002407
rs10568213	1.00E-31	SSH2	Monocyte count	GCST90018967
rs7208859	1.00E-31	ADAP2	mean corpuscular volume (MCV, maximum, inv-norm transformed)	GCST90475466
rs116999970	2.00E-31	TAOK1	Mean platelet thrombocyte volume (UKB data field 30100)	GCST90468087
rs3760456	2.00E-31	ABHD15-AS1,CORO6	Whole brain restricted isotropic diffusion (multivariate analysis)	GCST90131904
rs74677442	3.00E-31	EFCAB5	Height (baseline)	GCST90565843

rs188108837	4.00E-31	MYO18A,TWF1P1	Platelet count (UKB data field 30080)	GCST90468095
rs62068776	4.00E-31	RNF135	Whole body fat free mass (UKB data field 23101)	GCST90428120
rs12943365	6.00E-31	NF1	Sex hormone-binding globulin levels	GCST90012111
rs11656408	2.00E-30	BLMH,TMIGD1	X-14658 levels	GCST90245593
rs8069759	2.00E-30	RN7SL316P,SUZ12P1	Whole body fat free mass (UKB data field 23101)	GCST90428120
rs3764419	4.00E-30	ATAD5	Multi-trait sex score	GCST90270116
rs80063027	5.00E-30	TAOK1	Blood cell traits latent factor 5 (platelet)	GCST90559247
rs12943365	6.00E-30	NF1	Sex hormone-binding globulin levels adjusted for BMI	GCST90012110
rs2008998	8.00E-30	MIR4733HG	Low density lipoprotein cholesterol levels	GCST90239655
rs201971667	9.00E-30	ABHD15,ABHD15-AS1,TP53I13	Height	GCST90435412
rs139846172	1.00E-29	TMIGD1,Y_RNA	BLMH protein levels	GCST90468447
rs35494186	2.00E-29	TAOK1	Blood cell traits latent factor 5 (platelet)	GCST90559247
rs3760456	3.00E-29	ABHD15-AS1,CORO6	Heel bone mineral density	GCST006979
rs7208859	3.00E-29	ADAP2	Mean spheric corpuscular volume	GCST90002397
rs143006132	3.00E-29	ATAD5	BLMH protein levels	GCST90468447
rs3809789	6.00E-29	ABHD15-AS1,SSH2	Whole brain restricted directional diffusion (multivariate analysis)	GCST90131905
rs13723	1.00E-28	CORO6,ABHD15-AS1	Brain morphology (MOSTest)	GCST010703
rs2905801	1.00E-28	NF1	Sex hormone-binding globulin levels adjusted for BMI	GCST90012108
rs6505216	1.00E-28	ATAD5	Body size (confirmatory factor analysis Factor 21)	GCST90309355
rs113059482	2.00E-28	ABHD15-AS1,SSH2	Height (baseline)	GCST90565843
rs6505216	2.00E-28	ATAD5	What is your weight? (pounds, inv-normal transformed)	GCST90480729
rs11656408	3.00E-28	BLMH,TMIGD1	Glycochenodeoxycholate sulfate levels	GCST90245229
rs2905801	4.00E-28	NF1	Sex hormone-binding globulin levels	GCST90012109
rs9890032	4.00E-28	ATAD5	Multi-trait sex score	GCST90270118
rs7208859	4.00E-28	ADAP2	mean corpuscular volume (MCV, minimum, inv-norm transformed)	GCST90475474
rs117221215	6.00E-28	TMIGD1	GPA33 protein levels	GCST90469385
rs1048317	6.00E-28	NF1	Type 2 diabetes	GCST90492734
rs2138852	7.00E-28	RNU4-34P,RPL35AP35	Mean platelet volume	GCST000305
rs7223535	2.00E-27	ATAD5	Fat-free mass	GCST007063
rs536327	2.00E-27	TAOK1	F2R/SPINT2 protein level ratio	GCST90314737
rs62070648	6.00E-27	ATAD5	Lung function (FVC)	GCST007081
rs148894198	6.00E-27	BLMH	BLMH protein levels	GCST90468447
rs182158610	6.00E-27	SMURF2P1,KRT17P3	BLMH protein levels	GCST90468447
rs3760318	6.00E-27	ADAP2	Height	GCST90245846
rs8080078	1.00E-26	SSH2	Heel bone mineral density	GCST006433
rs191010498	2.00E-26	TAOK1	Mean platelet volume	GCST004599
rs72811661	3.00E-26	ATAD5	Height	GCST90435412
rs79749090	3.00E-26	MYO18A	BLMH protein levels	GCST90468447
rs1388175	3.00E-26	MYO18A	Platelet count	GCST90056183
rs495814	4.00E-26	ABHD15,TAOK1	HBEGF protein levels	GCST90469433
rs11310424	6.00E-26	SUZ12P1	Mean platelet volume	GCST90002395
rs60170945	6.00E-26	TAOK1	Blood cell traits latent factor 5 (platelet)	GCST90559247
rs117165347	9.00E-26	RNU4-34P,RPL35AP35	Mean platelet thrombocyte volume (UKB data field 30100)	GCST90468087
rs62070645	9.00E-26	ATAD5	Weight	GCST90018949
rs1973252	1.00E-25	RN7SL316P,SUZ12P1	Platelet distribution width (UKB data field 30110)	GCST90468097
rs8067252	1.00E-25	ADAP2	Lung function (forced vital capacity)	GCST90244093
rs2854332	2.00E-25	RAB11FIP4,NF1	Appendicular lean mass	GCST90000025
rs542939	3.00E-25	ABHD15,ABHD15-AS1	Physical function (baseline)	GCST90565837
rs6505216	3.00E-25	ATAD5	Height (maximum, inv-normal transformed)	GCST90475358
rs111619531	5.00E-25	PIPOX,MYO18A	Intrinsic epigenetic age acceleration	GCST90014296
rs7218758	6.00E-25	RNU4-34P,RPL35AP35	Red cell distribution width	GCST007074
rs8614	7.00E-25	NUFIP2	Smoking initiation	GCST90243968
rs3115094	8.00E-25	GIT1,ABHD15-AS1	Height (baseline)	GCST90565843
rs12945088	8.00E-25	MIR4733HG	Total cholesterol levels	GCST90239676
rs2250320	9.00E-25	SSH2	Neutrophil count	GCST90002351
rs6505211	1.00E-24	CRLF3	Platelet distribution width	GCST90002401
rs139469282	1.00E-24	MIR4733HG	Mean platelet thrombocyte volume (UKB data field 30100)	GCST90468087
rs178867	1.00E-24	RAB11FIP4	Height (baseline)	GCST90565843
rs151317021	1.00E-24	SSH2	Heel bone mineral density	GCST006433
rs72823964	1.00E-24	SSH2	Appendicular lean mass	GCST90000025
rs117221215	1.00E-24	TMIGD1	EPCAM protein levels	GCST90469126
rs6505216	2.00E-24	ATAD5	height (mean, inv-normal transformed)	GCST90475361
rs3110494	3.00E-24	ABHD15-AS1,SSH2	Whole brain free water diffusion (multivariate analysis)	GCST90131906
rs184447505	3.00E-24	CPD	BLMH protein levels	GCST90468447
rs33980254	4.00E-24	BLMH	Glycohyocholate levels	GCST90245234
rs1129506	4.00E-24	EVI2A,NF1	Sex hormone-binding globulin levels	GCST90239821
rs2940182	5.00E-24	MIR4733HG	Non-HDL cholesterol levels	GCST90239667
rs11651127	1.00E-23	RNU4-34P,TAOK1	Mean platelet thrombocyte volume (UKB data field 30100)	GCST90468087
rs72811657	1.00E-23	ATAD5	Height (baseline)	GCST90565843
rs11650795	1.00E-23	EFCAB5	Height (baseline)	GCST90565843
rs7211375	1.00E-23	PIPOX	Intrinsic epigenetic age acceleration	GCST90014296
rs880749	1.00E-23	SSH2	monocyte (absolute count, mean, inv-norm transformed)	GCST90479702
rs11654523	2.00E-23	ABHD15-AS1,SSH2	Height	GCST007841

rs8073965	2.00E-23	SLC6A4	Height (baseline)	GCST90565843
rs138723501	3.00E-23	GIT1,ABHD15-AS1	GIT1 protein levels	GCST90469351
rs56853305	3.00E-23	NUFIP2,RPL35AP35	Total cholesterol levels	GCST90239673
rs4310926	5.00E-23	EFCAB5	Cortical surface area (MOSTest)	GCST010701
rs7503951	6.00E-23	NF1	Height	GCST007841
rs11412443	9.00E-23	SSH2	Edge-level brain connectivity (multivariate analysis)	GCST90165318
rs2138852	1.00E-22	RNU4-34P,RPL35AP35	Mean platelet volume	GCST000497
rs12602426	1.00E-22	SSH2	Neutrophil count	GCST90002355
rs149777351	1.00E-22	RNU4-34P,RPL35AP35	Standing height (UKB data field 50)	GCST90468178
rs12945088	2.00E-22	MIR4733HG	Low density lipoprotein cholesterol levels	GCST90239658
rs141906452	2.00E-22	RNU4-34P,RPL35AP35	BLMH protein levels	GCST90468447
rs797967	2.00E-22	NUFIP2	Height	GCST90245844
rs7208859	3.00E-22	ADAP2	Mean reticulocyte volume	GCST90002396
rs145759993	3.00E-22	TWF1P1,CRYBA1	BLMH protein levels	GCST90468447
rs556340201	6.00E-22	ADAP2	BLMH protein levels	GCST90468447
rs35958868	6.00E-22	ADAP2	Hip circumference adjusted for BMI	GCST012227
rs7208859	6.00E-22	ADAP2	mean corpuscular hemoglobin (MCH, mean, inv-norm transformed)	GCST90479673
rs2001287	9.00E-22	NF1	Gamma glutamyl transferase levels	GCST90428730
rs3764419	2.00E-21	ATAD5	Height	GCST000817
rs2952983	2.00E-21	NF1	Testosterone levels	GCST90483487
rs2087634	3.00E-21	PIPOX	Mean platelet thrombocyte volume (UKB data field 30100)	GCST90468087
rs7208859	3.00E-21	ADAP2	Mean sphered cell volume (UKB data field 30270)	GCST90468089
rs149777351	3.00E-21	RNU4-34P,RPL35AP35	Height (baseline)	GCST90565843
rs8614	3.00E-21	NUFIP2	Educational attainment	GCST90105038
rs952190	4.00E-21	RNF135	Height (baseline)	GCST90565843
rs181607280	4.00E-21	ABHD15,TAOK1	BLMH protein levels	GCST90468447
rs11370020	4.00E-21	SSH2	Chronic obstructive pulmonary disease liability (machine learning-based score)	GCST90244098
rs59015798	4.00E-21	NF1	Testosterone levels	GCST90483498
rs146185988	4.00E-21	TAOK1	Blood cell traits latent factor 18 (platelet)	GCST90559260
rs148378911	5.00E-21	NF1	Height (baseline)	GCST90565843
rs8080078	5.00E-21	SSH2	Brain shape (segment 62)	GCST90012941
rs638730	1.00E-20	TAOK1	Blood cell traits latent factor 5 (platelet)	GCST90559247
rs57812147	2.00E-20	TMIGD1,BLMH	Appendicular lean mass	GCST90000025
rs8069884	2.00E-20	SUZ12P1,RN7SL316P	Lung function (FEV1)	GCST90244092
rs138472821	3.00E-20	RNU4-34P,RPL35AP35	Height	GCST007841
rs17884401	3.00E-20	NF1	Height (baseline)	GCST90565843
rs141870697	3.00E-20	CRLF3	Height (baseline)	GCST90565843
rs12939322	4.00E-20	RNF135,MIR4733HG	Height	GCST90245848
rs34216701	6.00E-20	SSH2	Monocyte percentage of white cells	GCST90002394
rs7208859	6.00E-20	ADAP2	mean corpuscular hemoglobin (MCH, minimum, inv-norm transformed)	GCST90479674
rs880749	7.00E-20	SSH2	White blood cell count	GCST007070
rs6505216	7.00E-20	ATAD5	height (minimum, inv-normal transformed)	GCST90475364
rs7208859	1.00E-19	ADAP2	Mean corpuscular volume	GCST004602
rs552761103	1.00E-19	NF1	Platelet count	GCST90002402
rs145046282	1.00E-19	TAOK1	Standing height (UKB data field 50)	GCST90468178
rs2940181	1.00E-19	MIR4733HG	Non-HDL cholesterol levels	GCST90239670
rs67546072	1.00E-19	TAOK1	Blood cell traits latent factor 5 (platelet)	GCST90559247
rs72815624	2.00E-19	NF1,OMG	General risk tolerance (MTAG)	GCST007325
rs113074297	2.00E-19	ADAP2	Height (baseline)	GCST90565843
rs117870634	2.00E-19	ADAP2	Physical function (baseline)	GCST90565837
rs56336338	2.00E-19	RPL35AP35,NUFIP2	Low density lipoprotein cholesterol levels	GCST90239655
rs111970489	2.00E-19	RN7SL316P,SUZ12P1	Multi-trait sex score	GCST90270118
rs565366829	3.00E-19	TAOK1	Mean platelet volume	GCST90002345
rs191010498	3.00E-19	TAOK1	Platelet count	GCST90002402
rs62068776	3.00E-19	RNF135	Multi-trait sex score	GCST90270116
rs7208859	3.00E-19	ADAP2	mean corpuscular hemoglobin (MCH, maximum, inv-norm transformed)	GCST90479672
rs7210288	3.00E-19	SSH2	monocyte (absolute count, minimum, inv-norm transformed)	GCST90479703
rs529223795	4.00E-19	LRRC37BP1,SH3GL1P2	Height (baseline)	GCST90565843
rs2905801	4.00E-19	NF1	Total testosterone levels	GCST90012113
rs34102989	5.00E-19	TAOK1	Blood cell traits latent factor 5 (platelet)	GCST90559247
rs7210288	5.00E-19	SSH2	white blood cell count (WBC, minimum, inv-norm transformed)	GCST90480725
rs2905804	6.00E-19	NF1	Adventurousness	GCST007324
rs62068776	6.00E-19	RNF135	Hip circumference adjusted for BMI	GCST90020028
rs34977734	6.00E-19	RNU4-34P,RPL35AP35	Blood cell traits latent factor 5 (platelet)	GCST90559247
rs200309755	7.00E-19	TAOK1	FKBP1B protein levels	GCST90469242
rs762452535	8.00E-19	RPL35AP35,NUFIP2	Red blood cell erythrocyte distribution width (UKB data field 30070)	GCST90468099
rs12943365	9.00E-19	NF1	Monocyte count	GCST90002393
rs141906452	9.00E-19	RNU4-34P,RPL35AP35	Mean platelet thrombocyte volume (UKB data field 30100)	GCST90468087
rs7208859	1.00E-18	ADAP2	Mean corpuscular hemoglobin	GCST004630
rs3840872I	1.00E-18	ABHD15-AS1,SSH2	Lymphocyte count	GCST90002316
rs7208859	1.00E-18	ADAP2	Mean reticulocyte volume (UKB data field 30260)	GCST90468088
rs7503951	1.00E-18	NF1	Height (baseline)	GCST90565843
rs34530466	1.00E-18	TAOK1	Blood cell traits latent factor 5 (platelet)	GCST90559247

rs7210288	1.00E-18	SSH2	white blood cell count (WBC, mean, inv-norm transformed)	GCST90480724
rs117108035	2.00E-18	TMIGD1,BLMH	Height (baseline)	GCST90565843
rs3764449	2.00E-18	BLMH,TMIGD1	BLMH protein levels	GCST90277710
rs768073610	2.00E-18	TAOK1	Mean platelet thrombocyte volume (UKB data field 30100)	GCST90468087
rs8081496	2.00E-18	RN7SL316P,LRRC37BP1	Multi-trait sex score	GCST90270118
rs559972	3.00E-18	TAOK1	Platelet count	GCST001337
rs9907984	3.00E-18	TAOK1	Platelet count	GCST90002402
rs7210399	3.00E-18	TAOK1	MYH9 protein levels	GCST90469975
rs2905801	3.00E-18	NF1	Total testosterone levels	GCST90239819
rs547178969	4.00E-18	TAOK1	Blood cell traits latent factor 18 (platelet)	GCST90559260
rs2952985	4.00E-18	NF1	total cholesterol (mean, inv-norm transformed)	GCST90480715
rs149416569	5.00E-18	ATAD5	Height (baseline)	GCST90565843
rs7342976	5.00E-18	ATAD5	Hip circumference adjusted for BMI	GCST90020028
rs3794806	5.00E-18	BLMH	Glycochenodeoxycholate 3-sulfate levels	GCST90200022
rs62070270	7.00E-18	EFCAB5	Lung function (FEV1/FVC)	GCST004185
rs181126944	7.00E-18	BLMH	BLMH protein levels	GCST90468447
rs62070652	7.00E-18	ATAD5	Knee osteoarthritis	GCST90566800
rs12603885	1.00E-17	NF1	Apolipoprotein B levels	GCST010243
rs7504112	1.00E-17	SUZ12P1	Waist circumference adjusted for body mass index	GCST009867
rs113934718	1.00E-17	ATAD5	Forced vital capacity FVC Z score (UKB data field 20257)	GCST90468167
rs72811632	1.00E-17	SMURF2P1,SH3GL1P2	Standing height (UKB data field 50)	GCST90468178
rs3110494	1.00E-17	ABHD15-AS1,SSH2	Cortical surface area	GCST90091060
rs9911167	1.00E-17	ANKRD13B,ABHD15-AS1	Cortical thickness	GCST90091061
rs3760318	1.00E-17	ADAP2	Waist circumference adjusted for body mass index	GCST012226
rs200309755	1.00E-17	TAOK1	HSBP1 protein levels	GCST90469481
rs4474742	2.00E-17	SSH2	Monocyte count	GCST004625
rs3809790	2.00E-17	ABHD15-AS1,SSH2	Lymphocyte count	GCST90002320
rs141870697	2.00E-17	CRLF3	Mean platelet thrombocyte volume (UKB data field 30100)	GCST90468087
rs9893872	2.00E-17	NF1	Gamma glutamyl transpeptidase	GCST90018954
rs755174191	2.00E-17	TAOK1	Mean platelet thrombocyte volume (UKB data field 30100)	GCST90468087
rs80168287	2.00E-17	SUZ12P1,CRLF3	Mean corpuscular hemoglobin	GCST90018744
rs78899080	2.00E-17	MYO18A	Height (baseline)	GCST90565843
rs9901272	2.00E-17	ANKRD13B,ABHD15-AS1	Height (baseline)	GCST90565843
rs148033030	2.00E-17	RAB11FIP4	BLMH protein levels	GCST90468447
rs117757092	2.00E-17	CRYBA1	Wears glasses or contact lenses (UKB data field 2207)	GCST90042875
rs56336338	2.00E-17	RPL35AP35,NUFIP2	Total cholesterol levels	GCST90239676
rs11656408	2.00E-17	BLMH,TMIGD1	S100A16 protein levels	GCST90470516
rs7208859	2.00E-17	ADAP2	mean corpuscular volume (MCV, mean, inv-norm transformed)	GCST90479676
rs9916613	3.00E-17	Y_RNA,RNU6-990P	Apolipoprotein A1 levels	GCST010241
rs4258685	3.00E-17	RAB11FIP4	Whole body water mass (UKB data field 23102)	GCST90468184
rs7210867	3.00E-17	TAOK1	Blood cell traits latent factor 18 (platelet)	GCST90559260
rs145110672	4.00E-17	RPL35AP35,RNU4-34P	Mean platelet thrombocyte volume (UKB data field 30100)	GCST90468087
rs112707384	5.00E-17	KRT17P3,TBC1D29P	Multi-trait sex score	GCST90270118
rs75204514	5.00E-17	SSH2	monocyte (absolute count, maximum, inv-norm transformed)	GCST90479701
rs140233429	6.00E-17	BLMH	BLMH protein levels	GCST90468447
rs112953042	6.00E-17	NF1	Whole body fat free mass (UKB data field 23101)	GCST90428120
rs117133183	7.00E-17	NSRP1,EFCAB5	BLMH protein levels	GCST90468447
rs7216243	9.00E-17	TAOK1	Mean platelet volume during first trimester of pregnancy	GCST90302229
rs56336338	9.00E-17	RPL35AP35,NUFIP2	total cholesterol (mean, inv-norm transformed)	GCST90476424
rs61127551	1.00E-16	ABHD15-AS1,SSH2	Height (baseline)	GCST90565843
rs7223535	1.00E-16	ATAD5	Hip shape mode 2	GCST90482705
rs150916102	2.00E-16	SSH2	White blood cell count	GCST90018978
rs547816076	2.00E-16	NUFIP2,RPL35AP35	Mean platelet thrombocyte volume (UKB data field 30100)	GCST90468087
rs112707384	2.00E-16	KRT17P3,TBC1D29P	Physical function (baseline)	GCST90565837
rs36070425	2.00E-16	TAOK1	Inflammatory markers and poor diet (confirmatory factor analysis Factor 30)	GCST90309364
rs200309755	2.00E-16	TAOK1	Mitochondrial DNA copy number	GCST90026371
rs7223535	3.00E-16	ATAD5	Fat-free mass	GCST007063
rs117032306	3.00E-16	TAOK1	Platelet count	GCST90056183
rs62068776	3.00E-16	RNF135	Waist circumference adjusted for body mass index	GCST012226
rs10591199	3.00E-16	TAOK1	Blood cell traits latent factor 5 (platelet)	GCST90559247
rs3809789	4.00E-16	ABHD15-AS1,SSH2	Lymphocyte count	GCST90002388
rs10512432	4.00E-16	GOSR1	Apolipoprotein A levels (UKB data field 30630)	GCST90468061
rs112588760	4.00E-16	EFCAB5	BLMH protein levels	GCST90468447
rs533654721	4.00E-16	TMIGD1	Protein S100-A14 levels	GCST90249404
rs79461387	5.00E-16	ATAD5	Neutrophil percentage of white cells	GCST90002399
rs149237084	6.00E-16	KRT17P3,SMURF2P1	Physical function (baseline)	GCST90565837
rs61296342	6.00E-16	NF1	Whole body fat free mass (UKB data field 23101)	GCST90428120
rs144092611	7.00E-16	CPD,GOSR1	Height (baseline)	GCST90565843
rs56336338	7.00E-16	RPL35AP35,NUFIP2	Low density lipoprotein cholesterol levels	GCST90239658
rs113770589	7.00E-16	NF1	Whole body fat free mass (UKB data field 23101)	GCST90428120
rs4795591	7.00E-16	OMG,NF1	total cholesterol (maximum, inv-norm transformed)	GCST90480714
rs11872020	8.00E-16	SLC6A4	Leg fat percentage right (UKB data field 23111)	GCST90468175
rs11080134	8.00E-16	ATAD5	Multi-trait sex score	GCST90270116

rs7210604	8.00E-16	NF1,EVI2B	Whole body fat free mass (UKB data field 23101)	GCST90428120
rs117202101	9.00E-16	NF1	BLMH protein levels	GCST90468447
rs72815614	9.00E-16	NF1,OMG	Smoking initiation	GCST90243985
rs9894551	1.00E-15	TAOK1,ABHD15	Glucose levels (UKB data field 30740)	GCST90468071
rs183300440	1.00E-15	MIR4733HG	Mean platelet thrombocyte volume (UKB data field 30100)	GCST90468087
rs4258685	1.00E-15	RAB11FIP4	Basal metabolic rate (UKB data field 23105)	GCST90468159
rs1038088	1.00E-15	SSH2	Body fat percentage	GCST90020232
rs80063027	1.00E-15	TAOK1	Blood cell traits latent factor 18 (platelet)	GCST90559260
rs7208859	1.00E-15	ADAP2	mean corpuscular volume (MCV, minimum, inv-norm transformed)	GCST90479677
rs2952985	1.00E-15	NF1	total cholesterol (minimum, inv-norm transformed)	GCST90480716
rs201740279	2.00E-15	ABHD15-AS1,SSH2	Neutrophil count	GCST90002398
rs2040792	2.00E-15	NF1	Type 2 diabetes	GCST010557
rs11871003	2.00E-15	TAOK1	Mean platelet thrombocyte volume (UKB data field 30100)	GCST90468087
rs35278712	2.00E-15	NF1,OMG	Cholesterol levels (UKB data field 30690)	GCST90468066
rs185838251	2.00E-15	SSH2	Mean platelet thrombocyte volume (UKB data field 30100)	GCST90468087
rs112178027	2.00E-15	CRYBA1,TWF1P1	Platelet count	GCST90056183
rs62068776	2.00E-15	RNF135	Multi-trait sex score	GCST90270118
rs7223535	3.00E-15	ATAD5	Fat-free mass	GCST007063
rs71372238	3.00E-15	CRLF3	Mean platelet thrombocyte volume (UKB data field 30100)	GCST90468087
rs149707369	3.00E-15	EFCAB5	Vitronectin levels	GCST90161304
rs3103307	3.00E-15	TMIGD1	Educational attainment	GCST90105038
rs35974893	3.00E-15	NF1	Hip circumference adjusted for BMI	GCST012227
rs7223535	3.00E-15	ATAD5	Uterine leiomyoma or breast cancer (pleiotropy)	GCST90134420
rs7223535	3.00E-15	ATAD5	Osteoarthritis	GCST90566795
rs76422200	3.00E-15	SLC6A4,SNORD63	Whole body fat free mass (UKB data field 23101)	GCST90428120
rs55885610	4.00E-15	CRYBA1	Mean reticulocyte volume	GCST90002396
rs7223373	4.00E-15	CRLF3	Hip circumference adjusted for BMI	GCST90020028
rs3764419	4.00E-15	ATAD5	Waist circumference adjusted for body mass index	GCST90020029
rs62070628	4.00E-15	SUZ12P1	Waist circumference adjusted for body mass index	GCST012226
rs145952101	4.00E-15	RN7SL316P,LRRC37BP1	SLC51B protein levels	GCST90470664
rs6505173	4.00E-15	TMIGD1	Taurolithocholate 3-sulfate levels	GCST90245450
rs7208859	4.00E-15	ADAP2	red cell diameter width (RDW, maximum, inv-norm transformed)	GCST90476357
rs62070648	5.00E-15	ATAD5	Lung function (FVC)	GCST007429
rs9913470	5.00E-15	NF1	Total testosterone levels	GCST90012114
rs7214290	5.00E-15	NUFIP2	White blood cell count	GCST90002407
rs7208859	5.00E-15	ADAP2	mean corpuscular volume (MCV, maximum, inv-norm transformed)	GCST90479675
rs112178027	6.00E-15	CRYBA1,TWF1P1	hormone-binding globulin levels adjusted for BMI and heel estimated bone mineral de	GCST90399398
rs76479286	7.00E-15	TAOK1	Mean platelet thrombocyte volume (UKB data field 30100)	GCST90468087
rs183783019	7.00E-15	TAOK1	Red blood cell erythrocyte distribution width (UKB data field 30070)	GCST90468099
rs74815160	7.00E-15	CRLF3,ATAD5	Height	GCST90278637
rs200309755	8.00E-15	TAOK1	CALCOCO2 protein levels	GCST90468531
rs9915622	8.00E-15	TAOK1	Mean platelet volume during second trimester of pregnancy	GCST90302230
rs10645005	8.00E-15	CRLF3	Multi-trait sex score	GCST90270118
rs35958868	9.00E-15	ADAP2	Height	GCST008163
rs187678828	9.00E-15	CPD	BLMH protein levels	GCST90468447
rs3764419	9.00E-15	ATAD5	Hip circumference adjusted for BMI	GCST012227
rs12603885	1.00E-14	NF1	LDL cholesterol levels	GCST010245
rs117662433	1.00E-14	ATAD5	Mean corpuscular hemoglobin	GCST90002390
rs62066572	1.00E-14	TAOK1	Mean platelet thrombocyte volume (UKB data field 30100)	GCST90468087
rs4368212	1.00E-14	EVI2B,NF1	Aspartate aminotransferase levels	GCST90018944
rs56336338	1.00E-14	RPL35AP35,NUFIP2	Neutrophill count (UKB data field 30140)	GCST90468092
rs11656408	1.00E-14	BLMH,TMIGD1	Plasma glycochenodeoxycholate 3-sulfate levels in chronic kidney disease	GCST90265282
rs138818953	1.00E-14	ABHD15,ABHD15-AS1	Standing height (UKB data field 50)	GCST90468178
rs140020617	1.00E-14	NF1	BLMH protein levels	GCST90468447
rs62070804	1.00E-14	ABHD15-AS1,ABHD15	Waist-to-hip ratio adjusted for BMI	GCST90020025
rs56357032	1.00E-14	NSRP1	Physical function (baseline)	GCST90565837
rs72823964	1.00E-14	SSH2	Physical function (baseline)	GCST90565837
rs200739560	1.00E-14	NUFIP2,RPL35AP35	Mean platelet volume during second trimester of pregnancy	GCST90302230
rs62070645	1.00E-14	ATAD5	Prostate-specific antigen levels	GCST90461907
rs55797987	2.00E-14	SSH2,ABHD15-AS1	Hemoglobin concentration	GCST90002314
rs8081187	2.00E-14	LRRC37BP1,RN7SL316P	Waist circumference adjusted for body mass index	GCST009867
rs2244592	2.00E-14	SSH2	Peak expiratory flow	GCST007430
rs13723	2.00E-14	CORO6,ABHD15-AS1	Cortical thickness (MOSTest)	GCST010700
rs56853305	2.00E-14	NUFIP2,RPL35AP35	Mean reticulocyte volume (UKB data field 30260)	GCST90468088
rs187468027	2.00E-14	NSRP1	BLMH protein levels	GCST90468447
rs62070804	2.00E-14	ABHD15-AS1,ABHD15	Waist-hip index	GCST90020027
rs73267833	2.00E-14	RN7SL316P,LRRC37BP1	Physical function (baseline)	GCST90565837
rs3760456	2.00E-14	ABHD15-AS1,CORO6	T1 brain MRIs Unsupervised Deep learning derived Imaging Phenotypes (dimension 47	GCST90320610
rs12603885	3.00E-14	NF1	Low density lipoprotein cholesterol levels	GCST010204
rs7222696	3.00E-14	RPL35AP35,RNU4-34P	Heel bone mineral density	GCST006433
rs1038088	3.00E-14	SSH2	Metabolic biomarkers (multivariate analysis)	GCST90038594
rs62070652	3.00E-14	ATAD5	Hip minimal joint space width	GCST90281365
rs11080134	3.00E-14	ATAD5	Telomere length (principal component 1)	GCST90435144

rs12450936	3.00E-14	MYO18A	Height	GCST90245848
rs71142016	4.00E-14	NF1	Direct low density lipoprotein levels (UKB data field 30780)	GCST90468080
rs734403	4.00E-14	RAB11FIP4	Total cholesterol levels	GCST90018974
rs62070631	4.00E-14	SUZ12P1	Hip circumference adjusted for BMI	GCST012227
rs797971	4.00E-14	RPL35AP35,NUFIP2	Type 2 diabetes	GCST90492734
rs2952985	4.00E-14	NF1	low density lipoprotein cholesterol (LDLC, mean, inv-norm transformed)	GCST90479661
rs8068104	4.00E-14	RNU4-34P,RPL35AP35	platelet count (mean, inv-norm transformed)	GCST90476297
rs3110494	5.00E-14	ABHD15-AS1,SSH2	Brain region volumes	GCST009518
rs1048317	5.00E-14	NF1	Drinks per week	GCST90243989
rs1048317	6.00E-14	NF1	Type 2 diabetes	GCST90132183
rs3760318	6.00E-14	ADAP2	Diastolic blood pressure	GCST90292474
rs11080150	7.00E-14	NF1	Total cholesterol levels	GCST009145
rs34030976	7.00E-14	TAOK1	Blood cell traits latent factor 5 (platelet)	GCST90559247
rs1038088	7.00E-14	SSH2	Body mass index	GCST90255621
rs56336338	7.00E-14	RPL35AP35,NUFIP2	total cholesterol (minimum, inv-norm transformed)	GCST90476427
rs548458037	8.00E-14	RNU4-34P,RPL35AP35	Blood cell traits latent factor 5 (platelet)	GCST90559247
rs3764419	9.00E-14	ATAD5	Height	GCST001956
rs28636673	9.00E-14	SUZ12P1,RN7SL316P	Multi-trait sex score	GCST90270116
rs146171833	1.00E-13	EFCAB5	Height	GCST007841
rs10512432	1.00E-13	GOSR1	Haematocrit percentage (UKB data field 30030)	GCST90468073
rs4638642	1.00E-13	EVI2B,NF1	Aspartate aminotransferase levels	GCST90011899
rs7220770	1.00E-13	RN7SL316P,LRRC37BP1	Waist circumference adjusted for body mass index	GCST012226
rs12943365	1.00E-13	NF1	Smoking initiation	GCST90243968
rs56336338	1.00E-13	RPL35AP35,NUFIP2	total cholesterol (maximum, inv-norm transformed)	GCST90476420
rs8068104	1.00E-13	RNU4-34P,RPL35AP35	platelet count (minimum, inv-norm transformed)	GCST90476300
rs6505216	1.00E-13	ATAD5	Weight (maximum, inv-normal transformed)	GCST90476459
rs25533	2.00E-13	SLC6A4	Mean platelet thrombocyte volume (UKB data field 30100)	GCST90468087
rs11395406	2.00E-13	SSH2	Body mass index	GCST90018947
rs115492363	2.00E-13	LRRC37BP1,RN7SL316P	Height (baseline)	GCST90565843
rs2854332	2.00E-13	RAB11FIP4,NF1	LDL cholesterol	GCST90018961
rs7223535	2.00E-13	ATAD5	Birth weight	GCST008362
rs79461387	2.00E-13	ATAD5	Monocyte percentage (UKB data field 30190)	GCST90468091
rs148611709	2.00E-13	CRLF3	Standing height (UKB data field 50)	GCST90468178
rs189998180	2.00E-13	MIR4733HG	BLMH protein levels	GCST90468447
rs11872020	2.00E-13	SLC6A4	Leg fat percentage left (UKB data field 23115)	GCST90468174
rs7223535	2.00E-13	ATAD5	Waist circumference adjusted for body mass index	GCST90020029
rs542939	2.00E-13	ABHD15,ABHD15-AS1	Hip circumference adjusted for BMI	GCST012227
rs62068776	2.00E-13	RNF135	Hip circumference adjusted for BMI	GCST012227
rs8614	2.00E-13	NUFIP2	Cigarettes smoked per day	GCST90243987
rs201771285	2.00E-13	TAOK1	Blood cell traits latent factor 5 (platelet)	GCST90559247
rs9890032	2.00E-13	ATAD5	Height	GCST90245845
rs1124918	2.00E-13	NF1	low density lipoprotein cholesterol (LDLC, maximum, inv-norm transformed)	GCST90479660
rs56336338	2.00E-13	RPL35AP35,NUFIP2	total cholesterol (mean, inv-norm transformed)	GCST90480715
rs2530394	3.00E-13	RPL35AP35,NUFIP2	Heel bone mineral density	GCST007066
rs137907953	3.00E-13	RNF135	Height (baseline)	GCST90565843
rs189217595	3.00E-13	KRT17P3,SMURF2P1	BLMH protein levels	GCST90468447
rs74677442	3.00E-13	EFCAB5	Physical function (baseline)	GCST90565837
rs75581564	3.00E-13	PIPOX	Depression	GCST007342
rs200309755	3.00E-13	TAOK1	SPRY2 protein levels	GCST90470738
rs8614	3.00E-13	NUFIP2	Cigarettes smoked per day	GCST90243976
rs12943365	3.00E-13	NF1	Multi-trait sex score	GCST90270118
rs55764512	4.00E-13	SUZ12P1	Hemoglobin concentration	GCST90002310
rs56336338	4.00E-13	RPL35AP35,NUFIP2	Neutrophil count	GCST90002398
rs2011614	4.00E-13	CPD	HDL cholesterol levels	GCST010242
rs1055669	4.00E-13	SUZ12P1,CRLF3	Height (baseline)	GCST90565843
rs2002094	4.00E-13	SSH2	Sex hormone-binding globulin levels adjusted for BMI	GCST90012108
rs62070651	4.00E-13	ATAD5	Forced expiratory volume in 1 second FEV1 Z score (UKB data field 20256)	GCST90468166
rs1872924	4.00E-13	SLC6A4	GPA33 protein levels	GCST90469385
rs200309755	4.00E-13	TAOK1	HHEX protein levels	GCST90469451
rs200309755	4.00E-13	TAOK1	MINK1 protein levels	GCST90469909
rs7214014	4.00E-13	BLMH	X-14626 levels	GCST90245592
rs34015426	4.00E-13	TAOK1	Blood cell traits latent factor 18 (platelet)	GCST90559260
rs111919672	5.00E-13	BLMH	Sex hormone-binding globulin levels adjusted for BMI	GCST90012110
rs35958868	5.00E-13	ADAP2	Breast cancer or ovarian cancer (pleiotropy)	GCST90270342
rs2729443	5.00E-13	SSH2	Neutrophil forward scatter	GCST90281224
rs370500262	5.00E-13	SSH2	lymphocyte (absolute count, maximum, inv-norm transformed)	GCST90475423
rs8073217	6.00E-13	SSH2	Hemoglobin	GCST90018957
rs2020934	6.00E-13	SLC6A4	Height (baseline)	GCST90565843
rs11080121	6.00E-13	SLC6A4	Resistance to COVID-19 infection (Exposed negative vs positive)	GCST90255358
rs370500262	6.00E-13	SSH2	lymphocyte (absolute count, maximum, inv-norm transformed)	GCST90479663
rs35888506	7.00E-13	NF1	Drinks per week	GCST90243984
rs2559621	7.00E-13	TAOK1	Heparin-binding EGF-like growth factor levels	GCST90247852
rs6505216	7.00E-13	ATAD5	What is your height? (cm, inv-normal transformed)	GCST90475367

rs8614	8.00E-13	NUFIP2	Glucose levels	GCST90018955
rs71360708	8.00E-13	TAOK1	CCL28 protein levels	GCST90468580
rs5819871	8.00E-13	TAOK1	Waist-hip index	GCST90020027
rs28715670	8.00E-13	SSH2	Monocyte count	GCST90056177
rs73265633	9.00E-13	ADAP2	Mean corpuscular hemoglobin	GCST90002323
rs146976827	9.00E-13	NSRP1	Mean platelet thrombocyte volume (UKB data field 30100)	GCST90468087
rs5819871	9.00E-13	TAOK1	Waist-to-hip ratio adjusted for BMI	GCST90020025
rs62070652	9.00E-13	ATAD5	Osteoarthritis (with total knee replacement)	GCST90566805
rs8073217	1.00E-12	SSH2	Red blood cell count	GCST007069
rs1038088	1.00E-12	SSH2	Body mass index	GCST009004
rs8073217	1.00E-12	SSH2	Haemoglobin concentration (UKB data field 30020)	GCST90468074
rs8073217	1.00E-12	SSH2	Hematocrit	GCST90018960
rs11872020	1.00E-12	SLC6A4	Body fat percentage (UKB data field 23099)	GCST90468160
rs188492388	1.00E-12	GOSR1	BLMH protein levels	GCST90468447
rs28617748	1.00E-12	RPL35AP35,RNU4-34P	Educational attainment	GCST90105038
rs1038088	1.00E-12	SSH2	Body mass index (MTAG)	GCST90179150
rs55920122	1.00E-12	NF1	Omega-6 fatty acid levels	GCST90502095
rs6505216	1.00E-12	ATAD5	Weight (mean, inv-normal transformed)	GCST90476462
rs2953015	1.00E-12	NF1	platelet count (mean, inv-norm transformed)	GCST90480651
rs9890032	2.00E-12	ATAD5	Hip circumference adjusted for BMI	GCST004067
rs6505239	2.00E-12	RAB11FIP4	Adventurousness	GCST007324
rs138044297	2.00E-12	LRRC37BP1,RN7SL316P	Height	GCST007841
rs9907214	2.00E-12	CRYBA1,TWF1P1	Waist circumference adjusted for body mass index	GCST009867
rs148379086	2.00E-12	TAOK1	Mean platelet thrombocyte volume (UKB data field 30100)	GCST90468087
rs1967556	2.00E-12	TAOK1	CERT protein levels	GCST90468717
rs11653826	2.00E-12	RPL35AP35,NUFIP2	Direct low density lipoprotein levels (UKB data field 30780)	GCST90468080
rs2020942	2.00E-12	SLC6A4	Arm fat percentage right (UKB data field 23119)	GCST90468158
rs8073217	2.00E-12	SSH2	Red blood cell erythrocyte count (UKB data field 30010)	GCST90468098
rs183790295	2.00E-12	SSH2,ABHD15-AS1	Standing height (UKB data field 50)	GCST90468178
rs117835451	2.00E-12	ADAP2	BLMH protein levels	GCST90468447
rs149683607	2.00E-12	KRT17P3,SMURF2P1	BLMH protein levels	GCST90468447
rs2525568	2.00E-12	NF1	Platelet count	GCST90056183
rs542939	2.00E-12	ABHD15,ABHD15-AS1	Hip circumference adjusted for BMI	GCST90020028
rs36070425	2.00E-12	TAOK1	ACADSB protein levels	GCST90468195
rs11870192	2.00E-12	TMIGD1,BLMH	Total cholesterol levels	GCST90239673
rs1872924	2.00E-12	SLC6A4	EPCAM protein levels	GCST90469126
rs35840638	2.00E-12	ADAP2	Uterine leiomyoma or ER positive breast cancer (pleiotropy)	GCST90134421
rs36070425	2.00E-12	TAOK1	MED18 protein levels	GCST90469880
rs2905787	2.00E-12	NF1	Sex hormone-binding globulin levels	GCST90027091
rs72815624	2.00E-12	NF1,OMG	Oligodendrocyte-myelin glycoprotein levels	GCST90248782
rs72811672	2.00E-12	ADAP2,LINC02978	Benign neoplasm of thyroid glands (PheCode 226)	GCST90479853
rs76947600	2.00E-12	ATAD5	prostate-specific antigen (PSA, mean, inv-norm transformed)	GCST90480660
rs3110494	3.00E-12	ABHD15-AS1,SSH2	Brain region volumes	GCST009518
rs3110493	3.00E-12	SSH2,ABHD15-AS1	Brain region volumes	GCST009518
rs764786839	3.00E-12	CRLF3,SUZ12P1	Lymphocyte percentage of white cells	GCST90002389
rs8073217	3.00E-12	SSH2	Red blood cell count	GCST90002363
rs140070219	3.00E-12	TAOK1	Alkaline phosphatase (UKB data field 30610)	GCST90468060
rs11656408	3.00E-12	BLMH,TMIGD1	Urine glycochenodeoxycholate 3-sulfate levels in chronic kidney disease	GCST90265283
rs559972	3.00E-12	TAOK1	Mean platelet volume during the postpartum period	GCST90302233
rs34107657	3.00E-12	NF1	Standing height (UKB data field 50)	GCST90468178
rs3103307	3.00E-12	TMIGD1	Smoking initiation	GCST90243968
rs7214248	3.00E-12	SNORD63,BLMH	OCLN protein levels	GCST90470114
rs3760318	3.00E-12	ADAP2	Diastolic blood pressure	GCST90310295
rs113334146	3.00E-12	TAOK1	Platelet distribution width variance	GCST90565703
rs55920122	3.00E-12	NF1	Polyunsaturated fatty acid levels	GCST90502134
rs4368212	3.00E-12	EVI2B,NF1	platelet count (minimum, inv-norm transformed)	GCST90480652
rs56336338	3.00E-12	RPL35AP35,NUFIP2	total cholesterol (maximum, inv-norm transformed)	GCST90480714
rs545467829	3.00E-12	CPD	Transient mental disorders due to conditions classified elsewhere (PheCode 291.1)	GCST90480738
rs4291964	4.00E-12	EFCAB5	Cortical surface area	GCST010282
rs56336338	4.00E-12	RPL35AP35,NUFIP2	Monocyte count	GCST90002393
rs731759	4.00E-12	RAB11FIP4	Metabolic syndrome	GCST90444487
rs552761103	4.00E-12	NF1	Plateletcrit	GCST90002400
rs2953016	4.00E-12	NF1	Aspartate aminotransferase levels (UKB data field 30650)	GCST90468063
rs871014	4.00E-12	ABHD15-AS1,CORO6	Subcortical volume (MOSTest)	GCST010702
rs2259857	4.00E-12	SSH2	Cortical surface area (min-P)	GCST010697
rs3760318	4.00E-12	ADAP2	Height	GCST90095033
rs9915622	4.00E-12	TAOK1	Mean platelet volume during third trimester of pregnancy	GCST90302231
rs12103563	4.00E-12	OMG,NF1	Brain morphology (MOSTest)	GCST90239729
rs76947600	4.00E-12	ATAD5	prostate-specific antigen (PSA, minimum, inv-norm transformed)	GCST90480661
rs117662433	5.00E-12	ATAD5	Appendicular lean mass	GCST90000025
rs80168287	5.00E-12	SUZ12P1,CRLF3	Mean corpuscular volume	GCST90018746
rs58841564	5.00E-12	SSH2	Neutrophill count (UKB data field 30140)	GCST90468092
rs75076615	5.00E-12	BLMH	BLMH protein levels	GCST90468447

rs113059482	5.00E-12	ABHD15-AS1,SSH2	Physical function (baseline)	GCST90565837
rs9893528	5.00E-12	MYO18A	Cigarettes smoked per day	GCST90243987
rs36070425	5.00E-12	TAOK1	TIMM10 protein levels	GCST90470867
rs76947600	5.00E-12	ATAD5	prostate-specific antigen (PSA, maximum, inv-norm transformed)	GCST90480659
rs11080107	6.00E-12	ANKRD13B,ABHD15-AS1	Coronary artery disease	GCST90132314
rs516051	6.00E-12	TAOK1,ABHD15	Educational attainment	GCST90105038
rs200309755	6.00E-12	TAOK1	JPT2 protein levels	GCST90469663
rs572949964	6.00E-12	NF1	Systemic lupus erythematosus (PheCode 695.42)	GCST90480453
rs145772295	7.00E-12	TAOK1	Mean platelet thrombocyte volume (UKB data field 30100)	GCST90468087
rs76222783	7.00E-12	CRLF3	Height (baseline)	GCST90565843
rs9898911	7.00E-12	ATAD5	Height (baseline)	GCST90565843
rs12952704	7.00E-12	RPL35AP35,NUFIP2	Mean corpuscular haemoglobin (UKB data field 30050)	GCST90468084
rs8073965	7.00E-12	SLC6A4	Physical function (baseline)	GCST90565837
rs76422200	7.00E-12	SLC6A4,SNORD63	Physical function (baseline)	GCST90565837
rs12943365	8.00E-12	NF1	Type 2 diabetes	GCST90018926
rs9912537	8.00E-12	RAB11FIP4	Hip circumference adjusted for BMI	GCST012227
rs62070652	8.00E-12	ATAD5	Osteoarthritis of the hip or knee	GCST90566799
rs61296342	9.00E-12	NF1	Monocyte percentage of white cells	GCST90002394
rs36070425	9.00E-12	TAOK1	TOMM20 protein levels	GCST90470931
rs373379528	9.00E-12	NUFIP2,RPL35AP35	Sex hormone-binding globulin levels	GCST90239821
rs2530394	1.00E-11	RPL35AP35,NUFIP2	Heel bone mineral density	GCST006979
rs116913103	1.00E-11	CPD	Height	GCST007841
rs56208742	1.00E-11	ABHD15,TAOK1	Low density lipoprotein cholesterol levels	GCST010204
rs11080107	1.00E-11	ANKRD13B,ABHD15-AS1	Coronary artery disease	GCST010866
rs112375390	1.00E-11	ADAP2	Height (baseline)	GCST90565843
rs147640667	1.00E-11	NF1	Height (baseline)	GCST90565843
rs117431025	1.00E-11	GOSR1	Height (baseline)	GCST90565843
rs2525570	1.00E-11	NF1	COMP protein levels	GCST90468827
rs11370020	1.00E-11	SSH2	Neutrophil count	GCST90018968
rs721479	1.00E-11	ABHD15-AS1,ABHD15	Height (baseline)	GCST90565843
rs2020942	1.00E-11	SLC6A4	Arm fat percentage left (UKB data field 23123)	GCST90468157
rs3110098	1.00E-11	BLMH	BLMH protein levels	GCST90468447
rs516051	1.00E-11	TAOK1,ABHD15	CEP20 protein levels	GCST90468712
rs3115094	1.00E-11	GIT1,ABHD15-AS1	Height (standard GWA)	GCST90267284
rs28587908	1.00E-11	MYO18A	Cigarettes smoked per day	GCST90243987
rs1967556	1.00E-11	TAOK1	MAX protein levels	GCST90469867
rs200309755	1.00E-11	TAOK1	PLEKHO1 protein levels	GCST90470256
rs142256814	1.00E-11	RN7SL316P,LRRC37BP1	Multi-trait sex score	GCST90270116
rs9894551	1.00E-11	TAOK1,ABHD15	Random glucose levels	GCST90271557
rs12945042	1.00E-11	SLC6A4,SNORD63	Taurochenodeoxycholic acid 3-sulfate levels	GCST90200242
rs370500262	1.00E-11	SSH2	lymphocyte (absolute count, mean, inv-norm transformed)	GCST90479664
rs543128317	1.00E-11	NF1	Lupus (localized and systemic) (PheCode 695.4)	GCST90480454
rs6354	1.00E-11	SLC6A4	Height	GCST90245848
rs72624990	2.00E-11	ABHD15-AS1,GIT1	Height	GCST90435412
rs2259857	2.00E-11	SSH2	Brain morphology (min-P)	GCST010699
rs138818953	2.00E-11	ABHD15,ABHD15-AS1	Height (baseline)	GCST90565843
rs56336338	2.00E-11	RPL35AP35,NUFIP2	Non-HDL cholesterol levels	GCST90239667
rs1038088	2.00E-11	SSH2	Body mass index	GCST90446645
rs6505216	2.00E-11	ATAD5	Ascending thoracic aortic diameter	GCST90094400
rs6505173	2.00E-11	TMIGD1	Taurocholenate sulfate levels	GCST90245448
rs2292973	2.00E-11	ABHD15-AS1,ABHD15	Blood cell traits latent factor 5 (platelet)	GCST90559247
rs11653943	2.00E-11	TMIGD1	Multi-trait sex score	GCST90270118
rs2020942	2.00E-11	SLC6A4	Body mass index	GCST90301650
rs6505216	2.00E-11	ATAD5	neutrophil (fraction, mean, inv-norm transformed)	GCST90479714
rs6505216	2.00E-11	ATAD5	neutrophil (fraction, minimum, inv-norm transformed)	GCST90479715
rs17883623	2.00E-11	NF1	Other specified disorders of plasma protein metabolism (PheCode 270.38)	GCST90479919
rs56336338	2.00E-11	RPL35AP35,NUFIP2	low density lipoprotein cholesterol (LDLC, mean, inv-norm transformed)	GCST90475416
rs1986555	2.00E-11	SSH2,ABHD15-AS1	white blood cell count (WBC, maximum, inv-norm transformed)	GCST90480723
rs56208742	2.00E-11	ABHD15,TAOK1	Total cholesterol levels	GCST90019501
rs4470197	3.00E-11	NSRP1	Brain region volumes	GCST009518
rs3794809	3.00E-11	SLC6A4	PR interval	GCST010320
rs55866125	3.00E-11	EFCAB5	PR interval	GCST010321
rs58018068	3.00E-11	RNU4-34P,RPL35AP35	Red cell distribution width	GCST90002404
rs8081496	3.00E-11	RN7SL316P,LRRC37BP1	Hip circumference adjusted for BMI	GCST90020028
rs62070804	3.00E-11	ABHD15-AS1,ABHD15	Triglyceride levels	GCST90239661
rs147529200	3.00E-11	ABHD15-AS1,SSH2	Testosterone levels	GCST90483487
rs554877749	3.00E-11	NF1	Total testosterone levels	GCST90027095
rs542939	3.00E-11	ABHD15,ABHD15-AS1	Multi-trait sex score	GCST90270118
rs28588622	3.00E-11	ADAP2	red cell diameter width (RDW, minimum, inv-norm transformed)	GCST90480673
rs200678690	4.00E-11	NSRP1	White matter microstructure (fractional anisotropy)	GCST009539
rs2020942	4.00E-11	SLC6A4	Body mass index	GCST007039
rs2020942	4.00E-11	SLC6A4	Body mass index	GCST009871
rs7502556	4.00E-11	NF1	Type 2 diabetes	GCST010118

rs7216246	4.00E-11	TAOK1	Height (baseline)	GCST90565843
rs7225149	4.00E-11	MYO18A	Hip circumference adjusted for BMI	GCST012227
rs78961639	4.00E-11	ATAD5	Body composition (MOSTest)	GCST90267367
rs56881390	4.00E-11	BLMH	Total cholesterol levels	GCST90239676
rs76274570	4.00E-11	TAOK1	Blood cell traits latent factor 5 (platelet)	GCST90559247
rs3110095	4.00E-11	TMIGD1	Glycohyocholate levels	GCST90199935
rs12943365	4.00E-11	NF1	Sex hormone-binding globulin levels	GCST90239822
rs28437451	4.00E-11	SLC6A4,SNORD63	Height	GCST90245848
rs7504112	5.00E-11	SUZ12P1	Red cell distribution width	GCST007074
rs9913225	5.00E-11	CRYBA1,TWF1P1	Type 2 diabetes	GCST010557
rs216466	5.00E-11	ALOX12P1	Highest math class taken (MTAG)	GCST006568
rs147529200	5.00E-11	ABHD15-AS1,SSH2	Testosterone levels	GCST90483498
rs2617866	5.00E-11	SSH2	Body fat percentage (adjusted for testosterone and SHBG)	GCST90432179
rs55920122	5.00E-11	NF1	Linoleic acid levels	GCST90502004
rs9890032	5.00E-11	ATAD5	red blood cell count (RBC, maximum, inv-norm transformed)	GCST90480668
rs8073217	6.00E-11	SSH2	Red blood cell count	GCST90002367
rs2525565	6.00E-11	NF1	Alcohol consumption (drinks per week) (MTAG)	GCST007472
rs117113241	7.00E-11	EFCAB5	Height (baseline)	GCST90565843
rs6505216	7.00E-11	ATAD5	Ascending thoracic aortic diameter	GCST90094402
rs117390397	8.00E-11	SLC6A4	Height (baseline)	GCST90565843
rs11080149	8.00E-11	OMG,NF1	Gamma glutamyl transferase levels	GCST90019507
rs377249930	9.00E-11	CRLF3	Waist circumference adjusted for body mass index	GCST90020029
rs9894551	9.00E-11	TAOK1,ABHD15	Random glucose levels	GCST90271558
rs8073217	1.00E-10	SSH2	Hematocrit	GCST90002308
rs3102560	1.00E-10	ABHD15-AS1,SSH2	Hemoglobin levels	GCST010083
rs3785961	1.00E-10	NUFIP2	Adolescent idiopathic scoliosis	GCST006287
rs9900280	1.00E-10	TAOK1	Mean platelet volume	GCST002184
rs8077048	1.00E-10	RPL35AP35,NUFIP2	Electrocardiogram morphology (amplitude at temporal datapoints)	GCST010796
rs11652593	1.00E-10	EFCAB5	Physical function (baseline)	GCST90565837
rs4795583	1.00E-10	NF1	Physical function (baseline)	GCST90565837
rs35974893	1.00E-10	NF1	Hip circumference adjusted for BMI	GCST90020028
rs8614	1.00E-10	NUFIP2	Smoking initiation (ever regular vs never regular) (MTAG)	GCST007468
rs536130305	1.00E-10	SMURF2P1	Multi-trait sex score	GCST90270118
rs112944916	1.00E-10	CRLF3	Multi-trait sex score	GCST90270118
rs55920122	1.00E-10	NF1	Omega-6 fatty acid levels	GCST90502101
rs141870697	2.00E-10	CRLF3	Lung function (FEV1/FVC)	GCST007080
rs8614	2.00E-10	NUFIP2	Lifetime smoking index	GCST009096
rs8073217	2.00E-10	SSH2	Hematocrit	GCST90002304
rs62070648	2.00E-10	ATAD5	FEV1	GCST007432
rs542939	2.00E-10	ABHD15,ABHD15-AS1	Waist circumference adjusted for body mass index	GCST009867
rs75581564	2.00E-10	PIPOX	Bipolar disorder or major depressive disorder	GCST010416
rs3794730	2.00E-10	SSH2	Metabolic syndrome	GCST90444487
rs7221134	2.00E-10	CRLF3	Height (baseline)	GCST90565843
rs3809790	2.00E-10	ABHD15-AS1,SSH2	Lymphocyte count	GCST90018962
rs8077048	2.00E-10	RPL35AP35,NUFIP2	Electrocardiogram morphology (amplitude at temporal datapoints)	GCST010796
rs8077048	2.00E-10	RPL35AP35,NUFIP2	Electrocardiogram morphology (amplitude at temporal datapoints)	GCST010796
rs3098948	2.00E-10	SSH2,ABHD15-AS1	Precuneus area	GCST90572701
rs8614	2.00E-10	NUFIP2	Externalizing behaviour (multivariate analysis)	GCST90061435
rs11080134	2.00E-10	ATAD5	Hip circumference adjusted for BMI	GCST90020028
rs8614	2.00E-10	NUFIP2	Highest math class taken (MTAG)	GCST006568
rs2940181	2.00E-10	MIR4733HG	Sex hormone-binding globulin levels	GCST90027091
rs201284071	2.00E-10	TAOK1	Blood cell traits latent factor 5 (platelet)	GCST90559247
rs66489046	2.00E-10	TAOK1	Blood cell traits latent factor 5 (platelet)	GCST90559247
rs6505216	2.00E-10	ATAD5	Ascending aorta diameter	GCST90267390
rs34102989	2.00E-10	TAOK1	Blood cell traits latent factor 18 (platelet)	GCST90559260
rs2953016	2.00E-10	NF1	Body mass index	GCST90255621
rs7223535	2.00E-10	ATAD5	Femoral neck width	GCST90399549
rs9915139	2.00E-10	CRLF3,ATAD5	Height	GCST90245846
rs72813627	2.00E-10	NF1	Gamma glutamyl transferase levels	GCST90019507
rs72813607	3.00E-10	NF1	Oily fish consumption	GCST010135
rs72813607	3.00E-10	NF1	Pork consumption	GCST010140
rs11080107	3.00E-10	ANKRD13B,ABHD15-AS1	Coronary artery disease	GCST005195
rs17882020	3.00E-10	NF1,EVI2B	Medication use (drugs used in diabetes)	GCST90018981
rs12943365	3.00E-10	NF1	Estimated glomerular filtration rate (creatinine)	GCST90100220
rs55885610	3.00E-10	CRYBA1	Total testosterone levels	GCST90012114
rs113934718	3.00E-10	ATAD5	Height	GCST90131910
rs72809812	3.00E-10	CPD	Physical function (baseline)	GCST90565837
rs2953013	3.00E-10	NF1	Osteoarthritis of the hip or knee	GCST007092
rs216472	3.00E-10	ALOX12P1	Educational attainment (MTAG)	GCST006571
rs3815156	3.00E-10	NF1	Insomnia	GCST90131901
rs6505216	3.00E-10	ATAD5	Hand grip strength (baseline)	GCST90565845
rs117595964	3.00E-10	CPD	Multi-trait sex score	GCST90270118
rs117870634	3.00E-10	ADAP2	Multi-trait sex score	GCST90270116

rs7223535	4.00E-10	ATAD5	Breast cancer	GCST004988
rs62070804	4.00E-10	ABHD15-AS1,ABHD15	Waist-to-hip ratio adjusted for BMI	GCST008994
rs9904753	4.00E-10	ATAD5	Mean corpuscular volume	GCST90002335
rs12945708	4.00E-10	MIR4733HG	Brain morphology (MOSTest)	GCST010703
rs56853305	4.00E-10	NUFIP2,RPL35AP35	Total testosterone levels	GCST90012113
rs2525570	4.00E-10	NF1	Sex hormone-binding globulin levels	GCST90012107
rs74411146	4.00E-10	CRYBA1,TWF1P1	Appendicular lean mass	GCST90000025
rs2681183	4.00E-10	TAOK1	Blood cell traits latent factor 5 (platelet)	GCST90559247
rs1038088	4.00E-10	SSH2	Body mass index	GCST90301650
rs11080150	5.00E-10	NF1	Total cholesterol levels	GCST009146
rs7220401	5.00E-10	ANKRD13B,ABHD15-AS1	Coronary artery disease	GCST005194
rs138491296	5.00E-10	SSH2,ABHD15-AS1	White blood cell count	GCST004610
rs112871226	5.00E-10	MIR4733HG,RNF135	Height (baseline)	GCST90565843
rs8077048	5.00E-10	RPL35AP35,NUFIP2	Electrocardiogram morphology (amplitude at temporal datapoints)	GCST010796
rs7216631	5.00E-10	NSRP1	Precuneus area	GCST90572701
rs62068618	5.00E-10	SSH2	Height (baseline)	GCST90565843
rs117662433	5.00E-10	ATAD5	Uterine leiomyoma or breast cancer (pleiotropy)	GCST90134420
rs539815106	5.00E-10	TAOK1	Blood cell traits latent factor 18 (platelet)	GCST90559260
rs8080772	6.00E-10	EFCAB5	Brain region volumes	GCST009518
rs2058122	6.00E-10	RPL35AP35,NUFIP2	Apolipoprotein B levels	GCST010243
rs561089333	6.00E-10	CPD	Waist-to-hip ratio adjusted for BMI	GCST009858
rs731758	6.00E-10	RAB11FIP4	Apolipoprotein A1 levels	GCST010241
rs146699004	6.00E-10	TEFM	Breast cancer	GCST90090980
rs13723	6.00E-10	CORO6,ABHD15-AS1	Coronary artery disease	GCST005196
rs12946563	6.00E-10	CRLF3,SUZ12P1	Mean corpuscular hemoglobin	GCST005993
rs12943131	6.00E-10	TAOK1	Fibrosis-4 index in high alcohol intake	GCST90011736
rs137873167	6.00E-10	TWF1P1,CRYBA1	Waist circumference adjusted for body mass index	GCST012226
rs5819889	6.00E-10	SNORD63,SLC6A4	Waist circumference adjusted for body mass index	GCST012226
rs59015798	6.00E-10	NF1	Osteoarthritis (with total hip replacement)	GCST90566802
rs3760318	6.00E-10	ADAP2	Systolic blood pressure	GCST90292477
rs55920122	6.00E-10	NF1	Total fatty acid levels	GCST90502212
rs112178027	7.00E-10	CRYBA1,TWF1P1	Brainstem volume	GCST009697
rs141385558	7.00E-10	TP53I13,ABHD15-AS1,ABHD15	Waist-to-hip ratio adjusted for BMI	GCST009858
rs181952298	7.00E-10	GOSR1,CPD	Height (baseline)	GCST90565843
rs12602834	7.00E-10	NF1,EVI2B	Type 2 diabetes	GCST010555
rs8077048	7.00E-10	RPL35AP35,NUFIP2	Electrocardiogram morphology (amplitude at temporal datapoints)	GCST010796
rs2525570	7.00E-10	NF1	Haemorrhoidal disease	GCST90014033
rs7214248	7.00E-10	SNORD63,BLMH	Total cholesterol levels	GCST006614
rs75581564	7.00E-10	PIPOX	Major depressive disorder	GCST90020227
rs141448761	7.00E-10	OMG,NF1	Multi-trait sex score	GCST90270118
rs2854334	8.00E-10	RAB11FIP4,NF1	Alcohol consumption (drinks per week)	GCST007461
rs8077381,rs1750[illegible]	8.00E-10	PIPOX x LINC02064 - UBL5P1	Total amyloid (SNP x SNP interaction)	GCST010339
rs11653826	9.00E-10	RPL35AP35,NUFIP2	White blood cell count	GCST007070
rs149777351	9.00E-10	RNU4-34P,RPL35AP35	Waist circumference adjusted for body mass index	GCST009867
rs3760318	9.00E-10	ADAP2	Waist circumference adjusted for body mass index	GCST004063
rs2011614	9.00E-10	CPD	HDL cholesterol	GCST90018956
rs59723374	9.00E-10	TAOK1	Fibrosis-4 index in high alcohol intake	GCST90011736
rs11080134	9.00E-10	ATAD5	Diastolic blood pressure	GCST006630
rs56336338	9.00E-10	RPL35AP35,NUFIP2	Non-HDL cholesterol levels	GCST90239670
rs55920122	9.00E-10	NF1	Polyunsaturated fatty acid levels	GCST90502140
rs9900684	9.00E-10	TAOK1	Blood glucose levels	GCST90019508
rs7178240,rs1982[illegible]	9.00E-10	TJP1 x MSI2	Total PHF-tau (SNP x SNP interaction)	GCST010340
rs143119709	1.00E-09	NF1	Height	GCST007841
rs11872020	1.00E-09	SLC6A4	Mean corpuscular hemoglobin	GCST007068
rs3760456	1.00E-09	ABHD15-AS1,CORO6	Brain region volumes	GCST009518
rs75637630	1.00E-09	ATAD5	Granulocyte percentage of myeloid white cells	GCST004608
rs17881327	1.00E-09	NF1	Height (baseline)	GCST90565843
rs138044297	1.00E-09	LRRC37BP1,RN7SL316P	Height (baseline)	GCST90565843
rs12952704	1.00E-09	RPL35AP35,NUFIP2	Serum alkaline phosphatase levels	GCST90018942
rs2525570	1.00E-09	NF1	Sex hormone-binding globulin levels adjusted for BMI	GCST90012106
rs8077048	1.00E-09	RPL35AP35,NUFIP2	Electrocardiogram morphology (amplitude at temporal datapoints)	GCST010796
rs548640283	1.00E-09	SSH2	Height (baseline)	GCST90565843
rs60967688	1.00E-09	EFCAB5,NSRP1	Chronic obstructive pulmonary disease x current smoker interaction (main effect)	GCST90016591
rs5819871	1.00E-09	TAOK1	A body shape index	GCST90020024
rs8081187	1.00E-09	LRRC37BP1,RN7SL316P	Hip circumference adjusted for BMI	GCST012227
rs8065261	1.00E-09	EFCAB5	Resistance to COVID-19 infection (Exposed negative vs positive)	GCST90255358
rs3830317	1.00E-09	BLMH	Glycolithocholate sulfate levels	GCST90245236
rs7214252	1.00E-09	MYO18A	Hand grip strength (baseline)	GCST90565845
rs12945088	1.00E-09	MIR4733HG	Total testosterone levels	GCST90570527
rs56208742	1.00E-09	ABHD15,TAOK1	Apolipoprotein B levels	GCST90019496
rs72813627	1.00E-09	NF1	Sex hormone-binding globulin levels	GCST90019518
rs11080149	1.00E-09	OMG,NF1	Sex hormone-binding globulin levels	GCST90019518
rs62065197	2.00E-09	NUFIP2	Red cell distribution width	GCST007074

rs2020942	2.00E-09	SLC6A4	Predicted visceral adipose tissue	GCST008744
rs146699004	2.00E-09	TEFM	Breast cancer	GCST004988
rs8073217	2.00E-09	SSH2	Hemoglobin	GCST90002384
rs3760318	2.00E-09	ADAP2	Height	GCST000175
rs144843919	2.00E-09	SUZ12P1,RN7SL316P	Birth weight	GCST005146
rs4795538	2.00E-09	NSRP1,SLC6A4	Serum total protein levels	GCST90018976
rs8614	2.00E-09	NUFIP2	Medication use (drugs used in diabetes)	GCST90018981
rs8077048	2.00E-09	RPL35AP35,NUFIP2	Electrocardiogram morphology (amplitude at temporal datapoints)	GCST010796
rs62070652	2.00E-09	ATAD5	Educational attainment	GCST90105038
rs60967688	2.00E-09	EFCAB5,NSRP1	Chronic obstructive pulmonary disease in non-current smokers	GCST90016594
rs1478797900	2.00E-09	SSH2,ABHD15-AS1	Protein quantitative trait loci (liver)	GCST011427
rs35840638	2.00E-09	ADAP2	Uterine leiomyoma or ER negative breast cancer (pleiotropy)	GCST90134422
rs7342938	2.00E-09	ATAD5	Peripheral artery disease or triglycerides (pleiotropy)	GCST90104002
rs7210089	2.00E-09	TAOK1	Blood cell traits latent factor 18 (platelet)	GCST90559260
rs17245607	2.00E-09	RN7SL316P	Alcohol withdrawal factor score	GCST90275372
rs56853305	2.00E-09	NUFIP2,RPL35AP35	Abdominal aortic aneurysm	GCST90432152
rs55920122	2.00E-09	NF1	Linoleic acid levels	GCST90502010
rs11657989	2.00E-09	MIR4733HG	Gamma glutamyl transferase levels	GCST90019507
rs56208742	2.00E-09	ABHD15,TAOK1	Low density lipoprotein cholesterol levels	GCST90019512
rs71689248	3.00E-09	SSH2	Lymphocyte count	GCST004627
rs74815160	3.00E-09	CRLF3,ATAD5	Mean platelet volume	GCST004599
rs79461387	3.00E-09	ATAD5	Neutrophil percentage of white cells	GCST004633
rs568201365	3.00E-09	SSH2	White matter microstructure (fractional anisotropy)	GCST009539
rs8077048	3.00E-09	RPL35AP35,NUFIP2	Electrocardiogram morphology (amplitude at temporal datapoints)	GCST010796
rs183783019	3.00E-09	TAOK1	Red cell distribution width	GCST90002404
rs59723374	3.00E-09	TAOK1	Forns index in high alcohol intake	GCST90011734
rs111919672	3.00E-09	BLMH	Low density lipoprotein cholesterol levels	GCST90239658
rs56336338	3.00E-09	RPL35AP35,NUFIP2	Gout	GCST90455676
rs56387760	3.00E-09	TAOK1	Physical function (baseline)	GCST90565837
rs8076005	3.00E-09	SLC6A4	Insomnia	GCST90131901
rs147287326	3.00E-09	TAOK1	Platelet count	GCST90056183
rs7223535	3.00E-09	ATAD5	Height	GCST90131910
rs35958868	3.00E-09	ADAP2	Height	GCST90131910
rs17767256	3.00E-09	BLMH	Triglyceride levels	GCST90239664
rs28623270	3.00E-09	MIR4733HG	Risk-taking behavior (multivariate analysis)	GCST90239693
rs8067252	3.00E-09	ADAP2	FVC x serum 25-hydroxyvitamin D interaction in ever smokers (2df test)	GCST90590344
rs1017529	3.00E-09	ABHD15-AS1,GIT1	Whole body fat mass (UKB data field 23100)	GCST90428121
rs2040792	3.00E-09	NF1	Serum alkaline phosphatase levels	GCST90019494
rs8073217	4.00E-09	SSH2	Hematocrit	GCST90002383
rs8067440	4.00E-09	NF1	Alcohol consumption (drinks per week) (MTAG)	GCST010543
rs8614	4.00E-09	NUFIP2	Headache	GCST005337
rs7220340	4.00E-09	TWF1P1,CRYBA1	Type 2 diabetes	GCST010555
rs66634575	4.00E-09	MYO18A	Calcium levels	GCST012398
rs8077048	4.00E-09	RPL35AP35,NUFIP2	Electrocardiogram morphology (amplitude at temporal datapoints)	GCST010796
rs12943131	4.00E-09	TAOK1	Forns index in high alcohol intake	GCST90011734
rs5819871	4.00E-09	TAOK1	Waist circumference adjusted for body mass index	GCST90020029
rs115745246	4.00E-09	MIR4733HG,RNF135	Protein quantitative trait loci (liver)	GCST011427
rs11653144	4.00E-09	RNU4-34P,RPL35AP35	Educational attainment (MTAG)	GCST006571
rs74677442	4.00E-09	EFCAB5	Educational attainment (MTAG)	GCST006571
rs11650214	4.00E-09	NF1	Oily fish consumption	GCST90132993
rs113934718	4.00E-09	ATAD5	Tibia length to body height ratio	GCST90270258
rs58713975	5.00E-09	SMURF2P1,KRT17P3	Cardiovascular disease	GCST007072
rs2854322	5.00E-09	NF1	Total cholesterol levels	GCST007134
rs72817635	5.00E-09	MYO18A	Total cholesterol levels	GCST90018974
rs8077048	5.00E-09	RPL35AP35,NUFIP2	Electrocardiogram morphology (amplitude at temporal datapoints)	GCST010796
rs11291804	5.00E-09	BLMH,TMIGD1	Total cholesterol levels	GCST006614
rs12051631	5.00E-09	SSH2	Precuneus area	GCST90572701
rs148378911	5.00E-09	NF1	Physical function (baseline)	GCST90565837
rs7225149	5.00E-09	MYO18A	Waist circumference adjusted for body mass index	GCST012226
rs11080090	5.00E-09	MYO18A	Body mass index (MTAG)	GCST90179150
rs114658410	5.00E-09	EFCAB5	Height (standard GWA)	GCST90267284
rs75581564	5.00E-09	PIPOX	Depression	GCST90239706
rs72815624	5.00E-09	NF1,OMG	Alcohol use disorder (MTAG)	GCST90296428
rs72813627	5.00E-09	NF1	Apolipoprotein B levels	GCST90019496
rs8614	6.00E-09	NUFIP2	Brain region volumes	GCST009518
rs7211246	6.00E-09	NSRP1	QRS complex (12-leadsum)	GCST003872
rs62070652	6.00E-09	ATAD5	Cancer (pleiotropy)	GCST90011822
rs3760456	6.00E-09	ABHD15-AS1,CORO6	Motor premotor area	GCST90572696
rs731759	6.00E-09	RAB11FIP4	Body mass index	GCST90446645
rs62070652	6.00E-09	ATAD5	Breast cancer	GCST90011804
rs2138852	6.00E-09	RNU4-34P,RPL35AP35	Mean platelet volume	GCST90188383
rs3110496	7.00E-09	GIT1,ABHD15-AS1,ANKRD13B	Height	GCST000817
rs8073217	7.00E-09	SSH2	Red blood cell count	GCST90002403

rs8077048	7.00E-09	RPL35AP35,NUFIP2	Electrocardiogram morphology (amplitude at temporal datapoints)	GCST010796
rs28623270	7.00E-09	MIR4733HG	Hip circumference adjusted for BMI	GCST012227
rs17180371	7.00E-09	GOSR1	Human milk oligosaccharide concentration (lacto-N-fucopentaose III) in secretors	GCST90027226
rs9904350	7.00E-09	RNU4-34P,RPL35AP35	Blood cell traits latent factor 18 (platelet)	GCST90559260
rs9890032	8.00E-09	ATAD5	Hip circumference adjusted for BMI	GCST004067
rs62070804	8.00E-09	ABHD15-AS1,ABHD15	Waist-to-hip ratio adjusted for BMI	GCST009858
rs17826255	8.00E-09	MIR4733HG,RNF135	Myopia (pathological)	GCST001712
rs141153629	8.00E-09	RAB11FIP4,NF1	Height (baseline)	GCST90565843
rs1017529	8.00E-09	ABHD15-AS1,GIT1	Adult body size	GCST010988
rs77135925	8.00E-09	MYO18A	autism spectrum disorder, bipolar disorder, major depression, obsessive-compulsive	GCST009600
rs12943783	8.00E-09	MYO18A,TWF1P1	Platelet count	GCST90002402
rs2525570	8.00E-09	NF1	Sex hormone-binding globulin levels	GCST90104297
rs2525570	8.00E-09	NF1	Sex hormone-binding globulin levels	GCST90104273
rs60967688	8.00E-09	EFCAB5,NSRP1	Chronic obstructive pulmonary disease x current smoker interaction (2df)	GCST90016590
rs8069884	8.00E-09	SUZ12P1,RN7SL316P	Neutrophil-to-lymphocyte ratio	GCST90056182
rs216450	8.00E-09	TBC1D29P,ALOX12P1	Body mass index	GCST90301650
rs9903862	8.00E-09	ATAD5	Mitochondrial DNA heteroplasmy (chrM:302:A:AC case-only heteroplasmy)	GCST90268467
rs7225461	9.00E-09	ADAP2	Red blood cell count	GCST007069
rs56397640	9.00E-09	NSRP1	Brain region volumes	GCST009518
rs2002094	9.00E-09	SSH2	Sex hormone-binding globulin levels	GCST90012109
rs11080150	9.00E-09	NF1	Subcortical volume (MOSTest)	GCST010702
rs11653826	9.00E-09	RPL35AP35,NUFIP2	C-reactive protein levels	GCST90029070
rs17727765	9.00E-09	CRYBA1	Depression	GCST005839
rs34977734	9.00E-09	RNU4-34P,RPL35AP35	Blood cell traits latent factor 18 (platelet)	GCST90559260
rs4795574	9.00E-09	NF1	Osteoarthritis (hip)	GCST90566798
rs8081496	9.00E-09	RN7SL316P,LRRC37BP1	Multi-trait sex score	GCST90270116
rs2011614	9.00E-09	CPD	Multi-trait sex score	GCST90270116
rs34638952	1.00E-08	TWF1P1,CRYBA1	Sitting height ratio	GCST002843
rs8080772	1.00E-08	EFCAB5	Chronic obstructive pulmonary disease	GCST007692
rs2729455	1.00E-08	SSH2	Body mass index	GCST90435413
rs78474684	1.00E-08	TWF1P1,MYO18A	Protein quantitative trait loci (liver)	GCST011427
rs8614	1.00E-08	NUFIP2	Self-reported math ability (MTAG)	GCST006569
rs8077048	1.00E-08	RPL35AP35,NUFIP2	Electrocardiogram morphology (amplitude at temporal datapoints)	GCST010796
rs571569087	1.00E-08	SUZ12P1,RN7SL316P	Breast cancer	GCST90651041
rs567563190	1.00E-08	TAOK1	Height (baseline)	GCST90565843
rs113934718	1.00E-08	ATAD5	Breast cancer	GCST90018799
rs8065496	1.00E-08	RAB11FIP4	Estimated glomerular filtration rate (creatinine)	GCST90103633
rs62070804	1.00E-08	ABHD15-AS1,ABHD15	Hip index	GCST90020026
rs78274378	1.00E-08	EFCAB5	Fluid intelligence score (baseline)	GCST90565842
rs2321706	1.00E-08	TMIGD1	Smoking initiation (ever regular vs never regular) (MTAG)	GCST007468
rs1478797900	1.00E-08	SSH2,ABHD15-AS1	Protein quantitative trait loci (liver)	GCST011427
rs8073292	1.00E-08	SSH2	Insomnia	GCST90131901
rs111919672	1.00E-08	BLMH	Low density lipoprotein cholesterol levels	GCST90239655
rs146246379	1.00E-08	SUZ12P1	Waist circumference adjusted for body mass index	GCST012226
rs75581564	1.00E-08	PIPOX	Major depressive disorder (broad)	GCST90328131
rs141167135	1.00E-08	CPD	Height (standard GWA)	GCST90267284
rs62070270	1.00E-08	EFCAB5	Resistance to COVID-19 infection (Exposed negative vs positive)	GCST90255358
rs2854334	1.00E-08	RAB11FIP4,NF1	Total testosterone levels	GCST90503313
rs60450790	1.00E-08	TMIGD1	Gut microbial network clusters (Salmon (at 1 year) x Vaginal Birth interaction	GCST90569412
rs75581564	1.00E-08	PIPOX	Depression	GCST90319327
rs34530466	1.00E-08	TAOK1	Blood cell traits latent factor 18 (platelet)	GCST90559260
rs11080090	1.00E-08	MYO18A	Body mass index	GCST90255621
rs2129785	1.00E-08	BLMH	Multi-trait sex score	GCST90270116
rs72811666	1.00E-08	ADAP2	Body fat percentage (adjusted for testosterone and SHBG)	GCST90432179
rs7213208	1.00E-08	TAOK1	Height	GCST90245848
rs56397640	2.00E-08	NSRP1	Brain region volumes	GCST009518
rs56208742	2.00E-08	ABHD15,TAOK1	LDL cholesterol levels	GCST010245
rs200678690	2.00E-08	NSRP1	White matter microstructure (radial diusivities)	GCST009540
rs8067440	2.00E-08	NF1	Problematic alcohol use (MTAG)	GCST010544
rs62070634	2.00E-08	SUZ12P1	Waist circumference adjusted for body mass index	GCST009867
rs12601994	2.00E-08	MYO18A	Smoking behaviour (cigarette pack-years)	GCST008803
rs3087591	2.00E-08	EVI2B,NF1	Hip circumference	GCST004066
rs3098950	2.00E-08	SSH2,ABHD15-AS1	Body mass index	GCST009871
rs11080090	2.00E-08	MYO18A	Body mass index	GCST009871
rs186786250	2.00E-08	CPD,GOSR1	Height (baseline)	GCST90565843
rs144845777	2.00E-08	RN7SL316P,LRRC37BP1	Height (baseline)	GCST90565843
rs62070638	2.00E-08	CRLF3	Lymphocyte count	GCST90018962
rs3815156	2.00E-08	NF1	Body size at age 10	GCST010989
rs1478797900	2.00E-08	SSH2,ABHD15-AS1	Protein quantitative trait loci (liver)	GCST011427
rs8077048	2.00E-08	RPL35AP35,NUFIP2	Electrocardiogram morphology (amplitude at temporal datapoints)	GCST010796
rs8077048	2.00E-08	RPL35AP35,NUFIP2	Electrocardiogram morphology (amplitude at temporal datapoints)	GCST010796
rs8077048	2.00E-08	RPL35AP35,NUFIP2	Electrocardiogram morphology (amplitude at temporal datapoints)	GCST010796
rs56336338	2.00E-08	RPL35AP35,NUFIP2	Breast cancer or prostate cancer	GCST90308763

rs11080107	2.00E-08	ANKRD13B,ABHD15-AS1	Body mass index	GCST90018727
rs142816786	2.00E-08	SMURF2P1	Lung function (FEV1)	GCST90054783
rs117108035	2.00E-08	TMIGD1,BLMH	Physical function (baseline)	GCST90565837
rs12453269	2.00E-08	RNF135	Physical function (baseline)	GCST90565837
rs3760456	2.00E-08	ABHD15-AS1,CORO6	Bone density (confirmatory factor analysis Factor 19)	GCST90309353
rs201275066	2.00E-08	GOSR1,CPD	Hip circumference adjusted for BMI	GCST012227
rs11080090	2.00E-08	MYO18A	Body mass index	GCST90446645
rs12938581	2.00E-08	MYO18A	Highest math class taken (MTAG)	GCST006568
rs140700	2.00E-08	SLC6A4	Insomnia	GCST90131901
rs559972	2.00E-08	TAOK1	Mean platelet volume	GCST90188383
rs12951287	2.00E-08	NUFIP2,RPL35AP35	Blood cell traits latent factor 5 (platelet)	GCST90559247
rs55920122	2.00E-08	NF1	Polyunsaturated fatty acid levels	GCST90502606
rs28914827	2.00E-08	SLC6A4	Multi-trait sex score	GCST90270118
rs8614	2.00E-08	NUFIP2	Right molecular layer hippocampal volume (body)	GCST90267931
rs3760318	2.00E-08	ADAP2	Diastolic blood pressure	GCST90000059
rs74680845	2.00E-08	RPL35AP35,RNU4-34P	Platelet count	GCST90278640
rs9911167	3.00E-08	ANKRD13B,ABHD15-AS1	White matter microstructure (fractional anisotropy)	GCST009539
rs117870634	3.00E-08	ADAP2	Waist circumference adjusted for body mass index	GCST009867
rs9897794	3.00E-08	EFCAB5	Hemoglobin levels	GCST010083
rs180950758	3.00E-08	RN7SL316P,SUZ12P1	Body mass index	GCST004904
rs17826219	3.00E-08	ATAD5	Body mass index	GCST004904
rs17884466	3.00E-08	NF1	Feeling nervous	GCST006948
rs9902453	3.00E-08	EFCAB5	Coffee consumption (cups per day)	GCST002650
rs547262783	3.00E-08	NF1,EVI2A	Serum alkaline phosphatase levels	GCST90018942
rs111919672	3.00E-08	BLMH	Sex hormone-binding globulin levels	GCST90012111
rs2854313	3.00E-08	NF1	Type 2 diabetes	GCST90013693
rs78274378	3.00E-08	EFCAB5	Cognitive performance (MTAG)	GCST006570
rs6505191	3.00E-08	GOSR1	Cognitive performance (MTAG)	GCST006570
rs62070631	3.00E-08	SUZ12P1	Lung function (FEV1)	GCST006481
rs4427857	3.00E-08	NSRP1	Motor premotor area	GCST90572696
rs1048317	3.00E-08	NF1	Type 2 diabetes	GCST90132185
rs56336338	3.00E-08	RPL35AP35,NUFIP2	Gout	GCST90455677
rs142256814	3.00E-08	RN7SL316P,LRRC37BP1	Hip circumference adjusted for BMI	GCST90020028
rs137873167	3.00E-08	TWF1P1,CRYBA1	Hip circumference adjusted for BMI	GCST012227
rs73267833	3.00E-08	RN7SL316P,LRRC37BP1	Hip circumference adjusted for BMI	GCST012227
rs2953016	3.00E-08	NF1	Insomnia	GCST90131901
rs3815156	3.00E-08	NF1	Insomnia	GCST90131903
rs7504112	3.00E-08	SUZ12P1	Waist circumference adjusted for body mass index	GCST012226
rs2011614	3.00E-08	CPD	High-density lipoprotein levels (MTAG)	GCST90179147
rs8614	3.00E-08	NUFIP2	Alcohol consumption	GCST90133003
rs72624990	3.00E-08	ABHD15-AS1,GIT1	Total diffusion tensor imaging along the perivascular space index	GCST90455546
rs731758	3.00E-08	RAB11FIP4	Multi-trait sex score	GCST90270116
rs56129908	3.00E-08	EFCAB5	Hippocampal subfield left CA4 volume (body)	GCST90267898
rs12450956	3.00E-08	SLC6A4	Intelligence	GCST90264174
rs17767418	3.00E-08	GOSR1	Multi-trait sex score	GCST90270116
rs3815156	3.00E-08	NF1	Multi-trait sex score	GCST90270118
rs8614	3.00E-08	NUFIP2	Pain (pleiotropy)	GCST90104572
rs62070651	3.00E-08	ATAD5	Polyunsaturated fatty acid levels	GCST90502134
rs55920122	3.00E-08	NF1	Saturated fatty acids levels	GCST90502186
rs55920122	3.00E-08	NF1	Omega-6 fatty acid levels	GCST90502502
rs3764419	4.00E-08	ATAD5	Waist circumference adjusted for body mass index	GCST004562
rs140529657	4.00E-08	RAB11FIP4	Heel bone mineral density	GCST007066
rs11080134	4.00E-08	ATAD5	Systolic blood pressure	GCST007087
rs71372253	4.00E-08	MIR4733HG	Type 2 diabetes	GCST009379
rs11653144	4.00E-08	RNU4-34P,RPL35AP35	Mean platelet volume	GCST001439
rs8074306	4.00E-08	KRT17P3,TBC1D29P	Body mass index	GCST009871
rs17669584	4.00E-08	SMURF2P1,KRT17P3	Insomnia symptoms (never/rarely vs. sometimes/usually)	GCST007387
rs113900126	4.00E-08	CRLF3	Height	GCST008163
rs8077048	4.00E-08	RPL35AP35,NUFIP2	Electrocardiogram morphology (amplitude at temporal datapoints)	GCST010796
rs1129506	4.00E-08	EVI2A,NF1	Endometrial cancer	GCST006464
rs116691607	4.00E-08	BLMH	Lewy body pathology	GCST90276159
rs79814671	4.00E-08	NSRP1	Educational attainment	GCST90105038
rs9915567	4.00E-08	CRLF3	Hip circumference adjusted for BMI	GCST90020028
rs192802156	4.00E-08	NF1	Metabolonic lactone sulfate levels	GCST90054779
rs8067252	4.00E-08	ADAP2	Mitochondrial DNA copy number	GCST90026371
rs2905787	4.00E-08	NF1	Total testosterone levels	GCST90027095
rs10512434	4.00E-08	EVI2B,NF1	Type 2 diabetes (PheCode 250.2)	GCST90651113
rs7209032	4.00E-08	RN7SL316P,LRRC37BP1	Multi-trait sex score	GCST90270118
rs3110093	4.00E-08	TMIGD1,Y_RNA	Multi-trait sex score	GCST90270118
rs12453269	4.00E-08	RNF135	Multi-trait sex score	GCST90270116
rs9893169	4.00E-08	EFCAB5	Left hippocampal volume (tail)	GCST90267907
rs55920122	4.00E-08	NF1	Total fatty acid levels	GCST90502218

Supplemental Table 10. Genome-wide significant associations reported in the GWAS catalog within hg19 chr20:58,780,895-59,560,457 (hg38 chr20:60,205,836-60,985,401).

Variant	P Value	Mapped Genes	Reported Trait	Study Accession
rs186999	2.00E-14	MIR646HG	Smoking initiation	GCST90243985
rs6027503	3.00E-14	MIR646HG	Educational attainment	GCST90105038
rs164475	3.00E-14	MIR646HG	Smoking initiation	GCST90243985
rs561754527	7.00E-14	MIR646HG	GLIPR1 protein levels	GCST90469357
rs62198849	6.00E-13	LINC01718,MIR646HG	Bone mineral density mean	GCST90321120
rs62207181	2.00E-12	MIR646HG,LINC01718	Smoking initiation	GCST90243985
rs192966399	2.00E-11	MIR646HG	Arterial embolism and thrombosis of lower extremity artery (PheCode 444.1)	GCST90480211
rs62207181	3.00E-11	MIR646HG,LINC01718	Smoking initiation	GCST90243968
rs6027896	4.00E-11	MIR646HG,LINC01718	Educational attainment	GCST90105038
rs530546566	4.00E-11	MIR646HG	Anomalies of pupillary function (PheCode 379.4)	GCST90480099
rs6027900	5.00E-11	MIR646HG,LINC01718	Gut microbiome abundance (class Tyzzerella sp. 3 (at 3 months) x Any Breastfeeding (3 Months) interaction	GCST90568516
rs6092934	9.00E-11	MIR646HG	Smoking initiation	GCST90243968
rs348838	5.00E-10	MIR646HG	Human milk oligosaccharide concentration (lacto-N-fucopentaose III) in non-secretors	GCST90027228
rs61350676	8.00E-10	MIR646HG	Educational attainment	GCST90105038
rs17801257	2.00E-09	MIR646HG	Educational attainment (MTAG)	GCST006571
rs73297683	2.00E-09	LINC01718,MIR646HG	Protein quantitative trait loci (liver)	GCST011427
rs56363609	3.00E-09	MIR646HG	Human milk oligosaccharide concentration (lacto-N-fucopentaose III) in non-secretors	GCST90027228
rs6065013	5.00E-09	MIR646HG,LINC01718	Gut microbial network clusters (Salmon (at 1 year) x Any Breastfeeding (3 Months) interaction	GCST90569450
rs348809	1.00E-08	MIR646HG	Lifetime smoking index	GCST009096
rs11700264	1.00E-08	MIR646HG	Highest math class taken (MTAG)	GCST006568
rs1018785343	1.00E-08	MIR646HG,LINC01718	Premenopausal menorrhagia (PheCode 627.4)	GCST90651469
rs1201929	2.00E-08	MIR646HG,LINC01718	Highest math class taken	GCST006574
rs6027740	2.00E-08	MIR646HG,LINC01718	Externalizing behaviour (multivariate analysis)	GCST90061435
rs520838	3.00E-08	MIR646HG	Depression	GCST90319327
rs164926	4.00E-08	MIR646HG	Self-reported math ability (MTAG)	GCST006569
rs35384758	4.00E-08	MIR646HG	Haemorrhoidal disease	GCST90014033
rs6015731	4.00E-08	MIR646HG	RBC levels of Pyruvate (uM)	GCST90267519

Supplemental Table 11. Genome-wide significant associations reported in the GWAS catalog within hg19 chr2:234,115,093-234,945,577 (hg38 chr2:233,206,447-234,036,933).

Variant	P Value	Mapped Genes	Reported Trait	Study Accession
rs887829	<1E-304	UGT1A5,UGT1A9,UGT1A10,UGT1A7,UGT1A4,UGT1A8,UGT1A3,UGT1A6	Bilirubin degradation product, C16H18N2O5 (3) levels	GCST90200271
rs6704644	<1E-304	USP40	Total bilirubin levels	GCST90019521
rs2741012	<1E-304	UGT1A12P,UGT1A11P	Disorders of bilirubin excretion (PheCode 277.4)	GCST90475756
rs2741012	<1E-304	UGT1A12P,UGT1A11P	Disorders of bilirubin excretion (PheCode 277.4)	GCST90479948
rs62192955	<1E-304	ATG16L1	Total bilirubin levels	GCST90019521
rs1377460	<1E-304	UGT1A8	Total bilirubin levels	GCST90019521
rs13013882	<1E-304	MROH2A	Total bilirubin levels	GCST90019521
rs114252547	<1E-304	DGKD	Total bilirubin levels	GCST90019521
rs78936963	<1E-304	SAG	Total bilirubin levels	GCST90019521
rs1976391	<1E-304	UGT1A9,UGT1A6,UGT1A5,UGT1A3,UGT1A8,UGT1A10,UGT1A7,UGT1A4	X-11530 levels	GCST90245503
rs6704644	<1E-304	USP40	Direct bilirubin levels	GCST90019505
rs114346341	<1E-304	UGT1A10,UGT1A8	Direct bilirubin levels	GCST90019505
rs13401281	<1E-304	UGT1A7,UGT1A8,UGT1A4,UGT1A9,UGT1A10,UGT1A6,UGT1A5	Total bilirubin levels	GCST005980
rs114346341	<1E-304	UGT1A10,UGT1A8	Total bilirubin levels	GCST90019521
rs2602372	<1E-304	UGT1A8,UGT1A10	Total bilirubin levels	GCST90019521
rs10929302	<1E-304	UGT1A4,UGT1A3,UGT1A10,UGT1A8,UGT1A5,UGT1A6,UGT1A7,UGT1A9	Total bilirubin levels	GCST90019521
rs78936963	<1E-304	SAG	Direct bilirubin levels	GCST90019505
rs887829	<1E-304	UGT1A5,UGT1A9,UGT1A10,UGT1A7,UGT1A4,UGT1A8,UGT1A3,UGT1A6	Bilirubin (E,E) levels	GCST90102890
rs111712352	<1E-304	USP40	Direct bilirubin levels	GCST90019505
rs887829	<1E-304	UGT1A5,UGT1A9,UGT1A10,UGT1A7,UGT1A4,UGT1A8,UGT1A3,UGT1A6	Bilirubin (E,E) levels	GCST90200686
rs13002399	<1E-304	USP40,UGT1A12P	Total bilirubin levels	GCST90019521
rs887829	<1E-304	UGT1A5,UGT1A9,UGT1A10,UGT1A7,UGT1A4,UGT1A8,UGT1A3,UGT1A6	X-16946 levels	GCST90245615
rs887829	<1E-304	UGT1A5,UGT1A9,UGT1A10,UGT1A7,UGT1A4,UGT1A8,UGT1A3,UGT1A6	Bilirubin degradation product, C17H18N2O4 (3) levels	GCST90200703
rs72980341	<1E-304	DGKD	Total bilirubin levels	GCST90019521
rs72976383	<1E-304	SAG	Total bilirubin levels	GCST90019521
rs1042640	<1E-304	UGT1A7,UGT1A5,UGT1A10,UGT1A3,UGT1A9,UGT1A6,UGT1A8,UGT1A4,UGT1A1	Direct bilirubin levels	GCST90019505
rs62192778	<1E-304	USP40	Direct bilirubin levels	GCST90019505
rs111712352	<1E-304	USP40	Total bilirubin levels	GCST90019521
rs17868336	<1E-304	UGT1A3,UGT1A7,UGT1A4,UGT1A8,UGT1A10,UGT1A5,UGT1A6,UGT1A9	Direct bilirubin levels	GCST90019505
rs2602372	<1E-304	UGT1A8,UGT1A10	Direct bilirubin levels	GCST90019505
rs1976391	<1E-304	UGT1A9,UGT1A6,UGT1A5,UGT1A3,UGT1A8,UGT1A10,UGT1A7,UGT1A4	Biliverdin levels	GCST90245128
rs28946889	<1E-304	UGT1A1,UGT1A7,UGT1A9,UGT1A3,UGT1A6,UGT1A10,UGT1A5,UGT1A4,UGT1A8	Total bilirubin levels	GCST005980
rs35754645	<1E-304	UGT1A9,UGT1A8,UGT1A3,UGT1A5,UGT1A6,UGT1A7,UGT1A4,UGT1A10	Total bilirubin levels	GCST90018973
rs2270854	<1E-304	MROH2A	Total bilirubin levels	GCST90019521
rs73998327	<1E-304	ATG16L1	Total bilirubin levels	GCST90019521
rs17864714	<1E-304	MROH2A	Total bilirubin levels	GCST90019521
rs887829	<1E-304	UGT1A5,UGT1A9,UGT1A10,UGT1A7,UGT1A4,UGT1A8,UGT1A3,UGT1A6	Biliverdin levels	GCST90139667
rs17862876	<1E-304	UGT1A7,UGT1A6,UGT1A9,UGT1A8,UGT1A4,UGT1A3,UGT1A5,UGT1A10	Direct bilirubin levels	GCST90019505
rs887829	<1E-304	UGT1A5,UGT1A9,UGT1A10,UGT1A7,UGT1A4,UGT1A8,UGT1A3,UGT1A6	Bilirubin (z,z) levels	GCST90139663
rs4663335	<1E-304	MROH2A	Direct bilirubin levels	GCST90019505
rs1976391	<1E-304	UGT1A9,UGT1A6,UGT1A5,UGT1A3,UGT1A8,UGT1A10,UGT1A7,UGT1A4	X-11522 levels	GCST90245502
rs75520741	<1E-304	UGT1A9,UGT1A5,UGT1A4,UGT1A7,UGT1A6,UGT1A3,UGT1A10,UGT1A8	Total bilirubin levels	GCST005980
rs11683356	<1E-304	UGT1A10,UGT1A8	Direct bilirubin levels	GCST90019505
rs17868336	<1E-304	UGT1A3,UGT1A7,UGT1A4,UGT1A8,UGT1A10,UGT1A5,UGT1A6,UGT1A9	Total bilirubin levels	GCST90019521
rs28946889	<1E-304	UGT1A1,UGT1A7,UGT1A9,UGT1A3,UGT1A6,UGT1A10,UGT1A5,UGT1A4,UGT1A8	Total bilirubin levels	GCST90018753
rs34622615	<1E-304	UGT1A6,UGT1A4,UGT1A10,UGT1A5,UGT1A7,UGT1A8,UGT1A3,UGT1A9,DNAJB3	Total bilirubin levels	GCST90019521
rs1112310	<1E-304	UGT1A10,UGT1A8	Total bilirubin levels	GCST90019521
rs887829	<1E-304	UGT1A5,UGT1A9,UGT1A10,UGT1A7,UGT1A4,UGT1A8,UGT1A3,UGT1A6	Succinimide levels	GCST90245438
rs114099762	<1E-304	USP40	Direct bilirubin levels	GCST90019505
rs11683356	<1E-304	UGT1A10,UGT1A8	Total bilirubin levels	GCST90019521
rs6742078	<1E-304	UGT1A1,UGT1A9,UGT1A4,UGT1A6,UGT1A5,UGT1A10,UGT1A7,UGT1A3,UGT1A8	Bilirubin levels	GCST000386
rs887829	<1E-304	UGT1A5,UGT1A9,UGT1A10,UGT1A7,UGT1A4,UGT1A8,UGT1A3,UGT1A6	Bilirubin (z,z) levels	GCST90102892
rs17862876	<1E-304	UGT1A7,UGT1A6,UGT1A9,UGT1A8,UGT1A4,UGT1A3,UGT1A5,UGT1A10	Total bilirubin levels	GCST90019521
rs1976391	<1E-304	UGT1A9,UGT1A6,UGT1A5,UGT1A3,UGT1A8,UGT1A10,UGT1A7,UGT1A4	Bilirubin (E,E) levels	GCST90245126
rs4663335	<1E-304	MROH2A	Total bilirubin levels	GCST90019521
rs62192778	<1E-304	USP40	Total bilirubin levels	GCST90019521
rs34622615	<1E-304	UGT1A6,UGT1A4,UGT1A10,UGT1A5,UGT1A7,UGT1A8,UGT1A3,UGT1A9,DNAJB3	Direct bilirubin levels	GCST90019505
rs1112310	<1E-304	UGT1A10,UGT1A8	Direct bilirubin levels	GCST90019505
rs1976391	<1E-304	UGT1A9,UGT1A6,UGT1A5,UGT1A3,UGT1A8,UGT1A10,UGT1A7,UGT1A4	Bilirubin (z,z) levels	GCST90245127
rs10929302	<1E-304	UGT1A4,UGT1A3,UGT1A10,UGT1A8,UGT1A5,UGT1A6,UGT1A7,UGT1A9	Direct bilirubin levels	GCST90019505
rs28946889	<1E-304	UGT1A1,UGT1A7,UGT1A9,UGT1A3,UGT1A6,UGT1A10,UGT1A5,UGT1A4,UGT1A8	Total bilirubin levels	GCST90278643
rs10170160	<1E-304	MROH2A	Total bilirubin levels	GCST90019521
rs114099762	<1E-304	USP40	Total bilirubin levels	GCST90019521
rs1042640	<1E-304	UGT1A7,UGT1A5,UGT1A10,UGT1A3,UGT1A9,UGT1A6,UGT1A8,UGT1A4,UGT1A1	Total bilirubin levels	GCST90019521
rs35754645	3.00E-303	UGT1A9,UGT1A8,UGT1A3,UGT1A5,UGT1A6,UGT1A7,UGT1A4,UGT1A10	X-11522 levels	GCST90103249
rs887829	4.00E-301	UGT1A5,UGT1A9,UGT1A10,UGT1A7,UGT1A4,UGT1A8,UGT1A3,UGT1A6	Bilirubin (E,Z or Z,E) levels	GCST90102891
rs62192926	1.00E-300	ATG16L1	Direct bilirubin levels	GCST90134482
rs6431631	1.00E-300	MROH2A	Direct bilirubin levels	GCST90134482
rs28898605	1.00E-300	UGT1A5,UGT1A7,UGT1A9,UGT1A6,UGT1A10,UGT1A8	Total bilirubin levels	GCST90134489
rs6736508	1.00E-300	UGT1A9,UGT1A10,UGT1A7,UGT1A8	Bilirubin levels	GCST90448601
rs4281899	1.00E-300	UGT1A11P,UGT1A8	Total bilirubin levels	GCST90239631
rs7571915	1.00E-300	UGT1A10,UGT1A8,UGT1A9	Total bilirubin levels	GCST90239631
rs887829	1.00E-300	UGT1A5,UGT1A9,UGT1A10,UGT1A7,UGT1A4,UGT1A8,UGT1A3,UGT1A6	X-24849 levels	GCST90103344
rs114252547	3.00E-300	DGKD	Direct bilirubin levels	GCST90019505
rs35754645	3.00E-300	UGT1A9,UGT1A8,UGT1A3,UGT1A5,UGT1A6,UGT1A7,UGT1A4,UGT1A10	X-24849 levels	GCST90103344
rs887829	8.00E-300	UGT1A5,UGT1A9,UGT1A10,UGT1A7,UGT1A4,UGT1A8,UGT1A3,UGT1A6	X-24849 levels	GCST90140622
rs887829	3.00E-298	UGT1A5,UGT1A9,UGT1A10,UGT1A7,UGT1A4,UGT1A8,UGT1A3,UGT1A6	X-11530 levels	GCST90140366
rs35754645	1.00E-295	UGT1A9,UGT1A8,UGT1A3,UGT1A5,UGT1A6,UGT1A7,UGT1A4,UGT1A10	X-21448 levels	GCST90103303
rs887829	3.00E-295	UGT1A5,UGT1A9,UGT1A10,UGT1A7,UGT1A4,UGT1A8,UGT1A3,UGT1A6	Bilirubin (E,E) levels	GCST90139644
rs887829	8.00E-295	UGT1A5,UGT1A9,UGT1A10,UGT1A7,UGT1A4,UGT1A8,UGT1A3,UGT1A6	X-11522 levels	GCST90140365
rs887829	3.00E-294	UGT1A5,UGT1A9,UGT1A10,UGT1A7,UGT1A4,UGT1A8,UGT1A3,UGT1A6	X-16946 levels	GCST90140468
rs887829	3.00E-292	UGT1A5,UGT1A9,UGT1A10,UGT1A7,UGT1A4,UGT1A8,UGT1A3,UGT1A6	Bilirubin degradation product, C16H18N2O5 (1) levels	GCST90200277
rs17863786	2.00E-291	UGT1A7,UGT1A8,UGT1A6,UGT1A9,UGT1A10	Total bilirubin levels (UKB data field 30840)	GCST90468104
rs887829	7.00E-290	UGT1A5,UGT1A9,UGT1A10,UGT1A7,UGT1A4,UGT1A8,UGT1A3,UGT1A6	Bilirubin (E,Z or Z,E) levels	GCST90139645
rs35754645	8.00E-290	UGT1A9,UGT1A8,UGT1A3,UGT1A5,UGT1A6,UGT1A7,UGT1A4,UGT1A10	Metabolite levels (bilirubin (Z,Z); bilirubin; Bilirubin)	GCST90299553
rs35754645	3.00E-289	UGT1A9,UGT1A8,UGT1A3,UGT1A5,UGT1A6,UGT1A7,UGT1A4,UGT1A10	X-11530 levels	GCST90103250
rs13013882	6.00E-288	MROH2A	Direct bilirubin levels	GCST90019505
rs887829	2.00E-287	UGT1A5,UGT1A9,UGT1A10,UGT1A7,UGT1A4,UGT1A8,UGT1A3,UGT1A6	X-21448 levels	GCST90140526
rs887829	3.00E-281	UGT1A5,UGT1A9,UGT1A10,UGT1A7,UGT1A4,UGT1A8,UGT1A3,UGT1A6	Bilirubin degradation product, C16H18N2O5 (2) levels	GCST90200275
rs887829	4.00E-277	UGT1A5,UGT1A9,UGT1A10,UGT1A7,UGT1A4,UGT1A8,UGT1A3,UGT1A6	Bilirubin degradation product, C16H18N2O5 (4) levels	GCST90200265

rs72976383	3.00E-275	SAG	Direct bilirubin levels	GCST90019505
rs35754645	5.00E-268	UGT1A9,UGT1A8,UGT1A3,UGT1A5,UGT1A6,UGT1A7,UGT1A4,UGT1A10	X-16946 levels	GCST90103280
rs887829	2.00E-253	UGT1A5,UGT1A9,UGT1A10,UGT1A7,UGT1A4,UGT1A8,UGT1A3,UGT1A6	X-11442 levels	GCST90245494
rs35754645	2.00E-250	UGT1A9,UGT1A8,UGT1A3,UGT1A5,UGT1A6,UGT1A7,UGT1A4,UGT1A10	Metabolite levels (biliverdin)	GCST90299554
rs79208193	2.00E-239	MROH2A	Total bilirubin levels	GCST90019521
rs62192762	1.00E-238	USP40	Total bilirubin levels	GCST90019521
rs185177860	2.00E-238	USP40	Total bilirubin levels (UKB data field 30840)	GCST90468104
rs1377460	5.00E-236	UGT1A8	Direct bilirubin levels	GCST90019505
rs72974350	8.00E-235	ATG16L1	Total bilirubin levels	GCST90019521
rs13002399	7.00E-233	USP40,UGT1A12P	Direct bilirubin levels	GCST90019505
rs35754645	6.00E-228	UGT1A9,UGT1A8,UGT1A3,UGT1A5,UGT1A6,UGT1A7,UGT1A4,UGT1A10	Succinimide levels	GCST90103200
rs4148325	2.00E-227	UGT1A9,UGT1A7,UGT1A3,UGT1A5,UGT1A8,UGT1A1,UGT1A4,UGT1A10,UGT1A6	Plasma bilirubin (Z,Z) levels in chronic kidney disease	GCST90264863
rs72980341	3.00E-225	DGKD	Direct bilirubin levels	GCST90019505
rs73998327	4.00E-223	ATG16L1	Direct bilirubin levels	GCST90019505
rs62192955	9.00E-223	ATG16L1	Direct bilirubin levels	GCST90019505
rs887829	2.00E-221	UGT1A5,UGT1A9,UGT1A10,UGT1A7,UGT1A4,UGT1A8,UGT1A3,UGT1A6	X-11441 levels	GCST90245493
rs17864714	5.00E-221	MROH2A	Direct bilirubin levels	GCST90019505
rs75130623	2.00E-220	UGT1A5,UGT1A10,UGT1A6,UGT1A7,UGT1A8,RPL17P11,UGT1A9,UGT1A4	Total bilirubin levels (UKB data field 30840)	GCST90468104
rs77900844	2.00E-217	DGKD	Total bilirubin levels	GCST90019521
rs35754645	1.00E-216	UGT1A9,UGT1A8,UGT1A3,UGT1A5,UGT1A6,UGT1A7,UGT1A4,UGT1A10	Metabolite levels (bilirubin (E,E))	GCST90299552
rs17864702	4.00E-208	UGT1A7,UGT1A5,UGT1A10,UGT1A4,UGT1A8,UGT1A9,UGT1A3,UGT1A6	Total bilirubin levels (UKB data field 30840)	GCST90468104
rs4148325	5.00E-204	UGT1A9,UGT1A7,UGT1A3,UGT1A5,UGT1A8,UGT1A1,UGT1A4,UGT1A10,UGT1A6	Plasma bilirubin (E,E)* levels in chronic kidney disease	GCST90264861
rs2270854	8.00E-204	MROH2A	Direct bilirubin levels	GCST90019505
rs10170160	1.00E-200	MROH2A	Direct bilirubin levels	GCST90019505
rs146900547	4.00E-199	MROH2A	Total bilirubin levels (UKB data field 30840)	GCST90468104
rs6742078	4.00E-195	UGT1A1,UGT1A9,UGT1A4,UGT1A6,UGT1A5,UGT1A10,UGT1A7,UGT1A3,UGT1A8	bilirubin degradation product, C17H18N2O4 (2)** levels in chronic kidney	GCST90264869
rs17868337	3.00E-192	UGT1A4,UGT1A10,UGT1A7,UGT1A5,UGT1A9,UGT1A3,UGT1A6,UGT1A8	Total bilirubin levels (UKB data field 30840)	GCST90468104
rs80271431	3.00E-191	SAG	Total bilirubin levels	GCST90019521
rs7593557	3.00E-191	TRPM8	Total bilirubin levels	GCST90019521
rs887829	7.00E-191	UGT1A5,UGT1A9,UGT1A10,UGT1A7,UGT1A4,UGT1A8,UGT1A3,UGT1A6	bilirubin degradation product, C16H18N2O5 (2)** levels in chronic kidney	GCST90264866
rs150329302	4.00E-189	MROH2A	Total bilirubin levels (UKB data field 30840)	GCST90468104
rs185924649	1.00E-188	UGT1A8,UGT1A10	Total bilirubin levels (UKB data field 30840)	GCST90468104
rs887829	3.00E-187	UGT1A5,UGT1A9,UGT1A10,UGT1A7,UGT1A4,UGT1A8,UGT1A3,UGT1A6	bilirubin degradation product, C17H18N2O4 (1)** levels in chronic kidney	GCST90264868
rs35754645	2.00E-185	UGT1A9,UGT1A8,UGT1A3,UGT1A5,UGT1A6,UGT1A7,UGT1A4,UGT1A10	Metabolite levels (bilirubin (E,Z or Z,E))	GCST90299941
rs887829	3.00E-185	UGT1A5,UGT1A9,UGT1A10,UGT1A7,UGT1A4,UGT1A8,UGT1A3,UGT1A6	bilirubin degradation product, C17H18N2O4 (3)** levels in chronic kidney	GCST90264870
rs887829	4.00E-185	UGT1A5,UGT1A9,UGT1A10,UGT1A7,UGT1A4,UGT1A8,UGT1A3,UGT1A6	Plasma biliverdin levels in chronic kidney disease	GCST90264873
rs60697520	9.00E-185	TRPM8,SPP2	Total bilirubin levels (UKB data field 30840)	GCST90468104
rs887829	2.00E-184	UGT1A5,UGT1A9,UGT1A10,UGT1A7,UGT1A4,UGT1A8,UGT1A3,UGT1A6	bilirubin degradation product, C16H18N2O5 (4)** levels in chronic kidney	GCST90264867
rs887829	5.00E-183	UGT1A5,UGT1A9,UGT1A10,UGT1A7,UGT1A4,UGT1A8,UGT1A3,UGT1A6	Plasma bilirubin (E,Z or Z,E)* levels in chronic kidney disease	GCST90264862
rs115604196	3.00E-182	DGKD	Total bilirubin levels	GCST90019521
rs17868337	3.00E-178	UGT1A4,UGT1A10,UGT1A7,UGT1A5,UGT1A9,UGT1A3,UGT1A6,UGT1A8	Total bilirubin levels	GCST90019521
rs115836766	6.00E-178	MROH2A	Total bilirubin levels	GCST90019521
rs887829	4.00E-175	UGT1A5,UGT1A9,UGT1A10,UGT1A7,UGT1A4,UGT1A8,UGT1A3,UGT1A6	Serum metabolite levels	GCST012020
rs12053445	8.00E-174	MROH2A	Total bilirubin levels	GCST90019521
rs887829	3.00E-168	UGT1A5,UGT1A9,UGT1A10,UGT1A7,UGT1A4,UGT1A8,UGT1A3,UGT1A6	Biliverdin levels	GCST90243459
rs149828607	1.00E-162	UGT1A12P,USP40	Total bilirubin levels (UKB data field 30840)	GCST90468104
rs62192762	1.00E-161	USP40	Direct bilirubin levels	GCST90019505
rs1976391	6.00E-159	UGT1A9,UGT1A6,UGT1A5,UGT1A3,UGT1A8,UGT1A10,UGT1A7,UGT1A4	Jaundice (not of newborn) (PheCode 573.5)	GCST90480344
rs6742078	1.00E-158	UGT1A1,UGT1A9,UGT1A4,UGT1A6,UGT1A5,UGT1A10,UGT1A7,UGT1A3,UGT1A8	Bilirubin levels	GCST000730
rs182693186	4.00E-155	UGT1A10,UGT1A8	Total bilirubin levels (UKB data field 30840)	GCST90468104
rs555099891	1.00E-153	UGT1A8	Total bilirubin levels (UKB data field 30840)	GCST90468104
rs887829	2.00E-152	UGT1A5,UGT1A9,UGT1A10,UGT1A7,UGT1A4,UGT1A8,UGT1A3,UGT1A6	Bilirubin (z,z) levels	GCST90243562
rs6431270	2.00E-151	SAG,ATG16L1	Total bilirubin levels	GCST90019521
rs2221198	3.00E-150	UGT1A6,UGT1A4,UGT1A7,UGT1A10,UGT1A9,UGT1A5,UGT1A8,UGT1A3	Serum total bilirubin levels	GCST90104158
rs35754645	5.00E-150	UGT1A9,UGT1A8,UGT1A3,UGT1A5,UGT1A6,UGT1A7,UGT1A4,UGT1A10	Serum metabolite levels	GCST012020
rs187774270	9.00E-145	MROH2A	Total bilirubin levels (UKB data field 30840)	GCST90468104
rs77900844	1.00E-143	DGKD	Direct bilirubin levels	GCST90019505
rs72974350	4.00E-141	ATG16L1	Direct bilirubin levels	GCST90019505
rs79208193	1.00E-140	MROH2A	Direct bilirubin levels	GCST90019505
rs887829	2.00E-140	UGT1A5,UGT1A9,UGT1A10,UGT1A7,UGT1A4,UGT1A8,UGT1A3,UGT1A6	X-11530 levels	GCST90243643
rs4148323	3.00E-139	UGT1A10,UGT1A6,UGT1A9,UGT1A4,UGT1A8,UGT1A1,UGT1A7,UGT1A3,UGT1A5	Bilirubin levels	GCST000730
rs887829	4.00E-139	UGT1A5,UGT1A9,UGT1A10,UGT1A7,UGT1A4,UGT1A8,UGT1A3,UGT1A6	Bilirubin degradation product, C17H20N2O5 (2) levels	GCST90200264
rs34707303	8.00E-138	UGT1A10,UGT1A9,UGT1A8	Total bilirubin levels (UKB data field 30840)	GCST90468104
rs17863818	1.00E-135	MROH2A	Total bilirubin levels	GCST90019521
rs1976391	2.00E-135	UGT1A9,UGT1A6,UGT1A5,UGT1A3,UGT1A8,UGT1A10,UGT1A7,UGT1A4	Jaundice (not of newborn) (PheCode 573.5)	GCST90476093
rs17868337	9.00E-135	UGT1A4,UGT1A10,UGT1A7,UGT1A5,UGT1A9,UGT1A3,UGT1A6,UGT1A8	Direct bilirubin levels	GCST90019505
rs6742078	9.00E-135	UGT1A1,UGT1A9,UGT1A4,UGT1A6,UGT1A5,UGT1A10,UGT1A7,UGT1A3,UGT1A8	Bilirubin degradation product, C17H20N2O5 (1) levels	GCST90200269
rs3755322	2.00E-134	UGT1A5,UGT1A7,UGT1A10,UGT1A6,UGT1A9,UGT1A8	Total bilirubin levels	GCST90019521
rs3755322	4.00E-134	UGT1A5,UGT1A7,UGT1A10,UGT1A6,UGT1A9,UGT1A8	Direct bilirubin levels	GCST90019505
rs887829	4.00E-133	UGT1A5,UGT1A9,UGT1A10,UGT1A7,UGT1A4,UGT1A8,UGT1A3,UGT1A6	bilirubin degradation product, C16H18N2O5 (1)** levels in chronic kidney	GCST90264864
rs887829	4.00E-131	UGT1A5,UGT1A9,UGT1A10,UGT1A7,UGT1A4,UGT1A8,UGT1A3,UGT1A6	X-11442 levels	GCST90140360
rs62191951	5.00E-127	MROH2A	Total bilirubin levels	GCST90019521
rs115836766	1.00E-125	MROH2A	Direct bilirubin levels	GCST90019505
rs887829	5.00E-124	UGT1A5,UGT1A9,UGT1A10,UGT1A7,UGT1A4,UGT1A8,UGT1A3,UGT1A6	X-11441 levels	GCST90140359
rs7593557	8.00E-124	TRPM8	Direct bilirubin levels	GCST90019505
rs114667609	7.00E-123	UGT1A12P	Total bilirubin levels	GCST90019521
rs6717651	3.00E-121	TRPM8	Total bilirubin levels	GCST90019521
rs111741722	2.00E-118	UGT1A3,UGT1A10,UGT1A7,UGT1A8,UGT1A5,UGT1A4,UGT1A9,UGT1A6	Serum metabolite levels	GCST012020
rs115604196	3.00E-118	DGKD	Direct bilirubin levels	GCST90019505
rs80271431	3.00E-117	SAG	Direct bilirubin levels	GCST90019505
rs4148324	5.00E-117	UGT1A3,UGT1A10,UGT1A4,UGT1A6,UGT1A7,UGT1A8,UGT1A9,UGT1A1,UGT1A5	X-11442 levels	GCST90243629
rs58817302	7.00E-117	USP40,DGKD	Total bilirubin levels (UKB data field 30840)	GCST90468104
rs4148324	2.00E-116	UGT1A3,UGT1A10,UGT1A4,UGT1A6,UGT1A7,UGT1A8,UGT1A9,UGT1A1,UGT1A5	X-11441 levels	GCST90243628
rs111741722	4.00E-115	UGT1A3,UGT1A10,UGT1A7,UGT1A8,UGT1A5,UGT1A4,UGT1A9,UGT1A6	Serum metabolite levels	GCST012020
rs887829	3.00E-114	UGT1A5,UGT1A9,UGT1A10,UGT1A7,UGT1A4,UGT1A8,UGT1A3,UGT1A6	X-11793--oxidized bilirubin levels	GCST90243657
rs35283790	1.00E-113	UGT1A3,UGT1A8,UGT1A1,UGT1A6,UGT1A7,UGT1A9,UGT1A4,UGT1A10,UGT1A5	Total bilirubin levels (UKB data field 30840)	GCST90468104
rs78712204	3.00E-113	HJURP	Total bilirubin levels	GCST90019521
rs12053445	3.00E-111	MROH2A	Direct bilirubin levels	GCST90019505
rs35754645	2.00E-110	UGT1A9,UGT1A8,UGT1A3,UGT1A5,UGT1A6,UGT1A7,UGT1A4,UGT1A10	X-11442 levels	GCST90103244
rs62195074	4.00E-109	DGKD	Bone mineral density mean	GCST90321120
rs36075906	2.00E-107	USP40	Total bilirubin levels	GCST90019521
rs12993249	2.00E-105	UGT1A12P,USP40	Bilirubin levels	GCST008817
rs60064064	1.00E-103	DGKD	Bilirubin levels	GCST90448601

rs34983651	2.00E-102	UGT1A6,UGT1A4,UGT1A8,UGT1A5,UGT1A7,UGT1A3,UGT1A9,UGT1A10	Angiopoietin-related protein 1 levels (ANGPTL1.9092.33.3)	GCST90240286
rs10929321	5.00E-102	TRPM8	Total bilirubin levels	GCST90019521
rs35754645	9.00E-102	UGT1A9,UGT1A8,UGT1A3,UGT1A5,UGT1A6,UGT1A7,UGT1A4,UGT1A10	X-11441 levels	GCST90103243
rs11563246	3.00E-100	MROH2A	Total bilirubin levels	GCST90019521
rs887829	8.00E-100	UGT1A5,UGT1A9,UGT1A10,UGT1A7,UGT1A4,UGT1A8,UGT1A3,UGT1A6	Biliverdin levels	GCST90176159
rs887829	1.00E-99	UGT1A5,UGT1A9,UGT1A10,UGT1A7,UGT1A4,UGT1A8,UGT1A3,UGT1A6	Metabolite peak levels (QI14569)	GCST90176905
rs35754645	3.00E-99	UGT1A9,UGT1A8,UGT1A3,UGT1A5,UGT1A6,UGT1A7,UGT1A4,UGT1A10	Metabolite peak levels (QI13939)	GCST90176792
rs4439950	6.00E-99	UGT1A7,UGT1A10,UGT1A6,UGT1A8,UGT1A9	Total bilirubin levels (UKB data field 30840)	GCST90468104
rs12474441	3.00E-98	UGT1A3,UGT1A5,UGT1A6,UGT1A4,UGT1A8,UGT1A10,UGT1A1,UGT1A7,UGT1A9	Total bilirubin levels	GCST90019521
rs138562568	8.00E-98	UGT1A8,UGT1A10	Total bilirubin levels (UKB data field 30840)	GCST90468104
rs114318917	3.00E-97	MSL3B,TRPM8	Total bilirubin levels	GCST90019521
rs6431270	2.00E-95	SAG,ATG16L1	Direct bilirubin levels	GCST90019505
rs887829	4.00E-95	UGT1A5,UGT1A9,UGT1A10,UGT1A7,UGT1A4,UGT1A8,UGT1A3,UGT1A6	Bilirubin (E,E) levels	GCST90243605
rs4148325	2.00E-93	UGT1A9,UGT1A7,UGT1A3,UGT1A5,UGT1A8,UGT1A1,UGT1A4,UGT1A10,UGT1A6	Metabolite peak levels (QI14931)	GCST90176996
rs4148325	5.00E-93	UGT1A9,UGT1A7,UGT1A3,UGT1A5,UGT1A8,UGT1A1,UGT1A4,UGT1A10,UGT1A6	Bilirubin levels in extreme obesity	GCST002628
rs887829	2.00E-92	UGT1A5,UGT1A9,UGT1A10,UGT1A7,UGT1A4,UGT1A8,UGT1A3,UGT1A6	Metabolite peak levels (QI14719)	GCST90176946
rs35754645	8.00E-92	UGT1A9,UGT1A8,UGT1A3,UGT1A5,UGT1A6,UGT1A7,UGT1A4,UGT1A10	UDP-glucuronosyltransferase 1-1 levels	GCST90250096
rs62191951	9.00E-90	MROH2A	Direct bilirubin levels	GCST90019505
rs6742078	1.00E-89	UGT1A1,UGT1A9,UGT1A4,UGT1A6,UGT1A5,UGT1A10,UGT1A7,UGT1A3,UGT1A8	Bilirubin levels	GCST001846
rs35754645	4.00E-89	UGT1A9,UGT1A8,UGT1A3,UGT1A5,UGT1A6,UGT1A7,UGT1A4,UGT1A10	Metabolite peak levels (QI13938)	GCST90176791
rs887829	4.00E-88	UGT1A5,UGT1A9,UGT1A10,UGT1A7,UGT1A4,UGT1A8,UGT1A3,UGT1A6	Metabolite peak levels (QI14798)	GCST90176971
rs887829	9.00E-87	UGT1A5,UGT1A9,UGT1A10,UGT1A7,UGT1A4,UGT1A8,UGT1A3,UGT1A6	Disorders of bilirubin excretion (PheCode 277.4)	GCST90476485
rs6755193	2.00E-85	MROH2A	Total bilirubin levels	GCST90019521
rs17863818	3.00E-84	MROH2A	Direct bilirubin levels	GCST90019505
rs887829	3.00E-84	UGT1A5,UGT1A9,UGT1A10,UGT1A7,UGT1A4,UGT1A8,UGT1A3,UGT1A6	X-21796 levels	GCST90140537
rs838717	4.00E-84	DGKD	Calcium levels	GCST90018951
rs572770153	4.00E-83	MSL3B,TRPM8	Total bilirubin levels (UKB data field 30840)	GCST90468104
rs838718	1.00E-82	DGKD	Calcium levels (UKB data field 30680)	GCST90468065
rs114667609	2.00E-82	UGT1A12P	Direct bilirubin levels	GCST90019505
rs887829	2.00E-82	UGT1A5,UGT1A9,UGT1A10,UGT1A7,UGT1A4,UGT1A8,UGT1A3,UGT1A6	Bilirubin levels	GCST90176037
rs6431630	3.00E-81	UGT1A9,UGT1A4,UGT1A5,UGT1A6,UGT1A10,UGT1A1,UGT1A7,UGT1A8,UGT1A3	Total bilirubin levels	GCST005980
rs6717651	1.00E-80	TRPM8	Direct bilirubin levels	GCST90019505
rs17866593	6.00E-79	SPP2,TRPM8	Total bilirubin levels	GCST90019521
rs6742078	2.00E-78	UGT1A1,UGT1A9,UGT1A4,UGT1A6,UGT1A5,UGT1A10,UGT1A7,UGT1A3,UGT1A8	Bilirubin levels	GCST001846
rs887829	2.00E-78	UGT1A5,UGT1A9,UGT1A10,UGT1A7,UGT1A4,UGT1A8,UGT1A3,UGT1A6	Metabolite peak levels (QI2179)	GCST90177457
rs6738490	4.00E-78	ATG16L1	Crohn's disease	GCST003044
rs35754645	8.00E-78	UGT1A9,UGT1A8,UGT1A3,UGT1A5,UGT1A6,UGT1A7,UGT1A4,UGT1A10	Metabolite peak levels (QI14717)	GCST90176944
rs35754645	9.00E-78	UGT1A9,UGT1A8,UGT1A3,UGT1A5,UGT1A6,UGT1A7,UGT1A4,UGT1A10	Metabolite peak levels (QI422)	GCST90177750
rs13011156	2.00E-77	ATG16L1	Total bilirubin levels	GCST90019521
rs79566441	2.00E-77	HJURP	Total bilirubin levels	GCST90019521
rs112967138	5.00E-77	TRPM8	Total bilirubin levels (UKB data field 30840)	GCST90468104
rs12474441	1.00E-76	UGT1A3,UGT1A5,UGT1A6,UGT1A4,UGT1A8,UGT1A10,UGT1A1,UGT1A7,UGT1A9	Direct bilirubin levels	GCST90019505
rs887829	4.00E-76	UGT1A5,UGT1A9,UGT1A10,UGT1A7,UGT1A4,UGT1A8,UGT1A3,UGT1A6	Metabolite peak levels (QI14769)	GCST90176962
rs887829	4.00E-76	UGT1A5,UGT1A9,UGT1A10,UGT1A7,UGT1A4,UGT1A8,UGT1A3,UGT1A6	Metabolite peak levels (QI14012)	GCST90176813
rs17868345	1.00E-75	MROH2A	Total bilirubin levels (UKB data field 30840)	GCST90468104
rs4148325	3.00E-75	UGT1A9,UGT1A7,UGT1A3,UGT1A5,UGT1A8,UGT1A1,UGT1A4,UGT1A10,UGT1A6	Metabolite peak levels (QI1087)	GCST90176457
rs887829	3.00E-74	UGT1A5,UGT1A9,UGT1A10,UGT1A7,UGT1A4,UGT1A8,UGT1A3,UGT1A6	Metabolic traits	GCST001217
rs887829	3.00E-74	UGT1A5,UGT1A9,UGT1A10,UGT1A7,UGT1A4,UGT1A8,UGT1A3,UGT1A6	Metabolite peak levels (QI1071)	GCST90176442
rs36075906	2.00E-73	USP40	Direct bilirubin levels	GCST90019505
rs35754645	3.00E-73	UGT1A9,UGT1A8,UGT1A3,UGT1A5,UGT1A6,UGT1A7,UGT1A4,UGT1A10	Metabolite peak levels (QI15739)	GCST90177234
rs35754645	3.00E-73	UGT1A9,UGT1A8,UGT1A3,UGT1A5,UGT1A6,UGT1A7,UGT1A4,UGT1A10	Metabolite peak levels (QI1234)	GCST90176622
rs17863775	4.00E-73	UGT1A10,UGT1A8	Total bilirubin levels	GCST90019521
rs887829	7.00E-73	UGT1A5,UGT1A9,UGT1A10,UGT1A7,UGT1A4,UGT1A8,UGT1A3,UGT1A6	Metabolite peak levels (QI14216)	GCST90176843
rs838563	1.00E-72	USP40	Total bilirubin levels	GCST90019521
rs4148325	2.00E-72	UGT1A9,UGT1A7,UGT1A3,UGT1A5,UGT1A8,UGT1A1,UGT1A4,UGT1A10,UGT1A6	Metabolite peak levels (QI14010)	GCST90176812
rs41264153	3.00E-72	UGT1A10,UGT1A8,UGT1A9	Total bilirubin levels	GCST90019521
rs7562378	2.00E-71	TRPM8	Bilirubin levels	GCST90448601
rs75528846	3.00E-70	UGT1A12P,UGT1A11P	Total bilirubin levels	GCST90019521
rs12994997	4.00E-70	ATG16L1	Crohn's disease	GCST001729
rs887829	6.00E-70	UGT1A5,UGT1A9,UGT1A10,UGT1A7,UGT1A4,UGT1A8,UGT1A3,UGT1A6	bilirubin degradation product, C17H20N2O5 (2)** levels in chronic kidney	GCST90264872
rs887829	1.00E-69	UGT1A5,UGT1A9,UGT1A10,UGT1A7,UGT1A4,UGT1A8,UGT1A3,UGT1A6	Bilirubin levels	GCST000388
rs3732218	2.00E-69	UGT1A6,UGT1A7,UGT1A10,UGT1A9,UGT1A5,UGT1A8	X-21441 levels	GCST90245676
rs6742078	3.00E-69	UGT1A1,UGT1A9,UGT1A4,UGT1A6,UGT1A5,UGT1A10,UGT1A7,UGT1A3,UGT1A8	Total bilirubin levels x insomnia interaction	GCST90026657
rs6742078	3.00E-69	UGT1A1,UGT1A9,UGT1A4,UGT1A6,UGT1A5,UGT1A10,UGT1A7,UGT1A3,UGT1A8	Direct bilirubin levels x insomnia interaction	GCST90026656
rs4148323	5.00E-69	UGT1A10,UGT1A6,UGT1A9,UGT1A4,UGT1A8,UGT1A1,UGT1A7,UGT1A3,UGT1A5	Bilirubin levels	GCST001846
rs887829	5.00E-69	UGT1A5,UGT1A9,UGT1A10,UGT1A7,UGT1A4,UGT1A8,UGT1A3,UGT1A6	Total bilirubin levels x insomnia interaction	GCST90026657
rs887829	5.00E-69	UGT1A5,UGT1A9,UGT1A10,UGT1A7,UGT1A4,UGT1A8,UGT1A3,UGT1A6	Direct bilirubin levels x insomnia interaction	GCST90026656
rs78712204	5.00E-69	HJURP	Direct bilirubin levels	GCST90019505
rs6742078	6.00E-69	UGT1A1,UGT1A9,UGT1A4,UGT1A6,UGT1A5,UGT1A10,UGT1A7,UGT1A3,UGT1A8	UDP-glucuronosyltransferase 1-6 levels	GCST90250097
rs34983651	1.00E-67	UGT1A6,UGT1A4,UGT1A8,UGT1A5,UGT1A7,UGT1A3,UGT1A9,UGT1A10	Total bilirubin levels x insomnia interaction	GCST90026657
rs34983651	1.00E-67	UGT1A6,UGT1A4,UGT1A8,UGT1A5,UGT1A7,UGT1A3,UGT1A9,UGT1A10	Direct bilirubin levels x insomnia interaction	GCST90026656
rs35300242	5.00E-67	ATG16L1	nkylosing spondylitis, Crohn's disease, psoriasis, primary sclerosing cholang	GCST005537
rs35754645	6.00E-66	UGT1A9,UGT1A8,UGT1A3,UGT1A5,UGT1A6,UGT1A7,UGT1A4,UGT1A10	Metabolite peak levels (QI1631)	GCST90177367
rs150491876	5.00E-65	DGKD	Total bilirubin levels (UKB data field 30840)	GCST90468104
rs4148325	1.00E-64	UGT1A9,UGT1A7,UGT1A3,UGT1A5,UGT1A8,UGT1A1,UGT1A4,UGT1A10,UGT1A6	Metabolite peak levels (QI14055)	GCST90176821
rs10929302	3.00E-64	UGT1A4,UGT1A3,UGT1A10,UGT1A8,UGT1A5,UGT1A6,UGT1A7,UGT1A9	Bilirubin levels	GCST010048
rs575323861	3.00E-64	USP40,UGT1A12P	Total bilirubin levels (UKB data field 30840)	GCST90468104
rs887829	4.00E-64	UGT1A5,UGT1A9,UGT1A10,UGT1A7,UGT1A4,UGT1A8,UGT1A3,UGT1A6	Disorders of bilirubin excretion (PheCode 277.4)	GCST90476486
rs12692254	2.00E-63	ATG16L1	Crohn's disease	GCST90446792
rs184726344	2.00E-63	UGT1A8	Total bilirubin levels (UKB data field 30840)	GCST90468104
rs11563246	4.00E-63	MROH2A	Direct bilirubin levels	GCST90019505
rs887829	4.00E-63	UGT1A5,UGT1A9,UGT1A10,UGT1A7,UGT1A4,UGT1A8,UGT1A3,UGT1A6	Metabolite peak levels (QI14741)	GCST90176955
rs887829	4.00E-63	UGT1A5,UGT1A9,UGT1A10,UGT1A7,UGT1A4,UGT1A8,UGT1A3,UGT1A6	bilirubin degradation product, C17H20N2O5 (1)** levels in chronic kidney	GCST90264871
rs4148323	2.00E-62	UGT1A10,UGT1A6,UGT1A9,UGT1A4,UGT1A8,UGT1A1,UGT1A7,UGT1A3,UGT1A5	Bilirubin levels	GCST001846
rs562616023	4.00E-62	USP40	Total bilirubin levels (UKB data field 30840)	GCST90468104
rs4148325	5.00E-62	UGT1A9,UGT1A7,UGT1A3,UGT1A5,UGT1A8,UGT1A1,UGT1A4,UGT1A10,UGT1A6	Bilirubin levels	GCST001091
rs6755193	1.00E-61	MROH2A	Direct bilirubin levels	GCST90019505
rs6752107	2.00E-61	ATG16L1	Crohn's disease	GCST004132
rs148540096	3.00E-61	UGT1A8	Total bilirubin levels (UKB data field 30840)	GCST90468104
rs838717	1.00E-60	DGKD	Calcium levels	GCST012398
rs41264153	2.00E-60	UGT1A10,UGT1A8,UGT1A9	Direct bilirubin levels	GCST90019505
rs35754645	3.00E-60	UGT1A9,UGT1A8,UGT1A3,UGT1A5,UGT1A6,UGT1A7,UGT1A4,UGT1A10	Metabolite peak levels (QI14045)	GCST90176819
rs887829	1.00E-59	UGT1A5,UGT1A9,UGT1A10,UGT1A7,UGT1A4,UGT1A8,UGT1A3,UGT1A6	Metabolite peak levels (QI2180)	GCST90177458

rs183044833	1.00E-59	UGT1A8	Total bilirubin levels (UKB data field 30840)	GCST90468104
rs17863787	2.00E-59	UGT1A7,UGT1A6,UGT1A9,UGT1A10,UGT1A8	Total bilirubin levels x insomnia interaction	GCST90026657
rs17863787	2.00E-59	UGT1A7,UGT1A6,UGT1A9,UGT1A10,UGT1A8	Direct bilirubin levels x insomnia interaction	GCST90026656
rs10929321	3.00E-59	TRPM8	Direct bilirubin levels	GCST90019505
rs887829	1.00E-58	UGT1A5,UGT1A9,UGT1A10,UGT1A7,UGT1A4,UGT1A8,UGT1A3,UGT1A6	Cerebrospinal fluid bilirubin (Z,Z) levels	GCST90318171
rs759173	2.00E-58	MROH2A	Total bilirubin levels	GCST90019521
rs28899170	2.00E-58	UGT1A8,UGT1A7,UGT1A10,UGT1A9,UGT1A6	Total bilirubin levels x insomnia interaction	GCST90026657
rs28899170	2.00E-58	UGT1A8,UGT1A7,UGT1A10,UGT1A9,UGT1A6	Direct bilirubin levels x insomnia interaction	GCST90026656
rs6714634	8.00E-58	UGT1A3,UGT1A6,UGT1A4,UGT1A9,UGT1A8,UGT1A7,UGT1A5,UGT1A10	Total bilirubin levels x insomnia interaction	GCST90026657
rs6714634	8.00E-58	UGT1A3,UGT1A6,UGT1A4,UGT1A9,UGT1A8,UGT1A7,UGT1A5,UGT1A10	Direct bilirubin levels x insomnia interaction	GCST90026656
rs10929302	1.00E-57	UGT1A4,UGT1A3,UGT1A10,UGT1A8,UGT1A5,UGT1A6,UGT1A7,UGT1A9	Total bilirubin levels x insomnia interaction	GCST90026657
rs10929302	1.00E-57	UGT1A4,UGT1A3,UGT1A10,UGT1A8,UGT1A5,UGT1A6,UGT1A7,UGT1A9	Direct bilirubin levels x insomnia interaction	GCST90026656
rs3771341	2.00E-57	UGT1A6,UGT1A10,UGT1A4,UGT1A5,UGT1A8,UGT1A1,UGT1A7,UGT1A9,UGT1A3	Total bilirubin levels x insomnia interaction	GCST90026657
rs3771341	2.00E-57	UGT1A6,UGT1A10,UGT1A4,UGT1A5,UGT1A8,UGT1A1,UGT1A7,UGT1A9,UGT1A3	Direct bilirubin levels x insomnia interaction	GCST90026656
rs6431644	2.00E-57	MSL3B	Bilirubin levels	GCST90448601
rs2885296	3.00E-57	UGT1A5,UGT1A3,UGT1A7,UGT1A4,UGT1A10,UGT1A9,UGT1A6,UGT1A8	Total bilirubin levels x insomnia interaction	GCST90026657
rs6747843	3.00E-57	UGT1A8,UGT1A10,UGT1A9,UGT1A3,UGT1A6,UGT1A5,UGT1A7,UGT1A4	Total bilirubin levels x insomnia interaction	GCST90026657
rs2885296	3.00E-57	UGT1A5,UGT1A3,UGT1A7,UGT1A4,UGT1A10,UGT1A9,UGT1A6,UGT1A8	Direct bilirubin levels x insomnia interaction	GCST90026656
rs6747843	3.00E-57	UGT1A8,UGT1A10,UGT1A9,UGT1A3,UGT1A6,UGT1A5,UGT1A7,UGT1A4	Direct bilirubin levels x insomnia interaction	GCST90026656
rs4148325	6.00E-57	UGT1A9,UGT1A7,UGT1A3,UGT1A5,UGT1A8,UGT1A1,UGT1A4,UGT1A10,UGT1A6	Metabolite peak levels (QI1481)	GCST90176973
rs887829	7.00E-57	UGT1A5,UGT1A9,UGT1A10,UGT1A7,UGT1A4,UGT1A8,UGT1A3,UGT1A6	Metabolite peak levels (QI961)	GCST90178592
rs17864701	1.00E-56	UGT1A8,UGT1A3,UGT1A6,UGT1A5,UGT1A9,UGT1A10,UGT1A4,UGT1A7	Total bilirubin levels x insomnia interaction	GCST90026657
rs11695484	1.00E-56	UGT1A5,UGT1A3,UGT1A8,UGT1A4,UGT1A6,UGT1A10,UGT1A9,UGT1A7	Total bilirubin levels x insomnia interaction	GCST90026657
rs17864701	1.00E-56	UGT1A8,UGT1A3,UGT1A6,UGT1A5,UGT1A9,UGT1A10,UGT1A4,UGT1A7	Direct bilirubin levels x insomnia interaction	GCST90026656
rs11695484	1.00E-56	UGT1A5,UGT1A3,UGT1A8,UGT1A4,UGT1A6,UGT1A10,UGT1A9,UGT1A7	Direct bilirubin levels x insomnia interaction	GCST90026656
rs145084767	2.00E-56	UGT1A9,UGT1A10,UGT1A8	Total bilirubin levels	GCST90019521
rs7567468	2.00E-56	UGT1A7,UGT1A6,UGT1A9,UGT1A10,UGT1A3,UGT1A5,UGT1A8,UGT1A4	Total bilirubin levels x insomnia interaction	GCST90026657
rs17862875	2.00E-56	UGT1A3,UGT1A5,UGT1A9,UGT1A4,UGT1A10,UGT1A7,UGT1A8,UGT1A6	Total bilirubin levels x insomnia interaction	GCST90026657
rs34352510	2.00E-56	UGT1A5,UGT1A4,UGT1A10,UGT1A9,UGT1A8,UGT1A7,UGT1A3,UGT1A6	Total bilirubin levels x insomnia interaction	GCST90026657
rs7567468	2.00E-56	UGT1A7,UGT1A6,UGT1A9,UGT1A10,UGT1A3,UGT1A5,UGT1A8,UGT1A4	Direct bilirubin levels x insomnia interaction	GCST90026656
rs17862875	2.00E-56	UGT1A3,UGT1A5,UGT1A9,UGT1A4,UGT1A10,UGT1A7,UGT1A8,UGT1A6	Direct bilirubin levels x insomnia interaction	GCST90026656
rs34352510	2.00E-56	UGT1A5,UGT1A4,UGT1A10,UGT1A9,UGT1A8,UGT1A7,UGT1A3,UGT1A6	Direct bilirubin levels x insomnia interaction	GCST90026656
rs887829	2.00E-56	UGT1A5,UGT1A9,UGT1A10,UGT1A7,UGT1A4,UGT1A8,UGT1A3,UGT1A6	Bilirubin (E,Z or Z,E) levels	GCST90243750
rs1003757	7.00E-56	TRPM8	Total bilirubin levels	GCST90019521
rs114318917	3.00E-55	MSL3B,TRPM8	Direct bilirubin levels	GCST90019505
rs6722076	4.00E-55	UGT1A4,UGT1A10,UGT1A8,UGT1A5,UGT1A3,UGT1A6,UGT1A9,UGT1A7	Total bilirubin levels x insomnia interaction	GCST90026657
rs6722076	4.00E-55	UGT1A4,UGT1A10,UGT1A8,UGT1A5,UGT1A3,UGT1A6,UGT1A9,UGT1A7	Direct bilirubin levels x insomnia interaction	GCST90026656
rs838718	7.00E-55	DGKD	Calcium (mean, inv-norm transformed)	GCST90475176
rs773965339	8.00E-55	UGT1A10,UGT1A9,UGT1A6,UGT1A8,UGT1A7	Total bilirubin levels x insomnia interaction	GCST90026657
rs773965339	8.00E-55	UGT1A10,UGT1A9,UGT1A6,UGT1A8,UGT1A7	Direct bilirubin levels x insomnia interaction	GCST90026656
rs887829	1.00E-54	UGT1A5,UGT1A9,UGT1A10,UGT1A7,UGT1A4,UGT1A8,UGT1A3,UGT1A6	Metabolite peak levels (QI1565)	GCST90177205
rs35754645	3.00E-54	UGT1A9,UGT1A8,UGT1A3,UGT1A5,UGT1A6,UGT1A7,UGT1A4,UGT1A10	Total bilirubin levels	GCST012461
rs543158518	3.00E-54	MROH2A	Total bilirubin levels (UKB data field 30840)	GCST90468104
rs115700531	8.00E-54	USP40	Total bilirubin levels	GCST90019521
rs112132688	1.00E-53	UGT1A7,UGT1A10,UGT1A9,UGT1A4,UGT1A8,UGT1A6,UGT1A5	Total bilirubin levels x insomnia interaction	GCST90026657
rs112132688	1.00E-53	UGT1A7,UGT1A10,UGT1A9,UGT1A4,UGT1A8,UGT1A6,UGT1A5	Direct bilirubin levels x insomnia interaction	GCST90026656
rs79566441	1.00E-53	HJURP	Direct bilirubin levels	GCST90019505
rs4148325	1.00E-53	UGT1A9,UGT1A7,UGT1A3,UGT1A5,UGT1A8,UGT1A1,UGT1A4,UGT1A10,UGT1A6	Metabolite peak levels (QI11242)	GCST90176497
rs887829	1.00E-53	UGT1A5,UGT1A9,UGT1A10,UGT1A7,UGT1A4,UGT1A8,UGT1A3,UGT1A6	Metabolite peak levels (QI1568)	GCST90177213
rs75528846	4.00E-53	UGT1A12P,UGT1A11P	Direct bilirubin levels	GCST90019505
rs184026545	4.00E-53	UGT1A11P,UGT1A12P	Total bilirubin levels (UKB data field 30840)	GCST90468104
rs887829	6.00E-53	UGT1A5,UGT1A9,UGT1A10,UGT1A7,UGT1A4,UGT1A8,UGT1A3,UGT1A6	Metabolite peak levels (QI1027)	GCST90176392
rs887829	8.00E-53	UGT1A5,UGT1A9,UGT1A10,UGT1A7,UGT1A4,UGT1A8,UGT1A3,UGT1A6	Metabolite peak levels (QI2240)	GCST90177473
rs17863775	5.00E-52	UGT1A10,UGT1A8	Direct bilirubin levels	GCST90019505
rs13411202	5.00E-52	TRPM8	Total bilirubin levels (UKB data field 30840)	GCST90468104
rs201829156	1.00E-51	UGT1A6,UGT1A7,UGT1A4,UGT1A9,UGT1A3,UGT1A5,UGT1A8,UGT1A10	X-11880 levels	GCST90245518
rs10166942	9.00E-51	MSL3B,TRPM8	Migraine	GCST90102553
rs887829	1.00E-50	UGT1A5,UGT1A9,UGT1A10,UGT1A7,UGT1A4,UGT1A8,UGT1A3,UGT1A6	Metabolite peak levels (QI1480)	GCST90176972
rs13432541	3.00E-49	ATG16L1	Total bilirubin levels	GCST90019521
rs62192778	4.00E-49	USP40	Bilirubin levels	GCST90448601
rs114748237	4.00E-48	DGKD	Total bilirubin levels	GCST90019521
rs28946889	6.00E-48	UGT1A1,UGT1A7,UGT1A9,UGT1A3,UGT1A6,UGT1A10,UGT1A5,UGT1A4,UGT1A8	Total bilirubin levels	GCST90429160
rs10168416	7.00E-48	UGT1A8,UGT1A7,UGT1A9,UGT1A10	Total bilirubin levels x insomnia interaction	GCST90026657
rs10168416	7.00E-48	UGT1A8,UGT1A7,UGT1A9,UGT1A10	Direct bilirubin levels x insomnia interaction	GCST90026656
rs747484438	8.00E-48	UGT1A10,UGT1A9,UGT1A8,UGT1A7	Total bilirubin levels x insomnia interaction	GCST90026657
rs747484438	8.00E-48	UGT1A10,UGT1A9,UGT1A8,UGT1A7	Direct bilirubin levels x insomnia interaction	GCST90026656
rs2070959	1.00E-47	UGT1A9,UGT1A7,UGT1A10,UGT1A8,UGT1A6	Total bilirubin levels x insomnia interaction	GCST90026657
rs2070959	1.00E-47	UGT1A9,UGT1A7,UGT1A10,UGT1A8,UGT1A6	Direct bilirubin levels x insomnia interaction	GCST90026656
rs7571915	1.00E-47	UGT1A10,UGT1A8,UGT1A9	Total bilirubin levels x insomnia interaction	GCST90026657
rs7571915	1.00E-47	UGT1A10,UGT1A8,UGT1A9	Direct bilirubin levels x insomnia interaction	GCST90026656
rs35754645	1.00E-47	UGT1A9,UGT1A8,UGT1A3,UGT1A5,UGT1A6,UGT1A7,UGT1A4,UGT1A10	Metabolite peak levels (QI4307)	GCST90177765
rs11692664	2.00E-47	UGT1A8,UGT1A9,UGT1A10	Direct bilirubin levels x insomnia interaction	GCST90026656
rs10202865	2.00E-47	UGT1A9,UGT1A10,UGT1A8	Direct bilirubin levels x insomnia interaction	GCST90026656
rs11692664	2.00E-47	UGT1A8,UGT1A9,UGT1A10	Total bilirubin levels x insomnia interaction	GCST90026657
rs10202865	2.00E-47	UGT1A9,UGT1A10,UGT1A8	Total bilirubin levels x insomnia interaction	GCST90026657
rs13011156	3.00E-47	ATG16L1	Direct bilirubin levels	GCST90019505
rs4148325	3.00E-47	UGT1A9,UGT1A7,UGT1A3,UGT1A5,UGT1A8,UGT1A1,UGT1A4,UGT1A10,UGT1A6	Metabolite peak levels (QI1379)	GCST90176765
rs838718	3.00E-47	DGKD	Calcium (maximum, inv-norm transformed)	GCST90475173
rs838718	4.00E-47	DGKD	Calcium (mean, inv-norm transformed)	GCST90479530
rs148968964	5.00E-47	USP40	Total bilirubin levels (UKB data field 30840)	GCST90468104
rs887829	6.00E-47	UGT1A5,UGT1A9,UGT1A10,UGT1A7,UGT1A4,UGT1A8,UGT1A3,UGT1A6	Serum metabolite levels	GCST006249
rs112277298	6.00E-47	UGT1A7,UGT1A6,UGT1A4,UGT1A8,UGT1A10,UGT1A3,UGT1A1,UGT1A9,UGT1A5	Total bilirubin levels (UKB data field 30840)	GCST90468104
rs17863795	8.00E-47	UGT1A5,UGT1A9,UGT1A4,UGT1A7,UGT1A6,UGT1A3,UGT1A10,UGT1A8	Bilirubin (z,z) levels	GCST90200704
rs887829	1.00E-46	UGT1A5,UGT1A9,UGT1A10,UGT1A7,UGT1A4,UGT1A8,UGT1A3,UGT1A6	Serum metabolite levels	GCST006249
rs17866593	5.00E-46	SPP2,TRPM8	Direct bilirubin levels	GCST90019505
rs4148325	1.00E-45	UGT1A9,UGT1A7,UGT1A3,UGT1A5,UGT1A8,UGT1A1,UGT1A4,UGT1A10,UGT1A6	Metabolite peak levels (QI1483)	GCST90176979
rs887829	5.00E-45	UGT1A5,UGT1A9,UGT1A10,UGT1A7,UGT1A4,UGT1A8,UGT1A3,UGT1A6	Metabolite peak levels (QI14032)	GCST90176816
rs192201144	5.00E-45	USP40	Total bilirubin levels (UKB data field 30840)	GCST90468104
rs6758440	5.00E-45	SPP2,TRPM8	Total bilirubin levels (UKB data field 30840)	GCST90468104
rs17868358	7.00E-45	MROH2A	Total bilirubin levels	GCST90019521
rs35984508	1.00E-44	UGT1A9,UGT1A10,UGT1A8	Direct bilirubin levels x insomnia interaction	GCST90026656
rs75444879	1.00E-44	UGT1A10,UGT1A9,UGT1A8	Total bilirubin levels x insomnia interaction	GCST90026657
rs35984508	1.00E-44	UGT1A9,UGT1A10,UGT1A8	Total bilirubin levels x insomnia interaction	GCST90026657

rs75444879	1.00E-44	UGT1A10,UGT1A9,UGT1A8	Direct bilirubin levels x insomnia interaction	GCST90026656
rs838717	1.00E-44	DGKD	Phosphate levels (UKB data field 30810)	GCST90468094
rs1105880	2.00E-44	UGT1A6,UGT1A7,UGT1A9,UGT1A10,UGT1A8	Total bilirubin levels x insomnia interaction	GCST90026657
rs1105880	2.00E-44	UGT1A6,UGT1A7,UGT1A9,UGT1A10,UGT1A8	Direct bilirubin levels x insomnia interaction	GCST90026656
rs1105879	3.00E-44	UGT1A9,UGT1A6,UGT1A7,UGT1A10,UGT1A8	Total bilirubin levels x insomnia interaction	GCST90026657
rs6715829	3.00E-44	UGT1A9,UGT1A8,UGT1A6,UGT1A7,UGT1A10	Total bilirubin levels x insomnia interaction	GCST90026657
rs1105879	3.00E-44	UGT1A9,UGT1A6,UGT1A7,UGT1A10,UGT1A8	Direct bilirubin levels x insomnia interaction	GCST90026656
rs6715829	3.00E-44	UGT1A9,UGT1A8,UGT1A6,UGT1A7,UGT1A10	Direct bilirubin levels x insomnia interaction	GCST90026656
rs77070100	3.00E-44	UGT1A10,UGT1A8,UGT1A9	Total bilirubin levels x insomnia interaction	GCST90026657
rs77070100	3.00E-44	UGT1A10,UGT1A8,UGT1A9	Direct bilirubin levels x insomnia interaction	GCST90026656
rs1003757	4.00E-44	TRPM8	Direct bilirubin levels	GCST90019505
rs34670649	1.00E-43	MROH2A	Total bilirubin levels	GCST90019521
rs17863822	2.00E-43	HJURP	Total bilirubin levels	GCST90019521
rs17863834	3.00E-43	MSL3B,TRPM8	Total bilirubin levels	GCST90019521
rs13015823	3.00E-43	DGKD	Platelet count	GCST90662907
rs35754645	5.00E-43	UGT1A9,UGT1A8,UGT1A3,UGT1A5,UGT1A6,UGT1A7,UGT1A4,UGT1A10	Metabolite levels (succinimide)	GCST90300476
rs142882603	1.00E-42	DGKD	Total bilirubin levels	GCST90019521
rs2302154	2.00E-42	HJURP	Total bilirubin levels	GCST90019521
rs1976391	2.00E-42	UGT1A9,UGT1A6,UGT1A5,UGT1A3,UGT1A8,UGT1A10,UGT1A7,UGT1A4	Tissue factor levels	GCST90249810
rs4148325	4.00E-42	UGT1A9,UGT1A7,UGT1A3,UGT1A5,UGT1A8,UGT1A1,UGT1A4,UGT1A10,UGT1A6	Metabolite peak levels (QI4952)	GCST90177843
rs11891311	1.00E-41	UGT1A6,UGT1A10,UGT1A3,UGT1A5,UGT1A8,UGT1A4,UGT1A9,UGT1A7	Bilirubin levels	GCST001846
rs213549	1.00E-41	TRPM8,MSL3B	Atopic dermatitis (moderate to severe)	GCST90086172
rs34781889	2.00E-41	UGT1A7,UGT1A10,UGT1A8,UGT1A6,UGT1A9	X-23782 levels	GCST90245734
rs12994997	3.00E-41	ATG16L1	Inflammatory bowel disease	GCST003043
rs4148325	3.00E-41	UGT1A9,UGT1A7,UGT1A3,UGT1A5,UGT1A8,UGT1A1,UGT1A4,UGT1A10,UGT1A6	Bilirubin levels in sarilumab-treated rheumatoid arthritis patients	GCST90103787
rs146673916	4.00E-41	USP40	Total bilirubin levels (UKB data field 30840)	GCST90468104
rs4148325	6.00E-41	UGT1A9,UGT1A7,UGT1A3,UGT1A5,UGT1A8,UGT1A1,UGT1A4,UGT1A10,UGT1A6	Cholelithiasis and cholecystitis (PheCode 574)	GCST90476100
rs3792109	7.00E-41	ATG16L1,SCARNA5	Crohn's disease	GCST000879
rs838563	7.00E-41	USP40	Direct bilirubin levels	GCST90019505
rs2012736	1.00E-40	UGT1A9,UGT1A8,UGT1A6,UGT1A7,UGT1A10,UGT1A5	Serum 25-Hydroxyvitamin D levels (conditioned on BMI)	GCST90000616
rs6742078	6.00E-40	UGT1A1,UGT1A9,UGT1A4,UGT1A6,UGT1A5,UGT1A10,UGT1A7,UGT1A3,UGT1A8	Bilirubin levels	GCST001846
rs78338185	7.00E-40	SAG	Total bilirubin levels	GCST90019521
rs838718	9.00E-40	DGKD	Calcium (maximum, inv-norm transformed)	GCST90479529
rs3732220	1.00E-39	UGT1A9,UGT1A7,UGT1A5,UGT1A8,UGT1A6,UGT1A10	Serum 25-Hydroxyvitamin D levels	GCST90000618
rs887829	2.00E-39	UGT1A5,UGT1A9,UGT1A10,UGT1A7,UGT1A4,UGT1A8,UGT1A3,UGT1A6	Metabolite peak levels (QI1459)	GCST90176909
rs10166942	2.00E-39	MSL3B,TRPM8	Migraine	GCST90429186
rs759174	3.00E-39	UGT1A5,UGT1A3,UGT1A6,UGT1A7,UGT1A4,UGT1A10,UGT1A8,UGT1A9	Total bilirubin levels	GCST90134514
rs1057258	4.00E-39	INPP5D	Eosinophill percentage (UKB data field 30210)	GCST90468069
rs1860162	6.00E-39	TRPM8	Total bilirubin levels	GCST90019521
rs2011425	8.00E-39	UGT1A8,UGT1A6,UGT1A10,UGT1A9,UGT1A5,UGT1A4,UGT1A7	X-21441 levels	GCST90200581
rs6742078	2.00E-38	UGT1A1,UGT1A9,UGT1A4,UGT1A6,UGT1A5,UGT1A10,UGT1A7,UGT1A3,UGT1A8	Serum metabolite levels	GCST006249
rs114748237	3.00E-38	DGKD	Direct bilirubin levels	GCST90019505
rs35203651	3.00E-38	UGT1A10,UGT1A3,UGT1A6,UGT1A4,UGT1A7,UGT1A8,UGT1A9,UGT1A5,UGT1A1	UDP-glucuronosyltransferase 1-6 levels	GCST90250097
rs759173	4.00E-38	MROH2A	Direct bilirubin levels	GCST90019505
rs572730173	7.00E-38	TRPM8	Total bilirubin levels (UKB data field 30840)	GCST90468104
rs4616477	1.00E-37	TRPM8,SPP2	Total bilirubin levels	GCST90019521
rs2011425	1.00E-37	UGT1A8,UGT1A6,UGT1A10,UGT1A9,UGT1A5,UGT1A4,UGT1A7	Serum 25-Hydroxyvitamin D levels	GCST010144
rs4148325	2.00E-37	UGT1A9,UGT1A7,UGT1A3,UGT1A5,UGT1A8,UGT1A1,UGT1A4,UGT1A10,UGT1A6	Xanthurenate levels	GCST90245760
rs2278610	3.00E-37	ATG16L1	Bilirubin levels	GCST90448601
rs35203651	4.00E-37	UGT1A10,UGT1A3,UGT1A6,UGT1A4,UGT1A7,UGT1A8,UGT1A9,UGT1A5,UGT1A1	Total cholesterol levels	GCST90239673
rs2119503	5.00E-37	ATG16L1	Bilirubin levels	GCST008817
rs35754645	7.00E-37	UGT1A9,UGT1A8,UGT1A3,UGT1A5,UGT1A6,UGT1A7,UGT1A4,UGT1A10	Membrane-associated progesterone receptor component 1 levels	GCST90248411
rs17862931	9.00E-37	TRPM8	Total bilirubin levels	GCST90019521
rs528273505	1.00E-36	USP40	Total bilirubin levels (UKB data field 30840)	GCST90468104
rs6742078	2.00E-36	UGT1A1,UGT1A9,UGT1A4,UGT1A6,UGT1A5,UGT1A10,UGT1A7,UGT1A3,UGT1A8	Serum levels of protein UGT1A6	GCST90089906
rs2012736	4.00E-36	UGT1A9,UGT1A8,UGT1A6,UGT1A7,UGT1A10,UGT1A5	25-hydroxyvitamin D levels (skin colour stratified)	GCST90448682
rs529116171	4.00E-36	TRPM8,SPP2	Total bilirubin levels (UKB data field 30840)	GCST90468104
rs4148325	5.00E-36	UGT1A9,UGT1A7,UGT1A3,UGT1A5,UGT1A8,UGT1A1,UGT1A4,UGT1A10,UGT1A6	Cholelithiasis (PheCode 574.1)	GCST90476101
rs887829	1.00E-35	UGT1A5,UGT1A9,UGT1A10,UGT1A7,UGT1A4,UGT1A8,UGT1A3,UGT1A6	Metabolite peak levels (QI2236)	GCST90177470
rs72551330	2.00E-35	UGT1A10,UGT1A8,UGT1A9	Indole-3-carboxylic acid levels	GCST90245259
rs1057258	3.00E-35	INPP5D	Eosinophil count	GCST90002298
rs1057258	5.00E-35	INPP5D	Eosinophil count	GCST90002302
rs3771338	6.00E-35	HJURP	Total bilirubin levels	GCST90019521
rs1105880	7.00E-35	UGT1A6,UGT1A7,UGT1A9,UGT1A10,UGT1A8	N-acetyl-4-chlorophenylalanine levels	GCST90140913
rs116241540	8.00E-35	TRPM8	Total bilirubin levels	GCST90019521
rs115700531	2.00E-34	USP40	Direct bilirubin levels	GCST90019505
rs145084767	3.00E-34	UGT1A9,UGT1A10,UGT1A8	Direct bilirubin levels	GCST90019505
rs565454744	3.00E-34	SPP2,TRPM8	Total bilirubin levels (UKB data field 30840)	GCST90468104
rs1057258	5.00E-34	INPP5D	Eosinophil percentage of white cells	GCST90002382
rs1976391	6.00E-34	UGT1A9,UGT1A6,UGT1A5,UGT1A3,UGT1A8,UGT1A10,UGT1A7,UGT1A4	Metabolite peak levels (QI11739)	GCST90176555
rs111741722	8.00E-34	UGT1A3,UGT1A10,UGT1A7,UGT1A8,UGT1A5,UGT1A4,UGT1A9,UGT1A6	Serum metabolite levels	GCST012020
rs78338185	9.00E-34	SAG	Direct bilirubin levels	GCST90019505
rs76384978	9.00E-34	ATG16L1	Total bilirubin levels (UKB data field 30840)	GCST90468104
rs1057258	1.00E-33	INPP5D	Eosinophil count	GCST007065
rs113483143	2.00E-33	DGKD	Total bilirubin levels	GCST90019521
rs144759885	2.00E-33	UGT1A8,UGT1A10	Total bilirubin levels	GCST90019521
rs72972302	3.00E-33	ATG16L1	Total bilirubin levels (UKB data field 30840)	GCST90468104
rs12692254	6.00E-33	ATG16L1	Inflammatory bowel disease	GCST90292538
rs1057258	1.00E-32	INPP5D	eosinophil (fraction, mean, inv-norm transformed)	GCST90475300
rs3828309	2.00E-32	ATG16L1	Crohn's disease	GCST000207
rs13432541	2.00E-32	ATG16L1	Direct bilirubin levels	GCST90019505
rs12185625	3.00E-32	TRPM8	Total bilirubin levels	GCST90019521
rs10166942	4.00E-32	MSL3B,TRPM8	Pain (pleiotropy)	GCST90104572
rs3755322	7.00E-32	UGT1A5,UGT1A7,UGT1A10,UGT1A6,UGT1A9,UGT1A8	Vitamin D levels	GCST90019526
rs1057258	7.00E-32	INPP5D	eosinophil (fraction, mean, inv-norm transformed)	GCST90479605
rs187295904	7.00E-32	UGT1A9,UGT1A10,UGT1A7,UGT1A3,UGT1A6,UGT1A4,UGT1A5,UGT1A8	Total bilirubin levels (UKB data field 30840)	GCST90468104
rs370625278	3.00E-31	ATG16L1	Total bilirubin levels (UKB data field 30840)	GCST90468104
rs838718	4.00E-31	DGKD	Calcium (minimum, inv-norm transformed)	GCST90475179
rs887829	7.00E-31	UGT1A5,UGT1A9,UGT1A10,UGT1A7,UGT1A4,UGT1A8,UGT1A3,UGT1A6	Total bilirubin levels in HIV-1 infection	GCST002745
rs544347953	8.00E-31	TRPM8,MSL3B	Total bilirubin levels (UKB data field 30840)	GCST90468104
rs4148325	1.00E-30	UGT1A9,UGT1A7,UGT1A3,UGT1A5,UGT1A8,UGT1A1,UGT1A4,UGT1A10,UGT1A6	Total bilirubin levels in HIV-1 infection	GCST002745
rs55664157	2.00E-30	DGKD	Plateletcrit	GCST90002400
rs34983651	2.00E-30	UGT1A6,UGT1A4,UGT1A8,UGT1A5,UGT1A7,UGT1A3,UGT1A9,UGT1A10	UDP-glucuronosyltransferase 1-6 levels (UGT1A6.7891.45.3)	GCST90243256

rs13035837	4.00E-30	DGKD	platelet count (maximum, inv-norm transformed)	GCST90476296
rs1441091	4.00E-30	ATG16L1	Total bilirubin levels (UKB data field 30840)	GCST90468104
rs527978237	4.00E-30	DGKD	Total bilirubin levels (UKB data field 30840)	GCST90468104
rs76895192	5.00E-30	MROH2A	Total bilirubin levels	GCST90019521
rs6742078	6.00E-30	UGT1A1,UGT1A9,UGT1A4,UGT1A6,UGT1A5,UGT1A10,UGT1A7,UGT1A3,UGT1A8	Serum metabolite levels	GCST006249
rs4148323	7.00E-30	UGT1A10,UGT1A6,UGT1A9,UGT1A4,UGT1A8,UGT1A1,UGT1A7,UGT1A3,UGT1A5	Bilirubin levels	GCST001846
rs6431270	8.00E-30	SAG,ATG16L1	Calcium levels	GCST90019500
rs182385495	4.00E-29	UGT1A6,UGT1A8,UGT1A7,UGT1A9,UGT1A10	Total bilirubin levels (UKB data field 30840)	GCST90468104
rs1976391	5.00E-29	UGT1A9,UGT1A6,UGT1A5,UGT1A3,UGT1A8,UGT1A10,UGT1A7,UGT1A4	X-21796 levels	GCST90245695
rs7564805	6.00E-29	SAG	Photoreceptor cell layer thickness phenotypes (MTAG)	GCST90255614
rs13015823	8.00E-29	DGKD	Platelet count	GCST90002361
rs17863840	9.00E-29	TRPM8	Total bilirubin levels	GCST90019521
rs28946889	1.00E-28	UGT1A1,UGT1A7,UGT1A9,UGT1A3,UGT1A6,UGT1A10,UGT1A5,UGT1A4,UGT1A8	Direct bilirubin levels	GCST90429161
rs62192317	1.00E-28	TRPM8,MSL3B	Total bilirubin levels (UKB data field 30840)	GCST90468104
rs17864784	2.00E-28	SPP2,TRPM8	Total bilirubin levels	GCST90019521
rs1057258	2.00E-28	INPP5D	eosinophil (fraction, maximum, inv-norm transformed)	GCST90479604
rs6752107	3.00E-28	ATG16L1	Inflammatory bowel disease	GCST004131
rs34670649	3.00E-28	MROH2A	Direct bilirubin levels	GCST90019505
rs13015823	3.00E-28	DGKD	Platelet count	GCST90002357
rs17863822	4.00E-28	HJURP	Direct bilirubin levels	GCST90019505
rs6742078	4.00E-28	UGT1A1,UGT1A9,UGT1A4,UGT1A6,UGT1A5,UGT1A10,UGT1A7,UGT1A3,UGT1A8	Total bilirubin levels	GCST90429160
rs4148324	5.00E-28	UGT1A3,UGT1A10,UGT1A4,UGT1A6,UGT1A7,UGT1A8,UGT1A9,UGT1A1,UGT1A5	X-21796 levels	GCST90200580
rs887829	6.00E-28	UGT1A5,UGT1A9,UGT1A10,UGT1A7,UGT1A4,UGT1A8,UGT1A3,UGT1A6	Plasma X-21796 levels in chronic kidney disease	GCST90266519
rs183484892	6.00E-28	UGT1A9,UGT1A4,UGT1A10,UGT1A6,UGT1A3,UGT1A5,UGT1A7,UGT1A8	Total bilirubin levels (UKB data field 30840)	GCST90468104
rs35203651	8.00E-28	UGT1A10,UGT1A3,UGT1A6,UGT1A4,UGT1A7,UGT1A8,UGT1A9,UGT1A5,UGT1A1	Non-HDL cholesterol levels	GCST90239667
rs28900987	8.00E-28	MSL3B,TRPM8	Total bilirubin levels (UKB data field 30840)	GCST90468104
rs72972302	1.00E-27	ATG16L1	Total bilirubin levels	GCST90019521
rs838716	1.00E-27	DGKD	Calcium (minimum, inv-norm transformed)	GCST90479531
rs142882603	2.00E-27	DGKD	Direct bilirubin levels	GCST90019505
rs72984479	2.00E-27	UGT1A8,UGT1A10	Total bilirubin levels (UKB data field 30840)	GCST90468104
rs141230614	2.00E-27	UGT1A8,UGT1A9,UGT1A10	Total bilirubin levels (UKB data field 30840)	GCST90468104
rs6431630	2.00E-27	UGT1A9,UGT1A4,UGT1A5,UGT1A6,UGT1A10,UGT1A1,UGT1A7,UGT1A8,UGT1A3	UDP-glucuronosyltransferase 1-6 levels (UGT1A6.7891.45.3)	GCST90243256
rs2741045	3.00E-27	UGT1A10,UGT1A8	total cholesterol (maximum, inv-norm transformed)	GCST90476420
rs887829	4.00E-27	UGT1A5,UGT1A9,UGT1A10,UGT1A7,UGT1A4,UGT1A8,UGT1A3,UGT1A6	Total cholesterol levels	GCST90239676
rs887829	7.00E-27	UGT1A5,UGT1A9,UGT1A10,UGT1A7,UGT1A4,UGT1A8,UGT1A3,UGT1A6	Hyperbilirubinemia in acute lymphoblastic leukemia	GCST90245885
rs10210101	9.00E-27	ATG16L1	Total bilirubin levels (UKB data field 30840)	GCST90468104
rs35754645	1.00E-26	UGT1A9,UGT1A8,UGT1A3,UGT1A5,UGT1A6,UGT1A7,UGT1A4,UGT1A10	Metabolite levels (xanthurenate; cmh_xanthurenate; Xanthurenate)	GCST90300270
rs887829	1.00E-26	UGT1A5,UGT1A9,UGT1A10,UGT1A7,UGT1A4,UGT1A8,UGT1A3,UGT1A6	BCHE protein levels	GCST90468429
rs4148328	2.00E-26	UGT1A6,UGT1A9,UGT1A5,UGT1A3,UGT1A4,UGT1A7,UGT1A1,UGT1A10,UGT1A8	Metabolite levels (bilirubin (Z,Z); bilirubin; Bilirubin)	GCST90299553
rs887829	3.00E-26	UGT1A5,UGT1A9,UGT1A10,UGT1A7,UGT1A4,UGT1A8,UGT1A3,UGT1A6	Serum metabolite levels	GCST006249
rs2011425	3.00E-26	UGT1A8,UGT1A6,UGT1A10,UGT1A9,UGT1A5,UGT1A4,UGT1A7	Total testosterone levels	GCST90012114
rs34231016	3.00E-26	DGKD	Platelet crit (UKB data field 30090)	GCST90468096
rs76384978	4.00E-26	ATG16L1	Total bilirubin levels	GCST90019521
rs117809958	4.00E-26	ATG16L1	Type 2 diabetes	GCST90492734
rs35203651	4.00E-26	UGT1A10,UGT1A3,UGT1A6,UGT1A4,UGT1A7,UGT1A8,UGT1A9,UGT1A5,UGT1A1	Low density lipoprotein cholesterol levels	GCST90239655
rs2302154	5.00E-26	HJURP	Direct bilirubin levels	GCST90019505
rs11673726	9.00E-26	UGT1A9,UGT1A8,UGT1A6,UGT1A10,UGT1A3,UGT1A5,UGT1A7,UGT1A4	Total bilirubin levels x insomnia interaction	GCST90026657
rs11673726	9.00E-26	UGT1A9,UGT1A8,UGT1A6,UGT1A10,UGT1A3,UGT1A5,UGT1A7,UGT1A4	Direct bilirubin levels x insomnia interaction	GCST90026656
rs6742078	1.00E-25	UGT1A1,UGT1A9,UGT1A4,UGT1A6,UGT1A5,UGT1A10,UGT1A7,UGT1A3,UGT1A8	Serum metabolite levels	GCST006249
rs13410335	1.00E-25	UGT1A4,UGT1A5,UGT1A10,UGT1A8,UGT1A7,UGT1A9,UGT1A6,UGT1A3	Total bilirubin levels x insomnia interaction	GCST90026657
rs7564935	1.00E-25	UGT1A3,UGT1A10,UGT1A5,UGT1A4,UGT1A7,UGT1A8,UGT1A9,UGT1A6	Total bilirubin levels x insomnia interaction	GCST90026657
rs11888459	1.00E-25	UGT1A9,UGT1A6,UGT1A2P,UGT1A10,UGT1A3,UGT1A8,UGT1A5,UGT1A4,UGT1A7	Total bilirubin levels x insomnia interaction	GCST90026657
rs10178992	1.00E-25	UGT1A6,UGT1A4,UGT1A10,UGT1A7,UGT1A3,UGT1A5,UGT1A9,UGT1A8	Total bilirubin levels x insomnia interaction	GCST90026657
rs7604115	1.00E-25	UGT1A10,UGT1A8,UGT1A7,UGT1A6,UGT1A5,UGT1A9,UGT1A4,UGT1A3	Total bilirubin levels x insomnia interaction	GCST90026657
rs13410335	1.00E-25	UGT1A4,UGT1A5,UGT1A10,UGT1A8,UGT1A7,UGT1A9,UGT1A6,UGT1A3	Direct bilirubin levels x insomnia interaction	GCST90026656
rs7564935	1.00E-25	UGT1A3,UGT1A10,UGT1A5,UGT1A4,UGT1A7,UGT1A8,UGT1A9,UGT1A6	Direct bilirubin levels x insomnia interaction	GCST90026656
rs11888459	1.00E-25	UGT1A9,UGT1A6,UGT1A2P,UGT1A10,UGT1A3,UGT1A8,UGT1A5,UGT1A4,UGT1A7	Direct bilirubin levels x insomnia interaction	GCST90026656
rs10178992	1.00E-25	UGT1A6,UGT1A4,UGT1A10,UGT1A7,UGT1A3,UGT1A5,UGT1A9,UGT1A8	Direct bilirubin levels x insomnia interaction	GCST90026656
rs7604115	1.00E-25	UGT1A10,UGT1A8,UGT1A7,UGT1A6,UGT1A5,UGT1A9,UGT1A4,UGT1A3	Direct bilirubin levels x insomnia interaction	GCST90026656
rs187729049	1.00E-25	USP40	Total bilirubin levels (UKB data field 30840)	GCST90468104
rs76895192	2.00E-25	MROH2A	Direct bilirubin levels	GCST90019505
rs1105880	2.00E-25	UGT1A6,UGT1A7,UGT1A9,UGT1A10,UGT1A8	Xanthurenate levels	GCST90139390
rs72551330	4.00E-25	UGT1A10,UGT1A8,UGT1A9	X-02249 levels	GCST90245479
rs887829	5.00E-25	UGT1A5,UGT1A9,UGT1A10,UGT1A7,UGT1A4,UGT1A8,UGT1A3,UGT1A6	Cholelithiasis-related traits in sickle cell anemia	GCST001497
rs116321528	5.00E-25	USP40	Total bilirubin levels (UKB data field 30840)	GCST90468104
rs4148325	6.00E-25	UGT1A9,UGT1A7,UGT1A3,UGT1A5,UGT1A8,UGT1A1,UGT1A4,UGT1A10,UGT1A6	Metabolite peak levels (QI15805)	GCST90177273
rs28900384	6.00E-25	UGT1A7,UGT1A9,UGT1A8,UGT1A3,UGT1A10,UGT1A5,UGT1A4,UGT1A6	X-24849 levels	GCST90103344
rs13401281	7.00E-25	UGT1A7,UGT1A8,UGT1A4,UGT1A9,UGT1A10,UGT1A6,UGT1A5	Total bilirubin levels x insomnia interaction	GCST90026657
rs13401281	7.00E-25	UGT1A7,UGT1A8,UGT1A4,UGT1A9,UGT1A10,UGT1A6,UGT1A5	Direct bilirubin levels x insomnia interaction	GCST90026656
rs887829	9.00E-25	UGT1A5,UGT1A9,UGT1A10,UGT1A7,UGT1A4,UGT1A8,UGT1A3,UGT1A6	Metabolite levels	GCST001882
rs2011425	9.00E-25	UGT1A8,UGT1A6,UGT1A10,UGT1A9,UGT1A5,UGT1A4,UGT1A7	Bioavailable testosterone levels	GCST90012104
rs2011425	9.00E-25	UGT1A8,UGT1A6,UGT1A10,UGT1A9,UGT1A5,UGT1A4,UGT1A7	X-11440 levels	GCST90245492
rs2362290	1.00E-24	TRPM8,MSL3B	Headache or migraine	GCST90267554
rs4148324	1.00E-24	UGT1A3,UGT1A10,UGT1A4,UGT1A6,UGT1A7,UGT1A8,UGT1A9,UGT1A1,UGT1A5	Thyroxine levels	GCST90245458
rs1057258	2.00E-24	INPP5D	Eosinophil count	GCST90002381
rs929596	3.00E-24	UGT1A3,UGT1A5,UGT1A4,UGT1A6,UGT1A9,UGT1A8,UGT1A10,UGT1A1,UGT1A7	Total bilirubin levels in HIV-1 infection	GCST002745
rs887829	3.00E-24	UGT1A5,UGT1A9,UGT1A10,UGT1A7,UGT1A4,UGT1A8,UGT1A3,UGT1A6	Serum metabolite levels	GCST006249
rs1983023	3.00E-24	UGT1A4,UGT1A9,UGT1A10,UGT1A6,UGT1A7,UGT1A8,UGT1A5	Direct bilirubin levels	GCST90429161
rs17868341	3.00E-24	UGT1A6,UGT1A8,UGT1A5,UGT1A10,UGT1A1,UGT1A3,UGT1A7,UGT1A9,UGT1A4	P-cresol-glucuronide levels	GCST90245367
rs149328116	3.00E-24	TRPM8	Total bilirubin levels (UKB data field 30840)	GCST90468104
rs116241540	4.00E-24	TRPM8	Direct bilirubin levels	GCST90019505
rs887829	6.00E-24	UGT1A5,UGT1A9,UGT1A10,UGT1A7,UGT1A4,UGT1A8,UGT1A3,UGT1A6	Abnormal results of function study of liver (PheCode 573.7)	GCST90476097
rs11690786	7.00E-24	MROH2A	Total bilirubin levels x insomnia interaction	GCST90026657
rs11690786	7.00E-24	MROH2A	Direct bilirubin levels x insomnia interaction	GCST90026656
rs1860162	8.00E-24	TRPM8	Direct bilirubin levels	GCST90019505
rs10166942	1.00E-23	MSL3B,TRPM8	Migraine	GCST003720
rs6722064	1.00E-23	MROH2A	Total bilirubin levels x insomnia interaction	GCST90026657
rs6722064	1.00E-23	MROH2A	Direct bilirubin levels x insomnia interaction	GCST90026656
rs1057258	1.00E-23	INPP5D	eosinophil (fraction, maximum, inv-norm transformed)	GCST90475297
rs6742078	2.00E-23	UGT1A1,UGT1A9,UGT1A4,UGT1A6,UGT1A5,UGT1A10,UGT1A7,UGT1A3,UGT1A8	Circulating cell-free DNA	GCST001478
rs17868358	2.00E-23	MROH2A	Direct bilirubin levels	GCST90019505
rs11563251	2.00E-23	UGT1A10,UGT1A8,UGT1A3,UGT1A6,UGT1A5,UGT1A4,UGT1A9,UGT1A7,UGT1A1	Serum levels of protein UGT1A6	GCST90089906
rs4148325	2.00E-23	UGT1A9,UGT1A7,UGT1A3,UGT1A5,UGT1A8,UGT1A1,UGT1A4,UGT1A10,UGT1A6	Xanthurenate levels	GCST90103347

rs148755655	2.00E-23	UGT1A1,UGT1A8,UGT1A10,UGT1A9,UGT1A6,UGT1A7,UGT1A4,UGT1A5,UGT1A3	Total bilirubin levels (UKB data field 30840)	GCST90468104
rs12185625	3.00E-23	TRPM8	Direct bilirubin levels	GCST90019505
rs17864784	3.00E-23	SPP2,TRPM8	Direct bilirubin levels	GCST90019505
rs4148325	3.00E-23	UGT1A9,UGT1A7,UGT1A3,UGT1A5,UGT1A8,UGT1A1,UGT1A4,UGT1A10,UGT1A6	Serum levels of protein F3	GCST90088822
rs72551330	4.00E-23	UGT1A10,UGT1A8,UGT1A9	X-18901 levels	GCST90245652
rs838717	6.00E-23	DGKD	Kidney stone disease	GCST90652506
rs2361502	7.00E-23	MROH2A	Bilirubin levels	GCST001091
rs10179094	7.00E-23	UGT1A7,UGT1A9,UGT1A8,UGT1A10	Total bilirubin levels x insomnia interaction	GCST90026657
rs10179094	7.00E-23	UGT1A7,UGT1A9,UGT1A8,UGT1A10	Direct bilirubin levels x insomnia interaction	GCST90026656
rs887829	8.00E-23	UGT1A5,UGT1A9,UGT1A10,UGT1A7,UGT1A4,UGT1A8,UGT1A3,UGT1A6	Serum metabolite levels	GCST002388
rs10167119	8.00E-23	UGT1A10,UGT1A8,UGT1A9	Direct bilirubin levels x insomnia interaction	GCST90026656
rs4530361	8.00E-23	UGT1A8,UGT1A10,UGT1A9	Direct bilirubin levels x insomnia interaction	GCST90026656
rs10167119	8.00E-23	UGT1A10,UGT1A8,UGT1A9	Total bilirubin levels x insomnia interaction	GCST90026657
rs4530361	8.00E-23	UGT1A8,UGT1A10,UGT1A9	Total bilirubin levels x insomnia interaction	GCST90026657
rs539982180	8.00E-23	TRPM8,MSL3B	Total bilirubin levels (UKB data field 30840)	GCST90468104
rs10197460	9.00E-23	UGT1A10,UGT1A8,UGT1A9	Total bilirubin levels x insomnia interaction	GCST90026657
rs7586110	9.00E-23	UGT1A8,UGT1A9,UGT1A10	Total bilirubin levels x insomnia interaction	GCST90026657
rs10197460	9.00E-23	UGT1A10,UGT1A8,UGT1A9	Direct bilirubin levels x insomnia interaction	GCST90026656
rs7586110	9.00E-23	UGT1A8,UGT1A9,UGT1A10	Direct bilirubin levels x insomnia interaction	GCST90026656
rs17863787	1.00E-22	UGT1A7,UGT1A6,UGT1A9,UGT1A10,UGT1A8	Total bilirubin levels in HIV-1 infection	GCST002745
rs7577677	1.00E-22	UGT1A9,UGT1A10,UGT1A7,UGT1A8	Direct bilirubin levels x insomnia interaction	GCST90026656
rs7577677	1.00E-22	UGT1A9,UGT1A10,UGT1A7,UGT1A8	Total bilirubin levels x insomnia interaction	GCST90026657
rs72551330	1.00E-22	UGT1A10,UGT1A8,UGT1A9	3-carboxy-4-methyl-5-pentyl-2-furanpropionate (3-CMPFP) levels	GCST90200172
rs887829	2.00E-22	UGT1A5,UGT1A9,UGT1A10,UGT1A7,UGT1A4,UGT1A8,UGT1A3,UGT1A6	Bilirubin levels	GCST001324
rs111741722	2.00E-22	UGT1A3,UGT1A10,UGT1A7,UGT1A8,UGT1A5,UGT1A4,UGT1A9,UGT1A6	Blood protein levels	GCST006585
rs4148325	2.00E-22	UGT1A9,UGT1A7,UGT1A3,UGT1A5,UGT1A8,UGT1A1,UGT1A4,UGT1A10,UGT1A6	Xanthurenate levels	GCST90199626
rs17863834	3.00E-22	MSL3B,TRPM8	Direct bilirubin levels	GCST90019505
rs12995326	3.00E-22	MROH2A	Total bilirubin levels x insomnia interaction	GCST90026657
rs12995326	3.00E-22	MROH2A	Direct bilirubin levels x insomnia interaction	GCST90026656
rs11692021	3.00E-22	UGT1A8,UGT1A9,UGT1A7,UGT1A10	Total bilirubin levels x insomnia interaction	GCST90026657
rs11692021	3.00E-22	UGT1A8,UGT1A9,UGT1A7,UGT1A10	Direct bilirubin levels x insomnia interaction	GCST90026656
rs11563210	5.00E-22	TRPM8	Total bilirubin levels	GCST90019521
rs4148323	5.00E-22	UGT1A10,UGT1A6,UGT1A9,UGT1A4,UGT1A8,UGT1A1,UGT1A7,UGT1A3,UGT1A5	Perinatal jaundice (PheCode 656.8)	GCST90651183
rs3771338	6.00E-22	HJURP	Direct bilirubin levels	GCST90019505
rs7581102	6.00E-22	MROH2A	Total bilirubin levels x insomnia interaction	GCST90026657
rs7581102	6.00E-22	MROH2A	Direct bilirubin levels x insomnia interaction	GCST90026656
rs55664157	6.00E-22	DGKD	Platelet count	GCST90002402
rs28899170	8.00E-22	UGT1A8,UGT1A7,UGT1A10,UGT1A9,UGT1A6	sulfate of piperine metabolite C16H17NO3 (1)* levels in chronic kidney di	GCST90266005
rs559511484	8.00E-22	MROH2A	Total bilirubin levels (UKB data field 30840)	GCST90468104
rs3732218	9.00E-22	UGT1A6,UGT1A7,UGT1A10,UGT1A9,UGT1A5,UGT1A8	Testosterone levels	GCST90483487
rs13002399	1.00E-21	USP40,UGT1A12P	Calcium levels	GCST90019500
rs72551330	1.00E-21	UGT1A10,UGT1A8,UGT1A9	3-carboxy-4-methyl-5-pentyl-2-furanpropionate (3-CMPFP) (X-02249) level	GCST90102794
rs6742078	1.00E-21	UGT1A1,UGT1A9,UGT1A4,UGT1A6,UGT1A5,UGT1A10,UGT1A7,UGT1A3,UGT1A8	Abnormal results of function study of liver (PheCode 573.7)	GCST90480346
rs145189643	1.00E-21	HJURP,MSL3B	Total bilirubin levels (UKB data field 30840)	GCST90468104
rs62192905	2.00E-21	ATG16L1	Total bilirubin levels	GCST90019521
rs887829	2.00E-21	UGT1A5,UGT1A9,UGT1A10,UGT1A7,UGT1A4,UGT1A8,UGT1A3,UGT1A6	Serum levels of protein PAXIP1	GCST90087296
rs6706384	2.00E-21	DGKD	Type 2 diabetes	GCST90492734
rs6431630	3.00E-21	UGT1A9,UGT1A4,UGT1A5,UGT1A6,UGT1A10,UGT1A1,UGT1A7,UGT1A8,UGT1A3	Non-HDL cholesterol levels	GCST90239670
rs4616477	4.00E-21	TRPM8,SPP2	Direct bilirubin levels	GCST90019505
rs6759892	4.00E-21	UGT1A8,UGT1A9,UGT1A7,UGT1A10,UGT1A6	Total bilirubin levels x insomnia interaction	GCST90026657
rs6759892	4.00E-21	UGT1A8,UGT1A9,UGT1A7,UGT1A10,UGT1A6	Direct bilirubin levels x insomnia interaction	GCST90026656
rs575144771	4.00E-21	TRPM8,MSL3B	Total bilirubin levels (UKB data field 30840)	GCST90468104
rs113483143	5.00E-21	DGKD	Direct bilirubin levels	GCST90019505
rs13002774	5.00E-21	UGT1A8,UGT1A9,UGT1A7,UGT1A10	Total bilirubin levels x insomnia interaction	GCST90026657
rs4338954	5.00E-21	UGT1A9,UGT1A10,UGT1A8,UGT1A7	Total bilirubin levels x insomnia interaction	GCST90026657
rs4553819	5.00E-21	UGT1A10,UGT1A9,UGT1A8,UGT1A7	Total bilirubin levels x insomnia interaction	GCST90026657
rs11902131	5.00E-21	UGT1A10,UGT1A7,UGT1A9,UGT1A8	Total bilirubin levels x insomnia interaction	GCST90026657
rs6753320	5.00E-21	UGT1A7,UGT1A9,UGT1A8,UGT1A10	Total bilirubin levels x insomnia interaction	GCST90026657
rs6736508	5.00E-21	UGT1A9,UGT1A10,UGT1A7,UGT1A8	Total bilirubin levels x insomnia interaction	GCST90026657
rs6753569	5.00E-21	UGT1A10,UGT1A8,UGT1A7,UGT1A9	Total bilirubin levels x insomnia interaction	GCST90026657
rs6736743	5.00E-21	UGT1A10,UGT1A9,UGT1A7,UGT1A8	Total bilirubin levels x insomnia interaction	GCST90026657
rs10203266	5.00E-21	UGT1A7,UGT1A10,UGT1A9,UGT1A8	Total bilirubin levels x insomnia interaction	GCST90026657
rs10168155	5.00E-21	UGT1A9,UGT1A7,UGT1A8,UGT1A10	Total bilirubin levels x insomnia interaction	GCST90026657
rs10175809	5.00E-21	UGT1A7,UGT1A9,UGT1A10,UGT1A8	Total bilirubin levels x insomnia interaction	GCST90026657
rs10168333	5.00E-21	UGT1A8,UGT1A9,UGT1A10,UGT1A7	Total bilirubin levels x insomnia interaction	GCST90026657
rs11680450	5.00E-21	UGT1A8,UGT1A9,UGT1A10,UGT1A7	Total bilirubin levels x insomnia interaction	GCST90026657
rs10171367	5.00E-21	UGT1A9,UGT1A10,UGT1A7,UGT1A8	Total bilirubin levels x insomnia interaction	GCST90026657
rs7563561	5.00E-21	UGT1A7,UGT1A10,UGT1A9,UGT1A8	Total bilirubin levels x insomnia interaction	GCST90026657
rs7608175	5.00E-21	UGT1A9,UGT1A7,UGT1A10,UGT1A8	Total bilirubin levels x insomnia interaction	GCST90026657
rs45615240	5.00E-21	UGT1A8,UGT1A9,UGT1A10,UGT1A7	Total bilirubin levels x insomnia interaction	GCST90026657
rs10445704	5.00E-21	UGT1A10,UGT1A8,UGT1A9,UGT1A6,UGT1A7	Total bilirubin levels x insomnia interaction	GCST90026657
rs6736743	5.00E-21	UGT1A10,UGT1A9,UGT1A7,UGT1A8	Direct bilirubin levels x insomnia interaction	GCST90026656
rs10203266	5.00E-21	UGT1A7,UGT1A10,UGT1A9,UGT1A8	Direct bilirubin levels x insomnia interaction	GCST90026656
rs10168155	5.00E-21	UGT1A9,UGT1A7,UGT1A8,UGT1A10	Direct bilirubin levels x insomnia interaction	GCST90026656
rs10175809	5.00E-21	UGT1A7,UGT1A9,UGT1A10,UGT1A8	Direct bilirubin levels x insomnia interaction	GCST90026656
rs10171367	5.00E-21	UGT1A9,UGT1A10,UGT1A7,UGT1A8	Direct bilirubin levels x insomnia interaction	GCST90026656
rs7563561	5.00E-21	UGT1A7,UGT1A10,UGT1A9,UGT1A8	Direct bilirubin levels x insomnia interaction	GCST90026656
rs13002774	5.00E-21	UGT1A8,UGT1A9,UGT1A7,UGT1A10	Direct bilirubin levels x insomnia interaction	GCST90026656
rs4338954	5.00E-21	UGT1A9,UGT1A10,UGT1A8,UGT1A7	Direct bilirubin levels x insomnia interaction	GCST90026656
rs4553819	5.00E-21	UGT1A10,UGT1A9,UGT1A8,UGT1A7	Direct bilirubin levels x insomnia interaction	GCST90026656
rs11902131	5.00E-21	UGT1A10,UGT1A7,UGT1A9,UGT1A8	Direct bilirubin levels x insomnia interaction	GCST90026656
rs6753320	5.00E-21	UGT1A7,UGT1A9,UGT1A8,UGT1A10	Direct bilirubin levels x insomnia interaction	GCST90026656
rs6736508	5.00E-21	UGT1A9,UGT1A10,UGT1A7,UGT1A8	Direct bilirubin levels x insomnia interaction	GCST90026656
rs6753569	5.00E-21	UGT1A10,UGT1A8,UGT1A7,UGT1A9	Direct bilirubin levels x insomnia interaction	GCST90026656
rs10168333	5.00E-21	UGT1A8,UGT1A9,UGT1A10,UGT1A7	Direct bilirubin levels x insomnia interaction	GCST90026656
rs11680450	5.00E-21	UGT1A8,UGT1A9,UGT1A10,UGT1A7	Direct bilirubin levels x insomnia interaction	GCST90026656
rs7608175	5.00E-21	UGT1A9,UGT1A7,UGT1A10,UGT1A8	Direct bilirubin levels x insomnia interaction	GCST90026656
rs45615240	5.00E-21	UGT1A8,UGT1A9,UGT1A10,UGT1A7	Direct bilirubin levels x insomnia interaction	GCST90026656
rs10445704	5.00E-21	UGT1A10,UGT1A8,UGT1A9,UGT1A6,UGT1A7	Direct bilirubin levels x insomnia interaction	GCST90026656
rs4148325	5.00E-21	UGT1A9,UGT1A7,UGT1A3,UGT1A5,UGT1A8,UGT1A1,UGT1A4,UGT1A10,UGT1A6	Bilirubin (z,z) levels	GCST90128875
rs12623271	6.00E-21	UGT1A10,UGT1A8,UGT1A9,UGT1A7	Total bilirubin levels x insomnia interaction	GCST90026657
rs13015720	6.00E-21	UGT1A8,UGT1A10,UGT1A9,UGT1A6,UGT1A7	Total bilirubin levels x insomnia interaction	GCST90026657
rs12623271	6.00E-21	UGT1A10,UGT1A8,UGT1A9,UGT1A7	Direct bilirubin levels x insomnia interaction	GCST90026656

rs13015720	6.00E-21	UGT1A8,UGT1A10,UGT1A9,UGT1A6,UGT1A7	Direct bilirubin levels x insomnia interaction	GCST90026656
rs6724485	6.00E-21	UGT1A8,UGT1A10,UGT1A9,UGT1A7	Total bilirubin levels x insomnia interaction	GCST90026657
rs4347832	6.00E-21	UGT1A7,UGT1A8,UGT1A9,UGT1A10	Total bilirubin levels x insomnia interaction	GCST90026657
rs6724485	6.00E-21	UGT1A8,UGT1A10,UGT1A9,UGT1A7	Direct bilirubin levels x insomnia interaction	GCST90026656
rs4347832	6.00E-21	UGT1A7,UGT1A8,UGT1A9,UGT1A10	Direct bilirubin levels x insomnia interaction	GCST90026656
rs17862931	7.00E-21	TRPM8	Direct bilirubin levels	GCST90019505
rs3732218	7.00E-21	UGT1A6,UGT1A7,UGT1A10,UGT1A9,UGT1A5,UGT1A8	Testosterone levels	GCST90483498
rs14243	7.00E-21	INPP5D	n corpuscular hemoglobin concentration (MCHC, mean, inv-norm transforr	GCST90475458
rs4261716	8.00E-21	UGT1A7,UGT1A8,UGT1A9,UGT1A10	Total bilirubin levels x insomnia interaction	GCST90026657
rs4261716	8.00E-21	UGT1A7,UGT1A8,UGT1A9,UGT1A10	Direct bilirubin levels x insomnia interaction	GCST90026656
rs144759885	9.00E-21	UGT1A8,UGT1A10	Direct bilirubin levels	GCST90019505
rs1057258	9.00E-21	INPP5D	Eosinophill count (UKB data field 30150)	GCST90468068
rs10166942	1.00E-20	MSL3B,TRPM8	Pain (pleiotropy)	GCST90104573
rs2008584	1.00E-20	UGT1A7,UGT1A6,UGT1A8,UGT1A5,UGT1A9,UGT1A4,UGT1A10	Total bilirubin levels	GCST90429160
rs869283	1.00E-20	UGT1A9,UGT1A10,UGT1A6,UGT1A8,UGT1A5,UGT1A7	X-11880 levels	GCST90103254
rs72551330	2.00E-20	UGT1A10,UGT1A8,UGT1A9	Gamma-CEHC levels	GCST90245198
rs6742078	3.00E-20	UGT1A1,UGT1A9,UGT1A4,UGT1A6,UGT1A5,UGT1A10,UGT1A7,UGT1A3,UGT1A8	Bilirubin (z,z) levels	GCST90104978
rs6431630	3.00E-20	UGT1A9,UGT1A4,UGT1A5,UGT1A6,UGT1A10,UGT1A1,UGT1A7,UGT1A8,UGT1A3	Low density lipoprotein cholesterol levels	GCST90239658
rs4663971	3.00E-20	UGT1A3,UGT1A9,UGT1A5,UGT1A4,UGT1A10,UGT1A6,UGT1A1,UGT1A8,UGT1A7	Total bilirubin levels	GCST90429160
rs8330	4.00E-20	UGT1A7,UGT1A8,UGT1A6,UGT1A9,UGT1A4,UGT1A5,UGT1A3,UGT1A10,UGT1A1	Total bilirubin levels	GCST005980
rs2011425	6.00E-20	UGT1A8,UGT1A6,UGT1A10,UGT1A9,UGT1A5,UGT1A4,UGT1A7	Bioavailable testosterone levels	GCST90012103
rs2011425	8.00E-20	UGT1A8,UGT1A6,UGT1A10,UGT1A9,UGT1A5,UGT1A4,UGT1A7	Urine X-21851 levels in chronic kidney disease	GCST90266550
rs887829	9.00E-20	UGT1A5,UGT1A9,UGT1A10,UGT1A7,UGT1A4,UGT1A8,UGT1A3,UGT1A6	Bilirubin levels	GCST001976
rs3755319	9.00E-20	UGT1A8,UGT1A10,UGT1A5,UGT1A7,UGT1A3,UGT1A6,UGT1A4,UGT1A9	Total bilirubin levels in HIV-1 infection	GCST002745
rs17862932	9.00E-20	TRPM8	Total bilirubin levels	GCST90019521
rs4148325	1.00E-19	UGT1A9,UGT1A7,UGT1A3,UGT1A5,UGT1A8,UGT1A1,UGT1A4,UGT1A10,UGT1A6	Biliverdin levels	GCST90104981
rs6742078	1.00E-19	UGT1A1,UGT1A9,UGT1A4,UGT1A6,UGT1A5,UGT1A10,UGT1A7,UGT1A3,UGT1A8	Serum levels of protein CKAP4	GCST90089745
rs4148325	2.00E-19	UGT1A9,UGT1A7,UGT1A3,UGT1A5,UGT1A8,UGT1A1,UGT1A4,UGT1A10,UGT1A6	Serum bilirubin levels in metabolic syndrome	GCST007018
rs139085833	2.00E-19	DGKD	Total bilirubin levels	GCST90019521
rs11674391	2.00E-19	TRPM8,MSL3B	Total bilirubin levels	GCST90019521
rs4663997	2.00E-19	TRPM8	Total bilirubin levels	GCST90019521
rs1976391	2.00E-19	UGT1A9,UGT1A6,UGT1A5,UGT1A3,UGT1A8,UGT1A10,UGT1A7,UGT1A4	total cholesterol (mean, inv-norm transformed)	GCST90480715
rs144267520	2.00E-19	MSL3B,HJURP	Total bilirubin levels (UKB data field 30840)	GCST90468104
rs1550532	3.00E-19	DGKD	Bilirubin levels	GCST000730
rs183383266	3.00E-19	SAG	Total bilirubin levels	GCST90019521
rs1057258	3.00E-19	INPP5D	eosinophil (absolute count, mean, inv-norm transformed)	GCST90479602
rs838718	3.00E-19	DGKD	Calculus of kidney (PheCode 594.1)	GCST90480395
rs11563062	3.00E-19	TRPM8	Total bilirubin levels (UKB data field 30840)	GCST90468104
rs2012736	5.00E-19	UGT1A9,UGT1A8,UGT1A6,UGT1A7,UGT1A10,UGT1A5	Total testosterone levels	GCST90012113
rs4148325	6.00E-19	UGT1A9,UGT1A7,UGT1A3,UGT1A5,UGT1A8,UGT1A1,UGT1A4,UGT1A10,UGT1A6	Bilirubin (E,E) levels	GCST90104984
rs4148325	6.00E-19	UGT1A9,UGT1A7,UGT1A3,UGT1A5,UGT1A8,UGT1A1,UGT1A4,UGT1A10,UGT1A6	Biliverdin levels in chronic kidney disease	GCST90257495
rs72551330	6.00E-19	UGT1A10,UGT1A8,UGT1A9	olite levels (indole-3-carboxylic acid; indole-3-carboxylate; Indole_3_carbo	GCST90300064
rs1965629	7.00E-19	TRPM8,MSL3B	Migraine	GCST003986
rs11568318	7.00E-19	UGT1A9,UGT1A4,UGT1A5,UGT1A10,UGT1A6,UGT1A3,UGT1A8,UGT1A7	Low density lipoprotein cholesterol levels	GCST90002412
rs4148325	8.00E-19	UGT1A9,UGT1A7,UGT1A3,UGT1A5,UGT1A8,UGT1A1,UGT1A4,UGT1A10,UGT1A6	Bilirubin (E,Z or Z,E) levels	GCST90104985
rs4148325	8.00E-19	UGT1A9,UGT1A7,UGT1A3,UGT1A5,UGT1A8,UGT1A1,UGT1A4,UGT1A10,UGT1A6	Metabolite peak levels (QI10761)	GCST90176447
rs114630474	9.00E-19	MSL3B	Total bilirubin levels	GCST90019521
rs9287650	9.00E-19	TRPM8,SPP2	Total bilirubin levels	GCST90019521
rs35754645	1.00E-18	UGT1A9,UGT1A8,UGT1A3,UGT1A5,UGT1A6,UGT1A7,UGT1A4,UGT1A10	Liver disease biomarker	GCST008965
rs143661480	1.00E-18	USP40	Total bilirubin levels	GCST90019521
rs140874442	1.00E-18	HJURP	Total bilirubin levels	GCST90019521
rs13035837	1.00E-18	DGKD	platelet count (mean, inv-norm transformed)	GCST90480651
rs4148325	2.00E-18	UGT1A9,UGT1A7,UGT1A3,UGT1A5,UGT1A8,UGT1A1,UGT1A4,UGT1A10,UGT1A6	Gallstone disease	GCST90128518
rs4148325	2.00E-18	UGT1A9,UGT1A7,UGT1A3,UGT1A5,UGT1A8,UGT1A1,UGT1A4,UGT1A10,UGT1A6	Urine xanthurenate levels in chronic kidney disease	GCST90266869
rs838717	2.00E-18	DGKD	Urinary calculus (PheCode 594)	GCST90480398
rs62195072	3.00E-18	DGKD,SAG	Sex hormone-binding globulin levels adjusted for BMI	GCST90012110
rs887829	3.00E-18	UGT1A5,UGT1A9,UGT1A10,UGT1A7,UGT1A4,UGT1A8,UGT1A3,UGT1A6	Jaundice (not of newborn) (PheCode 573.5)	GCST90476534
rs2304776	4.00E-18	SAG	Lymphocyte count	GCST90002316
rs62192317	4.00E-18	TRPM8,MSL3B	Total bilirubin levels	GCST90019521
rs838720	4.00E-18	DGKD	Type 2 diabetes	GCST010557
rs1057258	5.00E-18	INPP5D	eosinophil (absolute count, maximum, inv-norm transformed)	GCST90479601
rs6757418	6.00E-18	ATG16L1	Total bilirubin levels	GCST90019521
rs28626554	6.00E-18	MROH2A	Total bilirubin levels	GCST90019521
rs2011425	6.00E-18	UGT1A8,UGT1A6,UGT1A10,UGT1A9,UGT1A5,UGT1A4,UGT1A7	Free testosterone levels	GCST90239825
rs1057258	6.00E-18	INPP5D	eosinophil (absolute count, mean, inv-norm transformed)	GCST90475291
rs4148325	6.00E-18	UGT1A9,UGT1A7,UGT1A3,UGT1A5,UGT1A8,UGT1A1,UGT1A4,UGT1A10,UGT1A6	total cholesterol (minimum, inv-norm transformed)	GCST90480716
rs6431630	6.00E-18	UGT1A9,UGT1A4,UGT1A5,UGT1A6,UGT1A10,UGT1A1,UGT1A7,UGT1A8,UGT1A3	Total cholesterol levels	GCST90662895
rs2741045	6.00E-18	UGT1A10,UGT1A8	total cholesterol (maximum, inv-norm transformed)	GCST90480714
rs17863840	7.00E-18	TRPM8	Direct bilirubin levels	GCST90019505
rs2305538	8.00E-18	DGKD	estimated glomerular filtration rate (eGFR, mean, inv-norm transformed)	GCST90475282
rs7564935	9.00E-18	UGT1A3,UGT1A10,UGT1A5,UGT1A4,UGT1A7,UGT1A8,UGT1A9,UGT1A6	Bilirubin levels	GCST009051
rs887829	1.00E-17	UGT1A5,UGT1A9,UGT1A10,UGT1A7,UGT1A4,UGT1A8,UGT1A3,UGT1A6	Serum metabolite levels	GCST002388
rs17863787	1.00E-17	UGT1A7,UGT1A6,UGT1A9,UGT1A10,UGT1A8	Serum bilirubin levels in metabolic syndrome	GCST007018
rs72972302	1.00E-17	ATG16L1	Direct bilirubin levels	GCST90019505
rs838717	1.00E-17	DGKD	Kidney stone disease	GCST90428893
rs35754645	1.00E-17	UGT1A9,UGT1A8,UGT1A3,UGT1A5,UGT1A6,UGT1A7,UGT1A4,UGT1A10	X-23974 levels	GCST90140579
rs141527994	1.00E-17	UGT1A11P,UGT1A12P	Total bilirubin levels (UKB data field 30840)	GCST90468104
rs143487779	2.00E-17	UGT1A9,UGT1A8,UGT1A10	Total bilirubin levels	GCST90019521
rs111809359	2.00E-17	MROH2A	Total bilirubin levels	GCST90019521
rs3755320	3.00E-17	UGT1A9,UGT1A6,UGT1A10,UGT1A5,UGT1A7,UGT1A8	Pregnenetriol sulfate levels	GCST90200241
rs117809958	3.00E-17	ATG16L1	Fasting blood glucose	GCST90662896
rs6742078	4.00E-17	UGT1A1,UGT1A9,UGT1A4,UGT1A6,UGT1A5,UGT1A10,UGT1A7,UGT1A3,UGT1A8	X-11530 levels	GCST90104992
rs1976391	4.00E-17	UGT1A9,UGT1A6,UGT1A5,UGT1A3,UGT1A8,UGT1A10,UGT1A7,UGT1A4	Serum metabolite concentrations in chronic kidney disease	GCST012353
rs3792109	4.00E-17	ATG16L1,SCARNA5	Crohn's disease vs ulcerative colitis (ordinary least squares (OLS))	GCST90016611
rs2305538	4.00E-17	DGKD	creatinine (mean, inv-norm transformed)	GCST90475229
rs1441093	4.00E-17	DGKD	Height	GCST90245848
rs14243	4.00E-17	INPP5D	corpuscular hemoglobin concentration (MCHC, maximum, inv-norm transfo	GCST90475454
rs56263467	4.00E-17	DGKD	Platelet count (UKB data field 30080)	GCST90468095
rs62192955	5.00E-17	ATG16L1	Calcium levels	GCST90019500
rs139085833	5.00E-17	DGKD	Direct bilirubin levels	GCST90019505
rs1105880	5.00E-17	UGT1A6,UGT1A7,UGT1A9,UGT1A10,UGT1A8	Cholelithiasis	GCST90018819
rs34339006	8.00E-17	DGKD	Calcium levels	GCST005982
rs185330631	9.00E-17	TRPM8	Total bilirubin levels (UKB data field 30840)	GCST90468104

rs4148325	1.00E-16	UGT1A9,UGT1A7,UGT1A3,UGT1A5,UGT1A8,UGT1A1,UGT1A4,UGT1A10,UGT1A6	low density lipoprotein cholesterol (LDLC, mean, inv-norm transformed)	GCST90479661
rs34983651	1.00E-16	UGT1A6,UGT1A4,UGT1A8,UGT1A5,UGT1A7,UGT1A3,UGT1A9,UGT1A10	Protein-tyrosine kinase 6 levels (PTK6.3832.51.1)	GCST90242533
rs3792109	2.00E-16	ATG16L1,SCARNA5	Crohn's disease vs rheumatoid arthritis (ordinary least squares (OLS))	GCST90016610
rs2011425	2.00E-16	UGT1A8,UGT1A6,UGT1A10,UGT1A9,UGT1A5,UGT1A4,UGT1A7	Pregnenetriol sulfate levels	GCST90140161
rs137886124	3.00E-16	SAG	Total bilirubin levels	GCST90019521
rs6742078	3.00E-16	UGT1A1,UGT1A9,UGT1A4,UGT1A6,UGT1A5,UGT1A10,UGT1A7,UGT1A3,UGT1A8	X-24849 levels	GCST90104994
rs2013021	3.00E-16	UGT1A9,UGT1A8,UGT1A10,UGT1A6,UGT1A7	Total testosterone levels	GCST90239819
rs4663969	4.00E-16	UGT1A9,UGT1A4,UGT1A3,UGT1A5,UGT1A8,UGT1A7,UGT1A6,UGT1A10	Total bilirubin levels in HIV-1 infection	GCST002745
rs17863796	4.00E-16	UGT1A6,UGT1A3,UGT1A7,UGT1A8,UGT1A9,DNAJB3,UGT1A4,UGT1A10,UGT1A5	Direct bilirubin levels	GCST90019505
rs17868304	4.00E-16	UGT1A11P,UGT1A8	Cancer of bladder (PheCode 189.2)	GCST90479810
rs4148325	4.00E-16	UGT1A9,UGT1A7,UGT1A3,UGT1A5,UGT1A8,UGT1A1,UGT1A4,UGT1A10,UGT1A6	ow density lipoprotein cholesterol (LDLC, maximum, inv-norm transformed	GCST90479660
rs139502797	4.00E-16	TRPM8,SPP2	Total bilirubin levels (UKB data field 30840)	GCST90468104
rs10173355	5.00E-16	UGT1A9,UGT1A7,UGT1A8,UGT1A10	Total bilirubin levels in HIV-1 infection	GCST002745
rs4477910	5.00E-16	UGT1A9,UGT1A10,UGT1A5,UGT1A6,UGT1A3,UGT1A4,UGT1A8,UGT1A7	Total bilirubin levels in HIV-1 infection	GCST002745
rs3771341	5.00E-16	UGT1A6,UGT1A10,UGT1A4,UGT1A5,UGT1A8,UGT1A1,UGT1A7,UGT1A9,UGT1A3	X-11522 levels	GCST90104995
rs6742078	5.00E-16	UGT1A1,UGT1A9,UGT1A4,UGT1A6,UGT1A5,UGT1A10,UGT1A7,UGT1A3,UGT1A8	X-21448 levels	GCST90104996
rs2362290	6.00E-16	TRPM8,MSL3B	Headache	GCST005337
rs3828309	6.00E-16	ATG16L1	CALCOCO1/IKBKG protein level ratio	GCST90313602
rs145496107	6.00E-16	MROH2A	Total bilirubin levels (UKB data field 30840)	GCST90468104
rs12988953	7.00E-16	TRPM8	Headache or migraine	GCST90267554
rs4663997	8.00E-16	TRPM8	Direct bilirubin levels	GCST90019505
rs17863807	9.00E-16	MROH2A	GLIPR1 protein levels	GCST90469357
rs3755322	1.00E-15	UGT1A5,UGT1A7,UGT1A10,UGT1A6,UGT1A9,UGT1A8	Testosterone levels	GCST90019520
rs55664157	1.00E-15	DGKD	Platelet count	GCST90018969
rs1057258	1.00E-15	INPP5D	eosinophil (fraction, minimum, inv-norm transformed)	GCST90479606
rs1976391	1.00E-15	UGT1A9,UGT1A6,UGT1A5,UGT1A3,UGT1A8,UGT1A10,UGT1A7,UGT1A4	Androstenediol levels	GCST90101914
rs10803624	2.00E-15	SAG	Lymphocyte count	GCST90002320
rs76114682	2.00E-15	TRPM8,SPP2	Total bilirubin levels	GCST90019521
rs17868304	2.00E-15	UGT1A11P,UGT1A8	Malignant neoplasm of bladder (PheCode 189.21)	GCST90479809
rs117809958	2.00E-15	ATG16L1	Type 2 diabetes	GCST010118
rs6742078	2.00E-15	UGT1A1,UGT1A9,UGT1A4,UGT1A6,UGT1A5,UGT1A10,UGT1A7,UGT1A3,UGT1A8	ow density lipoprotein cholesterol (LDLC, minimum, inv-norm transformed	GCST90479662
rs2305538	2.00E-15	DGKD	stimated glomerular filtration rate (eGFR, minimum, inv-norm transformed	GCST90475285
rs10210302	2.00E-15	ATG16L1	AXIN1/CALCOCO1 protein level ratio	GCST90313402
rs7567229	3.00E-15	UGT1A6,UGT1A9,UGT1A7,UGT1A10,UGT1A8	Bilirubin levels in chronic kidney disease	GCST90257494
rs35754645	4.00E-15	UGT1A9,UGT1A8,UGT1A3,UGT1A5,UGT1A6,UGT1A7,UGT1A4,UGT1A10	X-10458 levels	GCST90140351
rs13035837	4.00E-15	DGKD	platelet count (maximum, inv-norm transformed)	GCST90480650
rs74320205	4.00E-15	DGKD,SAG	Platelet count	GCST90662881
rs6724624	5.00E-15	MSL3B,TRPM8	Chronic pain (confirmatory factor analysis Factor 18)	GCST90309352
rs838717	5.00E-15	DGKD	Hemoglobin A1c levels	GCST90018958
rs887829	5.00E-15	UGT1A5,UGT1A9,UGT1A10,UGT1A7,UGT1A4,UGT1A8,UGT1A3,UGT1A6	Metabolite peak levels (QI1249)	GCST90176646
rs17868335	5.00E-15	UGT1A8,UGT1A6,UGT1A10,UGT1A9,UGT1A7,UGT1A5	Vitamin D deficiency (PheCode 261.4)	GCST90479912
rs35754645	5.00E-15	UGT1A9,UGT1A8,UGT1A3,UGT1A5,UGT1A6,UGT1A7,UGT1A4,UGT1A10	Biliverdin levels in chronic kidney disease	GCST90258100
rs2361502	6.00E-15	MROH2A	Total bilirubin levels x insomnia interaction	GCST90026657
rs2361502	6.00E-15	MROH2A	Direct bilirubin levels x insomnia interaction	GCST90026656
rs17868336	6.00E-15	UGT1A3,UGT1A7,UGT1A4,UGT1A8,UGT1A10,UGT1A5,UGT1A6,UGT1A9	Neonatal jaundice (maternal effect)	GCST90435146
rs72551330	6.00E-15	UGT1A10,UGT1A8,UGT1A9	Indoleacetate levels	GCST90245260
rs10929285	6.00E-15	UGT1A9,UGT1A8,UGT1A10,UGT1A7,UGT1A6	Skin yellow/blue component	GCST90320259
rs929596	8.00E-15	UGT1A3,UGT1A5,UGT1A4,UGT1A6,UGT1A9,UGT1A8,UGT1A10,UGT1A1,UGT1A7	Serum bilirubin levels in metabolic syndrome	GCST007018
rs838717	8.00E-15	DGKD	Urolithiasis	GCST90018935
rs3755321	8.00E-15	UGT1A9,UGT1A6,UGT1A10,UGT1A8,UGT1A5,UGT1A7	Testosterone levels (UKB data field 30850)	GCST90468103
rs4663983	9.00E-15	MSL3B,TRPM8	Medication use (antimigraine preparations)	GCST007939
rs4148324	1.00E-14	UGT1A3,UGT1A10,UGT1A4,UGT1A6,UGT1A7,UGT1A8,UGT1A9,UGT1A1,UGT1A5	NmrA-like family domain-containing protein 1 levels	GCST90422389
rs17862875	1.00E-14	UGT1A3,UGT1A5,UGT1A9,UGT1A4,UGT1A10,UGT1A7,UGT1A8,UGT1A6	Serum levels of protein TYK2	GCST90088986
rs117809958	1.00E-14	ATG16L1	Type 2 diabetes	GCST90018706
rs71421698	1.00E-14	DGKD	Lymphocyte count	GCST90002388
rs887829	1.00E-14	UGT1A5,UGT1A9,UGT1A10,UGT1A7,UGT1A4,UGT1A8,UGT1A3,UGT1A6	X-11530 levels	GCST90128949
rs887829	1.00E-14	UGT1A5,UGT1A9,UGT1A10,UGT1A7,UGT1A4,UGT1A8,UGT1A3,UGT1A6	X-11793 levels	GCST90128954
rs4663983	1.00E-14	MSL3B,TRPM8	Height	GCST90245848
rs67863435	1.00E-14	DGKD	platelet count (minimum, inv-norm transformed)	GCST90480652
rs34983651	1.00E-14	UGT1A6,UGT1A4,UGT1A8,UGT1A5,UGT1A7,UGT1A3,UGT1A9,UGT1A10	Cholesterol levels (UKB data field 30690)	GCST90468066
rs6709005	1.00E-14	TRPM8	Migraine	GCST90468142
rs111741722	2.00E-14	UGT1A3,UGT1A10,UGT1A7,UGT1A8,UGT1A5,UGT1A4,UGT1A9,UGT1A6	Blood protein levels	GCST006585
rs2070959	2.00E-14	UGT1A9,UGT1A7,UGT1A10,UGT1A8,UGT1A6	Serum bilirubin levels in metabolic syndrome	GCST007018
rs114896551	2.00E-14	TRPM8,MSL3B	Total bilirubin levels	GCST90019521
rs12986677	2.00E-14	MROH2A	Total bilirubin levels x insomnia interaction	GCST90026657
rs12986677	2.00E-14	MROH2A	Direct bilirubin levels x insomnia interaction	GCST90026656
rs6431270	2.00E-14	SAG,ATG16L1	Serum phosphate levels	GCST90019516
rs76114682	2.00E-14	TRPM8,SPP2	Direct bilirubin levels	GCST90019505
rs4148325	2.00E-14	UGT1A9,UGT1A7,UGT1A3,UGT1A5,UGT1A8,UGT1A1,UGT1A4,UGT1A10,UGT1A6	X-16946 levels	GCST90105004
rs6747843	2.00E-14	UGT1A8,UGT1A10,UGT1A9,UGT1A3,UGT1A6,UGT1A5,UGT1A7,UGT1A4	sulfate of piperine metabolite C18H21NO3 (3)* levels in chronic kidney di	GCST90266020
rs34983651	2.00E-14	UGT1A6,UGT1A4,UGT1A8,UGT1A5,UGT1A7,UGT1A3,UGT1A9,UGT1A10	Total cholesterol levels	GCST90018974
rs2971869	2.00E-14	DGKD,USP40	Aspartate aminotransferase levels	GCST90662897
rs838717	2.00E-14	DGKD	Glycated haemoglobin HbA1c levels (UKB data field 30750)	GCST90468072
rs545335183	2.00E-14	SPP2,TRPM8	Total bilirubin levels (UKB data field 30840)	GCST90468104
rs34983651	2.00E-14	UGT1A6,UGT1A4,UGT1A8,UGT1A5,UGT1A7,UGT1A3,UGT1A9,UGT1A10	Testis-specific chromodomain protein Y 1 levels (CDY1.7097.8.3)	GCST90242992
rs2011425	2.00E-14	UGT1A8,UGT1A6,UGT1A10,UGT1A9,UGT1A5,UGT1A4,UGT1A7	INSL3 protein levels	GCST90469620
rs1057258	3.00E-14	INPP5D	Eosinophil count	GCST90056180
rs17863796	3.00E-14	UGT1A6,UGT1A3,UGT1A7,UGT1A8,UGT1A9,DNAJB3,UGT1A4,UGT1A10,UGT1A5	Total bilirubin levels	GCST90019521
rs10168416	3.00E-14	UGT1A8,UGT1A7,UGT1A9,UGT1A10	Biliverdin levels in elite athletes	GCST90133797
rs11892031	3.00E-14	UGT1A8,UGT1A10	P-cresol glucuronide levels	GCST90200003
rs55891750	3.00E-14	UGT1A9,UGT1A7,UGT1A5,UGT1A3,UGT1A8,UGT1A10,UGT1A6,UGT1A4	Total bilirubin levels	GCST90429160
rs2305538	3.00E-14	DGKD	creatinine (maximum, inv-norm transformed)	GCST90475226
rs1057258	3.00E-14	INPP5D	eosinophil (absolute count, maximum, inv-norm transformed)	GCST90475288
rs72551330	3.00E-14	UGT1A10,UGT1A8,UGT1A9	Indoleacetate levels	GCST90103020
rs34916116	3.00E-14	UGT1A10,UGT1A4,UGT1A8,UGT1A6,UGT1A7,UGT1A9,UGT1A3,UGT1A5	P-cresol-glucuronide levels	GCST90103136
rs6431630	3.00E-14	UGT1A9,UGT1A4,UGT1A5,UGT1A6,UGT1A10,UGT1A1,UGT1A7,UGT1A8,UGT1A3	Low-density lipoprotein levels	GCST90662892
rs706848	4.00E-14	DGKD	Bilirubin (z,z) levels	GCST90102892
rs550363518	4.00E-14	MSL3B,TRPM8	Total bilirubin levels (UKB data field 30840)	GCST90468104
rs10210302	5.00E-14	ATG16L1	Crohn's disease	GCST000042
rs114630474	5.00E-14	MSL3B	Direct bilirubin levels	GCST90019505
rs72551330	5.00E-14	UGT1A10,UGT1A8,UGT1A9	4-oxo-retinoic acid levels	GCST90199894
rs4663983	5.00E-14	MSL3B,TRPM8	Medication use (antimigraine preparations)	GCST90018997
rs838705	6.00E-14	DGKD	Calcium levels	GCST90018731

rs62195072	6.00E-14	DGKD,SAG	Sex hormone-binding globulin levels adjusted for BMI	GCST90012108
rs117809958	6.00E-14	ATG16L1	Type 2 diabetes	GCST90018926
rs2305538	6.00E-14	DGKD	Blood urea nitrogen (BUN, mean, inv-norm transformed)	GCST90475165
rs2305538	6.00E-14	DGKD	creatinine (minimum, inv-norm transformed)	GCST90475232
rs149247216	7.00E-14	UGT1A11P,UGT1A8	Neonatal jaundice (paternal effect)	GCST90435147
rs1057258	7.00E-14	INPP5D	Immature reticulocyte fraction (UKB data field 30280)	GCST90468079
rs62195072	8.00E-14	DGKD,SAG	Sex hormone-binding globulin levels	GCST90012111
rs1057258	8.00E-14	INPP5D	eosinophil (fraction, minimum, inv-norm transformed)	GCST90475303
rs838718	8.00E-14	DGKD	Hemoglobin levels	GCST90662903
rs6741751	9.00E-14	TRPM8	Migraine	GCST002081
rs114243364	9.00E-14	TRPM8,SPP2	Total bilirubin levels	GCST90019521
rs6757418	9.00E-14	ATG16L1	Direct bilirubin levels	GCST90019505
rs76384978	9.00E-14	ATG16L1	Direct bilirubin levels	GCST90019505
rs2241880	1.00E-13	ATG16L1	Crohn's disease	GCST000023
rs143661480	1.00E-13	USP40	Direct bilirubin levels	GCST90019505
rs4663971	1.00E-13	UGT1A3,UGT1A9,UGT1A5,UGT1A4,UGT1A10,UGT1A6,UGT1A1,UGT1A8,UGT1A7	Direct bilirubin levels	GCST90429161
rs887829	1.00E-13	UGT1A5,UGT1A9,UGT1A10,UGT1A7,UGT1A4,UGT1A8,UGT1A3,UGT1A6	Bilirubin levels in chronic kidney disease	GCST90258099
rs17864701	1.00E-13	UGT1A8,UGT1A3,UGT1A6,UGT1A5,UGT1A9,UGT1A10,UGT1A4,UGT1A7	a sulfate of piperine metabolite C18H21NO3 (1)* levels in chronic kidney d	GCST90266016
rs34983651	1.00E-13	UGT1A6,UGT1A4,UGT1A8,UGT1A5,UGT1A7,UGT1A3,UGT1A9,UGT1A10	Retinol dehydrogenase 16 levels (RDH16.12881.17.3)	GCST90242643
rs1057258	2.00E-13	INPP5D	Sum eosinophil basophil counts	GCST004624
rs7571337	2.00E-13	UGT1A10,UGT1A8	Total bilirubin levels x insomnia interaction	GCST90026657
rs7571337	2.00E-13	UGT1A10,UGT1A8	Direct bilirubin levels x insomnia interaction	GCST90026656
rs4148325	2.00E-13	UGT1A9,UGT1A7,UGT1A3,UGT1A5,UGT1A8,UGT1A1,UGT1A4,UGT1A10,UGT1A6	Bilirubin (E,E) levels	GCST90128873
rs6760630	2.00E-13	TRPM8,MSL3B	Headache or migraine	GCST90267554
rs6747843	2.00E-13	UGT1A8,UGT1A10,UGT1A9,UGT1A3,UGT1A6,UGT1A5,UGT1A7,UGT1A4	sulfate of piperine metabolite C18H21NO3 (1)* levels in chronic kidney di	GCST90266017
rs199815566	2.00E-13	TRPM8	Total bilirubin levels (UKB data field 30840)	GCST90468104
rs143487779	2.00E-13	UGT1A9,UGT1A8,UGT1A10	Total bilirubin levels (UKB data field 30840)	GCST90468104
rs2241878	3.00E-13	ATG16L1	Liver enzyme levels (alkaline phosphatase)	GCST90013406
rs9287650	3.00E-13	TRPM8,SPP2	Direct bilirubin levels	GCST90019505
rs35754645	3.00E-13	UGT1A9,UGT1A8,UGT1A3,UGT1A5,UGT1A6,UGT1A7,UGT1A4,UGT1A10	Total cholesterol levels	GCST006614
rs4663983	3.00E-13	MSL3B,TRPM8	Migraine	GCST90000016
rs11893247	3.00E-13	UGT1A8,UGT1A10	4-oxo-retinoic acid levels	GCST90199894
rs2011425	3.00E-13	UGT1A8,UGT1A6,UGT1A10,UGT1A9,UGT1A5,UGT1A4,UGT1A7	Pregnenetriol disulfate levels	GCST90140162
rs117809958	4.00E-13	ATG16L1	Type 2 diabetes	GCST90132185
rs2305538	4.00E-13	DGKD	Estimated glomerular filtration rate (creatinine)	GCST90100220
rs2885297	4.00E-13	MROH2A	Bilirubin (z,z) levels	GCST90102892
rs887829	5.00E-13	UGT1A5,UGT1A9,UGT1A10,UGT1A7,UGT1A4,UGT1A8,UGT1A3,UGT1A6	Cholinesterase levels	GCST90247015
rs13035837	5.00E-13	DGKD	White blood cell count	GCST90662906
rs887829	6.00E-13	UGT1A5,UGT1A9,UGT1A10,UGT1A7,UGT1A4,UGT1A8,UGT1A3,UGT1A6	Serum metabolite levels	GCST002388
rs111741722	6.00E-13	UGT1A3,UGT1A10,UGT1A7,UGT1A8,UGT1A5,UGT1A4,UGT1A9,UGT1A6	Blood protein levels	GCST006585
rs1976391	6.00E-13	UGT1A9,UGT1A6,UGT1A5,UGT1A3,UGT1A8,UGT1A10,UGT1A7,UGT1A4	Cerebrospinal fluid bilirubin (E,E)* levels	GCST90318036
rs10179091	6.00E-13	UGT1A4,UGT1A9,UGT1A5,UGT1A8,UGT1A7,UGT1A3,UGT1A10,UGT1A6	Direct bilirubin levels	GCST90429161
rs11689634	6.00E-13	DGKD	Bilirubin (z,z) levels	GCST90102892
rs6431633	6.00E-13	MROH2A	Bilirubin (z,z) levels	GCST90102892
rs765651860	6.00E-13	TRPM8,MSL3B	Total bilirubin levels (UKB data field 30840)	GCST90468104
rs546193454	6.00E-13	MSL3B,HJURP	Total bilirubin levels (UKB data field 30840)	GCST90468104
rs62192317	7.00E-13	TRPM8,MSL3B	Direct bilirubin levels	GCST90019505
rs2012736	7.00E-13	UGT1A9,UGT1A8,UGT1A6,UGT1A7,UGT1A10,UGT1A5	Vitamin D levels or COVID-19 (MTAG)	GCST90270936
rs6742078	7.00E-13	UGT1A1,UGT1A9,UGT1A4,UGT1A6,UGT1A5,UGT1A10,UGT1A7,UGT1A3,UGT1A8	Bilirubin concentration in dolutegravir-treated HIV infection	GCST90243991
rs11563251	8.00E-13	UGT1A10,UGT1A8,UGT1A3,UGT1A6,UGT1A5,UGT1A4,UGT1A9,UGT1A7,UGT1A1	Low density lipoprotein cholesterol levels	GCST010204
rs2305538	8.00E-13	DGKD	Blood urea nitrogen (BUN, mean, inv-norm transformed)	GCST90479525
rs3732218	8.00E-13	UGT1A6,UGT1A7,UGT1A10,UGT1A9,UGT1A5,UGT1A8	Plasma norclozapine levels in treatment-resistant schizophrenia	GCST90239837
rs557265313	8.00E-13	TRPM8	Cerebral aneurysm (PheCode 433.5)	GCST90480194
rs10166942	1.00E-12	MSL3B,TRPM8	Migraine	GCST001563
rs2241880	1.00E-12	ATG16L1	Crohn's disease	GCST001438
rs17862875	1.00E-12	UGT1A3,UGT1A5,UGT1A9,UGT1A4,UGT1A10,UGT1A7,UGT1A8,UGT1A6	Serum levels of protein MCTS1	GCST90087020
rs6759896	1.00E-12	ATG16L1,SAG	Serum alkaline phosphatase levels	GCST90018942
rs4300821	1.00E-12	UGT1A8	Total bilirubin levels	GCST90429160
rs2289473	1.00E-12	ATG16L1	Bilirubin (z,z) levels	GCST90102892
rs1024608	2.00E-12	TRPM8	Total bilirubin levels	GCST90019521
rs56201966	2.00E-12	DGKD	Platelet count during third trimester of pregnancy	GCST90302226
rs140874442	2.00E-12	HJURP	Direct bilirubin levels	GCST90019505
rs17862875	2.00E-12	UGT1A3,UGT1A5,UGT1A9,UGT1A4,UGT1A10,UGT1A7,UGT1A8,UGT1A6	Serum levels of protein TMEM52B	GCST90090819
rs10166942	2.00E-12	MSL3B,TRPM8	Migraine	GCST90429187
rs2305538	2.00E-12	DGKD	Blood urea nitrogen (BUN, maximum, inv-norm transformed)	GCST90475162
rs2305538	2.00E-12	DGKD	stimated glomerular filtration rate (eGFR, maximum, inv-norm transforme	GCST90475280
rs13410335	2.00E-12	UGT1A4,UGT1A5,UGT1A10,UGT1A8,UGT1A7,UGT1A9,UGT1A6,UGT1A3	X-11880 levels	GCST90200487
rs887829	2.00E-12	UGT1A5,UGT1A9,UGT1A10,UGT1A7,UGT1A4,UGT1A8,UGT1A3,UGT1A6	Other chronic nonalcoholic liver disease (PheCode 571.5)	GCST90480338
rs13002399	3.00E-12	USP40,UGT1A12P	Serum phosphate levels	GCST90019516
rs192970075	3.00E-12	DGKD,USP40	Coronary artery calcium score	GCST90104132
rs3732218	3.00E-12	UGT1A6,UGT1A7,UGT1A10,UGT1A9,UGT1A5,UGT1A8	Plasma clozapine levels in treatment-resistant schizophrenia	GCST90239836
rs3796092	3.00E-12	USP40	X-21448 levels	GCST90103303
rs17863787	3.00E-12	UGT1A7,UGT1A6,UGT1A9,UGT1A10,UGT1A8	Nuclear receptor subfamily 1 group D member 2 levels (NR1D2.12885.42.3	GCST90242145
rs11568318	4.00E-12	UGT1A9,UGT1A4,UGT1A5,UGT1A10,UGT1A6,UGT1A3,UGT1A8,UGT1A7	Apolipoprotein B levels	GCST010243
rs4148325	4.00E-12	UGT1A9,UGT1A7,UGT1A3,UGT1A5,UGT1A8,UGT1A1,UGT1A4,UGT1A10,UGT1A6	Serum metabolite concentrations in chronic kidney disease	GCST012353
rs685139	4.00E-12	HJURP	Headache or migraine	GCST90267554
rs11695484	4.00E-12	UGT1A5,UGT1A3,UGT1A8,UGT1A4,UGT1A6,UGT1A10,UGT1A9,UGT1A7	sulfate of piperine metabolite C16H19NO3 (2)* levels in chronic kidney di	GCST90266009
rs2011425	4.00E-12	UGT1A8,UGT1A6,UGT1A10,UGT1A9,UGT1A5,UGT1A4,UGT1A7	X-21441 levels	GCST90140524
rs887829	4.00E-12	UGT1A5,UGT1A9,UGT1A10,UGT1A7,UGT1A4,UGT1A8,UGT1A3,UGT1A6	Chronic liver disease and cirrhosis (PheCode 571)	GCST90480341
rs60485593	5.00E-12	DGKD	Plateletcrit	GCST004607
rs17868336	5.00E-12	UGT1A3,UGT1A7,UGT1A4,UGT1A8,UGT1A10,UGT1A5,UGT1A6,UGT1A9	Apolipoprotein B levels	GCST90019496
rs183383266	5.00E-12	SAG	Direct bilirubin levels	GCST90019505
rs72551330	5.00E-12	UGT1A10,UGT1A8,UGT1A9	Indole-3-carboxylate levels	GCST90199877
rs554460691	5.00E-12	ATG16L1	Certain early complications of trauma or procedure (PheCode 958)	GCST90480631
rs10166942	6.00E-12	MSL3B,TRPM8	Migraine	GCST001105
rs4663540	6.00E-12	DGKD,SAG	Hemoglobin concentration	GCST90002310
rs143487779	6.00E-12	UGT1A9,UGT1A8,UGT1A10	Direct bilirubin levels	GCST90019505
rs3755322	6.00E-12	UGT1A5,UGT1A7,UGT1A10,UGT1A6,UGT1A9,UGT1A8	Vitamin D insufficiency	GCST90020244
rs7567468	6.00E-12	UGT1A7,UGT1A6,UGT1A9,UGT1A10,UGT1A3,UGT1A5,UGT1A8,UGT1A4	Serum levels of protein CMPK1	GCST90090121
rs2305538	6.00E-12	DGKD	Blood urea nitrogen (BUN, maximum, inv-norm transformed)	GCST90479524
rs4402757	6.00E-12	UGT1A11P,UGT1A8	X-21339 levels	GCST90245667
rs887829	7.00E-12	UGT1A5,UGT1A9,UGT1A10,UGT1A7,UGT1A4,UGT1A8,UGT1A3,UGT1A6	Bilirubin levels in tenofovir-treated HIV infection	GCST006074
rs1875263	7.00E-12	UGT1A5,UGT1A6,UGT1A9,UGT1A8,UGT1A10,UGT1A7	Total bilirubin levels in HIV-1 infection	GCST002745

rs137886124	7.00E-12	SAG	Direct bilirubin levels	GCST90019505
rs36075906	7.00E-12	USP40	Bilirubin levels	GCST010048
rs562746713	7.00E-12	UGT1A3,UGT1A10,UGT1A9,UGT1A8,UGT1A6,UGT1A5,UGT1A4,UGT1A7	Spondylolisthesis, congenital (PheCode 754.2)	GCST90480566
rs28953469	7.00E-12	TRPM8	Phosphate levels (UKB data field 30810)	GCST90468094
rs115999431	7.00E-12	USP40	Total bilirubin levels (UKB data field 30840)	GCST90468104
rs11674391	8.00E-12	TRPM8,MSL3B	Direct bilirubin levels	GCST90019505
rs4148325	1.00E-11	UGT1A9,UGT1A7,UGT1A3,UGT1A5,UGT1A8,UGT1A1,UGT1A4,UGT1A10,UGT1A6	Bilirubin levels in tenofovir-treated HIV infection	GCST006074
rs17868336	1.00E-11	UGT1A3,UGT1A7,UGT1A4,UGT1A8,UGT1A10,UGT1A5,UGT1A6,UGT1A9	Total cholesterol levels	GCST90019501
rs11563210	1.00E-11	TRPM8	Direct bilirubin levels	GCST90019505
rs62192905	1.00E-11	ATG16L1	Direct bilirubin levels	GCST90019505
rs28626554	1.00E-11	MROH2A	Direct bilirubin levels	GCST90019505
rs10196794	1.00E-11	ATG16L1	Crohn's disease	GCST90319897
rs111741722	1.00E-11	UGT1A3,UGT1A10,UGT1A7,UGT1A8,UGT1A5,UGT1A4,UGT1A9,UGT1A6	Blood protein levels	GCST006585
rs117809958	1.00E-11	ATG16L1	Medication use (drugs used in diabetes)	GCST90018761
rs62195072	1.00E-11	DGKD,SAG	Sex hormone-binding globulin levels	GCST90012109
rs887829	1.00E-11	UGT1A5,UGT1A9,UGT1A10,UGT1A7,UGT1A4,UGT1A8,UGT1A3,UGT1A6	Serum metabolite levels	GCST012020
rs2011425	1.00E-11	UGT1A8,UGT1A6,UGT1A10,UGT1A9,UGT1A5,UGT1A4,UGT1A7	Free testosterone levels	GCST90027087
rs34983651	1.00E-11	UGT1A6,UGT1A4,UGT1A8,UGT1A5,UGT1A7,UGT1A3,UGT1A9,UGT1A10	Thyroxine levels	GCST90103219
rs28898590	1.00E-11	UGT1A7,UGT1A9,UGT1A6,UGT1A8,UGT1A10	X-21339 levels	GCST90103297
rs117809958	1.00E-11	ATG16L1	Medication use (drugs used in diabetes)	GCST90018981
rs13005285	1.00E-11	ATG16L1	Inflammatory bowel disease (MTAG)	GCST90503485
rs11673726	1.00E-11	UGT1A9,UGT1A8,UGT1A6,UGT1A10,UGT1A3,UGT1A5,UGT1A7,UGT1A4	Gamma glutamyl transferase levels	GCST90662899
rs34013402	1.00E-11	USP40	Lymphocyte count (UKB data field 30120)	GCST90468082
rs887829	1.00E-11	UGT1A5,UGT1A9,UGT1A10,UGT1A7,UGT1A4,UGT1A8,UGT1A3,UGT1A6	APOL1 protein levels	GCST90468341
rs838718	2.00E-11	DGKD	Hematocrit	GCST90002304
rs4148323	2.00E-11	UGT1A10,UGT1A6,UGT1A9,UGT1A4,UGT1A8,UGT1A1,UGT1A7,UGT1A3,UGT1A5	Total bilirubin levels	GCST90019521
rs17865681	2.00E-11	TRPM8	Total bilirubin levels	GCST90019521
rs17865681	2.00E-11	TRPM8	Direct bilirubin levels	GCST90019505
rs4663983	2.00E-11	MSL3B,TRPM8	Medication use (anilides)	GCST007938
rs117809958	2.00E-11	ATG16L1	Type 2 diabetes	GCST90132183
rs55891750	2.00E-11	UGT1A9,UGT1A7,UGT1A5,UGT1A3,UGT1A8,UGT1A10,UGT1A6,UGT1A4	Direct bilirubin levels	GCST90429161
rs2602376	2.00E-11	UGT1A8,UGT1A10,UGT1A9	Cholelithiasis (UKB data field 20002_1162)	GCST90432140
rs14243	2.00E-11	INPP5D	corpuscular hemoglobin concentration (MCHC, maximum, inv-norm transfo	GCST90479669
rs6722076	2.00E-11	UGT1A4,UGT1A10,UGT1A8,UGT1A5,UGT1A3,UGT1A6,UGT1A9,UGT1A7	Plasma xanthurenate levels in chronic kidney disease	GCST90266868
rs1105880	2.00E-11	UGT1A6,UGT1A7,UGT1A9,UGT1A10,UGT1A8	Sulfate of piperine metabolite C18H21NO3 (1) levels	GCST90140867
rs13003198	3.00E-11	DGKD,SAG	Estimated glomerular filtration rate	GCST008058
rs838718	3.00E-11	DGKD	Hematocrit	GCST90002308
rs62195072	3.00E-11	DGKD,SAG	Sex hormone-binding globulin levels	GCST90239821
rs6715829	3.00E-11	UGT1A9,UGT1A8,UGT1A6,UGT1A7,UGT1A10	Serum metabolite levels	GCST012020
rs6744284	3.00E-11	UGT1A5,UGT1A10,UGT1A6,UGT1A9,UGT1A8,UGT1A7	e sulfate of piperine metabolite C18H21NO3 (2)* levels in chronic kidney di	GCST90266018
rs6742078	4.00E-11	UGT1A1,UGT1A9,UGT1A4,UGT1A6,UGT1A5,UGT1A10,UGT1A7,UGT1A3,UGT1A8	Bilirubin levels in tenofovir-treated HIV infection	GCST006074
rs838718	4.00E-11	DGKD	Hemoglobin concentration	GCST90002314
rs7567229	4.00E-11	UGT1A6,UGT1A9,UGT1A7,UGT1A10,UGT1A8	LDL cholesterol	GCST006612
rs13003198	4.00E-11	DGKD,SAG	Kidney stones	GCST009598
rs4148325	4.00E-11	UGT1A9,UGT1A7,UGT1A3,UGT1A5,UGT1A8,UGT1A1,UGT1A4,UGT1A10,UGT1A6	Histidine betaine (hercynine) levels	GCST90199927
rs113033181	4.00E-11	UGT1A8,UGT1A11P	Cholelithiasis with other cholecystitis (PheCode 574.12)	GCST90480350
rs28898590	5.00E-11	UGT1A7,UGT1A9,UGT1A6,UGT1A8,UGT1A10	Sex hormone-binding globulin levels adjusted for BMI	GCST90012110
rs2011425	5.00E-11	UGT1A8,UGT1A6,UGT1A10,UGT1A9,UGT1A5,UGT1A4,UGT1A7	Bioavailable testosterone levels	GCST90027083
rs17864662	5.00E-11	UGT1A11P,UGT1A12P	Bilirubin (E,Z or Z,E) levels	GCST90102891
rs2971871	5.00E-11	DGKD	JT interval	GCST90179157
rs6431558	6.00E-11	UGT1A8	Direct bilirubin levels x insomnia interaction	GCST90026656
rs6431558	6.00E-11	UGT1A8	Total bilirubin levels x insomnia interaction	GCST90026657
rs10166942	6.00E-11	MSL3B,TRPM8	Pain (pleiotropy)	GCST90104574
rs2242100	6.00E-11	DGKD	X-21448 levels	GCST90103303
rs11563251	7.00E-11	UGT1A10,UGT1A8,UGT1A3,UGT1A6,UGT1A5,UGT1A4,UGT1A9,UGT1A7,UGT1A1	Body fat percentage and LDL-C (pairwise)	GCST011343
rs2879924	7.00E-11	SAG	Serum alkaline phosphatase levels	GCST90019494
rs10929302	7.00E-11	UGT1A4,UGT1A3,UGT1A10,UGT1A8,UGT1A5,UGT1A6,UGT1A7,UGT1A9	Total cholesterol levels	GCST90019501
rs2011425	7.00E-11	UGT1A8,UGT1A6,UGT1A10,UGT1A9,UGT1A5,UGT1A4,UGT1A7	Total testosterone levels	GCST90012112
rs36001488	8.00E-11	ATG16L1	Pediatric autoimmune diseases	GCST003097
rs1550532	8.00E-11	DGKD	Calcium levels	GCST002201
rs17864771	8.00E-11	TRPM8	Direct bilirubin levels	GCST90019505
rs1550532	8.00E-11	DGKD	Calcium levels	GCST012398
rs36075906	8.00E-11	USP40	Albumin levels	GCST90662901
rs4148324	9.00E-11	UGT1A3,UGT1A10,UGT1A4,UGT1A6,UGT1A7,UGT1A8,UGT1A9,UGT1A1,UGT1A5	Bilirubin levels in tenofovir-treated HIV infection	GCST006074
rs6741751	9.00E-11	TRPM8	Migraine without aura	GCST002078
rs2070959	9.00E-11	UGT1A9,UGT1A7,UGT1A10,UGT1A8,UGT1A6	Gallstone disease	GCST007209
rs56263467	9.00E-11	DGKD	Platelet count	GCST90278640
rs2741042	9.00E-11	UGT1A8,UGT1A10	Androstenediol levels	GCST90101914
rs929596	1.00E-10	UGT1A3,UGT1A5,UGT1A4,UGT1A6,UGT1A9,UGT1A8,UGT1A10,UGT1A1,UGT1A7	Bilirubin levels in tenofovir-treated HIV infection	GCST006074
rs3771341	1.00E-10	UGT1A6,UGT1A10,UGT1A4,UGT1A5,UGT1A8,UGT1A1,UGT1A7,UGT1A9,UGT1A3	Bilirubin levels in tenofovir-treated HIV infection	GCST006074
rs28898617	1.00E-10	UGT1A9,UGT1A7,UGT1A4,UGT1A10,UGT1A5,UGT1A8,UGT1A6,UGT1A3	Total bilirubin levels	GCST005980
rs1057258	1.00E-10	INPP5D	Allergic disease (asthma, hay fever or eczema)	GCST005038
rs13401703	1.00E-10	USP40,UGT1A12P	Adolescent idiopathic scoliosis	GCST006287
rs10490012	1.00E-10	TRPM8	Bilirubin levels	GCST008817
rs6744284	1.00E-10	UGT1A5,UGT1A10,UGT1A6,UGT1A9,UGT1A8,UGT1A7	Bilirubin (z,z) levels	GCST90026002
rs74669452	1.00E-10	UGT1A9,UGT1A8,UGT1A6,UGT1A5,UGT1A10,UGT1A7	Direct bilirubin levels	GCST90019505
rs11891546	1.00E-10	SAG	Inflammatory bowel disease (MTAG)	GCST90503485
rs17864771	2.00E-10	TRPM8	Total bilirubin levels	GCST90019521
rs72551330	2.00E-10	UGT1A10,UGT1A8,UGT1A9	X-24588 levels	GCST90200649
rs1985366	2.00E-10	MSL3B,TRPM8	Medication use (anilides)	GCST90018996
rs6805526,rs4663396	2.00E-10	IQCJ-SCHIP1, SCHIP1 x ATG16L1	Total PHF-tau (SNP x SNP interaction)	GCST010340
rs34352510	3.00E-10	UGT1A5,UGT1A4,UGT1A10,UGT1A9,UGT1A8,UGT1A7,UGT1A3,UGT1A6	Bilirubin levels in tenofovir-treated HIV infection	GCST006074
rs2011425	3.00E-10	UGT1A8,UGT1A6,UGT1A10,UGT1A9,UGT1A5,UGT1A4,UGT1A7	Pregnenetriol disulfate levels	GCST90200238
rs13418066	3.00E-10	ATG16L1	Serum alkaline phosphatase levels	GCST90011900
rs4663980	3.00E-10	MSL3B,TRPM8	Headache or migraine	GCST90267554
rs2011425	3.00E-10	UGT1A8,UGT1A6,UGT1A10,UGT1A9,UGT1A5,UGT1A4,UGT1A7	Total testosterone levels	GCST90027095
rs71421697	3.00E-10	DGKD	Serum total protein levels	GCST90018976
rs17868336	4.00E-10	UGT1A3,UGT1A7,UGT1A4,UGT1A8,UGT1A10,UGT1A5,UGT1A6,UGT1A9	Low density lipoprotein cholesterol levels	GCST90019512
rs111809359	4.00E-10	MROH2A	Direct bilirubin levels	GCST90019505
rs2971857	4.00E-10	DGKD	Appendicular lean mass	GCST90000025
rs838735	4.00E-10	DGKD	Type 2 diabetes	GCST010555
rs28898568	4.00E-10	UGT1A8,UGT1A7,UGT1A9,UGT1A10	Total bilirubin levels	GCST90429160
rs10179094	5.00E-10	UGT1A7,UGT1A9,UGT1A8,UGT1A10	5-hydroxyindole sulfate levels	GCST90200050

rs566529	5.00E-10	HJURP	Headache or migraine	GCST90267554
rs34622615	6.00E-10	UGT1A6,UGT1A4,UGT1A10,UGT1A5,UGT1A7,UGT1A8,UGT1A3,UGT1A9,DNAJB3	Total cholesterol levels	GCST90019501
rs145966524	6.00E-10	DGKD	Femur bone mineral density x serum urate levels interaction	GCST012490
rs114644056	6.00E-10	USP40	Femur bone mineral density x serum urate levels interaction	GCST012490
rs35555682	6.00E-10	UGT1A10,UGT1A8	Total bilirubin levels x insomnia interaction	GCST90026657
rs35555682	6.00E-10	UGT1A10,UGT1A8	Direct bilirubin levels x insomnia interaction	GCST90026656
rs71423603	6.00E-10	USP40	Platelet count during second trimester of pregnancy	GCST90302225
rs6722076	6.00E-10	UGT1A4,UGT1A10,UGT1A8,UGT1A5,UGT1A3,UGT1A6,UGT1A9,UGT1A7	Thyroxine levels	GCST90200293
rs838718	6.00E-10	DGKD	Hematocrit	GCST90662904
rs11568318	7.00E-10	UGT1A9,UGT1A4,UGT1A5,UGT1A10,UGT1A6,UGT1A3,UGT1A8,UGT1A7	LDL cholesterol levels	GCST010245
rs4663335	7.00E-10	MROH2A	Total cholesterol levels	GCST90019501
rs4663327	7.00E-10	UGT1A7,UGT1A10,UGT1A6,UGT1A8,UGT1A9	Total testosterone levels	GCST90027095
rs6724624	1.00E-09	MSL3B,TRPM8	Migraine without aura	GCST003721
rs10179091	1.00E-09	UGT1A4,UGT1A9,UGT1A5,UGT1A8,UGT1A7,UGT1A3,UGT1A10,UGT1A6	Bilirubin levels in tenofovir-treated HIV infection	GCST006074
rs11563251	1.00E-09	UGT1A10,UGT1A8,UGT1A3,UGT1A6,UGT1A5,UGT1A4,UGT1A9,UGT1A7,UGT1A1	Cholesterol, total	GCST002221
rs2741027	1.00E-09	UGT1A11P,UGT1A8	Serum bilirubin levels in metabolic syndrome	GCST007018
rs146371325	1.00E-09	USP40	Total bilirubin levels	GCST90019521
rs140912561	1.00E-09	HJURP	Total bilirubin levels	GCST90019521
rs114243364	1.00E-09	TRPM8,SPP2	Direct bilirubin levels	GCST90019505
rs17862932	1.00E-09	TRPM8	Direct bilirubin levels	GCST90019505
rs28946889	1.00E-09	UGT1A1,UGT1A7,UGT1A9,UGT1A3,UGT1A6,UGT1A10,UGT1A5,UGT1A4,UGT1A8	Cholelithiasis	GCST90018599
rs10180090	1.00E-09	UGT1A10,UGT1A7,UGT1A9,UGT1A6,UGT1A8	Gamma glutamyl transpeptidase	GCST90018954
rs10182651	1.00E-09	USP40,UGT1A12P	Rate of cognitive decline in Alzheimer's disease	GCST010567
rs148015174	1.00E-09	ATG16L1	Prostate-specific antigen levels	GCST90461907
rs13411660	1.00E-09	ATG16L1,SAG	Crohn's disease	GCST90446793
rs4148323	1.00E-09	UGT1A10,UGT1A6,UGT1A9,UGT1A4,UGT1A8,UGT1A1,UGT1A7,UGT1A3,UGT1A5	Other perinatal conditions of fetus or newborn (PheCode 656)	GCST90651319
rs10929302	2.00E-09	UGT1A4,UGT1A3,UGT1A10,UGT1A8,UGT1A5,UGT1A6,UGT1A7,UGT1A9	Bilirubin levels in tenofovir-treated HIV infection	GCST006074
rs6747843	2.00E-09	UGT1A8,UGT1A10,UGT1A9,UGT1A3,UGT1A6,UGT1A5,UGT1A7,UGT1A4	Bilirubin levels in tenofovir-treated HIV infection	GCST006074
rs6714634	2.00E-09	UGT1A3,UGT1A6,UGT1A4,UGT1A9,UGT1A8,UGT1A7,UGT1A5,UGT1A10	Bilirubin levels in tenofovir-treated HIV infection	GCST006074
rs2885296	2.00E-09	UGT1A5,UGT1A3,UGT1A7,UGT1A4,UGT1A10,UGT1A9,UGT1A6,UGT1A8	Bilirubin levels in tenofovir-treated HIV infection	GCST006074
rs2741012	2.00E-09	UGT1A12P,UGT1A11P	Total bilirubin levels in HIV-1 infection	GCST002745
rs838563	2.00E-09	USP40	Calcium levels	GCST90019500
rs74669452	2.00E-09	UGT1A9,UGT1A8,UGT1A6,UGT1A5,UGT1A10,UGT1A7	Vitamin D levels	GCST90019526
rs114644056	2.00E-09	USP40	Femur bone mineral density x serum urate levels interaction	GCST012490
rs838720	2.00E-09	DGKD	Type 2 diabetes	GCST007847
rs28969973	2.00E-09	UGT1A8	Total bilirubin levels	GCST90429160
rs28899170	2.00E-09	UGT1A8,UGT1A7,UGT1A10,UGT1A9,UGT1A6	ne abundance (class Fusicatenibacter sp. 3 (at 1 year) x Spring Birth (Mar-M	GCST90568956
rs2971872	2.00E-09	DGKD	JT interval	GCST90179158
rs2971870	2.00E-09	DGKD	QT interval	GCST90179153
rs566529	3.00E-09	HJURP	Migraine	GCST003720
rs11695484	3.00E-09	UGT1A5,UGT1A3,UGT1A8,UGT1A4,UGT1A6,UGT1A10,UGT1A9,UGT1A7	Bilirubin levels in tenofovir-treated HIV infection	GCST006074
rs36075906	3.00E-09	USP40	Calcium levels	GCST90019500
rs2011425	3.00E-09	UGT1A8,UGT1A6,UGT1A10,UGT1A9,UGT1A5,UGT1A4,UGT1A7	Free androgen index	GCST90239823
rs838717	3.00E-09	DGKD	Kidney stone disease	GCST90428894
rs72976321	3.00E-09	ATG16L1	Type 2 diabetes	GCST90492734
rs6431558	3.00E-09	UGT1A8	Triglyceride levels	GCST90662893
rs34891595	3.00E-09	SAG	Systolic blood pressure	GCST90662908
rs11673726	4.00E-09	UGT1A9,UGT1A8,UGT1A6,UGT1A10,UGT1A3,UGT1A5,UGT1A7,UGT1A4	Bilirubin levels in tenofovir-treated HIV infection	GCST006074
rs62192912	4.00E-09	ATG16L1	Total cholesterol levels	GCST90239673
rs838720	4.00E-09	DGKD	Type 2 diabetes	GCST010553
rs111712352	4.00E-09	USP40	Bone mineral density mean	GCST90321120
rs17864701	5.00E-09	UGT1A8,UGT1A3,UGT1A6,UGT1A5,UGT1A9,UGT1A10,UGT1A4,UGT1A7	Bilirubin levels in tenofovir-treated HIV infection	GCST006074
rs4148325	5.00E-09	UGT1A9,UGT1A7,UGT1A3,UGT1A5,UGT1A8,UGT1A1,UGT1A4,UGT1A10,UGT1A6	Metabolite levels	GCST009391
rs3792109	5.00E-09	ATG16L1,SCARNA5	Crohn's disease	GCST001652
rs12988520	5.00E-09	UGT1A8,UGT1A7,UGT1A6,UGT1A9,UGT1A10	Serum bilirubin levels in metabolic syndrome	GCST007018
rs13014016	5.00E-09	DGKD,SAG	Hematocrit	GCST90018960
rs10803625	5.00E-09	SAG	Lymphocyte count	GCST90018962
rs2741046	5.00E-09	UGT1A8,UGT1A10	Bilirubin (E,Z or Z,E) levels	GCST90128874
rs2012736	5.00E-09	UGT1A9,UGT1A8,UGT1A6,UGT1A7,UGT1A10,UGT1A5	Free testosterone levels	GCST90027087
rs2018985	5.00E-09	UGT1A5,UGT1A4,UGT1A10,UGT1A7,UGT1A3,UGT1A9,UGT1A8,UGT1A6	Jaundice (not of newborn) (PheCode 573.5)	GCST90651382
rs17862875	6.00E-09	UGT1A3,UGT1A5,UGT1A9,UGT1A4,UGT1A10,UGT1A7,UGT1A8,UGT1A6	Bilirubin levels in tenofovir-treated HIV infection	GCST006074
rs11888459	6.00E-09	UGT1A9,UGT1A6,UGT1A2P,UGT1A10,UGT1A3,UGT1A8,UGT1A5,UGT1A4,UGT1A7	Bilirubin levels in tenofovir-treated HIV infection	GCST006074
rs10178992	6.00E-09	UGT1A6,UGT1A4,UGT1A10,UGT1A7,UGT1A3,UGT1A5,UGT1A9,UGT1A8	Bilirubin levels in tenofovir-treated HIV infection	GCST006074
rs7604115	6.00E-09	UGT1A10,UGT1A8,UGT1A7,UGT1A6,UGT1A5,UGT1A9,UGT1A4,UGT1A3	Bilirubin levels in tenofovir-treated HIV infection	GCST006074
rs2924810	6.00E-09	DGKD,USP40	Self-reported math ability (MTAG)	GCST006569
rs2012736	6.00E-09	UGT1A9,UGT1A8,UGT1A6,UGT1A7,UGT1A10,UGT1A5	Bioavailable testosterone levels	GCST90027083
rs10929301	7.00E-09	UGT1A7,UGT1A3,UGT1A10,UGT1A8,UGT1A9,UGT1A6,UGT1A5,UGT1A4	Bilirubin levels in tenofovir-treated HIV infection	GCST006074
rs573393296	7.00E-09	UGT1A6,UGT1A10,UGT1A9,UGT1A8,UGT1A7	Free testosterone levels	GCST90239826
rs3732218	7.00E-09	UGT1A6,UGT1A7,UGT1A10,UGT1A9,UGT1A5,UGT1A8	N-desmethylclozapine concentration in schizophrenia	GCST010564
rs2108809	7.00E-09	TRPM8	Headache or migraine	GCST90267554
rs2011425	8.00E-09	UGT1A8,UGT1A6,UGT1A10,UGT1A9,UGT1A5,UGT1A4,UGT1A7	Plasma norclozapine levels in treatment-resistant schizophrenia	GCST007686
rs838714	8.00E-09	DGKD	Hemoglobin A1c levels	GCST90278632
rs573393296	9.00E-09	UGT1A6,UGT1A10,UGT1A9,UGT1A8,UGT1A7	Free androgen index	GCST90239824
rs6759355	9.00E-09	DGKD	Type 2 diabetes	GCST90013693
rs2603547	9.00E-09	USP40	QT interval	GCST90179154
rs838737	9.00E-09	DGKD	Glutamine levels	GCST90503746
rs6741751	1.00E-08	TRPM8	Migraine - clinic-based	GCST002079
rs145966524	1.00E-08	DGKD	Femur bone mineral density x serum urate levels interaction	GCST012490
rs6751673	1.00E-08	UGT1A9,UGT1A6,UGT1A8,UGT1A10,UGT1A7	Total bilirubin levels x insomnia interaction	GCST90026657
rs6751673	1.00E-08	UGT1A9,UGT1A6,UGT1A8,UGT1A10,UGT1A7	Direct bilirubin levels x insomnia interaction	GCST90026656
rs838718	1.00E-08	DGKD	Kidney stone disease	GCST90652507
rs2011425	1.00E-08	UGT1A8,UGT1A6,UGT1A10,UGT1A9,UGT1A5,UGT1A4,UGT1A7	Testosterone levels	GCST90104272
rs35203651	1.00E-08	UGT1A10,UGT1A3,UGT1A6,UGT1A4,UGT1A7,UGT1A8,UGT1A9,UGT1A5,UGT1A1	LDL cholesterol	GCST90018961
rs1976391	1.00E-08	UGT1A9,UGT1A6,UGT1A5,UGT1A3,UGT1A8,UGT1A10,UGT1A7,UGT1A4	Biliverdin levels	GCST90134443
rs1976391	1.00E-08	UGT1A9,UGT1A6,UGT1A5,UGT1A3,UGT1A8,UGT1A10,UGT1A7,UGT1A4	Biliverdin levels	GCST90134443
rs887829	1.00E-08	UGT1A5,UGT1A9,UGT1A10,UGT1A7,UGT1A4,UGT1A8,UGT1A3,UGT1A6	Biliverdin levels	GCST90134443
rs34847903	1.00E-08	DGKD	Hemoglobin levels	GCST90662873
rs14243	1.00E-08	INPP5D	Eosinophil count	GCST90662882
rs34339006	2.00E-08	DGKD	Glaucoma (primary open-angle)	GCST006065
rs11894379	2.00E-08	UGT1A6,UGT1A10,UGT1A9,UGT1A8,UGT1A7	Femur bone mineral density x serum urate levels interaction	GCST012490
rs838717	2.00E-08	DGKD	Kidney stones	GCST009599
rs2924808	2.00E-08	DGKD	Sex hormone-binding globulin levels adjusted for BMI	GCST90012106
rs1545524	2.00E-08	USP40	Highest math class taken (MTAG)	GCST006568

rs838718	2.00E-08	DGKD	Hemoglobin	GCST90018957
rs4148325	2.00E-08	UGT1A9,UGT1A7,UGT1A3,UGT1A5,UGT1A8,UGT1A1,UGT1A4,UGT1A10,UGT1A6	Biliverdin levels	GCST90128876
rs1057258	2.00E-08	INPP5D	Atopic dermatitis	GCST90297795
rs13385507	2.00E-08	TRPM8,SPP2	bial network clusters (Cyan (at 3 months) x Any Breastfeeding (3 Months) i	GCST90569274
rs6742078	2.00E-08	UGT1A1,UGT1A9,UGT1A4,UGT1A6,UGT1A5,UGT1A10,UGT1A7,UGT1A3,UGT1A8	Aldosterone levels	GCST90101911
rs4148325	2.00E-08	UGT1A9,UGT1A7,UGT1A3,UGT1A5,UGT1A8,UGT1A1,UGT1A4,UGT1A10,UGT1A6	Biliverdin levels	GCST90134443
rs10172939	2.00E-08	SAG	Inflammatory bowel disease (MTAG)	GCST90503485
rs13028996	2.00E-08	SAG	Inflammatory bowel disease (MTAG)	GCST90503485
rs3732218	2.00E-08	UGT1A6,UGT1A7,UGT1A10,UGT1A9,UGT1A5,UGT1A8	Testosterone levels	GCST90483486
rs838737	2.00E-08	DGKD	Glutamine levels	GCST90092818
rs7577262	3.00E-08	TRPM8,MSL3B	Blood pressure measurement (cold pressor test)	GCST002247
rs1076454	3.00E-08	MROH2A	Total protein levels x insomnia interaction	GCST90026658
rs838705	3.00E-08	DGKD	Lung function (FEV1/FVC)	GCST007080
rs6758317	3.00E-08	ATG16L1	Bilirubin levels	GCST010048
rs4663580	3.00E-08	DGKD	Bilirubin levels	GCST010048
rs3732218	3.00E-08	UGT1A6,UGT1A7,UGT1A10,UGT1A9,UGT1A5,UGT1A8	Bioavailable testosterone levels	GCST90012102
rs13410192	3.00E-08	DGKD	Sex hormone-binding globulin levels	GCST90239822
rs2741031	3.00E-08	UGT1A8	Direct bilirubin levels	GCST90429161
rs11563251	4.00E-08	UGT1A10,UGT1A8,UGT1A3,UGT1A6,UGT1A5,UGT1A4,UGT1A9,UGT1A7,UGT1A1	LDL cholesterol levels	GCST004233
rs838721	4.00E-08	DGKD	Total body bone mineral density	GCST005348
rs2361502	4.00E-08	MROH2A	Serum bilirubin levels in metabolic syndrome	GCST007018
rs887829	4.00E-08	UGT1A5,UGT1A9,UGT1A10,UGT1A7,UGT1A4,UGT1A8,UGT1A3,UGT1A6	AUC of 2-hydroxy atorvastatin lactone	GCST90624196
rs6742078	4.00E-08	UGT1A1,UGT1A9,UGT1A4,UGT1A6,UGT1A5,UGT1A10,UGT1A7,UGT1A3,UGT1A8	AUC of 2-hydroxy atorvastatin lactone	GCST90624196
rs4148325	4.00E-08	UGT1A9,UGT1A7,UGT1A3,UGT1A5,UGT1A8,UGT1A1,UGT1A4,UGT1A10,UGT1A6	AUC of 2-hydroxy atorvastatin lactone	GCST90624196
rs17868301	4.00E-08	UGT1A11P,UGT1A12P	Triglyceride levels	GCST90239661
rs12996460	4.00E-08	ATG16L1	bial network clusters (Cyan (at 3 months) x Any Breastfeeding (3 Months) i	GCST90569274
rs2361502	4.00E-08	MROH2A	X-12231 levels	GCST90245536
rs6431630	4.00E-08	UGT1A9,UGT1A4,UGT1A5,UGT1A6,UGT1A10,UGT1A1,UGT1A7,UGT1A8,UGT1A3	Polyunsaturated fatty acid levels	GCST90502134

Supplemental Table 12. Genome-wide significant associations reported in the GWAS catalog within hg19 chr4:102,544,804-104,384,534 (hg38 chr4:101,623,646-103,463,377).

Variant	P Value	Mapped Genes	Reported Trait	Study Accession
rs223492	2.00E-291	MANBA	Beta-mannosidase levels	GCST90246709
rs3113676	2.00E-279	BANK1	BANK1/BCR protein level ratio	GCST90313454
rs3113676	1.00E-259	BANK1	BANK1/TXLNA protein level ratio	GCST90313465
rs13107325	2.00E-231	SLC39A8	Pallidum iron levels (quantitative susceptibility mapping)	GCST90551867
rs3113676	1.00E-219	BANK1	BANK1/SH2B3 protein level ratio	GCST90313464
rs3113676	2.00E-214	BANK1	BANK1/GOPC protein level ratio	GCST90313457
rs3113676	4.00E-208	BANK1	BANK1/HEXIM1 protein level ratio	GCST90313458
rs3113676	2.00E-203	BANK1	BANK1/CALCOCO1 protein level ratio	GCST90313455
rs13107325	2.00E-197	SLC39A8	Substantia nigra iron levels (quantitative susceptibility mapping)	GCST90551871
rs13107325	1.00E-193	SLC39A8	high density lipoprotein cholesterol (HDLC, mean, inv-norm transformed)	GCST90475352
rs13107325	1.00E-185	SLC39A8	High density lipoprotein cholesterol levels	GCST90239649
rs3113676	3.00E-184	BANK1	BANK1/FOXO1 protein level ratio	GCST90313456
rs13107325	6.00E-175	SLC39A8	Vertex-wise cortical thickness	GCST90095131
rs13107325	8.00E-174	SLC39A8	high density lipoprotein cholesterol (HDLC, minimm, inv-norm transformed)	GCST90475356
rs13107325	3.00E-171	SLC39A8	high density lipoprotein cholesterol (HDLC, maximum, inv-norm transformed)	GCST90475348
rs13107325	6.00E-169	SLC39A8	Brain morphology (MOSTest)	GCST90239729
rs13107325	1.00E-167	SLC39A8	Caudate iron levels (quantitative susceptibility mapping)	GCST90551863
rs13107325	5.00E-165	SLC39A8	SDC1 protein levels	GCST90470558
rs3113676	4.00E-162	BANK1	BANK1/IRAK4 protein level ratio	GCST90313461
rs1125271	2.00E-158	BANK1	Calcineurin levels	GCST90246815
rs13107325	2.00E-153	SLC39A8	Pallidum iron levels (R2* MRI)	GCST90551868
rs13107325	1.00E-133	SLC39A8	Liver fibrosis and steatohepatitis severity (MRI cT1 measure)	GCST010418
rs13107325	6.00E-127	SLC39A8	SLITRK2 protein levels	GCST90470670
rs13107325	6.00E-127	SLC39A8	Substantia nigra iron levels (R2* MRI)	GCST90551872
rs13107325	6.00E-125	SLC39A8	Whole brain restricted isotropic diffusion (multivariate analysis)	GCST90131904
rs13107325	3.00E-124	SLC39A8	Brain morphology (MOSTest)	GCST010703
rs13107325	1.00E-119	SLC39A8	IDP T1 FAST ROIs L ventral striatum	GCST90002559
rs13107325	5.00E-118	SLC39A8	High density lipoprotein cholesterol levels	GCST90020233
rs13107325	6.00E-117	SLC39A8	Body mass index (BMI, maximum, inv-normal transformed)	GCST90475153
rs13107325	2.00E-115	SLC39A8	IDP T1 FAST ROIs R ventral striatum	GCST90002560
rs13107325	3.00E-115	SLC39A8	High density lipoprotein cholesterol levels	GCST90239652
rs227370	8.00E-113	MANBA	Beta-mannosidase levels (MANBA.6382.17.3)	GCST90240437
rs13107325	2.00E-108	SLC39A8	HDL cholesterol levels	GCST010242
rs13107325	5.00E-108	SLC39A8	Body mass index (BMI, mean, inv-normal transformed)	GCST90475156
rs223401	5.00E-106	UBE2D3	Serum levels of protein MANBA	GCST90089383
rs13107325	3.00E-104	SLC39A8	IDP T1 FAST ROIs R putamen	GCST90002552
rs13107325	2.00E-103	SLC39A8	IDP T1 FAST ROIs L putamen	GCST90002551
rs13107325	7.00E-103	SLC39A8	Haematocrit percentage (UKB data field 30030)	GCST90468073
rs13107325	1.00E-102	SLC39A8	CPQ protein levels	GCST90468846
rs13107325	4.00E-101	SLC39A8	Volume of right central nucleus	GCST90474916
rs13107325	5.00E-100	SLC39A8	Diastolic blood pressure	GCST90310295
rs13107325	7.00E-99	SLC39A8	Central nucleus volume	GCST90310229
rs13107325	1.00E-98	SLC39A8	Volume of right medial nucleus	GCST90474925
rs13107325	6.00E-98	SLC39A8	diastolic blood pressure (DBP, mean, inv-normal transformed)	GCST90475255
rs13107325	5.00E-97	SLC39A8	aseg rh intensity Accumbens-area	GCST90003788
rs13107325	2.00E-95	SLC39A8	CD300C/LRRC25 protein level ratio	GCST90313790
rs13107325	2.00E-94	SLC39A8	Body mass index	GCST90255621
rs7662107	6.00E-94	LINC02428	Height	GCST90245848
rs13107325	1.00E-93	SLC39A8	IDP T1 FAST ROIs V cerebellum IX	GCST90002585
rs145761363	1.00E-89	BANK1,SLC39A8	Bone mineral density mean	GCST90321120
rs114147514	2.00E-88	SLC9B2	Bone mineral density mean	GCST90321120
rs13126505	2.00E-88	BANK1	Height	GCST90245848
rs13107325	2.00E-86	SLC39A8	high density lipoprotein cholesterol (HDLC, mean, inv-norm transformed)	GCST90479632
rs5026470	1.00E-84	MANBA	Lymphocyte count	GCST90002316
rs13107325	1.00E-84	SLC39A8	Central nucleus volume	GCST90310219
rs5026470	6.00E-84	MANBA	Lymphocyte count	GCST90002320
rs3113676	2.00E-83	BANK1	BANK1/LBR protein level ratio	GCST90313462
rs78336913	4.00E-83	CENPE	3-hydroxybutyrate dehydrogenase type 2 levels	GCST90246683
rs13107325	1.00E-82	SLC39A8	high density lipoprotein cholesterol (HDLC, maximum, inv-norm transformed)	GCST90479631
rs13107325	5.00E-82	SLC39A8	Apolipoprotein A1 levels	GCST010241
rs13107325	3.00E-81	SLC39A8	Diastolic blood pressure (MTAG)	GCST90449057
rs13114738	6.00E-81	SLC39A8	Height	GCST90245848
rs13107325	1.00E-80	SLC39A8	aseg lh intensity Accumbens-area	GCST90003774
rs13107325	4.00E-80	SLC39A8	high density lipoprotein cholesterol (HDLC, minimm, inv-norm transformed)	GCST90479633
rs13107325	6.00E-80	SLC39A8	HDL cholesterol	GCST90018956
rs13107325	2.00E-79	SLC39A8	Apolipoprotein A levels (UKB data field 30630)	GCST90468061
rs13107325	4.00E-79	SLC39A8	Caudate iron levels (R2* MRI)	GCST90551864
rs13107325	1.00E-77	SLC39A8	aseg rh intensity Putamen	GCST90003784
rs13107325	7.00E-77	SLC39A8	aseg lh intensity Putamen	GCST90003770
rs13107325	2.00E-74	SLC39A8	Left cerebellum white matter shape (Laplace-Beltrami spectrum)	GCST90559211
rs13107325	2.00E-74	SLC39A8	Volume of right cortical nucleus	GCST90474919
rs13107325	7.00E-74	SLC39A8	Whole brain restricted directional diffusion (multivariate analysis)	GCST90131905
rs13107325	5.00E-73	SLC39A8	Subcortical volume (MOSTest)	GCST010702

rs13107325	6.00E-73	SLC39A8	Diastolic blood pressure	GCST90244036
rs28396651	4.00E-72	BANK1	Mean platelet thrombocyte volume (UKB data field 30100)	GCST90468087
rs13107325	1.00E-71	SLC39A8	Right cerebellum white matter shape (Laplace-Beltrami spectrum)	GCST90559221
rs13107325	2.00E-71	SLC39A8	Body mass index (BMI, minimum, inv-normal transformed)	GCST90475159
rs13107325	2.00E-69	SLC39A8	Diastolic blood pressure	GCST90624421
rs223489	3.00E-69	MANBA	Blood protein levels	GCST006585
rs3113676	1.00E-68	BANK1	BANK1 protein levels	GCST90468421
rs13107325	8.00E-68	SLC39A8	CD300A protein levels	GCST90468619
rs13107325	2.00E-67	SLC39A8	Weight (maximum, inv-normal transformed)	GCST90476460
rs13107325	5.00E-67	SLC39A8	IDP SWI T2star right pallidum	GCST90003870
rs13107325	5.00E-66	SLC39A8	Volume of left central nucleus	GCST90474883
rs13107325	7.00E-65	SLC39A8	Metabolic syndrome	GCST90444487
rs13107325	1.00E-63	SLC39A8	Volume of left medial nucleus	GCST90474892
rs13107325	6.00E-63	SLC39A8	CD300C/GOLM2 protein level ratio	GCST90313788
rs17032705	9.00E-63	NFKB1	NFKB1 protein levels	GCST90470037
rs13107325	1.00E-62	SLC39A8	CD300C/FOLR2 protein level ratio	GCST90313787
rs13107325	4.00E-62	SLC39A8	Weight (mean, inv-normal transformed)	GCST90476463
rs230540	2.00E-61	NFKB1	Membranous nephropathy	GCST010006
rs1077358	2.00E-59	MANBA	Lymphocyte count (UKB data field 30120)	GCST90468082
rs13107325	3.00E-59	SLC39A8	Drinks per week	GCST90243989
rs13107325	6.00E-59	SLC39A8	Systolic blood pressure	GCST90310294
rs201081507	4.00E-58	BANK1	Pallidum iron levels (quantitative susceptibility mapping)	GCST90551867
rs13135092	4.00E-58	SLC39A8	Educational attainment	GCST90105038
rs13107325	4.00E-58	SLC39A8	Volume of right medial nucleus	GCST90475042
rs13107325	1.00E-57	SLC39A8	AmygNuclei rh volume Medial-nucleus	GCST90002663
rs13107325	2.00E-57	SLC39A8	High-density lipoprotein levels (MTAG)	GCST90179147
rs33969351	3.00E-57	MANBA	Lymphocyte count	GCST90002388
rs13107325	6.00E-57	SLC39A8	diastolic blood pressure (DBP, mean, inv-normal transformed)	GCST90479581
rs13107325	1.00E-56	SLC39A8	Volume of right central nucleus	GCST90475024
rs201081507	2.00E-56	BANK1	Substantia nigra iron levels (quantitative susceptibility mapping)	GCST90551871
rs13107325	2.00E-56	SLC39A8	Drinks per week	GCST90243984
rs200731261	1.00E-55	NFKB1,MANBA	Lymphocyte percentage (UKB data field 30180)	GCST90468083
rs13107325	1.00E-55	SLC39A8	CD300C/HAVCR2 protein level ratio	GCST90313789
rs13107325	2.00E-55	SLC39A8	HS6ST1 protein levels	GCST90469479
rs7679882	6.00E-55	BANK1	Mean platelet volume	GCST90002346
rs13107325	2.00E-54	SLC39A8	Vertex-wise sulcal depth	GCST90095129
rs13107325	3.00E-54	SLC39A8	Aspartate aminotransferase levels	GCST90020237
rs13107325	6.00E-54	SLC39A8	Diastolic blood pressure	GCST90132904
rs13107325	1.00E-53	SLC39A8	DSG3 protein levels	GCST90469043
rs13107325	2.00E-53	SLC39A8	AmygNuclei rh volume Central-nucleus	GCST90002662
rs13107325	6.00E-53	SLC39A8	IDP SWI T2star left pallidum	GCST90003869
rs13107325	7.00E-53	SLC39A8	Systolic blood pressure (MTAG)	GCST90449056
rs7679882	1.00E-52	BANK1	Mean platelet volume	GCST90002349
rs13107325	2.00E-52	SLC39A8	Body mass index or hip osteoarthritis (pleiotropy)	GCST90271771
rs13107325	9.00E-52	SLC39A8	aseg rh intensity Pallidum	GCST90003785
rs13135688	1.00E-51	SLC39A8	Central nucleus volume	GCST90310229
rs13107325	1.00E-51	SLC39A8	VCAN protein levels	GCST90471032
rs980455	1.00E-51	NFKB1,SLC39A8	Height	GCST90245848
rs201081507	2.00E-51	BANK1	Caudate iron levels (quantitative susceptibility mapping)	GCST90551863
rs13107325	2.00E-51	SLC39A8	Body mass index or osteoarthritis (pleiotropy)	GCST90271767
rs10516487	3.00E-51	BANK1	CASP8/PPP3R1 protein level ratio	GCST90313654
rs34592089	3.00E-51	BANK1	High density lipoprotein cholesterol levels	GCST90019510
rs13107325	6.00E-51	SLC39A8	Obesity (PheCode 278.1)	GCST90475762
rs13105682	8.00E-51	BANK1	IDP T1 FAST ROIs L ventral striatum	GCST90002559
rs13107325	9.00E-51	SLC39A8	NELL1 protein levels	GCST90470026
rs13107325	2.00E-50	SLC39A8	HDL cholesterol	GCST006611
rs13105682	8.00E-50	BANK1	IDP T1 FAST ROIs L putamen	GCST90002551
rs13107325	1.00E-49	SLC39A8	IDP T1 FAST ROIs R cerebellum IX	GCST90002586
rs13107325	3.00E-49	SLC39A8	Overweight, obesity and other hyperalimentation (PheCode 278)	GCST90475759
rs13107325	4.00E-49	SLC39A8	IDP T1 FAST ROIs L cerebellum IX	GCST90002584
rs13107325	5.00E-49	SLC39A8	Diastolic blood pressure	GCST90472777
rs17199964	1.00E-48	BANK1	IDP T1 FAST ROIs R ventral striatum	GCST90002560
rs13107325	2.00E-48	SLC39A8	MAMDC2 protein levels	GCST90469841
rs13107325	2.00E-48	SLC39A8	Body mass index or knee osteoarthritis (pleiotropy)	GCST90271770
rs13107325	3.00E-48	SLC39A8	Calcium levels	GCST012398
rs34333163	1.00E-47	SLC39A8	High density lipoprotein cholesterol levels	GCST90019510
rs13107325	4.00E-47	SLC39A8	Body mass index	GCST009004
rs13107325	4.00E-47	SLC39A8	Body mass index	GCST009003
rs13107325	4.00E-47	SLC39A8	Body mass index	GCST009001
rs13107325	5.00E-47	SLC39A8	Calcium levels (UKB data field 30680)	GCST90468065
rs13107325	5.00E-47	SLC39A8	Volume of right central nucleus	GCST90475027
rs13105682	6.00E-47	BANK1	IDP T1 FAST ROIs R putamen	GCST90002552
rs13107325	2.00E-46	SLC39A8	Body mass index (BMI, maximum, inv-normal transformed)	GCST90479521
rs13107325	3.00E-46	SLC39A8	MEGF9 protein levels	GCST90469884
rs13105682	4.00E-46	BANK1	aseg rh intensity Accumbens-area	GCST90003788
rs13107325	4.00E-46	SLC39A8	Body mass index (MTAG)	GCST90179150

rs56375208	7.00E-46	UBE2D3	Beta-mannosidase levels (MANBA.6382.17.3)	GCST90240437
rs13107325	8.00E-46	SLC39A8	HEPH protein levels	GCST90469446
rs34654981	1.00E-45	UBE2D3,UBE2D3-AS1	merular filtration rate based on creatinine and cystatin C in bottom 99% of individuals by cre	GCST90566737
rs13107325	3.00E-45	SLC39A8	Body mass index (BMI, mean, inv-normal transformed)	GCST90479522
rs7679882	4.00E-45	BANK1	Mean platelet volume	GCST90002395
rs13107325	4.00E-45	SLC39A8	Whole brain free water diffusion (multivariate analysis)	GCST90131906
rs71597109	5.00E-45	BANK1	PPP3R1 protein levels	GCST90470307
rs13107325	5.00E-45	SLC39A8	Volume of right cortical nucleus	GCST90475030
rs13107325	6.00E-45	SLC39A8	Total cholesterol levels	GCST90239673
rs34333163	1.00E-44	SLC39A8	Left cerebellum white matter shape (Laplace-Beltrami spectrum)	GCST90559211
rs151222359	2.00E-44	UBE2D3	Estimated glomerular filtration rate (creatinine, cystatin c)	GCST90428446
rs34333163	5.00E-44	SLC39A8	Right cerebellum white matter shape (Laplace-Beltrami spectrum)	GCST90559221
rs13107325	6.00E-44	SLC39A8	Aspartate aminotransferase levels (UKB data field 30650)	GCST90468063
rs13107325	7.00E-44	SLC39A8	CLEC14A protein levels	GCST90468766
rs13107325	8.00E-44	SLC39A8	Anterior amygdaloid area volume	GCST90310230
rs13107325	1.00E-43	SLC39A8	IDP T1 FAST ROIs V cerebellum X	GCST90002588
rs6855246	2.00E-43	SLC39A8,BANK1	Left cerebellum white matter shape (Laplace-Beltrami spectrum)	GCST90559211
rs13107325	2.00E-43	SLC39A8	Diastolic blood pressure	GCST90301698
rs13107325	3.00E-43	SLC39A8	Femur length to body height ratio	GCST90270256
rs13107325	4.00E-43	SLC39A8	Volume of right medial nucleus	GCST90475045
rs13107325	1.00E-42	SLC39A8	Brain imaging measurements	GCST006777
rs13135092	3.00E-42	SLC39A8	Problematic alcohol use (MTAG)	GCST010544
rs13107325	3.00E-42	SLC39A8	Aspartate aminotransferase levels	GCST90018944
rs34592089	4.00E-42	BANK1	Left cerebellum white matter shape (Laplace-Beltrami spectrum)	GCST90559211
rs13107325	5.00E-42	SLC39A8	Calcium levels	GCST90018951
rs13107325	5.00E-42	SLC39A8	Volume of left central nucleus	GCST90474958
rs17199964	1.00E-41	BANK1	Central nucleus volume	GCST90310229
rs5026472	2.00E-41	MANBA	Lymphocyte count	GCST90018962
rs13107325	2.00E-41	SLC39A8	Brain imaging measurements	GCST006777
rs34592089	2.00E-41	BANK1	Apolipoprotein A1 levels	GCST90019495
rs5026473	3.00E-41	MANBA	Lymphocyte-to-monocyte ratio	GCST90056181
rs13107325	4.00E-41	SLC39A8	diastolic blood pressure (DBP, maximum, inv-normal transformed)	GCST90475252
rs13107325	5.00E-41	SLC39A8	Anterior amygdaloid area volume	GCST90310221
rs6833745	5.00E-41	NFKB1,SLC39A8	Total protein levels (UKB data field 30860)	GCST90468105
rs34592089	8.00E-41	BANK1	Right cerebellum white matter shape (Laplace-Beltrami spectrum)	GCST90559221
rs13107325	1.00E-40	SLC39A8	Platelet distribution width (UKB data field 30110)	GCST90468097
rs13107325	1.00E-40	SLC39A8	CD300C protein levels	GCST90468620
rs13107325	2.00E-40	SLC39A8	Hematocrit	GCST90002304
rs13107325	2.00E-40	SLC39A8	SSC5D protein levels	GCST90470748
rs13107325	5.00E-40	SLC39A8	Body mass index	GCST008129
rs13107325	6.00E-40	SLC39A8	Problematic alcohol use (MTAG)	GCST90244189
rs4699032	7.00E-40	CISD2,SLC9B1	Estimated glomerular filtration rate (creatinine)	GCST90100220
rs13105682	2.00E-39	BANK1	aseg rh intensity Putamen	GCST90003784
rs13107325	2.00E-39	SLC39A8	AmygNuclei rh volume Cortical-nucleus	GCST90002664
rs13107325	2.00E-39	SLC39A8	Brain imaging measurements	GCST006777
rs13107325	2.00E-39	SLC39A8	Weight (minimum, inv-normal transformed)	GCST90476466
rs13105682	4.00E-39	BANK1	aseg lh intensity Putamen	GCST90003770
rs13126885	4.00E-39	SLC39A8	Height	GCST90245848
rs13107325	7.00E-39	SLC39A8	Brain imaging measurements	GCST006777
rs13107325	8.00E-39	SLC39A8	Body mass index	GCST009871
rs201081507	8.00E-39	BANK1	Pallidum iron levels (R2* MRI)	GCST90551868
rs13107325	1.00E-38	SLC39A8	Hemoglobin concentration	GCST90002310
rs13107325	2.00E-38	SLC39A8	Volume of left medial nucleus	GCST90474976
rs13107325	2.00E-38	SLC39A8	Alcohol-related disorders (PheCode 317)	GCST90476520
rs34333163	2.00E-38	SLC39A8	Apolipoprotein A1 levels	GCST90019495
rs13107325	3.00E-38	SLC39A8	Hematocrit	GCST90002308
rs13107325	3.00E-38	SLC39A8	ADAMTS8 protein levels	GCST90468227
rs13107325	3.00E-38	SLC39A8	LRRC25 protein levels	GCST90469801
rs6855246	3.00E-38	SLC39A8,BANK1	Right cerebellum white matter shape (Laplace-Beltrami spectrum)	GCST90559221
rs13107325	4.00E-38	SLC39A8	Body mass index	GCST90301650
rs13105682	7.00E-38	BANK1	aseg lh intensity Accumbens-area	GCST90003774
rs13107325	7.00E-38	SLC39A8	Accumbens iron levels (quantitative susceptibility mapping)	GCST90551859
rs13107325	8.00E-38	SLC39A8	Cortical thickness (MOSTest)	GCST010700
rs13107325	1.00E-37	SLC39A8	Hemoglobin concentration	GCST90002314
rs13107325	1.00E-37	SLC39A8	aseg lh intensity Pallidum	GCST90003771
rs13107325	2.00E-37	SLC39A8	Triglyceride levels	GCST90239661
rs13107325	3.00E-37	SLC39A8	X-12681 levels	GCST90245552
rs13107325	4.00E-37	SLC39A8	IDP dMRI TBSS ICVF Cerebral peduncle L	GCST90004342
rs13105682	6.00E-37	BANK1	IDP T1 FAST ROIs V cerebellum IX	GCST90002585
rs13107325	6.00E-37	SLC39A8	SOD3 protein levels	GCST90470706
rs201081507	6.00E-37	BANK1	Substantia nigra iron levels (R2* MRI)	GCST90551872
rs13107325	7.00E-37	SLC39A8	Body mass index (UKB data field 21001)	GCST90468161
rs13107325	1.00E-36	SLC39A8	Systolic blood pressure	GCST007087
rs13107325	1.00E-36	SLC39A8	Body mass index	GCST007039
rs13107325	2.00E-36	SLC39A8	IDP dMRI TBSS ICVF Cerebral peduncle R	GCST90004341
rs13107325	2.00E-36	SLC39A8	AmygNuclei lh volume Medial-nucleus	GCST90002653

rs13107325	2.00E-36	SLC39A8	Arm fat percentage left (UKB data field 23123)	GCST90468157
rs13107325	2.00E-36	SLC39A8	GFRA3 protein levels	GCST90469330
rs13107325	3.00E-36	SLC39A8	RGMA/RGMB protein level ratio	GCST90315769
rs13107325	3.00E-36	SLC39A8	PEAR1 protein levels	GCST90470204
rs13107325	4.00E-36	SLC39A8	Arm fat percentage right (UKB data field 23119)	GCST90468158
rs13107325	5.00E-36	SLC39A8	Systolic blood pressure	GCST90132903
rs6833745	6.00E-36	NFKB1,SLC39A8	Serum total protein levels	GCST90018976
rs13107325	6.00E-36	SLC39A8	Maximum habitual alcohol consumption (MTAG)	GCST90244188
rs13107325	8.00E-36	SLC39A8	TNFSF12 protein levels	GCST90470919
rs13107325	1.00E-35	SLC39A8	Volume of left cortical nucleus	GCST90474886
rs13107325	1.00E-35	SLC39A8	systolic blood pressure (SBP, mean, inv-normal transformed)	GCST90476403
rs13107325	1.00E-35	SLC39A8	diastolic blood pressure (DBP, minimum, inv-normal transformed)	GCST90475256
rs13135092	2.00E-35	SLC39A8	Alcohol consumption (drinks per week) (MTAG)	GCST010543
rs13107325	3.00E-35	SLC39A8	Right-hemisphere limbic network to hippocampus white-matter structural connectivity	GCST90302831
rs200731261	6.00E-35	NFKB1,MANBA	Neutrophill percentage (UKB data field 30200)	GCST90468093
rs13107325	6.00E-35	SLC39A8	Osteoarthritis	GCST90566795
rs11724614	1.00E-34	NFKB1,MANBA	Neutrophil-to-lymphocyte ratio	GCST90056182
rs13107325	1.00E-34	SLC39A8	Total cholesterol levels	GCST90239676
rs151222359	4.00E-34	UBE2D3	Estimated glomerular filtration rate (cystatin c)	GCST90428448
rs13107325	5.00E-34	SLC39A8	IDP T1 FAST ROIs L cerebellum VIIIb	GCST90002581
rs13109404	5.00E-34	BANK1	Diastolic blood pressure	GCST90132904
rs223397	5.00E-34	UBE2D3	Atrial fibrillation	GCST90624411
rs151222359	5.00E-34	UBE2D3	Estimated glomerular filtration rate (creatinine)	GCST90428447
rs13107325	6.00E-34	SLC39A8	APOA2 protein levels	GCST90468332
rs13107325	7.00E-34	SLC39A8	Body mass index (BMI, minimum, inv-normal transformed)	GCST90479523
rs13135092	7.00E-34	SLC39A8	Problematic alcohol use	GCST90274727
rs11726195	8.00E-34	NFKB1,MANBA	Neutrophil percentage of white cells	GCST90002399
rs227285	1.00E-33	MANBA	Beta-mannosidase levels	GCST90426664
rs6830624	1.00E-33	BDH2	3-hydroxybutyrate dehydrogenase type 2 levels	GCST90422951
rs13135092	2.00E-33	SLC39A8	IDL cholesterol levels x alcohol consumption (regular vs non-regular drinkers) interaction (2df	GCST008075
rs13107325	2.00E-33	SLC39A8	CCDC80 protein levels	GCST90468562
rs34654981	2.00E-33	UBE2D3,UBE2D3-AS1	Creatinine levels in bottom 99% of individuals by creatinine levels	GCST90566733
rs13107325	3.00E-33	SLC39A8	Sleep apnea	GCST90475824
rs13107325	5.00E-33	SLC39A8	Diastolic blood pressure	GCST007094
rs13107325	8.00E-33	SLC39A8	Serum albumin levels	GCST90018945
rs13107325	8.00E-33	SLC39A8	ITGB6 protein levels	GCST90469646
rs13107325	9.00E-33	SLC39A8	Diastolic blood pressure	GCST90435414
rs151407	1.00E-32	BANK1,SLC39A8	Central nucleus volume	GCST90310229
rs13107325	1.00E-32	SLC39A8	Volume of right cortical nucleus	GCST90475033
rs13135092	2.00E-32	SLC39A8	IDL cholesterol levels x alcohol consumption (regular vs non-regular drinkers) interaction (2df	GCST008075
rs13107325	2.00E-32	SLC39A8	Body mass index	GCST90435413
rs6533022	2.00E-32	MANBA	Primary biliary cholangitis	GCST90061442
rs13107325	2.00E-32	SLC39A8	Volume of right accessory basal nucleus	GCST90474907
rs13107325	5.00E-32	SLC39A8	Haemoglobin concentration (UKB data field 30020)	GCST90468074
rs13101632	5.00E-32	BANK1,SLC39A8	Central nucleus volume	GCST90310229
rs13107325	5.00E-32	SLC39A8	Triglyceride to HDL cholesterol ratio	GCST90295949
rs13107325	5.00E-32	SLC39A8	Metabolic biomarkers (multivariate analysis)	GCST90038594
rs13107325	7.00E-32	SLC39A8	Hemoglobin	GCST90002384
rs10012413	7.00E-32	SLC9B1	Atrial fibrillation	GCST90624412
rs7377083	7.00E-32	BANK1	Body mass index	GCST90255621
rs12644381	9.00E-32	MANBA	Lymphocyte count	GCST90085815
rs5026472	1.00E-31	MANBA	Lymphocyte count	GCST004627
rs13107325	1.00E-31	SLC39A8	Triglyceride to HDL cholesterol ratio	GCST90435481
rs63519	1.00E-31	SLC39A8	Volume of right medial nucleus	GCST90474924
rs13135092	2.00E-31	SLC39A8	HDL cholesterol levels x alcohol consumption (drinkers vs non-drinkers) interaction (2df)	GCST008084
rs13107325	2.00E-31	SLC39A8	Hematocrit	GCST90002383
rs13135092	2.00E-31	SLC39A8	Problematic alcohol use	GCST010546
rs13107325	2.00E-31	SLC39A8	CREG1 protein levels	GCST90468857
rs13105682	2.00E-31	BANK1	IDP SWI T2star right pallidum	GCST90003870
rs5026473	2.00E-31	MANBA	Platelet-to-lymphocyte ratio	GCST90056184
rs13107325	2.00E-31	SLC39A8	IL1RL1 protein levels	GCST90469574
rs13107325	2.00E-31	SLC39A8	Body mass index or osteoarthritis (pleiotropy)	GCST90271769
rs13107325	3.00E-31	SLC39A8	IDP dMRI TBSS OD Cerebral peduncle L	GCST90004417
rs223317	3.00E-31	CISD2	Estimated glomerular filtration rate (creatinine)	GCST90103633
rs13107325	4.00E-31	SLC39A8	DSG4 protein levels	GCST90469044
rs223485	4.00E-31	MANBA,UBE2D3	Cystatin C levels in bottom 99% of individuals by creatinine levels	GCST90566734
rs13107325	4.00E-31	SLC39A8	triglyceride (mean, inv-norm transformed)	GCST90476435
rs13107325	7.00E-31	SLC39A8	IDP T1 FAST ROIs R cerebellum VIIIb	GCST90002583
rs13107325	1.00E-30	SLC39A8	IDP SWI T2star right caudate	GCST90003866
rs13107325	1.00E-30	SLC39A8	IDP dMRI TBSS OD Cerebral peduncle R	GCST90004416
rs13107325	1.00E-30	SLC39A8	Diastolic blood pressure	GCST90472778
rs67037577	2.00E-30	UBE2D3,MANBA	Creatinine levels (UKB data field 30700)	GCST90468067
rs13107325	2.00E-30	SLC39A8	AmygNuclei lh volume Central-nucleus	GCST90002652
rs13107325	2.00E-30	SLC39A8	Alcohol use disorder (consumption score)	GCST008258
rs62327181	3.00E-30	NFKB1,MANBA	Monocyte percentage of white cells	GCST90002394
rs13107325	3.00E-30	SLC39A8	IDP T1 FAST ROIs V cerebellum VIIIb	GCST90002582

rs13105682	4.00E-30	BANK1	AmygNuclei rh volume Medial-nucleus	GCST90002663
rs13107325	4.00E-30	SLC39A8	hematocrit (maximum, inv-norm transformed)	GCST90475338
rs34592089	6.00E-30	BANK1	Calcium levels	GCST90019500
rs13135092	1.00E-29	SLC39A8	HDL cholesterol levels x alcohol consumption (drinkers vs non-drinkers) interaction (2df)	GCST008084
rs13107325	1.00E-29	SLC39A8	Platelet distribution width	GCST90002401
rs223376	1.00E-29	UBE2D3-AS1,UBE2D3	Serum creatinine levels	GCST90018979
rs13107325	1.00E-29	SLC39A8	Medial nucleus volume	GCST90310225
rs223471	2.00E-29	UBE2D3,MANBA	Estimated glomerular filtration rate (creatinine)	GCST90103633
rs13107325	2.00E-29	SLC39A8	CD55 protein levels	GCST90468637
rs13135092	3.00E-29	SLC39A8	Alcohol consumption (drinks per month) (UKB data field 1578, 4424)	GCST90132992
rs13107325	4.00E-29	SLC39A8	Accessory basal nucleus volume	GCST90310224
rs6855246	5.00E-29	SLC39A8,BANK1	Osteoarthrosis (PheCode 740)	GCST90476253
rs13107325	9.00E-29	SLC39A8	Low-density lipoprotein levels (MTAG)	GCST90179148
rs2272676	1.00E-28	NFKB1	Mean reticulocyte volume (UKB data field 30260)	GCST90468088
rs13107325	1.00E-28	SLC39A8	Body mass index	GCST90018947
rs13107325	1.00E-28	SLC39A8	Regional cortical thickness (lateraloccipital)	GCST90399885
rs13107325	1.00E-28	SLC39A8	diastolic blood pressure (DBP, maximum, inv-normal transformed)	GCST90479580
rs7688014	1.00E-28	SLC9B2	creatinine (mean, inv-norm transformed)	GCST90475229
rs13107325	2.00E-28	SLC39A8	IDP SWI T2star left caudate	GCST90003865
rs13107325	2.00E-28	SLC39A8	Regional cortical thickness (cuneus)	GCST90399879
rs62328536	2.00E-28	SLC39A8,NFKB1	Nuclear factor NF-kappa-B p105 subunit levels	GCST90248671
rs13135092	2.00E-28	SLC39A8	Body mass index	GCST90428119
rs13107325	3.00E-28	SLC39A8	Adult body size	GCST010988
rs13107325	3.00E-28	SLC39A8	aseg lh intensity Cerebellum-Cortex	GCST90003767
rs13107325	3.00E-28	SLC39A8	IDP T1 FAST ROIs L cerebellum I-IV	GCST90002562
rs13107325	3.00E-28	SLC39A8	Body fat percentage	GCST90020232
rs13107325	3.00E-28	SLC39A8	GALNT7 protein levels	GCST90469299
rs755492124	4.00E-28	BANK1	ANGPT1 protein levels	GCST90468300
rs13107325	4.00E-28	SLC39A8	Left accumbens area shape (Laplace-Beltrami spectrum)	GCST90559207
rs13107325	4.00E-28	SLC39A8	Overweight, obesity and other hyperalimentation (PheCode 278)	GCST90479955
rs13107325	5.00E-28	SLC39A8	Fluid intelligence score (baseline)	GCST90565842
rs13107325	6.00E-28	SLC39A8	SIGLEC6 protein levels	GCST90470634
rs13107325	6.00E-28	SLC39A8	hemoglobin (maximum, inv-norm transformed)	GCST90475372
rs11446392	8.00E-28	SLC39A8	Volume of right central nucleus	GCST90474915
rs13107325	1.00E-27	SLC39A8	Obstructive sleep apnea (PheCode 327.32)	GCST90475825
rs13107325	1.00E-27	SLC39A8	Calcium (mean, inv-norm transformed)	GCST90475176
rs7377083	2.00E-27	BANK1	Body mass index	GCST009871
rs13107325	2.00E-27	SLC39A8	Body fat percentage (UKB data field 23099)	GCST90468160
rs13107325	2.00E-27	SLC39A8	GALNT3 protein levels	GCST90469297
rs7377083	2.00E-27	BANK1	Body mass index (MTAG)	GCST90179150
rs13107325	2.00E-27	SLC39A8	Triglyceride levels	GCST90239664
rs13107325	2.00E-27	SLC39A8	Volume of left medial nucleus	GCST90474979
rs13105682	3.00E-27	BANK1	aseg rh intensity Pallidum	GCST90003785
rs5026472	3.00E-27	MANBA	Neutrophils and lymphocytes in blood (confirmatory factor analysis Factor 35)	GCST90309369
rs13107325	3.00E-27	SLC39A8	Basal ganglia structure	GCST90432154
rs34592089	3.00E-27	BANK1	Aspartate aminotransferase levels	GCST90019497
rs13107325	5.00E-27	SLC39A8	Volume of left central nucleus	GCST90474961
rs13107325	6.00E-27	SLC39A8	IDP T1 FAST ROIs V cerebellum VIIIa	GCST90002579
rs13107325	6.00E-27	SLC39A8	CA12 protein levels	GCST90468507
rs13107325	7.00E-27	SLC39A8	triglyceride (maximum, inv-norm transformed)	GCST90476431
rs13107325	1.00E-26	SLC39A8	Hip circumference (UKB data field 49)	GCST90468170
rs13107325	1.00E-26	SLC39A8	ANGPT2 protein levels	GCST90468301
rs35225200	1.00E-26	BANK1,SLC39A8	Osteoarthritis, localized (PheCode 740.1)	GCST90476254
rs13107325	2.00E-26	SLC39A8	RNASET2 protein levels	GCST90470481
rs13107325	2.00E-26	SLC39A8	Obesity (PheCode 278.1)	GCST90479952
rs13107325	2.00E-26	SLC39A8	Alcohol-related disorders (PheCode 317)	GCST90480768
rs13135092	3.00E-26	SLC39A8	Hand grip strength	GCST005830
rs13107325	3.00E-26	SLC39A8	HDL cholesterol levels	GCST90092822
rs13107325	4.00E-26	SLC39A8	Cholesteryl ester levels in HDL	GCST90092823
rs13107325	4.00E-26	SLC39A8	Ventromedial occipital thickness (unadjusted for global measures)	GCST90271813
rs4648010	5.00E-26	NFKB1	ALDH1A1/CA3 protein level ratio	GCST90313244
rs13107325	8.00E-26	SLC39A8	Accessory basal nucleus volume	GCST90310213
rs13107325	8.00E-26	SLC39A8	Volume of left cortical nucleus	GCST90474964
rs223471	1.00E-25	UBE2D3,MANBA	Estimated glomerular filtration rate	GCST008058
rs13107325	1.00E-25	SLC39A8	Alcohol use disorder (consumption score)	GCST008258
rs13107325	1.00E-25	SLC39A8	TEK protein levels	GCST90470829
rs13107325	1.00E-25	SLC39A8	Personality traits or cognitive traits (multivariate analysis)	GCST90270074
rs13107325	1.00E-25	SLC39A8	Body mass index or osteoarthritis (pleiotropy)	GCST90271768
rs13107325	1.00E-25	SLC39A8	Weight (maximum, inv-normal transformed)	GCST90480726
rs13107325	2.00E-25	SLC39A8	IDP T1 FAST ROIs brain stem	GCST90002561
rs13107325	3.00E-25	SLC39A8	Myelin protein P0 levels	GCST90248559
rs13107325	3.00E-25	SLC39A8	red blood cell count (RBC, maximum, inv-norm transformed)	GCST90476345
rs13107325	4.00E-25	SLC39A8	Hemoglobin	GCST90018957
rs13127398	4.00E-25	BANK1	Systolic blood pressure	GCST90132903
rs7377083	4.00E-25	BANK1	Body mass index	GCST90301650
rs13107325	6.00E-25	SLC39A8	Osteoarthrosis NOS (PheCode 740.9)	GCST90476256

rs13107325	8.00E-25	SLC39A8	CDH5 protein levels	GCST90468676
rs13107325	9.00E-25	SLC39A8	Free cholesterol to total lipids ratio in medium HDL	GCST90092897
rs13107325	9.00E-25	SLC39A8	PTPRM protein levels	GCST90470384
rs13107325	1.00E-24	SLC39A8	Alcohol consumption (drinks per week)	GCST007328
rs13107325	1.00E-24	SLC39A8	Free cholesterol levels in HDL	GCST90092824
rs13107325	1.00E-24	SLC39A8	Aspartate aminotransferase (AST, minimum, inv-norm transformed)	GCST90475124
rs13107325	2.00E-24	SLC39A8	Hemoglobin levels	GCST010083
rs13107325	2.00E-24	SLC39A8	Vertex-wise cortical surface area	GCST90095130
rs17199964	2.00E-24	BANK1	AmygNuclei rh volume Central-nucleus	GCST90002662
rs13107325	2.00E-24	SLC39A8	Educational attainment (MTAG)	GCST006571
rs13135092	2.00E-24	SLC39A8	Weight (mean, inv-normal transformed)	GCST90480727
rs34592089	2.00E-24	BANK1	Serum albumin levels	GCST90019493
rs34333163	2.00E-24	SLC39A8	Aspartate aminotransferase levels	GCST90019497
rs13107325	3.00E-24	SLC39A8	Trunk fat mass (UKB data field 23128)	GCST90468180
rs13107325	3.00E-24	SLC39A8	Decaffeinated coffee consumption and/or alcohol dependence and hazardous-use score	GCST90134527
rs93059	3.00E-24	NFKB1	IL5RA protein levels	GCST90469600
rs201081507	3.00E-24	BANK1	Caudate iron levels (R2* MRI)	GCST90551864
rs13107325	4.00E-24	SLC39A8	Hematocrit	GCST90018960
rs13107325	4.00E-24	SLC39A8	ANPEP/NT5E protein level ratio	GCST90313274
rs4643809	4.00E-24	BANK1	Systemic lupus erythematosus	GCST011956
rs13107325	4.00E-24	SLC39A8	Red blood cell erythrocyte count (UKB data field 30010)	GCST90468098
rs13107325	4.00E-24	SLC39A8	VIT protein levels	GCST90471041
rs17254118	5.00E-24	MANBA,LRRC37A15P	Bioavailable testosterone levels	GCST90012103
rs755492124	5.00E-24	BANK1	CD63 protein levels	GCST90468642
rs13107325	6.00E-24	SLC39A8	Red blood cell count	GCST007069
rs13107325	6.00E-24	SLC39A8	Walking pace	GCST011122
rs13107325	6.00E-24	SLC39A8	Total lipid levels in HDL	GCST90092825
rs34333163	6.00E-24	SLC39A8	Total cholesterol levels	GCST90019501
rs13107325	7.00E-24	SLC39A8	Insomnia	GCST90131901
rs13105682	8.00E-24	BANK1	IDP SWI T2star left pallidum	GCST90003869
rs13107325	8.00E-24	SLC39A8	Ventromedial occipital thickness	GCST90572713
rs755492124	8.00E-24	BANK1	APP protein levels	GCST90468344
rs13107325	9.00E-24	SLC39A8	Joint pain (confirmatory factor analysis Factor 14)	GCST90309348
rs4624655	9.00E-24	NFKB1,SLC39A8	Lymphocyte side scatter	GCST90281246
rs13107325	1.00E-23	SLC39A8	Subcortical volume (min-P)	GCST010698
rs13107325	1.00E-23	SLC39A8	Alcohol consumption (drinks per week) (MTAG)	GCST007472
rs13107325	1.00E-23	SLC39A8	B3GNT7 protein levels	GCST90468411
rs13107325	1.00E-23	SLC39A8	Phospholipid levels in large HDL	GCST90092852
rs13107325	1.00E-23	SLC39A8	Cognitive performance	GCST006572
rs13107325	1.00E-23	SLC39A8	Systolic blood pressure	GCST90472780
rs13107325	1.00E-23	SLC39A8	Morbid obesity (PheCode 278.11)	GCST90475765
rs13107325	1.00E-23	SLC39A8	Calcium (minimum, inv-norm transformed)	GCST90475179
rs13107325	2.00E-23	SLC39A8	Diastolic blood pressure x alcohol consumption interaction (2df test)	GCST006166
rs13107325	2.00E-23	SLC39A8	Brain morphology (min-P)	GCST010699
rs13114738	2.00E-23	SLC39A8	Body mass index	GCST90446645
rs13107325	2.00E-23	SLC39A8	Total lipid levels in large HDL	GCST90092850
rs13107325	2.00E-23	SLC39A8	Concentration of large HDL particles	GCST90092851
rs34592089	2.00E-23	BANK1	Total cholesterol levels	GCST90019501
rs13107325	3.00E-23	SLC39A8	Leg fat percentage left (UKB data field 23115)	GCST90468174
rs13107325	3.00E-23	SLC39A8	Schizophrenia	GCST90128471
rs13107325	3.00E-23	SLC39A8	LRRC37A2 protein levels	GCST90469802
rs13107325	4.00E-23	SLC39A8	aseg rh intensity Cerebellum-Cortex	GCST90003781
rs13107325	4.00E-23	SLC39A8	Hand grip strength	GCST005829
rs13107325	4.00E-23	SLC39A8	Diastolic blood pressure (UKB data field 4079)	GCST90468163
rs35978636	4.00E-23	BANK1	BANK1 protein levels	GCST90468421
rs13107325	4.00E-23	SLC39A8	Decaffeinated coffee consumption and/or alcohol use disorder score	GCST90134525
rs11446392	4.00E-23	SLC39A8	Volume of right cortical nucleus	GCST90474918
rs13107325	5.00E-23	SLC39A8	TNFRSF21 protein levels	GCST90470912
rs13107325	5.00E-23	SLC39A8	Intelligence	GCST90264174
rs13107325	6.00E-23	SLC39A8	Cognitive aspects of educational attainment	GCST90011875
rs13107325	7.00E-23	SLC39A8	Cholesterol levels (UKB data field 30690)	GCST90468066
rs223361	7.00E-23	UBE2D3	Diastolic blood pressure	GCST90310295
rs13107325	7.00E-23	SLC39A8	total cholesterol (mean, inv-norm transformed)	GCST90476424
rs13107325	8.00E-23	SLC39A8	Triglyceride percentage of total lipids in intermediate-density lipoprotein	GCST90454489
rs13107325	8.00E-23	SLC39A8	Alcohol use disorder	GCST90301660
rs13107325	8.00E-23	SLC39A8	diastolic blood pressure (DBP, minimum, inv-normal transformed)	GCST90479582
rs13107325	8.00E-23	SLC39A8	total cholesterol (minimum, inv-norm transformed)	GCST90476427
rs1598859	9.00E-23	NFKB1	Monocyte percentage (UKB data field 30190)	GCST90468091
rs13107325	9.00E-23	SLC39A8	Free cholesterol levels in medium HDL	GCST90092896
rs13107325	9.00E-23	SLC39A8	Calcium (mean, inv-norm transformed)	GCST90479530
rs13107325	1.00E-22	SLC39A8	aparc-a2009s rh thickness Pole-occipital	GCST90003718
rs13107325	1.00E-22	SLC39A8	BA-exvivo rh thickness V2	GCST90003537
rs12510679	1.00E-22	SLC39A8	Central nucleus volume	GCST90310229
rs13107325	1.00E-22	SLC39A8	Cholesterol levels in medium HDL	GCST90092892
rs755492124	1.00E-22	BANK1	BDNF protein levels	GCST90468437
rs28625045	1.00E-22	BANK1	PDGFA protein levels	GCST90470188

rs13107325	1.00E-22	SLC39A8	systolic blood pressure (SBP, maximum, inv-normal transformed)	GCST90476400
rs72696109	2.00E-22	NFKB1,SLC39A8	Mean corpuscular volume (UKB data field 30040)	GCST90468086
rs13107325	2.00E-22	SLC39A8	Body mass index and systole blood pressure (pairwise)	GCST011331
rs13107325	2.00E-22	SLC39A8	Alcohol consumption (drinks per week)	GCST007461
rs13107325	2.00E-22	SLC39A8	Cholesteryl ester levels in medium HDL	GCST90092894
rs13107325	2.00E-22	SLC39A8	Regional cortical thickness (paracentral)	GCST90399891
rs7674640	2.00E-22	NFKB1,MANBA	Primary biliary cholangitis	GCST90061440
rs13107325	2.00E-22	SLC39A8	AGRN protein levels	GCST90468255
rs13107325	2.00E-22	SLC39A8	ttention deficit hyperactivity disorder or autism spectrum disorder or intelligence (pleiotropy	GCST90134330
rs13107325	2.00E-22	SLC39A8	LAMP3 protein levels	GCST90469735
rs113473633	3.00E-22	NFKB1	Eosinophil counts	GCST90002298
rs13107325	3.00E-22	SLC39A8	QC SWI-to-T1 linear alignment discrepancy	GCST90006352
rs13107325	3.00E-22	SLC39A8	BA-exvivo rh thickness V1	GCST90003536
rs13135092	3.00E-22	SLC39A8	Total cerebellar volume (excluding Crus I vermis)	GCST90020190
rs13107325	3.00E-22	SLC39A8	LRPAP1 protein levels	GCST90469800
rs13107325	3.00E-22	SLC39A8	Alcohol use disorder	GCST90301659
rs1813006	3.00E-22	BANK1,SLC39A8	Body mass index	GCST90428119
rs13107325	4.00E-22	SLC39A8	Diastolic blood pressure x alcohol consumption interaction (2df test)	GCST006166
rs7377083	4.00E-22	BANK1	Body mass index	GCST90435413
rs13107325	4.00E-22	SLC39A8	Hand grip strength right (UKB data field 47)	GCST90468169
rs13107325	4.00E-22	SLC39A8	Cholesterol levels in large HDL	GCST90092844
rs13107325	4.00E-22	SLC39A8	Phospholipid levels in HDL	GCST90092827
rs7377083	5.00E-22	BANK1	Body size at age 10	GCST010989
rs13107325	5.00E-22	SLC39A8	Free cholesterol levels in large HDL	GCST90092848
rs13107325	5.00E-22	SLC39A8	Volume of left accessory basal nucleus	GCST90474874
rs13107325	6.00E-22	SLC39A8	High density lipoprotein cholesterol levels	GCST007140
rs13107325	6.00E-22	SLC39A8	Cholesteryl ester levels in large HDL	GCST90092846
rs230509	7.00E-22	NFKB1	Mean reticulocyte volume	GCST90002396
rs13107325	7.00E-22	SLC39A8	Decaffeinated coffee consumption and/or alcohol consumption score	GCST90134526
rs13107325	7.00E-22	SLC39A8	Apolipoprotein A1 levels	GCST90092808
rs13105682	9.00E-22	BANK1	IDP T1 FAST ROIs R cerebellum IX	GCST90002586
rs13107325	1.00E-21	SLC39A8	Height	GCST007841
rs10516497	1.00E-21	SLC9B2	Metabolic syndrome	GCST90444487
rs13107325	1.00E-21	SLC39A8	Diastolic blood pressure	GCST90018952
rs13107325	1.00E-21	SLC39A8	aparc-a2009s rh thickness G-cuneus	GCST90003687
rs13107325	2.00E-21	SLC39A8	Systolic blood pressure	GCST007099
rs4648045	2.00E-21	NFKB1	Lymphocyte percentage of white cells	GCST004632
rs141936164	2.00E-21	SLC39A8,NFKB1	Monocyte count	GCST90002340
rs13107325	2.00E-21	SLC39A8	Intelligence	GCST006250
rs13107325	2.00E-21	SLC39A8	CLEC3B protein levels	GCST90468770
rs2738926	2.00E-21	CISD2	Atrial fibrillation	GCST90559230
rs34333163	2.00E-21	SLC39A8	Calcium levels	GCST90019500
rs223308	3.00E-21	CISD2,SLC9B1	Estimated glomerular filtration rate	GCST008059
rs13107325	3.00E-21	SLC39A8	Total cholesterol levels	GCST90018974
rs28625045	3.00E-21	BANK1	EGF protein levels	GCST90469086
rs13107325	3.00E-21	SLC39A8	Body mass index	GCST006368
rs13107325	3.00E-21	SLC39A8	Cortical nucleus volume	GCST90310228
rs13107325	3.00E-21	SLC39A8	GFRA2 protein levels	GCST90469329
rs4572884	3.00E-21	BANK1	PPBP protein levels	GCST90470289
rs13135092	3.00E-21	SLC39A8	MELTF protein levels	GCST90469885
rs755492124	3.00E-21	BANK1	PDGFB protein levels	GCST90470189
rs7674004	3.00E-21	NFKB1,MANBA	lymphocyte (fraction, maximum, inv-norm transformed)	GCST90479666
rs13107325	3.00E-21	SLC39A8	hematocrit (maximum, inv-norm transformed)	GCST90479628
rs13107325	4.00E-21	SLC39A8	IDP T1 FAST ROIs L cerebellum V	GCST90002564
rs223361	4.00E-21	UBE2D3	Diastolic blood pressure	GCST006630
rs7688014	4.00E-21	SLC9B2	creatinine (maximum, inv-norm transformed)	GCST90475226
rs13107325	4.00E-21	SLC39A8	Alcoholism (PheCode 317.1)	GCST90480767
rs13107325	5.00E-21	SLC39A8	Concentration of medium HDL particles	GCST90092899
rs457134	5.00E-21	BANK1,SLC39A8	Body mass index or hip osteoarthritis (pleiotropy)	GCST90271771
rs13107325	6.00E-21	SLC39A8	High density lipoprotein cholesterol levels	GCST007140
rs11097787	6.00E-21	SLC39A8,NFKB1	Monocyte count	GCST90002344
rs13107325	6.00E-21	SLC39A8	wg rh intensity-contrast precuneus	GCST90003851
rs68103113	6.00E-21	SLC39A8	Central nucleus volume	GCST90310229
rs34592089	6.00E-21	BANK1	Body mass index	GCST90428119
rs13135092	7.00E-21	SLC39A8	High density lipoprotein cholesterol levels	GCST007140
rs13107325	7.00E-21	SLC39A8	systolic blood pressure (SBP, mean, inv-normal transformed)	GCST90480706
rs13107325	8.00E-21	SLC39A8	Dorsolateral prefrontal thickness	GCST90572705
rs13118152	8.00E-21	SLC39A8	Volume of right central nucleus	GCST90474915
rs13107325	8.00E-21	SLC39A8	Sleep apnea	GCST90479999
rs13107325	8.00E-21	SLC39A8	triglyceride (minimum, inv-norm transformed)	GCST90476439
rs577329140	9.00E-21	NFKB1,SLC39A8	Volume of right central nucleus	GCST90474915
rs13107325	9.00E-21	SLC39A8	Diastolic blood pressure	GCST90301700
rs13107325	9.00E-21	SLC39A8	Osteoarthrosis (PheCode 740)	GCST90480546
rs13107325	1.00E-20	SLC39A8	Waist-hip ratio	GCST007067
rs13140486	1.00E-20	SLC39A8,NFKB1	Anterior amygdaloid area volume	GCST90310230
rs13107325	1.00E-20	SLC39A8	Leg fat percentage right (UKB data field 23111)	GCST90468175

rs13107325	1.00E-20	SLC39A8	Hand grip strength (baseline)	GCST90565845
rs13118152	1.00E-20	SLC39A8	Volume of right medial nucleus	GCST90474924
rs13107325	1.00E-20	SLC39A8	Diastolic blood pressure	GCST90472779
rs13107325	1.00E-20	SLC39A8	Schizophrenia	GCST90503210
rs13107325	1.00E-20	SLC39A8	Osteoarthritis, localized (PheCode 740.1)	GCST90480544
rs7377083	2.00E-20	BANK1	Metabolic syndrome	GCST90444487
rs28625045	2.00E-20	BANK1	DKK1 protein levels	GCST90468997
rs13107325	2.00E-20	SLC39A8	IDP dMRI TBSS OD Anterior limb of internal capsule L	GCST90004419
rs13135092	2.00E-20	SLC39A8	aseg lh volume Accumbens-area	GCST90002626
rs230489	2.00E-20	SLC39A8,NFKB1	Central nucleus volume	GCST90310229
rs13107325	2.00E-20	SLC39A8	Lateral nucleus volume	GCST90310227
rs13107325	2.00E-20	SLC39A8	HEPACAM2 protein levels	GCST90469445
rs13140033	2.00E-20	NFKB1,SLC39A8	Diastolic blood pressure	GCST90472777
rs13109272	2.00E-20	SLC39A8	Body mass index	GCST90428119
rs13107325	2.00E-20	SLC39A8	Osteoarthrosis, localized, primary (PheCode 740.11)	GCST90476255
rs7688014	2.00E-20	SLC9B2	estimated glomerular filtration rate (eGFR, minimum, inv-norm transformed)	GCST90475285
rs35518360	2.00E-20	SLC39A8,BANK1	hematocrit (mean, inv-norm transformed)	GCST90475341
rs13107325	3.00E-20	SLC39A8	X-12339 levels	GCST90245539
rs13107325	4.00E-20	SLC39A8	Male-pattern baldness	GCST007020
rs230503	4.00E-20	NFKB1	High light scatter reticulocyte count	GCST90002385
rs13107325	5.00E-20	SLC39A8	Waist circumference adjusted for body mass index	GCST009867
rs4572884	5.00E-20	BANK1	CPXM1 protein levels	GCST90468849
rs13107325	5.00E-20	SLC39A8	Cortical surface area (MOSTest)	GCST010701
rs13107325	5.00E-20	SLC39A8	Systolic blood pressure	GCST90435415
rs13107325	5.00E-20	SLC39A8	hemoglobin (maximum, inv-norm transformed)	GCST90479638
rs3836561I	6.00E-20	NFKB1	Mean corpuscular volume	GCST90002334
rs13105682	6.00E-20	BANK1	IDP dMRI TBSS ICVF Cerebral peduncle L	GCST90004342
rs7377083	6.00E-20	BANK1	Body mass index	GCST90446645
rs71621626	6.00E-20	SLC39A8	Body mass index	GCST90428119
rs2720460	7.00E-20	CENPE	Testicular germ cell tumor	GCST004635
rs151431	7.00E-20	BANK1,SLC39A8	Volume of right medial nucleus	GCST90475041
rs13107325	8.00E-20	SLC39A8	GOLM2 protein levels	GCST90469376
rs223423	9.00E-20	UBE2D3	Type 2 diabetes	GCST90492734
rs2272676	1.00E-19	NFKB1	Mean sphered cell volume (UKB data field 30270)	GCST90468089
rs13107325	1.00E-19	SLC39A8	wg lh intensity-contrast precuneus	GCST90003816
rs13105682	1.00E-19	BANK1	IDP T1 FAST ROIs L cerebellum IX	GCST90002584
rs13107325	1.00E-19	SLC39A8	aparc-DKTatlas rh thickness lateraloccipital	GCST90003580
rs3974481	1.00E-19	SLC9B1	Urea levels (UKB data field 30670)	GCST90468108
rs13107325	1.00E-19	SLC39A8	Standing height (UKB data field 50)	GCST90468178
rs13107325	1.00E-19	SLC39A8	Decaffeinated coffee consumption and/or lifetime cannabis use	GCST90134528
rs13135092	1.00E-19	SLC39A8	Right-hemisphere control network to hippocampus white-matter structural connectivity	GCST90302840
rs13107325	1.00E-19	SLC39A8	Cyclic AMP-dependent transcription factor ATF-6 alpha levels	GCST90246610
rs7377083	1.00E-19	BANK1	Body mass index	GCST90428119
rs13135092	2.00E-19	SLC39A8	High density lipoprotein cholesterol levels	GCST007140
rs230533	2.00E-19	NFKB1	Mean corpuscular volume	GCST90002338
rs13107325	2.00E-19	SLC39A8	Red blood cell count	GCST90018971
rs230527	2.00E-19	NFKB1	Mean corpuscular volume	GCST90018966
rs13105682	2.00E-19	BANK1	AmygNuclei lh volume Medial-nucleus	GCST90002653
rs13107325	2.00E-19	SLC39A8	BA-exvivo lh thickness V2	GCST90003523
rs13107325	2.00E-19	SLC39A8	IDP T1 FAST ROIs R cerebellum VI	GCST90002568
rs230540	2.00E-19	NFKB1	Reticulocyte count	GCST90002405
rs13107325	2.00E-19	SLC39A8	Albumin levels	GCST90027079
rs13107325	2.00E-19	SLC39A8	Calcium (minimum, inv-norm transformed)	GCST90479531
rs13107325	2.00E-19	SLC39A8	RGMA protein levels	GCST90470463
rs13107325	2.00E-19	SLC39A8	Cholesteryl esters in HDL (UKB data field 23418)	GCST90269515
rs13107325	2.00E-19	SLC39A8	HDL cholesterol levels (UKB data field 23406)	GCST90269503
rs13135092	2.00E-19	SLC39A8	Whole body fat mass (UKB data field 23100)	GCST90428121
rs13107325	2.00E-19	SLC39A8	Aspartate aminotransferase (AST, mean, inv-norm transformed)	GCST90475121
rs13107325	3.00E-19	SLC39A8	Waist-to-hip ratio adjusted for BMI	GCST009858
rs13107325	3.00E-19	SLC39A8	AmygNuclei lh volume Cortical-nucleus	GCST90002654
rs13107325	3.00E-19	SLC39A8	aparc-Desikan rh thickness lateraloccipital	GCST90003489
rs13107325	3.00E-19	SLC39A8	Obstructive sleep apnea (PheCode 327.32)	GCST90479998
rs13107325	3.00E-19	SLC39A8	Back pain (PheCode 760)	GCST90476266
rs13107325	4.00E-19	SLC39A8	Waist-to-hip ratio adjusted for BMI	GCST008994
rs13107325	4.00E-19	SLC39A8	Waist-to-hip ratio adjusted for BMI	GCST008995
rs13107325	4.00E-19	SLC39A8	IDP dMRI TBSS MO Cerebral peduncle L	GCST90003967
rs13105682	4.00E-19	BANK1	aseg lh intensity Pallidum	GCST90003771
rs13107325	4.00E-19	SLC39A8	Total lipid levels in medium HDL	GCST90092898
rs151410	4.00E-19	BANK1,SLC39A8	Anterior amygdaloid area volume	GCST90310230
rs13107325	4.00E-19	SLC39A8	Concentration of HDL particles	GCST90092826
rs13107325	4.00E-19	SLC39A8	Average diameter for HDL particles	GCST90092828
rs28625045	4.00E-19	BANK1	FUT8 protein levels	GCST90469281
rs13107325	4.00E-19	SLC39A8	PAPPA protein levels	GCST90470157
rs10028805	4.00E-19	BANK1	Systemic lupus erythematosus (MTAG)	GCST90270940
rs13107325	4.00E-19	SLC39A8	Osteoarthrosis NOS (PheCode 740.9)	GCST90480545
rs113473633	5.00E-19	NFKB1	Eosinophil counts	GCST90002302

rs13135092	5.00E-19	SLC39A8	HDL cholesterol x physical activity interaction (2df test)	GCST007282
rs13107325	5.00E-19	SLC39A8	aparc-Desikan rh thickness cuneus	GCST90003483
rs13107325	5.00E-19	SLC39A8	Homoarginine levels	GCST90245247
rs7377083	6.00E-19	BANK1	Childhood body mass index	GCST90301649
rs17199964	7.00E-19	BANK1	AmygNuclei lh volume Central-nucleus	GCST90002652
rs13105682	7.00E-19	BANK1	AmygNuclei rh volume Cortical-nucleus	GCST90002664
rs13107325	7.00E-19	SLC39A8	aparc-DKTatlas rh thickness cuneus	GCST90003574
rs13107325	7.00E-19	SLC39A8	Argininate levels	GCST90199900
rs13107325	8.00E-19	SLC39A8	Alcohol use disorder	GCST010545
rs13107325	8.00E-19	SLC39A8	aparc-DKTatlas lh thickness lateraloccipital	GCST90003549
rs13107325	8.00E-19	SLC39A8	FOLR2/THY1 protein level ratio	GCST90314867
rs978998	8.00E-19	SLC39A8,BANK1	Central nucleus volume	GCST90310229
rs223317	8.00E-19	CISD2	Estimated glomerular filtration rate (cystatin c)	GCST90103635
rs13107325	8.00E-19	SLC39A8	Osteoarthritis	GCST007093
rs13107325	8.00E-19	SLC39A8	SLITRK1 protein levels	GCST90470669
rs13105682	1.00E-18	BANK1	IDP dMRI TBSS ICVF Cerebral peduncle R	GCST90004341
rs13107325	1.00E-18	SLC39A8	aseg rh volume Pallidum	GCST90002640
rs13107325	1.00E-18	SLC39A8	IDP T1 FAST ROIs L cerebellum VI	GCST90002566
rs238449	1.00E-18	BANK1,SLC39A8	Central nucleus volume	GCST90310229
rs13107325	1.00E-18	SLC39A8	Hand grip strength left (UKB data field 46)	GCST90468168
rs13135092	1.00E-18	SLC39A8	Fornix volume	GCST90269832
rs181121136	1.00E-18	BANK1,SLC39A8	Volume of right medial nucleus	GCST90474925
rs13107325	1.00E-18	SLC39A8	Alcohol use disorder (MTAG)	GCST90296428
rs35518360	1.00E-18	SLC39A8,BANK1	red blood cell count (RBC, mean, inv-norm transformed)	GCST90476349
rs13107325	1.00E-18	SLC39A8	total cholesterol (minimum, inv-norm transformed)	GCST90480716
rs13107325	2.00E-18	SLC39A8	Brain region volumes	GCST009518
rs3774937	2.00E-18	NFKB1	seases (ankylosing spondylitis, Crohn's disease, psoriasis, primary sclerosing cholangitis, ulcera	GCST005537
rs13107325	2.00E-18	SLC39A8	aparc-Desikan lh thickness lateraloccipital	GCST90003455
rs17199964	2.00E-18	BANK1	Anterior amygdaloid area volume	GCST90310230
rs13107325	2.00E-18	SLC39A8	Cortical nucleus volume	GCST90310215
rs13107325	2.00E-18	SLC39A8	Triglyceride to HDL cholesterol ratio	GCST90295950
rs7674212	2.00E-18	SLC9B2	Impedance of arm left (UKB data field 23110)	GCST90468171
rs13107325	2.00E-18	SLC39A8	Edge-level brain connectivity (multivariate analysis)	GCST90165318
rs13107325	2.00E-18	SLC39A8	Educational attainment (years of education)	GCST006442
rs13107325	2.00E-18	SLC39A8	FOLR1 protein levels	GCST90469259
rs13107325	2.00E-18	SLC39A8	Height (baseline)	GCST90565843
rs755492124	2.00E-18	BANK1	CD40LG protein levels	GCST90468632
rs34592089	2.00E-18	BANK1	ttention deficit hyperactivity disorder or autism spectrum disorder or intelligence (pleiotropy	GCST90134330
rs13107325	2.00E-18	SLC39A8	HS3ST3B1 protein levels	GCST90469478
rs13107325	2.00E-18	SLC39A8	Left-hemisphere limbic network to caudate white-matter structural connectivity	GCST90302737
rs35518360	2.00E-18	SLC39A8,BANK1	Phospholipids in large HDL (UKB data field 23560)	GCST90269657
rs13107325	3.00E-18	SLC39A8	Intelligence (MTAG)	GCST005316
rs13107325	3.00E-18	SLC39A8	IDP dMRI TBSS MO Cerebral peduncle R	GCST90003966
rs13107325	3.00E-18	SLC39A8	Cognitive performance (MTAG)	GCST006570
rs13107325	3.00E-18	SLC39A8	Regional cortical thickness (lingual)	GCST90399887
rs13107325	3.00E-18	SLC39A8	Idiopathic knee osteoarthritis	GCST90297779
rs13107325	3.00E-18	SLC39A8	Osteoarthritis (MTAG)	GCST90570208
rs28625045	3.00E-18	BANK1	NID2 protein levels	GCST90470048
rs13107325	3.00E-18	SLC39A8	RETN protein levels	GCST90470457
rs13107325	3.00E-18	SLC39A8	red blood cell count (RBC, maximum, inv-norm transformed)	GCST90480668
rs13107325	4.00E-18	SLC39A8	ThalamNuclei lh volume VAmc	GCST90002723
rs13107325	4.00E-18	SLC39A8	Lateral nucleus volume	GCST90310218
rs13107325	4.00E-18	SLC39A8	ROR1 protein levels	GCST90470492
rs13107325	4.00E-18	SLC39A8	total cholesterol (mean, inv-norm transformed)	GCST90480715
rs13107325	5.00E-18	SLC39A8	Height	GCST90435412
rs28625045	5.00E-18	BANK1	TREML1 protein levels	GCST90470961
rs34406062	5.00E-18	MANBA,UBE2D3	Pallidum iron levels (quantitative susceptibility mapping)	GCST90551867
rs13107325	5.00E-18	SLC39A8	LYPD3 protein levels	GCST90469828
rs13107325	5.00E-18	SLC39A8	Free cholesterol in HDL (UKB data field 23422)	GCST90269519
rs755492124	6.00E-18	BANK1	SERPINE1 protein levels	GCST90470596
rs13107325	6.00E-18	SLC39A8	Total lipids in HDL (UKB data field 23426)	GCST90269523
rs35518360	6.00E-18	SLC39A8,BANK1	Total lipids in large HDL (UKB data field 23559)	GCST90269656
rs113473633	7.00E-18	NFKB1	Eosinophil counts	GCST90002381
rs223361	8.00E-18	UBE2D3	Diastolic blood pressure (MTAG)	GCST90449057
rs13126505	8.00E-18	BANK1	Fluid intelligence score (baseline)	GCST90565842
rs13107325	9.00E-18	SLC39A8	Lung function (FVC)	GCST007081
rs13107325	9.00E-18	SLC39A8	Voxel-wise structural brain imaging measurements	GCST007357
rs13107325	9.00E-18	SLC39A8	Triglyceride to HDL cholesterol ratio	GCST90295951
rs13107325	1.00E-17	SLC39A8	Systolic blood pressure	GCST007267
rs1813006	1.00E-17	BANK1,SLC39A8	Hemoglobin levels	GCST010083
rs141936164	1.00E-17	SLC39A8,NFKB1	Monocyte count	GCST90002393
rs13135092	1.00E-17	SLC39A8	Weight (UKB data field 21002)	GCST90468183
rs4637409	1.00E-17	BANK1	Systemic lupus erythematosus	GCST005752
rs13107325	1.00E-17	SLC39A8	Low density lipoprotein cholesterol levels	GCST90239655
rs13107325	1.00E-17	SLC39A8	Insomnia	GCST90131903
rs13107325	1.00E-17	SLC39A8	Cortical surface area	GCST90091060

rs13107325	1.00E-17	SLC39A8	Cortical thickness	GCST90091061
rs13107325	1.00E-17	SLC39A8	Osteoarthritis of the hip or knee	GCST90566799
rs13107325	1.00E-17	SLC39A8	Free cholesterol to total lipids in medium HDL percentage (UKB data field 23642)	GCST90269739
rs35518360	1.00E-17	SLC39A8,BANK1	Concentration of large HDL particles (UKB data field 23558)	GCST90269655
rs223481	1.00E-17	MANBA,UBE2D3	Type 2 diabetes (PheCode 250.2)	GCST90475667
rs13107325	1.00E-17	SLC39A8	hemoglobin (mean, inv-norm transformed)	GCST90475376
rs13107325	2.00E-17	SLC39A8	Diastolic blood pressure	GCST001228
rs13107325	2.00E-17	SLC39A8	Height	GCST90018959
rs13119516	2.00E-17	BANK1	Central nucleus volume	GCST90310229
rs13107325	2.00E-17	SLC39A8	Total concentration of lipoprotein particles	GCST90092990
rs223413	2.00E-17	UBE2D3	Diastolic blood pressure	GCST90132904
rs13107325	2.00E-17	SLC39A8	MCAM protein levels	GCST90469870
rs13107325	2.00E-17	SLC39A8	Risk-taking behavior (multivariate analysis)	GCST90239693
rs13107325	2.00E-17	SLC39A8	Volume of right accessory basal nucleus	GCST90475006
rs151415	2.00E-17	SLC39A8,BANK1	Volume of right cortical nucleus	GCST90475029
rs10017306	2.00E-17	SLC39A8	Volume of right central nucleus	GCST90474915
rs13107325	2.00E-17	SLC39A8	Cholesterol in medium HDL (UKB data field 23568)	GCST90269665
rs35225200	2.00E-17	BANK1,SLC39A8	Pain in joint (PheCode 745)	GCST90476262
rs13135092	2.00E-17	SLC39A8	Weight (minimum, inv-normal transformed)	GCST90480728
rs34333163	2.00E-17	SLC39A8	Serum albumin levels	GCST90019493
rs13105682	3.00E-17	BANK1	IDP dMRI TBSS OD Cerebral peduncle R	GCST90004416
rs13135092	3.00E-17	SLC39A8	ThalamNuclei rh volume PuI	GCST90002738
rs13107325	3.00E-17	SLC39A8	aparc-a2009s lh thickness G-cuneus	GCST90003613
rs13107325	3.00E-17	SLC39A8	Urate levels	GCST011119
rs13101632	3.00E-17	BANK1,SLC39A8	Anterior amygdaloid area volume	GCST90310230
rs12511373	3.00E-17	BANK1	High density lipoprotein cholesterol levels	GCST90239649
rs755492124	3.00E-17	BANK1	BGN protein levels	GCST90468441
rs13107325	3.00E-17	SLC39A8	Osteoarthritis	GCST90034520
rs755492124	3.00E-17	BANK1	VEGFC protein levels	GCST90471036
rs113473633	3.00E-17	NFKB1	Lymphocyte count	GCST90085815
rs13107325	3.00E-17	SLC39A8	PRTG protein levels	GCST90470347
rs28625045	3.00E-17	BANK1	SPARC protein levels	GCST90470718
rs13107325	3.00E-17	SLC39A8	Free cholesterol in medium HDL (UKB data field 23570)	GCST90269667
rs11446392	3.00E-17	SLC39A8	Volume of left central nucleus	GCST90474882
rs13107325	3.00E-17	SLC39A8	Cholesteryl esters in medium HDL (UKB data field 23569)	GCST90269666
rs13107325	3.00E-17	SLC39A8	Morbid obesity (PheCode 278.11)	GCST90479951
rs10028805	4.00E-17	BANK1	Systemic lupus erythematosus	GCST003155
rs13107325	4.00E-17	SLC39A8	Refractive error	GCST010002
rs223481	4.00E-17	MANBA,UBE2D3	Diabetes mellitus (PheCode 250)	GCST90475658
rs13107325	4.00E-17	SLC39A8	Osteoarthrosis, localized, primary (PheCode 740.11)	GCST90480543
rs10026360	5.00E-17	LINC02428	Height	GCST007841
rs13107325	5.00E-17	SLC39A8	Alcohol consumption (drinks per week)	GCST008811
rs151381	5.00E-17	BANK1,SLC39A8	Central nucleus volume	GCST90310229
rs13107325	5.00E-17	SLC39A8	T2 brain MRIs Unsupervised Deep learning derived Imaging Phenotypes (dimension 67)	GCST90320886
rs35518360	5.00E-17	SLC39A8,BANK1	Free cholesterol in large HDL (UKB data field 23563)	GCST90269660
rs13107325	5.00E-17	SLC39A8	What is your weight? (pounds, inv-normal transformed)	GCST90476469
rs230503	6.00E-17	NFKB1	High light scatter reticulocyte percentage of red cells	GCST90002386
rs71621626	6.00E-17	SLC39A8	Medial nucleus volume	GCST90310225
rs13107325	6.00E-17	SLC39A8	Osteoarthritis	GCST90134288
rs13107325	6.00E-17	SLC39A8	Volume of right accessory basal nucleus	GCST90475009
rs12510679	6.00E-17	SLC39A8	Volume of right medial nucleus	GCST90474924
rs13118152	6.00E-17	SLC39A8	Volume of right cortical nucleus	GCST90474918
rs13107325	7.00E-17	SLC39A8	Mean arterial pressure	GCST90018963
rs233821	7.00E-17	SLC39A8	Central nucleus volume	GCST90310229
rs13107325	7.00E-17	SLC39A8	Free cholesterol to total lipids ratio in very large HDL	GCST90093009
rs10017306	7.00E-17	SLC39A8	Volume of right medial nucleus	GCST90474925
rs2168805	7.00E-17	NFKB1,SLC39A8	monocyte (absolute count, minimum, inv-norm transformed)	GCST90479703
rs13107325	8.00E-17	SLC39A8	General cognitive ability	GCST006269
rs13105682	8.00E-17	BANK1	IDP T1 FAST ROIs L cerebellum VIIIb	GCST90002581
rs13135092	8.00E-17	SLC39A8	Verbal-numerical reasoning	GCST90011298
rs11097789	8.00E-17	NFKB1	Reticulocyte count (UKB data field 30250)	GCST90468100
rs1813006	8.00E-17	BANK1,SLC39A8	FUCA1 protein levels	GCST90469275
rs10017306	8.00E-17	SLC39A8	Volume of right central nucleus	GCST90474916
rs13107325	9.00E-17	SLC39A8	Phospholipid levels in medium HDL	GCST90092900
rs35518360	9.00E-17	SLC39A8,BANK1	Phospholipids to total lipids ratio in large VLDL	GCST90092877
rs223331	9.00E-17	CISD2	Impedance of arm right (UKB data field 23109)	GCST90468172
rs13107325	9.00E-17	SLC39A8	Bifunctional heparan sulfate N-deacetylase/N-sulfotransferase 1 levels	GCST90248628
rs13107325	9.00E-17	SLC39A8	Phospholipids in HDL (UKB data field 23414)	GCST90269511
rs13107325	9.00E-17	SLC39A8	hematocrit (mean, inv-norm transformed)	GCST90479629
rs13107325	1.00E-16	SLC39A8	Red blood cell count	GCST90002363
rs2720460	1.00E-16	CENPE	Testicular germ cell tumor	GCST004713
rs7695096	1.00E-16	SLC9B1	Type 2 diabetes	GCST010555
rs13107325	1.00E-16	SLC39A8	Regional cortical thickness (transversetemporal)	GCST90399910
rs13107325	1.00E-16	SLC39A8	Schizophrenia	GCST006803
rs13107325	1.00E-16	SLC39A8	ADGRG2 protein levels	GCST90468241
rs13107325	1.00E-16	SLC39A8	SELE protein levels	GCST90470566

rs35518360	1.00E-16	SLC39A8,BANK1	Cholesterol in large HDL (UKB data field 23561)	GCST90269658
rs13107325	1.00E-16	SLC39A8	Systolic blood pressure	GCST90301695
rs223426	1.00E-16	UBE2D3	creatinine (mean, inv-norm transformed)	GCST90479562
rs13107325	1.00E-16	SLC39A8	Calcium (maximum, inv-norm transformed)	GCST90475173
rs113473633	2.00E-16	NFKB1	Lymphocyte count	GCST90002388
rs230511	2.00E-16	NFKB1	Mean corpuscular volume	GCST90002392
rs13107325	2.00E-16	SLC39A8	Total cholesterol levels in very large HDL	GCST90302126
rs13107325	2.00E-16	SLC39A8	aparc-DKTatlas rh thickness lingual	GCST90003582
rs151134704	2.00E-16	NFKB1	Serum uric acid levels	GCST007918
rs151134704	2.00E-16	NFKB1	Estimated glomerular filtration rate	GCST007917
rs151134704	2.00E-16	NFKB1	Creatinine levels	GCST007919
rs179195	2.00E-16	MANBA	Alkaline phosphatase (UKB data field 30610)	GCST90468060
rs13107325	2.00E-16	SLC39A8	Regional cortical thickness (precentral)	GCST90399900
rs13107325	2.00E-16	SLC39A8	Decaffeinated coffee consumption or major depression disorder	GCST90134529
rs13107325	2.00E-16	SLC39A8	Decaffeinated coffee consumption and/or neuroticism	GCST90134530
rs13107325	2.00E-16	SLC39A8	Decaffeinated coffee consumption and/or insomnia	GCST90134531
rs35518360	2.00E-16	SLC39A8,BANK1	Cholesteryl esters to total lipids ratio in IDL	GCST90092834
rs13107325	2.00E-16	SLC39A8	FLT1 protein levels	GCST90469249
rs13107325	2.00E-16	SLC39A8	ADAMTSL4 protein levels	GCST90468229
rs13107325	2.00E-16	SLC39A8	ITGAM protein levels	GCST90469638
rs34406062	2.00E-16	MANBA,UBE2D3	Substantia nigra iron levels (quantitative susceptibility mapping)	GCST90551871
rs11941622	2.00E-16	BANK1,SLC39A8	Volume of right central nucleus	GCST90474916
rs13107325	2.00E-16	SLC39A8	Apolipoprotein A1 levels (UKB data field 23440)	GCST90269537
rs13107325	2.00E-16	SLC39A8	Cholesteryl esters in large HDL (UKB data field 23562)	GCST90269659
rs11726195	2.00E-16	NFKB1,MANBA	lymphocyte (fraction, mean, inv-norm transformed)	GCST90479667
rs1585215	2.00E-16	NFKB1	monocyte (fraction, minimum, inv-norm transformed)	GCST90479706
rs13107325	2.00E-16	SLC39A8	Aspartate aminotransferase (AST, minimum, inv-norm transformed)	GCST90479511
rs13135092	3.00E-16	SLC39A8	Insomnia	GCST007988
rs113473633	3.00E-16	NFKB1	White blood cell count	GCST90002374
rs34592089	3.00E-16	BANK1	General cognitive ability	GCST006269
rs13107325	3.00E-16	SLC39A8	CLEC5A protein levels	GCST90468776
rs13107325	3.00E-16	SLC39A8	ThalamNuclei lh volume PuI	GCST90002714
rs13107325	3.00E-16	SLC39A8	aparc-Desikan lh thickness cuneus	GCST90003449
rs17199964	3.00E-16	BANK1	IDP T1 FAST ROIs R cerebellum VIIIb	GCST90002583
rs13107325	3.00E-16	SLC39A8	aparc-Desikan rh thickness lingual	GCST90003491
rs2216546	3.00E-16	BANK1	APRT/MIF protein level ratio	GCST90313336
rs13126505	3.00E-16	BANK1	Cognitive aspects of educational attainment	GCST90011875
rs10856963	3.00E-16	BANK1	CCL5 protein levels	GCST90468584
rs11097755	3.00E-16	BANK1	Impedance of whole body (UKB data field 23106)	GCST90468173
rs34166099	3.00E-16	BANK1	Diastolic blood pressure (MTAG)	GCST90449057
rs13107325	3.00E-16	SLC39A8	Spinal stenosis of lumbar region (PheCode 720.1)	GCST90476234
rs13107325	4.00E-16	SLC39A8	Body mass index	GCST005951
rs13107325	4.00E-16	SLC39A8	Free cholesterol in very large HDL	GCST90302130
rs12511373	4.00E-16	BANK1	Central nucleus volume	GCST90310229
rs223489	4.00E-16	MANBA	ex hormone-binding globulin levels adjusted for BMI and heel estimated bone mineral densit	GCST90399398
rs181121136	4.00E-16	BANK1,SLC39A8	Volume of right cortical nucleus	GCST90474919
rs13107325	4.00E-16	SLC39A8	Concentration of medium HDL particles (UKB data field 23565)	GCST90269662
rs6855246	4.00E-16	SLC39A8,BANK1	Pain in joint (PheCode 745)	GCST90480554
rs13107325	4.00E-16	SLC39A8	triglyceride (mean, inv-norm transformed)	GCST90480718
rs113473633	5.00E-16	NFKB1	Eosinophil counts	GCST007065
rs980455	5.00E-16	NFKB1,SLC39A8	Monocyte count (UKB data field 30130)	GCST90468090
rs13107325	5.00E-16	SLC39A8	Sphingomyelin levels	GCST90092982
rs28625045	5.00E-16	BANK1	CCN2 protein levels	GCST90468588
rs114087689	5.00E-16	CENPE	Testosterone levels	GCST90483498
rs13107325	5.00E-16	SLC39A8	Pain intensity (without opioid use disorder)	GCST90565152
rs13126505	5.00E-16	BANK1	Body mass index	GCST90428119
rs13126505	6.00E-16	BANK1	Intelligence	GCST006250
rs13107325	6.00E-16	SLC39A8	aparc-DKTatlas lh thickness cuneus	GCST90003543
rs13107325	6.00E-16	SLC39A8	Hypertension	GCST90468135
rs7674212	6.00E-16	SLC9B2	Impedance of whole body (UKB data field 23106)	GCST90468173
rs13107325	6.00E-16	SLC39A8	Albumin (mean, inv-norm transformed)	GCST90475103
rs13107325	6.00E-16	SLC39A8	Back pain (PheCode 760)	GCST90480570
rs13127398	7.00E-16	BANK1	Cardiovascular disease	GCST007072
rs6533052	7.00E-16	SLC9B1	Multiple sclerosis	GCST009597
rs1516740	7.00E-16	LINC02428	Estimated glomerular filtration rate (creatinine)	GCST90100220
rs230540	7.00E-16	NFKB1	Reticulocyte fraction of red cells	GCST90002406
rs13107325	7.00E-16	SLC39A8	Phospholipids to total lipids ratio in very small VLDL	GCST90093037
rs10016018	7.00E-16	BANK1	SERPINE2 protein levels	GCST90470597
rs11446392	7.00E-16	SLC39A8	Volume of right central nucleus	GCST90475026
rs150228862	7.00E-16	BANK1	Volume of right medial nucleus	GCST90474925
rs13135092	8.00E-16	SLC39A8	Central nucleus volume	GCST90310222
rs13143234	8.00E-16	SLC39A8	Accessory basal nucleus volume	GCST90310224
rs13107325	8.00E-16	SLC39A8	Serum urate levels	GCST90455669
rs13107325	8.00E-16	SLC39A8	Volume of right whole amygdala	GCST90474931
rs2168805	8.00E-16	NFKB1,SLC39A8	monocyte (absolute count, mean, inv-norm transformed)	GCST90479702
rs17266357	9.00E-16	BANK1	Calcineurin levels	GCST90100761

rs13127398	9.00E-16	BANK1	Insomnia	GCST90131901
rs13135092	9.00E-16	SLC39A8	Osteoarthritis	GCST90134286
rs35518360	9.00E-16	SLC39A8,BANK1	hemoglobin (mean, inv-norm transformed)	GCST90479639
rs457134	1.00E-15	BANK1,SLC39A8	Body mass index	GCST009871
rs12509403	1.00E-15	NFKB1	Allergic rhinitis	GCST006409
rs1585213	1.00E-15	NFKB1	Non-albumin protein levels	GCST005990
rs13107325	1.00E-15	SLC39A8	Systolic blood pressure x alcohol consumption interaction (2df test)	GCST006434
rs13107325	1.00E-15	SLC39A8	HDL cholesterol	GCST002223
rs34592089	1.00E-15	BANK1	Crohn's disease	GCST003044
rs13107325	1.00E-15	SLC39A8	Cholesterol esters in very large HDL	GCST90302128
rs13107325	1.00E-15	SLC39A8	CHGB protein levels	GCST90468742
rs13107325	1.00E-15	SLC39A8	Serum uric acid levels	GCST90018977
rs13107325	1.00E-15	SLC39A8	ThalamNuclei rh volume VAmc	GCST90002747
rs13107325	1.00E-15	SLC39A8	wg lh intensity-contrast isthmuscingulate	GCST90003801
rs13107325	1.00E-15	SLC39A8	Phospholipids to total lipids ratio in medium HDL	GCST90092901
rs13107325	1.00E-15	SLC39A8	Cholesteryl ester levels in very large HDL	GCST90093006
rs13107325	1.00E-15	SLC39A8	Regional cortical thickness (isthmuscingulate)	GCST90399884
rs13107325	1.00E-15	SLC39A8	Alcohol consumption	GCST008757
rs13107325	1.00E-15	SLC39A8	QPCT protein levels	GCST90470401
rs57957741	1.00E-15	BANK1,SLC39A8	Educational attainment	GCST90105038
rs13107325	1.00E-15	SLC39A8	Pain intensity (without opioid use disorder)	GCST90565151
rs10017306	1.00E-15	SLC39A8	Volume of right medial nucleus	GCST90474924
rs11941622	1.00E-15	BANK1,SLC39A8	Volume of right medial nucleus	GCST90474925
rs13107325	1.00E-15	SLC39A8	Pain intensity (NRS pain scale 0-11)	GCST90565148
rs13107325	1.00E-15	SLC39A8	ight-hemisphere default mode network to hippocampus white-matter structural connectivit	GCST90302848
rs13107325	1.00E-15	SLC39A8	Thoracic or lumbosacral neuritis or radiculitis, unspecified (PheCode 763)	GCST90476268
rs35518360	1.00E-15	SLC39A8,BANK1	red blood cell count (RBC, mean, inv-norm transformed)	GCST90480669
rs13107325	1.00E-15	SLC39A8	high density lipoprotein cholesterol (HDLC, mean, inv-norm transformed)	GCST90475349
rs13107325	2.00E-15	SLC39A8	Brain region volumes	GCST009518
rs34061204	2.00E-15	BANK1	Mean platelet volume	GCST004599
rs173048	2.00E-15	SLC39A8	Body mass index	GCST009871
rs13107325	2.00E-15	SLC39A8	Monocyte percentage (UKB data field 30190)	GCST90468091
rs13107325	2.00E-15	SLC39A8	AmygNuclei rh volume Accessory-Basal-nucleus	GCST90002660
rs13107325	2.00E-15	SLC39A8	OMD/SPP1 protein level ratio	GCST90315586
rs6822371	2.00E-15	SLC39A8	Central nucleus volume	GCST90310229
rs13105682	2.00E-15	BANK1	Medial nucleus volume	GCST90310225
rs13107325	2.00E-15	SLC39A8	Global cortical thickness (GlobalMeanMean)	GCST90399875
rs11097786	2.00E-15	NFKB1,SLC39A8	CA3 protein levels	GCST90468512
rs13107325	2.00E-15	SLC39A8	ANPEP protein levels	GCST90468313
rs13107325	2.00E-15	SLC39A8	GALNT5 protein levels	GCST90469298
rs223485	2.00E-15	MANBA,UBE2D3	GPC1 protein levels	GCST90469386
rs3974481	2.00E-15	SLC9B1	Liver enzyme levels (alkaline phosphatase)	GCST90013406
rs230501	2.00E-15	NFKB1	GZMA protein levels	GCST90469423
rs4572884	2.00E-15	BANK1	PF4 protein levels	GCST90470211
rs13126505	2.00E-15	BANK1	Hand grip strength (baseline)	GCST90565845
rs13107325	2.00E-15	SLC39A8	Mean volume of bilateral amygdala	GCST90474901
rs13107325	2.00E-15	SLC39A8	Left-hemisphere limbic network to hippocampus white-matter structural connectivity	GCST90302740
rs143148372	3.00E-15	SLC9B1	Haematocrit percentage (UKB data field 30030)	GCST90468073
rs13107325	3.00E-15	SLC39A8	wg lh intensity-contrast entorhinal	GCST90003797
rs13135092	3.00E-15	SLC39A8	aparc-a2009s lh thickness Pole-occipital	GCST90003644
rs13107325	3.00E-15	SLC39A8	SLITRK6 protein levels	GCST90470671
rs13119968	3.00E-15	SLC39A8	Volume of right medial nucleus	GCST90475041
rs13107325	3.00E-15	SLC39A8	Pain intensity (NRS pain scale 0-11)	GCST90565147
rs223481	3.00E-15	MANBA,UBE2D3	estimated glomerular filtration rate (eGFR, mean, inv-norm transformed)	GCST90479599
rs13107325	3.00E-15	SLC39A8	Albumin (maximum, inv-norm transformed)	GCST90475100
rs531685993	4.00E-15	NFKB1,SLC39A8	Brain region volumes	GCST009518
rs230509	4.00E-15	NFKB1	Mean spheric corpuscular volume	GCST90002397
rs13105682	4.00E-15	BANK1	IDP dMRI TBSS OD Cerebral peduncle L	GCST90004417
rs10026360	4.00E-15	LINC02428	Standing height (UKB data field 50)	GCST90468178
rs28625045	4.00E-15	BANK1	TIMP3 protein levels	GCST90470871
rs13107325	4.00E-15	SLC39A8	HGF protein levels	GCST90469449
rs10016018	4.00E-15	BANK1	SPINT2 protein levels	GCST90470728
rs35518360	4.00E-15	SLC39A8,BANK1	Average diameter for HDL particles (UKB data field 23433)	GCST90269530
rs13107325	4.00E-15	SLC39A8	Osteoarthritis (hip)	GCST90566798
rs13107325	4.00E-15	SLC39A8	Pain intensity (NRS pain scale 0-11)	GCST90565160
rs150228862	4.00E-15	BANK1	Volume of right central nucleus	GCST90474916
rs223426	4.00E-15	UBE2D3	creatinine (minimum, inv-norm transformed)	GCST90479563
rs13135092	5.00E-15	SLC39A8	Alcohol use disorder (dependence and problematic use scores)	GCST006717
rs13107325	5.00E-15	SLC39A8	Osteoarthritis	GCST90468144
rs230534	5.00E-15	NFKB1	Primary biliary cholangitis	GCST90061441
rs34316881	5.00E-15	SLC39A8,NFKB1	Thalamus volume	GCST012009
rs35518360	5.00E-15	SLC39A8,BANK1	Phospholipids to total lipids in large VLDL percentage (UKB data field 23589)	GCST90269686
rs11726195	5.00E-15	NFKB1,MANBA	lymphocyte (absolute count, maximum, inv-norm transformed)	GCST90479663
rs6839635	5.00E-15	SLC9B1	Glucose (fasting status unknown, mean, inv-norm transformed)	GCST90479626
rs13107325	5.00E-15	SLC39A8	high density lipoprotein cholesterol (HDLC, maximum, inv-norm transformed)	GCST90475345
rs13107325	6.00E-15	SLC39A8	Triglyceride levels	GCST010244

rs17032705	6.00E-15	NFKB1	TNIP1 protein levels	GCST90470925
rs28545534	6.00E-15	SLC9B2	Bone mineral density mean	GCST90321120
rs13107325	7.00E-15	SLC39A8	Low density lipoprotein cholesterol levels	GCST90239658
rs35518360	7.00E-15	SLC39A8,BANK1	Physical function (baseline)	GCST90565837
rs13107325	7.00E-15	SLC39A8	Maximum habitual alcohol consumption	GCST90244186
rs13107325	7.00E-15	SLC39A8	Sleep apnea	GCST90475257
rs35518360	8.00E-15	SLC39A8,BANK1	Schizophrenia	GCST002539
rs74332078	8.00E-15	UBE2D3	High density lipoprotein cholesterol levels	GCST90239649
rs13107325	8.00E-15	SLC39A8	Triglycerides to total lipids ratio in IDL	GCST90092842
rs13107325	8.00E-15	SLC39A8	NT5E protein levels	GCST90470093
rs13107325	8.00E-15	SLC39A8	Volume of left accessory basal nucleus	GCST90474940
rs201081507	8.00E-15	BANK1	Volume of right medial nucleus	GCST90474924
rs13107325	8.00E-15	SLC39A8	Spinal stenosis of lumbar region (PheCode 720.1)	GCST90480506
rs13107325	9.00E-15	SLC39A8	Cholesteryl esters to total lipids ratio in very small VLDL	GCST90093031
rs13107325	9.00E-15	SLC39A8	Maximum habitual alcohol consumption	GCST90244185
rs13107325	9.00E-15	SLC39A8	Total lipids in medium HDL (UKB data field 23566)	GCST90269663
rs230501	1.00E-14	NFKB1	Neutrophil percentage of white cells	GCST004633
rs7659468	1.00E-14	SLC9B1	Type 2 diabetes	GCST010557
rs6533030	1.00E-14	SLC9B1	Serum alkaline phosphatase levels	GCST90018942
rs3974479	1.00E-14	SLC9B1	Creatinine levels	GCST90092815
rs3733197	1.00E-14	BANK1	BANK1 protein levels	GCST90468421
rs34955446	1.00E-14	SLC9B2	Caudate iron levels (quantitative susceptibility mapping)	GCST90551863
rs13135092	1.00E-14	SLC39A8	Asthma or gastroesophageal reflux disease (MTAG)	GCST90570611
rs13118152	1.00E-14	SLC39A8	Volume of right medial nucleus	GCST90475041
rs2126627	1.00E-14	NFKB1,SLC39A8	Volume of right central nucleus	GCST90474916
rs13107325	1.00E-14	SLC39A8	Systolic blood pressure	GCST90472781
rs34592089	1.00E-14	BANK1	Intelligence	GCST90264174
rs35225200	1.00E-14	BANK1,SLC39A8	Left-hemisphere default mode network to hippocampus white-matter structural connectivity	GCST90302771
rs1585215	1.00E-14	NFKB1	monocyte (fraction, mean, inv-norm transformed)	GCST90479705
rs13107325	2.00E-14	SLC39A8	Red blood cell count	GCST90002367
rs6855246	2.00E-14	SLC39A8,BANK1	General cognitive ability	GCST006269
rs13107325	2.00E-14	SLC39A8	Diastolic blood pressure	GCST006258
rs13107325	2.00E-14	SLC39A8	Total lipids in very large HDL	GCST90302132
rs13107325	2.00E-14	SLC39A8	HAVCR2/TNFRSF1B protein level ratio	GCST90315030
rs13107325	2.00E-14	SLC39A8	Triglycerides to total lipids ratio in small HDL	GCST90092955
rs13107325	2.00E-14	SLC39A8	Triglyceride levels (UKB data field 30870)	GCST90468106
rs13107325	2.00E-14	SLC39A8	Triglycerides	GCST90020235
rs13107325	2.00E-14	SLC39A8	Gastroesophageal reflux disease	GCST90000514
rs35518360	2.00E-14	SLC39A8,BANK1	Cholesterol to total lipids ratio in IDL	GCST90092832
rs201081507	2.00E-14	BANK1	High-density lipoprotein levels (MTAG)	GCST90179147
rs13107325	2.00E-14	SLC39A8	Calcium (maximum, inv-norm transformed)	GCST90479529
rs13107325	2.00E-14	SLC39A8	Gastroesophageal reflux disease or schizophrenia (pleiotropy)	GCST90271339
rs35166705	2.00E-14	SLC39A8,BANK1	Volume of right central nucleus	GCST90475023
rs6816787	2.00E-14	BANK1	Systemic lupus erythematosus (MTAG)	GCST90270940
rs13107325	2.00E-14	SLC39A8	Gamma glutamyl transferase levels	GCST90428730
rs34333163	2.00E-14	SLC39A8	Whole body fat mass (UKB data field 23100)	GCST90428121
rs11726195	2.00E-14	NFKB1,MANBA	lymphocyte (absolute count, mean, inv-norm transformed)	GCST90479664
rs3733202	2.00E-14	MANBA	neutrophil (fraction, minimum, inv-norm transformed)	GCST90479715
rs13135092	2.00E-14	SLC39A8	high density lipoprotein cholesterol (HDLC, minimm, inv-norm transformed)	GCST90475353
rs13107325	2.00E-14	SLC39A8	triglyceride (maximum, inv-norm transformed)	GCST90480717
rs13107325	3.00E-14	SLC39A8	Systolic blood pressure	GCST001227
rs34654981	3.00E-14	UBE2D3,UBE2D3-AS1	Blood urea nitrogen levels	GCST90018948
rs755492124	3.00E-14	BANK1	COL2A1 protein levels	GCST90468816
rs13135092	3.00E-14	SLC39A8	Alcohol use disorder (total score)	GCST006716
rs13107325	3.00E-14	SLC39A8	BA-exvivo rh thickness BA4a	GCST90003531
rs13135092	3.00E-14	SLC39A8	Multisite chronic pain	GCST012332
rs13107325	3.00E-14	SLC39A8	Waist-hip index	GCST90020027
rs13135092	3.00E-14	SLC39A8	Bitter alcoholic beverage consumption	GCST008522
rs13107325	3.00E-14	SLC39A8	Alcohol use disorder	GCST008259
rs13107325	3.00E-14	SLC39A8	Alcohol use disorder	GCST008259
rs34333163	3.00E-14	SLC39A8	Insomnia	GCST90131901
rs13107325	3.00E-14	SLC39A8	ISM1 protein levels	GCST90469630
rs13107325	3.00E-14	SLC39A8	SEMA7A protein levels	GCST90470575
rs13107325	3.00E-14	SLC39A8	MANSC1 protein levels	GCST90469847
rs13107325	3.00E-14	SLC39A8	PGLYRP1 protein levels	GCST90470219
rs13107325	3.00E-14	SLC39A8	NTRK2 protein levels	GCST90470097
rs13107325	3.00E-14	SLC39A8	Pain intensity (NRS pain scale 0-11)	GCST90565159
rs13107325	3.00E-14	SLC39A8	Concentration of HDL particles (UKB data field 23430)	GCST90269527
rs13107325	4.00E-14	SLC39A8	DSG2 protein levels	GCST90469042
rs13107325	4.00E-14	SLC39A8	aparc-DKTatlas rh thickness pericalcarine	GCST90003590
rs13107325	4.00E-14	SLC39A8	aparc-Desikan rh thickness pericalcarine	GCST90003499
rs13107325	4.00E-14	SLC39A8	BA-exvivo lh thickness BA4a	GCST90003517
rs13107325	4.00E-14	SLC39A8	Alcohol use disorder (consumption score)	GCST012336
rs13107325	4.00E-14	SLC39A8	Cholesterol levels in very large HDL	GCST90093004
rs13107325	4.00E-14	SLC39A8	Concentration of very large HDL particles	GCST90093011
rs13107325	4.00E-14	SLC39A8	Protein SET levels	GCST90249505

rs13118152	4.00E-14	SLC39A8	Volume of right central nucleus	GCST90475023
rs13109272	4.00E-14	SLC39A8	Intelligence	GCST90264174
rs13107325	4.00E-14	SLC39A8	systolic blood pressure (SBP, maximum, inv-normal transformed)	GCST90480705
rs230504	5.00E-14	NFKB1	Eczema	GCST007075
rs3774937	5.00E-14	NFKB1	Ulcerative colitis	GCST003045
rs230523	5.00E-14	NFKB1	Tonsillectomy	GCST003995
rs230523	5.00E-14	NFKB1	Tonsillectomy	GCST005014
rs13107325	5.00E-14	SLC39A8	Left-handedness	GCST90013421
rs13105682	5.00E-14	BANK1	IDP T1 FAST ROIs L cerebellum I-IV	GCST90002562
rs11097789	5.00E-14	NFKB1	Reticulocyte percentage (UKB data field 30240)	GCST90468101
rs13107325	5.00E-14	SLC39A8	AOC3 protein levels	GCST90468322
rs74332078	5.00E-14	UBE2D3	Pallidum iron levels (R2* MRI)	GCST90551868
rs63519	5.00E-14	SLC39A8	Volume of right medial nucleus	GCST90475044
rs7664171	5.00E-14	MANBA,NFKB1	Lymphocyte side fluorescence distribution width	GCST90281250
rs6822371	5.00E-14	SLC39A8	Body mass index	GCST90255621
rs750527033	6.00E-14	UBE2D3,MANBA	Glycated haemoglobin HbA1c levels (UKB data field 30750)	GCST90468072
rs13107325	6.00E-14	SLC39A8	DPEP1 protein levels	GCST90469030
rs13107325	6.00E-14	SLC39A8	Cholesterol to total lipids ratio in medium HDL	GCST90092893
rs13118152	6.00E-14	SLC39A8	Volume of right cortical nucleus	GCST90475029
rs1813006	6.00E-14	BANK1,SLC39A8	Whole body fat mass (UKB data field 23100)	GCST90428121
rs13107325	6.00E-14	SLC39A8	height (mean, inv-normal transformed)	GCST90479635
rs13107325	6.00E-14	SLC39A8	Albumin (maximum, inv-norm transformed)	GCST90479503
rs13107325	6.00E-14	SLC39A8	Albumin (mean, inv-norm transformed)	GCST90479504
rs223502	6.00E-14	MANBA	Creatinine levels	GCST90019502
rs13107325	7.00E-14	SLC39A8	Brain region volumes	GCST009518
rs13107325	7.00E-14	SLC39A8	LAG3 protein levels	GCST90469727
rs13118152	7.00E-14	SLC39A8	Volume of left central nucleus	GCST90474882
rs34491518	7.00E-14	UBE2D3	alpha-Fetoprotein levels	GCST90278615
rs71621626	7.00E-14	SLC39A8	Whole body fat mass (UKB data field 23100)	GCST90428121
rs223502	7.00E-14	MANBA	Cystatin C levels	GCST90019504
rs7665090	8.00E-14	MANBA,NFKB1	Primary biliary cirrhosis	GCST005581
rs13107325	8.00E-14	SLC39A8	IDP dMRI TBSS ICVF Anterior limb of internal capsule L	GCST90004344
rs13107325	8.00E-14	SLC39A8	IDP T1 FAST ROIs R caudate	GCST90002550
rs13107325	8.00E-14	SLC39A8	Liver enzyme levels (gamma-glutamyl transferase)	GCST90013407
rs7692921	8.00E-14	SLC39A8	SDC1 protein levels	GCST90470558
rs13107325	8.00E-14	SLC39A8	Height (maximum, inv-normal transformed)	GCST90479634
rs13107325	8.00E-14	SLC39A8	total cholesterol (maximum, inv-norm transformed)	GCST90480714
rs6855246	9.00E-14	SLC39A8,BANK1	Insomnia	GCST007988
rs13107325	9.00E-14	SLC39A8	Total lipid levels in very large HDL	GCST90093010
rs201081507	9.00E-14	BANK1	Body mass index	GCST90428119
rs13107325	1.00E-13	SLC39A8	Diastolic blood pressure	GCST004777
rs13105682	1.00E-13	BANK1	IDP SWI T2star right caudate	GCST90003866
rs13107325	1.00E-13	SLC39A8	wg rh intensity-contrast isthmuscingulate	GCST90003836
rs17032400	1.00E-13	SLC39A8	Central nucleus volume	GCST90310229
rs13107325	1.00E-13	SLC39A8	Forced vital capacity FVC Z score (UKB data field 20257)	GCST90468167
rs13135092	1.00E-13	SLC39A8	Brain age (difference between predicted and chronological age)	GCST90269833
rs223420	1.00E-13	UBE2D3	Type 2 diabetes and Attention deficit hiperactivty disorder or obsessive compulsive disorder	GCST90624675
rs201081507	1.00E-13	BANK1	Body mass index or osteoarthritis (pleiotropy)	GCST90271767
rs13107325	1.00E-13	SLC39A8	Free cholesterol to total lipids in very large HDL percentage (UKB data field 23632)	GCST90269729
rs13107325	1.00E-13	SLC39A8	Fluid intelligence	GCST90448240
rs34592089	1.00E-13	BANK1	Whole body fat mass (UKB data field 23100)	GCST90428121
rs7681002	2.00E-13	SLC9B1	Cardiovascular disease	GCST007072
rs1813006	2.00E-13	BANK1,SLC39A8	Intelligence (MTAG)	GCST005316
rs13107325	2.00E-13	SLC39A8	Body mass index	GCST000830
rs13107325	2.00E-13	SLC39A8	CTSZ protein levels	GCST90468918
rs13107325	2.00E-13	SLC39A8	ThalamNuclei rh volume MGN	GCST90002737
rs13105682	2.00E-13	BANK1	IDP T1 FAST ROIs V cerebellum VIIIb	GCST90002582
rs13107325	2.00E-13	SLC39A8	aparc-a2009s rh thickness G-oc-temp-med-Lingual	GCST90003698
rs13135092	2.00E-13	SLC39A8	Multisite chronic pain	GCST008512
rs13107325	2.00E-13	SLC39A8	Phospholipid levels in very large HDL	GCST90093012
rs13107325	2.00E-13	SLC39A8	Waist circumference adjusted for body mass index	GCST90020029
rs13107325	2.00E-13	SLC39A8	PLA2G15 protein levels	GCST90470245
rs577329140	2.00E-13	NFKB1,SLC39A8	Volume of left central nucleus	GCST90474882
rs10017306	2.00E-13	SLC39A8	Volume of left central nucleus	GCST90474882
rs10017306	2.00E-13	SLC39A8	Volume of left central nucleus	GCST90474883
rs62688361	2.00E-13	BANK1	Volume of left medial nucleus	GCST90474892
rs181121136	2.00E-13	BANK1,SLC39A8	Volume of left medial nucleus	GCST90474892
rs238449	2.00E-13	BANK1,SLC39A8	Volume of right medial nucleus	GCST90474924
rs11941622	2.00E-13	BANK1,SLC39A8	Volume of right cortical nucleus	GCST90474919
rs2866414	2.00E-13	MANBA	Systemic lupus erythematosus (MTAG)	GCST90270940
rs146171187	2.00E-13	SLC39A8	Body mass index	GCST90255621
rs13107325	2.00E-13	SLC39A8	Superficial cellulitis and abscess (PheCode 681)	GCST90476165
rs223481	2.00E-13	MANBA,UBE2D3	estimated glomerular filtration rate (eGFR, maximum, inv-norm transformed)	GCST90479598
rs223502	2.00E-13	MANBA	Estimated glomerular filtration rate	GCST90019506
rs34592089	3.00E-13	BANK1	Brain region volumes	GCST009518
rs531685993	3.00E-13	NFKB1,SLC39A8	Brain region volumes	GCST009518

rs13135092	3.00E-13	SLC39A8	HDL cholesterol	GCST002899
rs113473633	3.00E-13	NFKB1	White blood cell count	GCST90002378
rs10013613	3.00E-13	NFKB1	Basophil percentage of white cells	GCST90002380
rs980455	3.00E-13	NFKB1,SLC39A8	Monocyte count	GCST90018967
rs13107325	3.00E-13	SLC39A8	wg rh intensity-contrast entorhinal	GCST90003832
rs13107325	3.00E-13	SLC39A8	aparc-DKTatlas rh thickness paracentral	GCST90003586
rs13107325	3.00E-13	SLC39A8	aparc-Desikan rh thickness paracentral	GCST90003495
rs13107325	3.00E-13	SLC39A8	Sleep duration (short sleep)	GCST007559
rs34592089	3.00E-13	BANK1	Dorsolateral prefrontal thickness	GCST90572705
rs13107325	3.00E-13	SLC39A8	Ratio of triglycerides to phosphoglycerides	GCST90092983
rs13107325	3.00E-13	SLC39A8	Cholesterol to total lipids ratio in very small VLDL	GCST90093029
rs13107325	3.00E-13	SLC39A8	Regional cortical thickness (pericalcarine)	GCST90399897
rs13135092	3.00E-13	SLC39A8	Cognitive processing accuracy	GCST90446169
rs13135092	3.00E-13	SLC39A8	TNC protein levels	GCST90470895
rs1813006	3.00E-13	BANK1,SLC39A8	ACAN protein levels	GCST90468196
rs13107325	3.00E-13	SLC39A8	LCN2 protein levels	GCST90469747
rs188539658	3.00E-13	MANBA,UBE2D3	Testosterone levels	GCST90483487
rs13135092	3.00E-13	SLC39A8	Albumin levels	GCST90027079
rs2851245	3.00E-13	BANK1,SLC39A8	Volume of right central nucleus	GCST90474915
rs181121136	3.00E-13	BANK1,SLC39A8	Volume of right central nucleus	GCST90474916
rs28396651	3.00E-13	BANK1	Platelet forward scatter distribution width	GCST90281197
rs13135092	3.00E-13	SLC39A8	Erectile dysfunction [ED] (PheCode 605)	GCST90476161
rs13107325	3.00E-13	SLC39A8	Diaphragmatic hernia (PheCode 550.2)	GCST90476061
rs7688014	3.00E-13	SLC9B2	creatinine (maximum, inv-norm transformed)	GCST90479561
rs13107325	3.00E-13	SLC39A8	Type 2 diabetes with neurological manifestations (PheCode 250.24)	GCST90475676
rs13107325	3.00E-13	SLC39A8	What is your height? (cm, inv-normal transformed)	GCST90479637
rs13107325	3.00E-13	SLC39A8	Bicarbonate (maximum, inv-norm transformed)	GCST90475145
rs13107325	4.00E-13	SLC39A8	Risk-taking tendency (4-domain principal component model)	GCST007323
rs13107325	4.00E-13	SLC39A8	Schizophrenia	GCST009337
rs223351	4.00E-13	UBE2D3	Hemoglobin A1c levels	GCST90018958
rs13107325	4.00E-13	SLC39A8	DNER protein levels	GCST90469019
rs201814405	4.00E-13	LINC02428,CENPE	Total testosterone levels	GCST90012113
rs13107325	4.00E-13	SLC39A8	wg rh intensity-contrast parahippocampal	GCST90003842
rs35225200	4.00E-13	BANK1,SLC39A8	Cognitive ability, years of educational attainment or schizophrenia (pleiotropy)	GCST008595
rs201081507	4.00E-13	BANK1	Central nucleus volume	GCST90310219
rs9992479	4.00E-13	SLC39A8	Central nucleus volume	GCST90310219
rs201081507	4.00E-13	BANK1	Body mass index (MTAG)	GCST90179150
rs13107325	4.00E-13	SLC39A8	ITGA11 protein levels	GCST90469633
rs13107325	4.00E-13	SLC39A8	PLXDC2 protein levels	GCST90470264
rs62688361	4.00E-13	BANK1	Volume of left medial nucleus	GCST90474891
rs12511373	4.00E-13	BANK1	Volume of right central nucleus	GCST90474915
rs35518360	4.00E-13	SLC39A8,BANK1	Abnormal movement (PheCode 350)	GCST90475840
rs13107325	4.00E-13	SLC39A8	Intervertebral disc disorders (PheCode 722)	GCST90476237
rs13107325	4.00E-13	SLC39A8	Sleep apnea	GCST90479583
rs7377083	5.00E-13	BANK1	Weight	GCST90018949
rs35225200	5.00E-13	BANK1,SLC39A8	Triglycerides	GCST90018975
rs13107325	5.00E-13	SLC39A8	aseg lh intensity VentralDC	GCST90003775
rs34592089	5.00E-13	BANK1	Ventromedial occipital thickness	GCST90572713
rs13107325	5.00E-13	SLC39A8	Glycine levels	GCST90503747
rs13107325	5.00E-13	SLC39A8	Cannabis use disorder (MTAG)	GCST90320053
rs13107325	5.00E-13	SLC39A8	Volume of right cortico-amygdaloid transition area	GCST90474913
rs13107325	5.00E-13	SLC39A8	Phospholipids in medium HDL (UKB data field 23567)	GCST90269664
rs13107325	5.00E-13	SLC39A8	Pain in limb (PheCode 773)	GCST90476270
rs7688014	5.00E-13	SLC9B2	estimated glomerular filtration rate (eGFR, minimum, inv-norm transformed)	GCST90479600
rs13107325	5.00E-13	SLC39A8	Monounsaturated fatty acids to total fatty acids percentage	GCST90502043
rs35518360	6.00E-13	SLC39A8,BANK1	Concentration of very large HDL particles	GCST90302133
rs13107325	6.00E-13	SLC39A8	Obstructive sleep apnea	GCST90274865
rs2851245	6.00E-13	BANK1,SLC39A8	Volume of left central nucleus	GCST90474882
rs13118152	6.00E-13	SLC39A8	Volume of left medial nucleus	GCST90474891
rs114614648	6.00E-13	BANK1	Volume of right central nucleus	GCST90474915
rs13135092	7.00E-13	SLC39A8	Predicted visceral adipose tissue	GCST008744
rs6822371	7.00E-13	SLC39A8	Metabolic syndrome	GCST90444487
rs10715507	7.00E-13	SLC9B2	Estimated glomerular filtration rate	GCST008747
rs13107325	7.00E-13	SLC39A8	Crohn's disease	GCST90446792
rs13107325	7.00E-13	SLC39A8	wg rh intensity-contrast inferiorparietal	GCST90003834
rs13107325	7.00E-13	SLC39A8	IDP dMRI TBSS L1 Cerebral peduncle L	GCST90004090
rs13135092	7.00E-13	SLC39A8	Regional cortical thickness (lateralorbitofrontal)	GCST90399886
rs13135092	7.00E-13	SLC39A8	PRTN3 protein levels	GCST90470348
rs7377083	7.00E-13	BANK1	Whole body fat mass (UKB data field 23100)	GCST90428121
rs13135092	8.00E-13	SLC39A8	F-cooking vegetables liking (derived food-liking factor)	GCST90094743
rs201081507	8.00E-13	BANK1	Fluid intelligence score (baseline)	GCST90565842
rs13107325	8.00E-13	SLC39A8	Waist-to-hip ratio adjusted for BMI	GCST90020025
rs13107325	8.00E-13	SLC39A8	Protein kinase C-binding protein NELL1 levels	GCST90248637
rs10017306	8.00E-13	SLC39A8	Volume of right cortical nucleus	GCST90474918
rs3733197	8.00E-13	BANK1	Body mass index	GCST90255621
rs13107325	8.00E-13	SLC39A8	Body fat percentage (adjusted for testosterone and SHBG)	GCST90432179

rs13107325	8.00E-13	SLC39A8	height (minimum, inv-normal transformed)	GCST90479636
rs13107325	8.00E-13	SLC39A8	triglyceride (minimum, inv-norm transformed)	GCST90480719
rs6855246	9.00E-13	SLC39A8,BANK1	Intelligence (MTAG)	GCST005316
rs13135092	9.00E-13	SLC39A8	Schizophrenia (MTAG)	GCST012089
rs6822371	9.00E-13	SLC39A8	Central nucleus volume	GCST90310219
rs93059	9.00E-13	NFKB1	DNA methylation Hannum age acceleration	GCST90014301
rs10026360	9.00E-13	LINC02428	Height (baseline)	GCST90565843
rs13107325	9.00E-13	SLC39A8	Anterior thalamic nuclei volume	GCST012005
rs13107325	9.00E-13	SLC39A8	ITGB2 protein levels	GCST90469644
rs6822371	9.00E-13	SLC39A8	Volume of right medial nucleus	GCST90474925
rs13107325	1.00E-12	SLC39A8	Youthful appearance (self-reported)	GCST011011
rs34592089	1.00E-12	BANK1	Intelligence (MTAG)	GCST005316
rs60371688	1.00E-12	NFKB1	Albumin-globulin ratio	GCST005987
rs35225200	1.00E-12	BANK1,SLC39A8	Mean arterial pressure x alcohol consumption interaction (2df test)	GCST006167
rs13107325	1.00E-12	SLC39A8	Body mass index	GCST002783
rs13107325	1.00E-12	SLC39A8	IDP dMRI TBSS L1 Cerebral peduncle R	GCST90004089
rs4426778	1.00E-12	BANK1	Systemic lupus erythematosus	GCST007400
rs68103113	1.00E-12	SLC39A8	Anterior amygdaloid area volume	GCST90310230
rs4371620	1.00E-12	BANK1	Central nucleus volume	GCST90310229
rs12511373	1.00E-12	BANK1	High density lipoprotein cholesterol levels	GCST90239652
rs13107325	1.00E-12	SLC39A8	BMI and adiposity (confirmatory factor analysis Factor 7)	GCST90309342
rs13107325	1.00E-12	SLC39A8	Lateral thalamic nuclei volume	GCST012008
rs28625045	1.00E-12	BANK1	HPSE protein levels	GCST90469473
rs13107325	1.00E-12	SLC39A8	LIFR protein levels	GCST90469769
rs4572884	1.00E-12	BANK1	MMP1 protein levels	GCST90469919
rs13107325	1.00E-12	SLC39A8	SPINT1 protein levels	GCST90470727
rs35518360	1.00E-12	SLC39A8,BANK1	Income (MTAG)	GCST90565742
rs13107325	1.00E-12	SLC39A8	Total concentration of lipoprotein particles (UKB data field 23427)	GCST90269524
rs768027456	1.00E-12	BANK1	Volume of right cortical nucleus	GCST90474918
rs10017306	1.00E-12	SLC39A8	Volume of right cortical nucleus	GCST90474919
rs13140486	1.00E-12	SLC39A8,NFKB1	Occipital area (unadjusted for global measures)	GCST90271795
rs223391	1.00E-12	UBE2D3	Body mass index	GCST90255621
rs13107325	1.00E-12	SLC39A8	Heparan-sulfate 6-O-sulfotransferase 1 levels	GCST90247934
rs13107325	2.00E-12	SLC39A8	Body mass index	GCST002783
rs6855246	2.00E-12	SLC39A8,BANK1	Brain region volumes	GCST009518
rs13107325	2.00E-12	SLC39A8	Leisure sedentary behaviour (television watching)	GCST010084
rs3960788	2.00E-12	SLC9B1	Atrial fibrillation	GCST006061
rs7688014	2.00E-12	SLC9B2	Estimated glomerular filtration rate	GCST008747
rs13126505	2.00E-12	BANK1	Crohn's disease	GCST001729
rs13107325	2.00E-12	SLC39A8	Schizophrenia	GCST003048
rs35225200	2.00E-12	BANK1,SLC39A8	Autism spectrum disorder or schizophrenia	GCST004521
rs13107325	2.00E-12	SLC39A8	wg rh intensity-contrast superiorparietal	GCST90003855
rs13107325	2.00E-12	SLC39A8	wg rh intensity-contrast supramarginal	GCST90003857
rs13105682	2.00E-12	BANK1	IDP SWI T2star left caudate	GCST90003865
rs13107325	2.00E-12	SLC39A8	wg lh intensity-contrast paracentral	GCST90003808
rs13105581	2.00E-12	SLC39A8	Red blood cell count	GCST90002403
rs151401	2.00E-12	SLC39A8	Aspartate aminotransferase levels	GCST90011899
rs13107325	2.00E-12	SLC39A8	Urate levels (UKB data field 30880)	GCST90468107
rs7377083	2.00E-12	BANK1	Waist circumference (UKB data field 48)	GCST90468182
rs13135092	2.00E-12	SLC39A8	F-vegetarian liking (derived food-liking factor)	GCST90094863
rs35978636	2.00E-12	BANK1	CCL13 protein levels	GCST90468565
rs34166099	2.00E-12	BANK1	Systolic blood pressure (MTAG)	GCST90449056
rs34205595	2.00E-12	BANK1	Height (baseline)	GCST90565843
rs10017313	2.00E-12	SLC9B1	Triglyceride levels	GCST90239661
rs13107325	2.00E-12	SLC39A8	TPK1 protein levels	GCST90470943
rs75877836	2.00E-12	LINC02428	Pallidum iron levels (quantitative susceptibility mapping)	GCST90551867
rs6846971	2.00E-12	NFKB1,SLC39A8	Volume of right central nucleus	GCST90475023
rs13107325	2.00E-12	SLC39A8	Volume of left cortical nucleus	GCST90474967
rs13135092	2.00E-12	SLC39A8	Opioid use disorder (MTAG)	GCST90296425
rs35225200	2.00E-12	BANK1,SLC39A8	Cholesteryl esters to total lipids in IDL percentage (UKB data field 23611)	GCST90269708
rs13114738	3.00E-12	SLC39A8	High density lipoprotein cholesterol levels	GCST007140
rs230540	3.00E-12	NFKB1	Membranous nephropathy	GCST010004
rs13107325	3.00E-12	SLC39A8	IDP T1 FAST ROIs L caudate	GCST90002549
rs13107325	3.00E-12	SLC39A8	Urine argininate* levels in chronic kidney disease	GCST90264827
rs13107325	3.00E-12	SLC39A8	Free cholesterol to total lipids ratio in large LDL	GCST90092861
rs13107325	3.00E-12	SLC39A8	Triglycerides to total lipids ratio in large LDL	GCST90092867
rs13107325	3.00E-12	SLC39A8	Cholesterol to total lipids ratio in large LDL	GCST90092857
rs223490	3.00E-12	MANBA	AFP levels	GCST90239635
rs2631249	3.00E-12	BANK1	Educational attainment	GCST90105038
rs1813006	3.00E-12	BANK1,SLC39A8	Osteoarthritis	GCST90134288
rs1813006	3.00E-12	BANK1,SLC39A8	Osteoarthritis	GCST90134286
rs13107325	3.00E-12	SLC39A8	Knee osteoarthritis	GCST90566800
rs12510679	3.00E-12	SLC39A8	Volume of left medial nucleus	GCST90474891
rs181121136	3.00E-12	BANK1,SLC39A8	Volume of right medial nucleus	GCST90475042
rs34592089	3.00E-12	BANK1	Ventromedial occipital thickness (unadjusted for global measures)	GCST90271813
rs1813006	3.00E-12	BANK1,SLC39A8	Addiction risk factors	GCST90274724

rs13107325	3.00E-12	SLC39A8	hemisphere salience/ventral attention network to amygdala white-matter structural connect	GCST90302724
rs230495	3.00E-12	NFKB1	Interleukin-22 receptor subunit alpha-2 levels	GCST90248060
rs13107325	3.00E-12	SLC39A8	Hyperglyceridemia (PheCode 272.12)	GCST90479925
rs6839635	3.00E-12	SLC9B1	Glucose (fasting status unknown, maximum, inv-norm transformed)	GCST90479625
rs72668656	3.00E-12	LINC02428	Creatinine levels	GCST90019502
rs62338910	4.00E-12	LINC02428	HDL cholesterol levels	GCST010242
rs11097787	4.00E-12	SLC39A8,NFKB1	Basophil count	GCST90002292
rs7665090	4.00E-12	MANBA,NFKB1	Primary biliary cholangitis	GCST001010
rs3774959	4.00E-12	NFKB1	Ulcerative colitis	GCST001728
rs228611	4.00E-12	MANBA	Glomerular filtration rate (creatinine)	GCST003372
rs4365725	4.00E-12	BANK1	CXCL6 protein levels	GCST90468933
rs13135092	4.00E-12	SLC39A8	DEFA1 or DEFA1B protein levels	GCST90468979
rs13107325	4.00E-12	SLC39A8	Brain shape (segment 20)	GCST90012899
rs4371620	4.00E-12	BANK1	High-density lipoprotein levels (MTAG)	GCST90179147
rs230534	4.00E-12	NFKB1	Systemic sclerosis	GCST90319682
rs13107325	4.00E-12	SLC39A8	Short sleep duration (<5 hours)	GCST90428611
rs35518360	4.00E-12	SLC39A8,BANK1	Abnormality of gait (PheCode 350.2)	GCST90475841
rs13107325	4.00E-12	SLC39A8	Thoracic or lumbosacral neuritis or radiculitis, unspecified (PheCode 763)	GCST90480572
rs72668656	4.00E-12	LINC02428	Cystatin C levels	GCST90019504
rs13107325	5.00E-12	SLC39A8	Hypertension	GCST009685
rs13135092	5.00E-12	SLC39A8	ThalamNuclei lh volume CM	GCST90002718
rs13101632	5.00E-12	BANK1,SLC39A8	Medial nucleus volume	GCST90310225
rs11724128	5.00E-12	SLC39A8	Diastolic blood pressure (MTAG)	GCST90449057
rs13107325	5.00E-12	SLC39A8	Triglyceride to phosphoglyceride ratio	GCST90454483
rs13109404	5.00E-12	BANK1	Sleep duration	GCST90272876
rs9992479	5.00E-12	SLC39A8	Body mass index	GCST90255621
rs13107325	5.00E-12	SLC39A8	Pain in limb (PheCode 773)	GCST90480581
rs2720460	6.00E-12	CENPE	Testicular germ cell tumor	GCST002855
rs233821	6.00E-12	SLC39A8	Anterior amygdaloid area volume	GCST90310230
rs2903282	6.00E-12	SLC39A8,NFKB1	C1QL2 protein levels	GCST90468487
rs11722146	6.00E-12	NFKB1	ICAM5 protein levels	GCST90469502
rs75877836	6.00E-12	LINC02428	Substantia nigra iron levels (quantitative susceptibility mapping)	GCST90551871
rs6855246	6.00E-12	SLC39A8,BANK1	SFRP1 protein levels	GCST90470610
rs17249745	6.00E-12	BANK1	Educational attainment	GCST90105038
rs11941622	6.00E-12	BANK1,SLC39A8	Volume of right central nucleus	GCST90474915
rs13135092	6.00E-12	SLC39A8	Osteoarthritis of the hip or knee (with total joint replacement)	GCST90566804
rs1813006	6.00E-12	BANK1,SLC39A8	Childhood body mass index	GCST90301649
rs13107325	6.00E-12	SLC39A8	Superficial cellulitis and abscess (PheCode 681)	GCST90480437
rs17199964	7.00E-12	BANK1	Intelligence	GCST006250
rs13135688	7.00E-12	SLC39A8	Cortical nucleus volume	GCST90310228
rs79952106	7.00E-12	BANK1	ANGPT1 protein levels	GCST90468300
rs34592089	7.00E-12	BANK1	Idiopathic knee osteoarthritis	GCST90297779
rs17032400	7.00E-12	SLC39A8	High-density lipoprotein levels (MTAG)	GCST90179147
rs9994654	7.00E-12	SLC9B1	High-density lipoprotein levels (MTAG)	GCST90179147
rs13107325	7.00E-12	SLC39A8	ERP44 protein levels	GCST90469150
rs13107325	7.00E-12	SLC39A8	LAMB1 protein levels	GCST90469732
rs13107325	8.00E-12	SLC39A8	Diastolic blood pressure	GCST007268
rs2851334	8.00E-12	BANK1	Volume of right central nucleus	GCST90474916
rs113473633	9.00E-12	NFKB1	Eosinophil counts	GCST004606
rs13107325	9.00E-12	SLC39A8	IDP dMRI TBSS ICVF Anterior limb of internal capsule R	GCST90004343
rs13135092	9.00E-12	SLC39A8	Cortical thickness (min-P)	GCST010696
rs12498921	9.00E-12	BANK1	TMEM106A protein levels	GCST90470886
rs28625045	9.00E-12	BANK1	LAMA4 protein levels	GCST90469731
rs150228862	9.00E-12	BANK1	Educational attainment	GCST90105038
rs17199964	9.00E-12	BANK1	Intelligence	GCST90264174
rs35518360	9.00E-12	SLC39A8,BANK1	Cholesteryl esters in very large HDL (UKB data field 23555)	GCST90269652
rs13107325	9.00E-12	SLC39A8	Short sleep duration (<5 hours)	GCST90428613
rs13107325	9.00E-12	SLC39A8	Asthma	GCST90476024
rs13107325	9.00E-12	SLC39A8	Enthesopathy (PheCode 726.1)	GCST90476239
rs13107325	9.00E-12	SLC39A8	Aspartate aminotransferase (AST, mean, inv-norm transformed)	GCST90479510
rs113473633	1.00E-11	NFKB1	Hypothyroidism	GCST007073
rs13109404	1.00E-11	BANK1	Sleep duration	GCST007982
rs13107325	1.00E-11	SLC39A8	Mean arterial pressure	GCST006231
rs13107325	1.00E-11	SLC39A8	aseg rh intensity Caudate	GCST90003783
rs35663811	1.00E-11	BANK1	Central nucleus volume	GCST90310229
rs34166099	1.00E-11	BANK1	Central nucleus volume	GCST90310219
rs74332078	1.00E-11	UBE2D3	High density lipoprotein cholesterol levels	GCST90239652
rs13107325	1.00E-11	SLC39A8	Gout	GCST90455677
rs35518360	1.00E-11	SLC39A8,BANK1	Diastolic blood pressure x low social support interaction (2df test)	GCST90093268
rs13107325	1.00E-11	SLC39A8	L1CAM protein levels	GCST90469723
rs13107325	1.00E-11	SLC39A8	2-oxoarginine levels	GCST90102787
rs13107325	1.00E-11	SLC39A8	Phospholipids to total lipids in medium HDL percentage (UKB data field 23639)	GCST90269736
rs35518360	1.00E-11	SLC39A8,BANK1	Triglycerides to total lipids in small HDL percentage (UKB data field 23648)	GCST90269745
rs35225200	1.00E-11	BANK1,SLC39A8	Diseases of nail, NOS (PheCode 703)	GCST90476207
rs34333163	2.00E-11	SLC39A8	Monocyte percentage of white cells	GCST90002394
rs9992479	2.00E-11	SLC39A8	Metabolic syndrome	GCST90444487

rs10026360	2.00E-11	LINC02428	Height	GCST90435412
rs13107325	2.00E-11	SLC39A8	Mean diameter of HDL particles	GCST90301972
rs13107325	2.00E-11	SLC39A8	Gamma glutamyl transpeptidase	GCST90018954
rs5860730	2.00E-11	MANBA	Total testosterone levels	GCST90012114
rs13107325	2.00E-11	SLC39A8	aparc-DKTatlas rh thickness transversetemporal	GCST90003601
rs13107325	2.00E-11	SLC39A8	aparc-DKTatlas lh thickness paracentral	GCST90003555
rs13107325	2.00E-11	SLC39A8	aparc-Desikan rh thickness transversetemporal	GCST90003511
rs13107325	2.00E-11	SLC39A8	Voxel-wise structural brain imaging measurements	GCST007357
rs13115343	2.00E-11	NFKB1,SLC39A8	Central nucleus volume	GCST90310219
rs13105682	2.00E-11	BANK1	Accessory basal nucleus volume	GCST90310224
rs13107325	2.00E-11	SLC39A8	Plasma argininate* levels in chronic kidney disease	GCST90264826
rs13107325	2.00E-11	SLC39A8	Total cholines levels	GCST90092812
rs34333163	2.00E-11	SLC39A8	Insomnia	GCST90131903
rs13107325	2.00E-11	SLC39A8	A body shape index	GCST90020024
rs13107325	2.00E-11	SLC39A8	ADAM22 protein levels	GCST90468218
rs112623404	2.00E-11	UBE2D3	Smoking initiation	GCST90243985
rs57957741	2.00E-11	BANK1,SLC39A8	Volume of right medial nucleus	GCST90474925
rs62688361	2.00E-11	BANK1	Volume of right medial nucleus	GCST90474924
rs150228862	2.00E-11	BANK1	Volume of right cortical nucleus	GCST90474919
rs13107325	2.00E-11	SLC39A8	Lung function (forced vital capacity)	GCST90244093
rs4648052	2.00E-11	NFKB1	ICD10 J35: Chronic diseases of tonsils and adenoids	GCST90269775
rs13107325	2.00E-11	SLC39A8	Diseases of esophagus (PheCode 530)	GCST90476045
rs13107325	2.00E-11	SLC39A8	Chronic ulcer of skin (PheCode 707)	GCST90476216
rs72668656	2.00E-11	LINC02428	Estimated glomerular filtration rate	GCST90019506
rs13135092	3.00E-11	SLC39A8	Adventurousness	GCST007324
rs230525	3.00E-11	NFKB1	Red blood cell count	GCST007069
rs17199964	3.00E-11	BANK1	Brain region volumes	GCST009518
rs13114738	3.00E-11	SLC39A8	High density lipoprotein cholesterol levels	GCST007140
rs12511373	3.00E-11	BANK1	Body mass index	GCST009871
rs13105581	3.00E-11	SLC39A8	General cognitive ability	GCST006269
rs13107325	3.00E-11	SLC39A8	Systolic blood pressure	GCST90018972
rs188539658	3.00E-11	MANBA,UBE2D3	Bioavailable testosterone levels	GCST90012104
rs13107325	3.00E-11	SLC39A8	aparc-Desikan rh thickness GlobalMeanThickness	GCST90003479
rs13107325	3.00E-11	SLC39A8	Triglycerides to total lipids ratio in medium HDL	GCST90092903
rs13107325	3.00E-11	SLC39A8	Glycine levels	GCST90092820
rs35225200	3.00E-11	BANK1,SLC39A8	CAMSIS occupational score (MTAG)	GCST90492678
rs13107325	3.00E-11	SLC39A8	Volume of left whole amygdala	GCST90474898
rs57957741	3.00E-11	BANK1,SLC39A8	Volume of right central nucleus	GCST90474916
rs12501273	3.00E-11	BANK1	Volume of right central nucleus	GCST90474916
rs238449	3.00E-11	BANK1,SLC39A8	Volume of right cortical nucleus	GCST90474918
rs13107325	3.00E-11	SLC39A8	Systolic blood pressure	GCST90472782
rs142074392	3.00E-11	BANK1	Bone mineral density mean	GCST90321120
rs13107325	3.00E-11	SLC39A8	Sleep disorders (PheCode 327)	GCST90475823
rs13107325	3.00E-11	SLC39A8	Hyperglyceridemia (PheCode 272.12)	GCST90475724
rs35225200	3.00E-11	BANK1,SLC39A8	Monounsaturated fatty acids to total fatty acids percentage	GCST90502372
rs559378663	3.00E-11	SLC39A8	specific abnormal findings on radiological and other examination of biliary tract (PheCode 57	GCST90480356
rs13107325	3.00E-11	SLC39A8	Diaphragmatic hernia (PheCode 550.2)	GCST90480312
rs223320	3.00E-11	CISD2	Sex hormone-binding globulin levels	GCST90019518
rs13107325	4.00E-11	SLC39A8	Diastolic blood pressure	GCST007098
rs227275	4.00E-11	MANBA	Allergic disease (asthma, hay fever or eczema)	GCST005038
rs13107325	4.00E-11	SLC39A8	Eosinophil percentage of white cells	GCST90002382
rs3960788	4.00E-11	SLC9B1	Atrial fibrillation	GCST006061
rs13107325	4.00E-11	SLC39A8	Mean arterial pressure x alcohol consumption interaction (2df test)	GCST006167
rs13109404	4.00E-11	BANK1	Pappalysin-1 levels	GCST90012036
rs13107325	4.00E-11	SLC39A8	Phosphatidylcholine levels	GCST90092937
rs13131500	4.00E-11	NFKB1,SLC39A8	Whole amygdala volume	GCST90310231
rs223317	4.00E-11	CISD2	Blood urea nitrogen levels	GCST90103632
rs4648051	4.00E-11	NFKB1	Pharyngeal disease	GCST90269785
rs35518360	4.00E-11	SLC39A8,BANK1	Phospholipids in very large HDL (UKB data field 23553)	GCST90269650
rs13107325	4.00E-11	SLC39A8	Phospholipids to total lipids in very small VLDL percentage (UKB data field 23604)	GCST90269701
rs181121136	4.00E-11	BANK1,SLC39A8	Volume of left central nucleus	GCST90474883
rs62329461	4.00E-11	SLC39A8	Volume of right central nucleus	GCST90474915
rs768027456	4.00E-11	BANK1	Volume of right medial nucleus	GCST90474924
rs75088572	4.00E-11	SLC39A8,BANK1	Volume of right medial nucleus	GCST90474924
rs13107325	4.00E-11	SLC39A8	Allergic rhinitis (PheCode 476)	GCST90476021
rs13107325	4.00E-11	SLC39A8	Esophagitis, GERD and related diseases (PheCode 530.1)	GCST90476046
rs35225200	4.00E-11	BANK1,SLC39A8	Abnormal movement (PheCode 350)	GCST90480029
rs223409	4.00E-11	UBE2D3	Hemoglobin A1c (HbA1c, mean, inv-norm transformed)	GCST90479501
rs223409	4.00E-11	UBE2D3	Hemoglobin A1c (HbA1c, maximum, inv-norm transformed)	GCST90479500
rs13107325	5.00E-11	SLC39A8	Total cholesterol levels	GCST007143
rs13135092	5.00E-11	SLC39A8	Total cholesterol levels	GCST007143
rs13107325	5.00E-11	SLC39A8	Systolic blood pressure	GCST006259
rs13107325	5.00E-11	SLC39A8	wg lh intensity-contrast precentral	GCST90003815
rs13107325	5.00E-11	SLC39A8	wg lh intensity-contrast caudalmiddlefrontal	GCST90003795
rs13135092	5.00E-11	SLC39A8	Posterolateral temporal area	GCST90572700
rs4699006	5.00E-11	SLC39A8	Fluid intelligence score (baseline)	GCST90565842

rs13127398	5.00E-11	BANK1	Insomnia	GCST90131903
rs35518360	5.00E-11	SLC39A8,BANK1	Total lipids in very large HDL (UKB data field 23552)	GCST90269649
rs13136118	5.00E-11	BANK1	Volume of left central nucleus	GCST90474882
rs12511373	5.00E-11	BANK1	Volume of right cortical nucleus	GCST90474918
rs233828	5.00E-11	SLC39A8	Intelligence	GCST90264174
rs35225200	5.00E-11	BANK1,SLC39A8	Cholesterol to total lipids in IDL percentage (UKB data field 23610)	GCST90269707
rs13107325	5.00E-11	SLC39A8	Diseases of esophagus (PheCode 530)	GCST90480294
rs7692921	6.00E-11	SLC39A8	Red cell distribution width	GCST007074
rs374187209	6.00E-11	SLC39A8,NFKB1	Basophil count	GCST90002379
rs13107325	6.00E-11	SLC39A8	Hypertension	GCST90446531
rs4699030	6.00E-11	NFKB1	Mouth ulcers	GCST007839
rs114601774	6.00E-11	LINC02428	Central nucleus volume	GCST90310229
rs13135092	6.00E-11	SLC39A8	Schizophrenia (MTAG)	GCST90020095
rs13107325	6.00E-11	SLC39A8	FVC or gastro-oesophageal reflux disease (pleiotropy)	GCST90454176
rs7695593	6.00E-11	LINC02428	Diastolic blood pressure	GCST90132904
rs151411	6.00E-11	SLC39A8,BANK1	Insomnia	GCST90131901
rs17199964	6.00E-11	BANK1	ttention deficit hyperactivity disorder or autism spectrum disorder or intelligence (pleiotropy	GCST90134330
rs223482	6.00E-11	MANBA,UBE2D3	Migraine or fasting glucose levels	GCST90281113
rs13135092	7.00E-11	SLC39A8	HDL cholesterol levels in current drinkers	GCST008085
rs113473633	7.00E-11	NFKB1	White blood cell count	GCST007070
rs13107325	7.00E-11	SLC39A8	HDL cholesterol	GCST000755
rs13107325	7.00E-11	SLC39A8	Phospholipids in very large HDL	GCST90302134
rs13107325	7.00E-11	SLC39A8	IDP dMRI TBSS MD Cerebral peduncle L	GCST90004042
rs13107325	7.00E-11	SLC39A8	aparc-Desikan lh thickness paracentral	GCST90003461
rs13107325	7.00E-11	SLC39A8	BA-exvivo lh thickness BA4p	GCST90003518
rs13107325	7.00E-11	SLC39A8	Low hand grip strength (60 years and older) (EWGSOP)	GCST90007526
rs13135688	7.00E-11	SLC39A8	Lateral nucleus volume	GCST90310227
rs151411	7.00E-11	SLC39A8,BANK1	Insomnia	GCST90131903
rs6855246	7.00E-11	SLC39A8,BANK1	ttention deficit hyperactivity disorder or autism spectrum disorder or intelligence (pleiotropy	GCST90134330
rs35225200	7.00E-11	BANK1,SLC39A8	ISEI occupational score (MTAG)	GCST90492677
rs2126627	7.00E-11	NFKB1,SLC39A8	Volume of right central nucleus	GCST90475024
rs13118152	7.00E-11	SLC39A8	Volume of left central nucleus	GCST90474957
rs35225200	7.00E-11	BANK1,SLC39A8	Cholesterol in very large HDL (UKB data field 23554)	GCST90269651
rs13107325	8.00E-11	SLC39A8	Diastolic blood pressure	GCST007098
rs11726195	8.00E-11	NFKB1,MANBA	Lymphocyte percentage of white cells	GCST90002389
rs13107325	8.00E-11	SLC39A8	Schizophrenia (MTAG)	GCST010640
rs11097755	8.00E-11	BANK1	Diastolic blood pressure (MTAG)	GCST90449057
rs7695096	8.00E-11	SLC9B1	Hypertension (confirmatory factor analysis Factor 12)	GCST90309346
rs13107325	8.00E-11	SLC39A8	Diastolic blood pressure x depressive symptoms interaction (2df test)	GCST90093171
rs35225200	8.00E-11	BANK1,SLC39A8	SIOPS occupational score (MTAG)	GCST90492679
rs12510679	8.00E-11	SLC39A8	Volume of right cortical nucleus	GCST90474918
rs35225200	8.00E-11	BANK1,SLC39A8	Triglycerides to total lipids in IDL percentage (UKB data field 23613)	GCST90269710
rs6855246	9.00E-11	SLC39A8,BANK1	Brain region volumes	GCST009518
rs113473633	9.00E-11	NFKB1	Sum eosinophil basophil counts	GCST004624
rs13109404	9.00E-11	BANK1	Sleep duration	GCST007561
rs13107325	9.00E-11	SLC39A8	Regional cortical thickness (superiorparietal)	GCST90399905
rs13107325	9.00E-11	SLC39A8	Regional cortical thickness (precuneus)	GCST90399901
rs13107325	9.00E-11	SLC39A8	Hemoglobin concentration	GCST90258653
rs11941622	9.00E-11	BANK1,SLC39A8	Volume of left medial nucleus	GCST90474892
rs13109404	9.00E-11	BANK1	Systolic blood pressure	GCST90310304
rs4648050	9.00E-11	NFKB1	B-cell lymphoblastic leukemia or primary biliary cholangitis (pleiotropy)	GCST90428827
rs113473633	1.00E-10	NFKB1	Eosinophil percentage of granulocytes	GCST004617
rs13114738	1.00E-10	SLC39A8	Age-related disease endophenotypes	GCST004046
rs71597109	1.00E-10	BANK1	Chronic lymphocytic leukemia	GCST004146
rs230501	1.00E-10	NFKB1	Lymphocyte percentage of white cells	GCST90002389
rs13107325	1.00E-10	SLC39A8	Low density lipoprotein cholesterol levels	GCST010204
rs13107325	1.00E-10	SLC39A8	Blood pressure	GCST001236
rs13126505	1.00E-10	BANK1	Inflammatory bowel disease	GCST003043
rs13107325	1.00E-10	SLC39A8	Mean corpuscular volume	GCST90002392
rs35225200	1.00E-10	BANK1,SLC39A8	er, autism spectrum disorder, bipolar disorder, major depression, obsessive-compulsive disor	GCST009600
rs179195	1.00E-10	MANBA	Serum alkaline phosphatase levels	GCST90011900
rs113473633	1.00E-10	NFKB1	White blood cell count	GCST90002407
rs13109404	1.00E-10	BANK1	Posterolateral temporal area	GCST90572700
rs34166099	1.00E-10	BANK1	Physical function (baseline)	GCST90565837
rs13107325	1.00E-10	SLC39A8	Triglyceride levels (MTAG)	GCST90179149
rs13101632	1.00E-10	BANK1,SLC39A8	ttention deficit hyperactivity disorder or autism spectrum disorder or intelligence (pleiotropy	GCST90134330
rs35518360	1.00E-10	SLC39A8,BANK1	Triglycerides to phosphoglycerides ratio (UKB data field 23435)	GCST90269532
rs3974479	1.00E-10	SLC9B1	Creatinine levels (UKB data field 23478)	GCST90269575
rs35225200	1.00E-10	BANK1,SLC39A8	Non-response to survey questionnaires: "I don't know" item	GCST90266935
rs35518360	1.00E-10	SLC39A8,BANK1	Cholesteryl esters to total lipids in very small VLDL percentage (UKB data field 23606)	GCST90269703
rs13107325	1.00E-10	SLC39A8	Cholesterol to total lipids in medium HDL percentage (UKB data field 23640)	GCST90269737
rs58611096	1.00E-10	LINC02428	Male puberty timing (age at voice breaking MTAG)	GCST90012088
rs2851245	1.00E-10	BANK1,SLC39A8	Volume of right medial nucleus	GCST90475041
rs10017306	1.00E-10	SLC39A8	Volume of right medial nucleus	GCST90475042
rs13118152	1.00E-10	SLC39A8	Volume of right accessory basal nucleus	GCST90474906
rs376206821	1.00E-10	BANK1	Volume of right medial nucleus	GCST90474924

rs13107325	1.00E-10	SLC39A8	Occipital thickness (unadjusted for global measures)	GCST90271808
rs13107325	1.00E-10	SLC39A8	Tinnitus	GCST90428112
rs35225200	1.00E-10	BANK1,SLC39A8	Concentration of very large HDL particles (UKB data field 23551)	GCST90269648
rs13126505	1.00E-10	BANK1	Whole body fat mass (UKB data field 23100)	GCST90428121
rs34592089	1.00E-10	BANK1	Urate levels	GCST90019524
rs289139,rs151404	1.00E-10	TLN2 x SLC39A8	Total PHF-tau (SNP x SNP interaction)	GCST010340
rs13107325	2.00E-10	SLC39A8	Body mass index (joint analysis main effects and physical activity interaction)	GCST004558
rs13107325	2.00E-10	SLC39A8	Eczema	GCST007075
rs13135092	2.00E-10	SLC39A8	HDL cholesterol levels in current drinkers	GCST008085
rs1580278	2.00E-10	LINC02428,CENPE	Type 2 diabetes	GCST009379
rs11097787	2.00E-10	SLC39A8,NFKB1	Basophil count	GCST90002296
rs13107325	2.00E-10	SLC39A8	Body mass index	GCST004557
rs13107325	2.00E-10	SLC39A8	Balding type 1	GCST007038
rs13105581	2.00E-10	SLC39A8	Intelligence (MTAG)	GCST005316
rs2720460	2.00E-10	CENPE	Testicular germ cell tumor	GCST002022
rs1598856	2.00E-10	NFKB1	Primary biliary cholangitis	GCST004302
rs13107325	2.00E-10	SLC39A8	Body mass index (joint analysis main effects and smoking interaction)	GCST004497
rs13105682	2.00E-10	BANK1	ThalamNuclei lh volume VAmc	GCST90002723
rs2033900	2.00E-10	BANK1,SLC39A8	Anterior amygdaloid area volume	GCST90310230
rs17032400	2.00E-10	SLC39A8	Diastolic blood pressure (MTAG)	GCST90449057
rs13107325	2.00E-10	SLC39A8	Cholesterol to total lipids ratio in large HDL	GCST90092845
rs35518360	2.00E-10	SLC39A8,BANK1	Triglycerides to total lipids ratio in large HDL	GCST90092855
rs13107325	2.00E-10	SLC39A8	Triglyceride levels in non-type 2 diabetes	GCST90244680
rs13107325	2.00E-10	SLC39A8	Sphingomyelins levels (UKB data field 23438)	GCST90269535
rs150228862	2.00E-10	BANK1	Volume of left central nucleus	GCST90474883
rs114601774	2.00E-10	LINC02428	Volume of left central nucleus	GCST90474883
rs11941622	2.00E-10	BANK1,SLC39A8	Volume of left central nucleus	GCST90474883
rs35476121	2.00E-10	BANK1	Volume of right central nucleus	GCST90475024
rs62688361	2.00E-10	BANK1	Volume of right central nucleus	GCST90474915
rs62329462	2.00E-10	SLC39A8	Volume of right central nucleus	GCST90474916
rs13140033	2.00E-10	NFKB1,SLC39A8	Systolic blood pressure	GCST90472780
rs112519623	2.00E-10	SLC39A8	Body mass index or hip osteoarthritis (pleiotropy)	GCST90271771
rs112519623	2.00E-10	SLC39A8	Body mass index	GCST90255621
rs13126505	2.00E-10	BANK1	Height	GCST90245844
rs34333163	2.00E-10	SLC39A8	Low density lipoprotein cholesterol levels	GCST90019512
rs13107325	3.00E-10	SLC39A8	Body mass index	GCST004557
rs13107325	3.00E-10	SLC39A8	Spherical equivalent	GCST010378
rs7676765	3.00E-10	SLC9B1	Schizophrenia	GCST009337
rs13107612	3.00E-10	BANK1	seases (ankylosing spondylitis, Crohn's disease, psoriasis, primary sclerosing cholangitis, ulcera	GCST005537
rs230542	3.00E-10	NFKB1	Mean corpuscular hemoglobin concentration	GCST90002328
rs13107325	3.00E-10	SLC39A8	BMI (adjusted for smoking behaviour)	GCST004495
rs13107325	3.00E-10	SLC39A8	Alcohol use disorder (consumption score)	GCST006718
rs13135092	3.00E-10	SLC39A8	Cognitive traits (MTAG)	GCST90011293
rs34208976	3.00E-10	BANK1	Central nucleus volume	GCST90310229
rs13107325	3.00E-10	SLC39A8	Triglycerides to total lipids ratio in very small VLDL	GCST90093039
rs201081507	3.00E-10	BANK1	Height (baseline)	GCST90565843
rs223449	3.00E-10	UBE2D3,MANBA	Atrial fibrillation	GCST90204201
rs13136118	3.00E-10	BANK1	Volume of left medial nucleus	GCST90474891
rs181121136	3.00E-10	BANK1,SLC39A8	Volume of right central nucleus	GCST90475024
rs577329140	3.00E-10	NFKB1,SLC39A8	Volume of right central nucleus	GCST90475026
rs6846971	3.00E-10	NFKB1,SLC39A8	Volume of right cortical nucleus	GCST90475029
rs1811810	3.00E-10	NFKB1,SLC39A8	Mean volume of bilateral amygdala	GCST90474900
rs11941622	3.00E-10	BANK1,SLC39A8	Volume of right medial nucleus	GCST90475045
rs6822371	3.00E-10	SLC39A8	Volume of right medial nucleus	GCST90475042
rs4699258	3.00E-10	BANK1	Volume of left central nucleus	GCST90474957
rs62688361	3.00E-10	BANK1	Volume of right central nucleus	GCST90474916
rs13107325	4.00E-10	SLC39A8	Metabolic syndrome	GCST009602
rs13107325	4.00E-10	SLC39A8	Body fat percentage	GCST007064
rs10516487	4.00E-10	BANK1	Systemic lupus erythematosus	GCST000143
rs13107325	4.00E-10	SLC39A8	Medication use (agents acting on the renin-angiotensin system)	GCST90018988
rs56095122	4.00E-10	UBE2D3	White blood cell count	GCST90018978
rs13107325	4.00E-10	SLC39A8	wg lh intensity-contrast inferiorparietal	GCST90003799
rs35518360	4.00E-10	SLC39A8,BANK1	ST2 protein levels	GCST90012040
rs13107325	4.00E-10	SLC39A8	High density lipoprotein cholesterol levels	GCST011348
rs13131500	4.00E-10	NFKB1,SLC39A8	Whole amygdala volume	GCST90310220
rs151404	4.00E-10	SLC39A8	Medial nucleus volume	GCST90310225
rs223401	4.00E-10	UBE2D3	Estimated glomerular filtration rate	GCST007876
rs13107325	4.00E-10	SLC39A8	Medication use (agents acting on the renin-angiotensin system)	GCST007930
rs13105682	4.00E-10	BANK1	Height (standard GWA)	GCST90267284
rs13105682	4.00E-10	BANK1	BMI (standard GWA)	GCST90267268
rs13135092	4.00E-10	SLC39A8	Opioid use disorder (MTAG)	GCST90134625
rs223361	4.00E-10	UBE2D3	Pulse pressure	GCST90310296
rs13107325	4.00E-10	SLC39A8	Osteoarthritis	GCST90134279
rs223482	4.00E-10	MANBA,UBE2D3	Headache or fasting glucose levels	GCST90281119
rs151381	4.00E-10	BANK1,SLC39A8	Volume of left central nucleus	GCST90474883
rs13115343	4.00E-10	NFKB1,SLC39A8	Volume of right cortical nucleus	GCST90475030

rs11446392	4.00E-10	SLC39A8	Volume of right accessory basal nucleus	GCST90474906
rs35225200	4.00E-10	BANK1,SLC39A8	Polyunsaturated fatty acids to monounsaturated fatty acids ratio	GCST90502147
rs13107325	5.00E-10	SLC39A8	Body fat percentage	GCST007064
rs13107325	5.00E-10	SLC39A8	Triglyceride levels	GCST010173
rs13144764	5.00E-10	LINC02428	Apolipoprotein A1 levels	GCST010241
rs230489	5.00E-10	SLC39A8,NFKB1	General cognitive ability	GCST006269
rs13107325	5.00E-10	SLC39A8	Body mass index	GCST004557
rs62323488	5.00E-10	SLC39A8,BANK1	Central nucleus volume	GCST90310229
rs112519623	5.00E-10	SLC39A8	Central nucleus volume	GCST90310229
rs11736322	5.00E-10	BANK1,SLC39A8	Central nucleus volume	GCST90310219
rs13107325	5.00E-10	SLC39A8	Regional cortical thickness (postcentral)	GCST90399898
rs12512546	5.00E-10	BANK1	Systolic blood pressure (MTAG)	GCST90449056
rs13107325	5.00E-10	SLC39A8	Self-reported math ability	GCST006573
rs11941622	5.00E-10	BANK1,SLC39A8	Hand grip strength (baseline)	GCST90565845
rs13107325	5.00E-10	SLC39A8	Occupational income	GCST90565739
rs112623404	5.00E-10	UBE2D3	Smoking initiation	GCST90243968
rs13109404	5.00E-10	BANK1	Osteoarthritis	GCST90134288
rs13105682	5.00E-10	BANK1	Osteoarthritis	GCST90134284
rs13107325	5.00E-10	SLC39A8	Osteoarthritis (with total hip replacement)	GCST90566802
rs10017306	5.00E-10	SLC39A8	Volume of left cortical nucleus	GCST90474885
rs4648051	5.00E-10	NFKB1	Inflammatory or infectious upper respiratory disease	GCST90269783
rs34592089	5.00E-10	BANK1	Fluid intelligence	GCST90448240
rs13105581	5.00E-10	SLC39A8	Short sleep duration (<5 hours)	GCST90428611
rs13107325	6.00E-10	SLC39A8	Systolic blood pressure	GCST004776
rs13107325	6.00E-10	SLC39A8	LDL cholesterol levels	GCST010245
rs13107325	6.00E-10	SLC39A8	NT-proBNP levels in acute coronary syndrome	GCST003298
rs17032400	6.00E-10	SLC39A8	Intelligence (MTAG)	GCST005316
rs7674212	6.00E-10	SLC9B2	Type 2 diabetes	GCST006867
rs35225200	6.00E-10	BANK1,SLC39A8	Phospholipids to total lipids ratio in very large HDL	GCST90093013
rs13105682	6.00E-10	BANK1	Cortical nucleus volume	GCST90310228
rs74332078	6.00E-10	UBE2D3	Substantia nigra iron levels (R2* MRI)	GCST90551872
rs11941622	6.00E-10	BANK1,SLC39A8	Volume of right central nucleus	GCST90475027
rs13107325	6.00E-10	SLC39A8	Volume of right whole amygdala	GCST90475057
rs13107325	6.00E-10	SLC39A8	Serum urate levels	GCST90319904
rs34592089	7.00E-10	BANK1	Brain region volumes	GCST009518
rs17033015	7.00E-10	MANBA,NFKB1	Hay fever and/or eczema	GCST009717
rs13107325	7.00E-10	SLC39A8	IDP dMRI TBSS OD Anterior limb of internal capsule R	GCST90004418
rs13107325	7.00E-10	SLC39A8	Phosphoglycerides levels	GCST90092938
rs6846971	7.00E-10	NFKB1,SLC39A8	Anterior amygdaloid area volume	GCST90310230
rs151381	7.00E-10	BANK1,SLC39A8	Educational attainment (years of education)	GCST006442
rs13126505	7.00E-10	BANK1	Diastolic blood pressure x low social support interaction (2df test)	GCST90093172
rs13135092	7.00E-10	SLC39A8	Dietary macronutrient intake (multi-trait analysis)	GCST90032651
rs13107325	7.00E-10	SLC39A8	Intelligence	GCST90179114
rs4371620	7.00E-10	BANK1	Educational attainment	GCST90105038
rs6855246	7.00E-10	SLC39A8,BANK1	Inflammatory bowel disease or schizophrenia (pleiotropy)	GCST90271322
rs13107325	7.00E-10	SLC39A8	Non-response to survey questionnaires: "prefer not to answer" item	GCST90266936
rs10017306	7.00E-10	SLC39A8	Volume of left medial nucleus	GCST90474891
rs10017306	7.00E-10	SLC39A8	Volume of right central nucleus	GCST90475023
rs998719,rs111969	7.00E-10	SLC39A8 x FHIP2A	Total PHF-tau (SNP x SNP interaction)	GCST010340
rs13107325	8.00E-10	SLC39A8	Body mass index (joint analysis main effects and physical activity interaction)	GCST004558
rs13135092	8.00E-10	SLC39A8	Total cholesterol levels	GCST007143
rs13107325	8.00E-10	SLC39A8	Nucleus accumbens volume	GCST009670
rs1054037	8.00E-10	MANBA	Primary biliary cholangitis	GCST003129
rs13107325	8.00E-10	SLC39A8	wg lh intensity-contrast posteriorcingulate	GCST90003814
rs13107325	8.00E-10	SLC39A8	Alanine aminotransferase levels	GCST90020236
rs17199964	8.00E-10	BANK1	Insomnia	GCST90131901
rs13117052	8.00E-10	UBE2D3-AS1,UBE2D3	Alanine aminotransferase levels	GCST90244009
rs13126505	8.00E-10	BANK1	Osteoarthritis	GCST90134286
rs223346	8.00E-10	UBE2D3	Schizophrenia vs ADHD (ordinary least squares (OLS))	GCST90016618
rs201081507	8.00E-10	BANK1	Volume of left medial nucleus	GCST90474891
rs372682919	8.00E-10	SLC39A8	Volume of left medial nucleus	GCST90474892
rs57957741	8.00E-10	BANK1,SLC39A8	Volume of right medial nucleus	GCST90475042
rs11097755	8.00E-10	BANK1	High density lipoprotein cholesterol levels	GCST90019510
rs6855246	9.00E-10	SLC39A8,BANK1	vWF levels	GCST007446
rs141936164	9.00E-10	SLC39A8,NFKB1	Mean corpuscular hemoglobin	GCST007068
rs13107325	9.00E-10	SLC39A8	Diastolic blood pressure	GCST005772
rs17033015	9.00E-10	MANBA,NFKB1	Primary biliary cholangitis	GCST007036
rs13107325	9.00E-10	SLC39A8	Genetically independent pain phenotypes (GIP1)	GCST90245879
rs4699258	9.00E-10	BANK1	Volume of left central nucleus	GCST90474958
rs6532988	9.00E-10	SLC39A8,BANK1	Alcohol use disorder (MTAG)	GCST90296428
rs77252379	9.00E-10	SLC39A8	Non-albumin protein levels	GCST90019515
rs13107325	1.00E-09	SLC39A8	Body mass index in physically active individuals	GCST004559
rs13107325	1.00E-09	SLC39A8	Body mass index	GCST004557
rs13107325	1.00E-09	SLC39A8	Total cholesterol levels	GCST007143
rs34592089	1.00E-09	BANK1	Hypertension	GCST009685
rs233811	1.00E-09	SLC39A8	General cognitive ability	GCST006269

rs200777151	1.00E-09	SLC9B2	Estimated glomerular filtration rate in non-diabetics	GCST008745
rs11097789	1.00E-09	NFKB1	Monocyte percentage of white cells	GCST004609
rs223498	1.00E-09	MANBA	Sarcoidosis	GCST005538
rs6855246	1.00E-09	SLC39A8,BANK1	Autism spectrum disorder or schizophrenia	GCST004521
rs62328536	1.00E-09	SLC39A8,NFKB1	Mean corpuscular hemoglobin	GCST90018964
rs13107325	1.00E-09	SLC39A8	wg rh intensity-contrast paracentral	GCST90003843
rs13107325	1.00E-09	SLC39A8	wg lh intensity-contrast supramarginal	GCST90003822
rs13107325	1.00E-09	SLC39A8	wg lh intensity-contrast parsopercularis	GCST90003809
rs13109404	1.00E-09	BANK1	Multisite chronic pain	GCST012332
rs11736123	1.00E-09	SLC39A8	Central nucleus volume	GCST90310229
rs181397936	1.00E-09	BANK1	Central nucleus volume	GCST90310229
rs11736322	1.00E-09	BANK1,SLC39A8	Central nucleus volume	GCST90310229
rs13107325	1.00E-09	SLC39A8	Basal nucleus volume	GCST90310223
rs13101632	1.00E-09	BANK1,SLC39A8	Accessory basal nucleus volume	GCST90310224
rs13107325	1.00E-09	SLC39A8	Brussel sprout liking	GCST90094714
rs1027013	1.00E-09	BANK1	Age at menarche	GCST90451680
rs10516497	1.00E-09	SLC9B2	Body mass index (MTAG)	GCST90179150
rs75877836	1.00E-09	LINC02428	Pallidum iron levels (R2* MRI)	GCST90551868
rs61161339	1.00E-09	SLC39A8,BANK1	Volume of left central nucleus	GCST90474883
rs150228862	1.00E-09	BANK1	Volume of left medial nucleus	GCST90474892
rs10017306	1.00E-09	SLC39A8	Volume of left medial nucleus	GCST90474892
rs10017306	1.00E-09	SLC39A8	Volume of right central nucleus	GCST90475024
rs10017306	1.00E-09	SLC39A8	Volume of right medial nucleus	GCST90475041
rs13118152	1.00E-09	SLC39A8	Volume of left medial nucleus	GCST90474975
rs4371620	1.00E-09	BANK1	Volume of right medial nucleus	GCST90474924
rs13101632	1.00E-09	BANK1,SLC39A8	Intelligence	GCST90264174
rs1506505	1.00E-09	BANK1,SLC39A8	Intelligence	GCST90264174
rs13107325	1.00E-09	SLC39A8	Tinnitus	GCST90428113
rs13107325	1.00E-09	SLC39A8	Right-hemisphere somatomotor network to amygdala white-matter structural connectivity	GCST90302799
rs35518360	1.00E-09	SLC39A8,BANK1	Free cholesterol to total lipids in large LDL percentage (UKB data field 23617)	GCST90269714
rs3774964	1.00E-09	NFKB1	Chronic diseases of tonsils or adenoids (MTAG)	GCST90269792
rs13107325	1.00E-09	SLC39A8	Serum urate levels	GCST90319906
rs35225200	1.00E-09	BANK1,SLC39A8	Degree of unsaturation	GCST90502225
rs13107325	2.00E-09	SLC39A8	Body mass index (joint analysis main effects and physical activity interaction)	GCST004558
rs230504	2.00E-09	NFKB1	Allergic disease (asthma, hay fever or eczema)	GCST009716
rs113473633	2.00E-09	NFKB1	Eosinophil percentage of white cells	GCST90002382
rs62327954	2.00E-09	SLC39A8	Mean spheric corpuscular volume	GCST90002397
rs13105581	2.00E-09	SLC39A8	Intelligence	GCST006250
rs17032400	2.00E-09	SLC39A8	General cognitive ability	GCST006269
rs17199964	2.00E-09	BANK1	General cognitive ability	GCST006269
rs17199964	2.00E-09	BANK1	Intelligence (MTAG)	GCST005316
rs13135092	2.00E-09	SLC39A8	Trihexosylcermide (d18:1/18:0) levels	GCST90024115
rs13107325	2.00E-09	SLC39A8	wg lh intensity-contrast inferiortemporal	GCST90003800
rs13135092	2.00E-09	SLC39A8	Multisite chronic pain	GCST012334
rs1813006	2.00E-09	BANK1,SLC39A8	Free cholesterol to total lipids ratio in medium LDL	GCST90092909
rs12511373	2.00E-09	BANK1	Anterior amygdaloid area volume	GCST90310230
rs13107325	2.00E-09	SLC39A8	Leisure screen time	GCST90104339
rs223423	2.00E-09	UBE2D3	Type 2 diabetes	GCST90132183
rs58611096	2.00E-09	LINC02428	Male puberty timing (late vs. average onset facial hair)	GCST90012085
rs13105682	2.00E-09	BANK1	Osteoarthritis	GCST90134286
rs184046517	2.00E-09	SLC39A8	Volume of left central nucleus	GCST90474883
rs238449	2.00E-09	BANK1,SLC39A8	Volume of left medial nucleus	GCST90474891
rs13103835	2.00E-09	SLC39A8	Volume of left medial nucleus	GCST90474892
rs10017306	2.00E-09	SLC39A8	Volume of right accessory basal nucleus	GCST90474906
rs13107325	2.00E-09	SLC39A8	Volume of left accessory basal nucleus	GCST90474943
rs11941622	2.00E-09	BANK1,SLC39A8	Volume of right medial nucleus	GCST90474924
rs17249745	2.00E-09	BANK1	Volume of right medial nucleus	GCST90474925
rs10017306	2.00E-09	SLC39A8	Volume of left accessory basal nucleus	GCST90474873
rs13107325	2.00E-09	SLC39A8	Pelvic height	GCST90448912
rs10031172	2.00E-09	BANK1	Substance use disorder (pleiotropy)	GCST90274726
rs13107325	2.00E-09	SLC39A8	Left-hemisphere somatomotor network to amygdala white-matter structural connectivity	GCST90302687
rs755492124	2.00E-09	BANK1	Mitochondrial DNA copy number (adjusted)	GCST90268497
rs13107325	3.00E-09	SLC39A8	Body mass index	GCST002783
rs34333163	3.00E-09	SLC39A8	Brain region volumes	GCST009518
rs13107325	3.00E-09	SLC39A8	Systolic blood pressure	GCST007095
rs238449	3.00E-09	BANK1,SLC39A8	Intelligence (MTAG)	GCST005316
rs223471	3.00E-09	UBE2D3,MANBA	Estimated glomerular filtration rate	GCST90446493
rs13107325	3.00E-09	SLC39A8	aparc-Desikan lh thickness precentral	GCST90003468
rs10017306	3.00E-09	SLC39A8	Central nucleus volume	GCST90310229
rs13107325	3.00E-09	SLC39A8	Triglycerides to total lipids ratio in very large HDL	GCST90093015
rs13118152	3.00E-09	SLC39A8	Accessory basal nucleus volume	GCST90310224
rs7695249	3.00E-09	SLC39A8	Diastolic blood pressure (MTAG)	GCST90449057
rs93059	3.00E-09	NFKB1	DNA methylation Hannum age acceleration	GCST90014289
rs13107325	3.00E-09	SLC39A8	Cognitive function (baseline)	GCST90565836
rs141936164	3.00E-09	SLC39A8,NFKB1	C-reactive protein levels	GCST90029070
rs13135092	3.00E-09	SLC39A8	Alcohol use and misuse (confirmatory factor analysis Factor 22)	GCST90309356

rs10516491	3.00E-09	BANK1	Height	GCST008053
rs113473633	3.00E-09	NFKB1	Eosinophil counts	GCST90056180
rs13107325	3.00E-09	SLC39A8	FVC x serum 25-hydroxyvitamin D interaction in ever smokers (2df test)	GCST90590344
rs13107325	3.00E-09	SLC39A8	Docosahexaenoic acid levels	GCST90501978
rs35225200	3.00E-09	BANK1,SLC39A8	Polyunsaturated fatty acids to monounsaturated fatty acids ratio	GCST90502554
rs12512546	3.00E-09	BANK1	Volume of left medial nucleus	GCST90474892
rs114614648	3.00E-09	BANK1	Volume of right central nucleus	GCST90475023
rs4698854	3.00E-09	NFKB1,SLC39A8	Mean volume of bilateral amygdala	GCST90474994
rs11941622	3.00E-09	BANK1,SLC39A8	Volume of right central nucleus	GCST90475026
rs181121136	3.00E-09	BANK1,SLC39A8	Volume of right cortical nucleus	GCST90475033
rs376206821	3.00E-09	BANK1	Volume of right cortical nucleus	GCST90474918
rs13107325	4.00E-09	SLC39A8	Body mass index in physically active individuals	GCST004559
rs13135092	4.00E-09	SLC39A8	White blood cell count	GCST007070
rs230503	4.00E-09	NFKB1	Immature fraction of reticulocytes	GCST90002387
rs13107325	4.00E-09	SLC39A8	BMI (adjusted for smoking behaviour)	GCST004495
rs113473633	4.00E-09	NFKB1	Eosinophil counts	GCST90018953
rs144759444	4.00E-09	SLC39A8	LGALS3 protein levels	GCST90277685
rs13107325	4.00E-09	SLC39A8	wg lh intensity-contrast parahippocampal	GCST90003807
rs13107325	4.00E-09	SLC39A8	AmygNuclei lh volume Accessory-Basal-nucleus	GCST90002650
rs13107325	4.00E-09	SLC39A8	aparc-DKTatlas lh thickness precentral	GCST90003562
rs13107325	4.00E-09	SLC39A8	Cholesteryl esters to total lipids ratio in medium HDL	GCST90092895
rs13135092	4.00E-09	SLC39A8	Total cholesterol levels	GCST006614
rs13107325	4.00E-09	SLC39A8	Total phospholipid levels in lipoprotein particles	GCST90092991
rs13107325	4.00E-09	SLC39A8	Age when finished full-time education (standard GWA)	GCST90267280
rs4013	4.00E-09	MANBA	QT interval	GCST90179153
rs10600502	4.00E-09	BANK1	Blood cell traits latent factor 16 (white cell)	GCST90559258
rs13107325	4.00E-09	SLC39A8	Mean volume of bilateral amygdala	GCST90474997
rs10017306	4.00E-09	SLC39A8	Volume of right central nucleus	GCST90475026
rs4698854	4.00E-09	NFKB1,SLC39A8	Volume of right whole amygdala	GCST90475054
rs139919861	4.00E-09	BANK1	Volume of right medial nucleus	GCST90475042
rs201081507	4.00E-09	BANK1	Volume of right medial nucleus	GCST90475044
rs223345	4.00E-09	UBE2D3	Alanine aminotransferase levels	GCST90428729
rs2125213	4.00E-09	SLC39A8,NFKB1	Abdominal aortic aneurysm	GCST90432152
rs34592089	4.00E-09	BANK1	Low density lipoprotein cholesterol levels	GCST90019512
rs6855246	5.00E-09	SLC39A8,BANK1	Brain region volumes	GCST009518
rs17199964	5.00E-09	BANK1	Brain region volumes	GCST009518
rs113473633	5.00E-09	NFKB1	Neutrophil percentage of granulocytes	GCST004623
rs13107325	5.00E-09	SLC39A8	Schizophrenia (MTAG)	GCST010645
rs223490	5.00E-09	MANBA	Estimated glomerular filtration rate	GCST90446493
rs11097755	5.00E-09	BANK1	Central nucleus volume	GCST90310229
rs151379	5.00E-09	BANK1,SLC39A8	Central nucleus volume	GCST90310229
rs230534	5.00E-09	NFKB1	Systemic sclerosis	GCST009131
rs13103835	5.00E-09	SLC39A8	High-density lipoprotein levels (MTAG)	GCST90179147
rs223337	5.00E-09	UBE2D3	Migraine or glucose levels	GCST90281115
rs11941622	5.00E-09	BANK1,SLC39A8	Volume of left medial nucleus	GCST90474891
rs80136231	5.00E-09	BANK1	Volume of left central nucleus	GCST90474883
rs63519	5.00E-09	SLC39A8	Volume of right cortical nucleus	GCST90475032
rs11941622	5.00E-09	BANK1,SLC39A8	Volume of right medial nucleus	GCST90475044
rs13107325	5.00E-09	SLC39A8	Volume of right basal nucleus	GCST90474910
rs58477215	6.00E-09	BDH2	Pulse pressure	GCST007269
rs7667281	6.00E-09	SLC39A8,BANK1	Intelligence (MTAG)	GCST005316
rs113473633	6.00E-09	NFKB1	Eosinophil percentage of white cells	GCST004600
rs223454	6.00E-09	MANBA,UBE2D3	Alanine aminotransferase levels	GCST90011898
rs13107325	6.00E-09	SLC39A8	Cholesterol to total lipids ratio in medium LDL	GCST90092905
rs200911786	6.00E-09	CENPE,LINC02428	Triglycerides	GCST006613
rs35518360	6.00E-09	SLC39A8,BANK1	Phospholipids to total lipids ratio in IDL	GCST90092840
rs13107325	6.00E-09	SLC39A8	Cholesteryl esters to total lipids ratio in large HDL	GCST90092847
rs577329140	6.00E-09	NFKB1,SLC39A8	Insomnia	GCST90131901
rs1598856	6.00E-09	NFKB1	JT interval	GCST90179157
rs13105682	6.00E-09	BANK1	Osteoarthritis	GCST90134288
rs4645215	6.00E-09	SLC9B1	Migraine or type 1 diabetes	GCST90281118
rs13135092	6.00E-09	SLC39A8	Schizophrenia vs anorexia nervosa (ordinary least squares (OLS))	GCST90016619
rs10017306	6.00E-09	SLC39A8	Volume of left cortical nucleus	GCST90474886
rs12501273	6.00E-09	BANK1	Volume of right central nucleus	GCST90475024
rs139919861	6.00E-09	BANK1	Volume of right cortical nucleus	GCST90475030
rs150228862	6.00E-09	BANK1	Volume of right medial nucleus	GCST90475045
rs62688361	6.00E-09	BANK1	Volume of right medial nucleus	GCST90475041
rs12512546	6.00E-09	BANK1	Volume of left medial nucleus	GCST90474976
rs10017306	6.00E-09	SLC39A8	Volume of left accessory basal nucleus	GCST90474874
rs13107325	6.00E-09	SLC39A8	Post-traumatic stress disorder (MTAG)	GCST90297545
rs13101632	7.00E-09	BANK1,SLC39A8	Brain region volumes	GCST009518
rs13107325	7.00E-09	SLC39A8	Alcohol consumption (heavy vs. light/non-drinkers)	GCST009797
rs230489	7.00E-09	SLC39A8,NFKB1	Intelligence (MTAG)	GCST005316
rs7674212	7.00E-09	SLC9B2	Type 2 diabetes	GCST004894
rs230539	7.00E-09	NFKB1	Mean corpuscular hemoglobin concentration	GCST90002391
rs4648052	7.00E-09	NFKB1	Allergic disease (asthma, hay fever and/or eczema) (multivariate analysis)	GCST010984

rs4648052	7.00E-09	NFKB1	Allergic disease (asthma, hay fever and/or eczema) (age of onset)	GCST010985
rs13105682	7.00E-09	BANK1	IDP dMRI TBSS OD Anterior limb of internal capsule L	GCST90004419
rs13107325	7.00E-09	SLC39A8	Free cholesterol levels in very large HDL	GCST90093008
rs13135092	7.00E-09	SLC39A8	F-salad vegetables liking (derived food-liking factor)	GCST90094824
rs1027013	7.00E-09	BANK1	Age at menarche	GCST90451679
rs13130741	7.00E-09	SLC9B2	Medication use (agents acting on the renin-angiotensin system)	GCST007930
rs7667281	7.00E-09	SLC39A8,BANK1	Multi-trait sex score	GCST90270118
rs10017306	7.00E-09	SLC39A8	Volume of right central nucleus	GCST90475027
rs10017306	7.00E-09	SLC39A8	Volume of right accessory basal nucleus	GCST90474907
rs11446392	7.00E-09	SLC39A8	Volume of left central nucleus	GCST90474960
rs35978636	7.00E-09	BANK1	Mitochondrial DNA copy number (adjusted)	GCST90268497
rs13109404	7.00E-09	BANK1	Short sleep duration (<5 hours)	GCST90428613
rs13109404	7.00E-09	BANK1	Short sleep duration (<5 hours)	GCST90428611
rs151412	8.00E-09	BANK1,SLC39A8	Intelligence	GCST006250
rs13105682	8.00E-09	BANK1	IDP dMRI TBSS MO Cerebral peduncle R	GCST90003966
rs13107325	8.00E-09	SLC39A8	Node-level brain connectivity (multivariate analysis)	GCST90165317
rs2850387	8.00E-09	BANK1	Gout	GCST90455676
rs10017313	8.00E-09	SLC9B1	Triglyceride levels	GCST90239664
rs13109272	8.00E-09	SLC39A8	Osteoarthritis	GCST90134288
rs11941622	8.00E-09	BANK1,SLC39A8	Volume of left central nucleus	GCST90474882
rs150228862	8.00E-09	BANK1	Volume of right central nucleus	GCST90475027
rs1811810	8.00E-09	NFKB1,SLC39A8	Volume of right whole amygdala	GCST90474930
rs114601774	8.00E-09	LINC02428	Volume of right central nucleus	GCST90474916
rs6822371,rs61140	8.00E-09	SLC39A8 x SRGAP3	Total PHF-tau (SNP x SNP interaction)	GCST010340
rs13107325	9.00E-09	SLC39A8	Estimated glomerular filtration rate (creatinine)	GCST90100220
rs201081507	9.00E-09	BANK1	Multisite chronic pain	GCST012332
rs13107325	9.00E-09	SLC39A8	Ratio of monounsaturated fatty acids to total fatty acids	GCST90092929
rs4698839	9.00E-09	BANK1	Mitochondrial DNA copy number	GCST90026371
rs13107325	9.00E-09	SLC39A8	Body mass index z-score	GCST90502757
rs62688361	9.00E-09	BANK1	Volume of right central nucleus	GCST90475023
rs13107325	1.00E-08	SLC39A8	Systolic blood pressure	GCST007095
rs13135092	1.00E-08	SLC39A8	Obese vs. thin	GCST007240
rs13135092	1.00E-08	SLC39A8	Bipolar disorder (MTAG)	GCST010641
rs1506505	1.00E-08	BANK1,SLC39A8	Intelligence	GCST006250
rs142592088	1.00E-08	NFKB1,SLC39A8	Serum total protein levels	GCST005989
rs7665090	1.00E-08	MANBA,NFKB1	Multiple sclerosis	GCST005531
rs13107325	1.00E-08	SLC39A8	Childhood body mass index	GCST003177
rs13107325	1.00E-08	SLC39A8	Body mass index (joint analysis main effects and smoking interaction)	GCST004497
rs113473633	1.00E-08	NFKB1	Medication use (thyroid preparations)	GCST90018990
rs223490	1.00E-08	MANBA	Alanine aminotransferase levels	GCST90018943
rs223413	1.00E-08	UBE2D3	Estimated glomerular filtration rate	GCST90446493
rs13107325	1.00E-08	SLC39A8	aparc-Desikan lh thickness GlobalMeanThickness	GCST90003445
rs13105682	1.00E-08	BANK1	IDP T1 FAST ROIs V cerebellum VIIIa	GCST90002579
rs7659650	1.00E-08	SLC9B1	Free cholesterol to total lipids ratio in very large VLDL	GCST90093021
rs13135092	1.00E-08	SLC39A8	Cauliflower liking	GCST90094727
rs13135092	1.00E-08	SLC39A8	Cabbage liking	GCST90094719
rs151404	1.00E-08	SLC39A8	Waist circumference adjusted for body mass index	GCST90020029
rs113473633	1.00E-08	NFKB1	Medication use (thyroid preparations)	GCST007932
rs13101632	1.00E-08	BANK1,SLC39A8	Alcohol frequency weekly (standard GWA)	GCST90267266
rs13105581	1.00E-08	SLC39A8	ttention deficit hyperactivity disorder or autism spectrum disorder or intelligence (pleiotropy	GCST90134330
rs227285	1.00E-08	MANBA	ttention deficit hyperactivity disorder or autism spectrum disorder or intelligence (pleiotropy	GCST90134330
rs13135092	1.00E-08	SLC39A8	Hippocampus iron levels (quantitative susceptibility mapping)	GCST90551865
rs13107325	1.00E-08	SLC39A8	Common executive function	GCST90162547
rs13107325	1.00E-08	SLC39A8	Multi-trait sum score	GCST90270119
rs13107325	1.00E-08	SLC39A8	Osteoarthritis	GCST90296705
rs6850893	1.00E-08	SLC39A8	Volume of left central nucleus	GCST90474882
rs13118152	1.00E-08	SLC39A8	Volume of right central nucleus	GCST90475026
rs12511373	1.00E-08	BANK1	Volume of right cortical nucleus	GCST90475029
rs11941622	1.00E-08	BANK1,SLC39A8	Volume of right cortical nucleus	GCST90475033
rs1811810	1.00E-08	NFKB1,SLC39A8	Volume of left whole amygdala	GCST90474897
rs75088572	1.00E-08	SLC39A8,BANK1	Volume of right medial nucleus	GCST90475041
rs10017306	1.00E-08	SLC39A8	Volume of left central nucleus	GCST90474958
rs12511373	1.00E-08	BANK1	Volume of left medial nucleus	GCST90474975
rs2851245	1.00E-08	BANK1,SLC39A8	Volume of left medial nucleus	GCST90474975
rs34670044	1.00E-08	BANK1,SLC39A8	Volume of left medial nucleus	GCST90474976
rs10031415	1.00E-08	SLC39A8,NFKB1	Volume of left basal nucleus	GCST90474877
rs62329496	1.00E-08	SLC39A8	Volume of right central nucleus	GCST90474916
rs11941622	1.00E-08	BANK1,SLC39A8	Volume of right cortical nucleus	GCST90474918
rs13103835	1.00E-08	SLC39A8	Volume of right cortical nucleus	GCST90474919
rs57957741	1.00E-08	BANK1,SLC39A8	Volume of right cortical nucleus	GCST90474919
rs6855246	1.00E-08	SLC39A8,BANK1	Occipital area (unadjusted for global measures)	GCST90271795
rs13114738	1.00E-08	SLC39A8	Pain (pleiotropy)	GCST90104572
rs11734042	1.00E-08	BANK1,SLC39A8	Body mass index	GCST90255621
rs922361,rs109785	1.00E-08	SLC39A8 x LINC01505	Total PHF-tau (SNP x SNP interaction)	GCST010340
rs13107325	2.00E-08	SLC39A8	Body mass index (joint analysis main effects and physical activity interaction)	GCST004558
rs62322696	2.00E-08	BANK1	Adventurousness	GCST007324

rs151411	2.00E-08	SLC39A8,BANK1	General cognitive ability	GCST006269
rs10031823	2.00E-08	BANK1,SLC39A8	General cognitive ability	GCST006269
rs4648050	2.00E-08	NFKB1	Allergic sensitization	GCST006408
rs223401	2.00E-08	UBE2D3	Estimated glomerular filtration rate	GCST007344
rs13135092	2.00E-08	SLC39A8	Bipolar disorder (MTAG)	GCST012091
rs13105682	2.00E-08	BANK1	IDP dMRI TBSS MO Cerebral peduncle L	GCST90003967
rs13105682	2.00E-08	BANK1	aseg rh volume Pallidum	GCST90002640
rs9884397	2.00E-08	SLC9B1	Cholesterol to total lipids ratio in medium VLDL	GCST90092917
rs13107325	2.00E-08	SLC39A8	Triglycerides to total lipids ratio in medium LDL	GCST90092915
rs9884397	2.00E-08	SLC9B1	Cholesteryl esters to total lipids ratio in medium VLDL	GCST90092919
rs28484580	2.00E-08	NFKB1	Total testosterone levels	GCST90012112
rs72917071	2.00E-08	BANK1	Bacteroides eggerthii abundance in stool	GCST90032210
rs13107325	2.00E-08	SLC39A8	Regional cortical thickness (frontalpole)	GCST90399909
rs13107325	2.00E-08	SLC39A8	Educational attainment (confirmatory factor analysis Factor 10)	GCST90309344
rs13103835	2.00E-08	SLC39A8	Height (baseline)	GCST90565843
rs577329140	2.00E-08	NFKB1,SLC39A8	Insomnia	GCST90131903
rs7694724	2.00E-08	SLC9B2	Schizophrenia	GCST90128471
rs13107325	2.00E-08	SLC39A8	Neck pain or shoulder pain	GCST90245883
rs13103835	2.00E-08	SLC39A8	Prostate cancer	GCST90274713
rs6822371	2.00E-08	SLC39A8	Body mass index (MTAG)	GCST90179150
rs6833764	2.00E-08	BANK1	Resistance to COVID-19 infection (Exposed negative vs positive)	GCST90255358
rs114601774	2.00E-08	LINC02428	Substantia nigra iron levels (R2* MRI)	GCST90551872
rs201081507	2.00E-08	BANK1	Accumbens iron levels (quantitative susceptibility mapping)	GCST90551859
rs13107325	2.00E-08	SLC39A8	Pulse pressure	GCST90310296
rs227378	2.00E-08	MANBA	ADHD vs obsessive compulsive disorder (ordinary least squares (OLS))	GCST90016597
rs181121136	2.00E-08	BANK1,SLC39A8	Volume of left cortical nucleus	GCST90474886
rs62688361	2.00E-08	BANK1	Volume of left central nucleus	GCST90474882
rs2126627	2.00E-08	NFKB1,SLC39A8	Volume of left central nucleus	GCST90474883
rs62688361	2.00E-08	BANK1	Volume of left central nucleus	GCST90474883
rs13118152	2.00E-08	SLC39A8	Volume of left cortical nucleus	GCST90474885
rs62688361	2.00E-08	BANK1	Volume of right central nucleus	GCST90475024
rs1314336	2.00E-08	SLC39A8,NFKB1	Mean volume of bilateral amygdala	GCST90474901
rs12511373	2.00E-08	BANK1	Volume of right medial nucleus	GCST90475041
rs150228862	2.00E-08	BANK1	Volume of right medial nucleus	GCST90475042
rs181121136	2.00E-08	BANK1,SLC39A8	Volume of left central nucleus	GCST90474958
rs17249745	2.00E-08	BANK1	Volume of left central nucleus	GCST90474958
rs12510679	2.00E-08	SLC39A8	Volume of left medial nucleus	GCST90474975
rs181121136	2.00E-08	BANK1,SLC39A8	Volume of left medial nucleus	GCST90474976
rs372682919	2.00E-08	SLC39A8	Volume of right central nucleus	GCST90474916
rs233806	2.00E-08	SLC39A8	Heart failure	GCST90274223
rs13107325	2.00E-08	SLC39A8	Medial temporal thickness (unadjusted for global measures)	GCST90271805
rs6532988	2.00E-08	SLC39A8,BANK1	Smoking initiation (MTAG)	GCST90296430
rs223497	2.00E-08	MANBA	Headache or glucose levels	GCST90281121
rs13114738	2.00E-08	SLC39A8	Short sleep duration (<5 hours)	GCST90428613
rs223484	2.00E-08	UBE2D3,MANBA	Creatinine levels	GCST90278624
rs227283	3.00E-08	MANBA	Asthma and attention deficit hyperactivity disorder	GCST008919
rs34592089	3.00E-08	BANK1	Brain region volumes	GCST009518
rs230489	3.00E-08	SLC39A8,NFKB1	Brain region volumes	GCST009518
rs62327949	3.00E-08	SLC39A8	Intelligence	GCST006250
rs11722779	3.00E-08	SLC9B1	Schizophrenia	GCST004946
rs13107325	3.00E-08	SLC39A8	Type 2 diabetes	GCST010557
rs35225200	3.00E-08	BANK1,SLC39A8	Low hand grip strength (60 years and older) (EWGSOP)	GCST90007528
rs2203640	3.00E-08	LINC02428	Adipsin levels	GCST90091147
rs7659650	3.00E-08	SLC9B1	Free cholesterol to total lipids ratio in medium VLDL	GCST90092921
rs7659650	3.00E-08	SLC9B1	Triglycerides to total lipids ratio in very large VLDL	GCST90093027
rs56095122	3.00E-08	UBE2D3	Eosinophil counts	GCST90134621
rs13107325	3.00E-08	SLC39A8	Regional cortical thickness (insula)	GCST90399911
rs12511373	3.00E-08	BANK1	Decaffeinated coffee consumption and/or lifetime cannabis use	GCST90134528
rs13107325	3.00E-08	SLC39A8	FEV1 or gastro-oesophageal reflux disease (pleiotropy)	GCST90454172
rs17199964	3.00E-08	BANK1	Lipid traits (pleiotropy) (HIPO component 1)	GCST006666
rs1813006	3.00E-08	BANK1,SLC39A8	Diastolic blood pressure x depressive symptoms interaction (2df test)	GCST90093171
rs17032400	3.00E-08	SLC39A8	Body mass index (MTAG)	GCST90179150
rs12506564	3.00E-08	BANK1	Hand grip strength (baseline)	GCST90565845
rs4371620	3.00E-08	BANK1	FVC x serum 25-hydroxyvitamin D interaction (2df test)	GCST90590345
rs13135092	3.00E-08	SLC39A8	Back pain	GCST90245851
rs13107325	3.00E-08	SLC39A8	Osteoarthritis	GCST90296701
rs223332	3.00E-08	CISD2	Schizophrenia vs autism spectrum disorder (ordinary least squares (OLS))	GCST90016620
rs11931658	3.00E-08	BANK1	Volume of left medial nucleus	GCST90474892
rs34670044	3.00E-08	BANK1,SLC39A8	Volume of left medial nucleus	GCST90474892
rs12511373	3.00E-08	BANK1	Volume of right central nucleus	GCST90475026
rs376206821	3.00E-08	BANK1	Volume of right cortical nucleus	GCST90475029
rs17249745	3.00E-08	BANK1	Volume of right cortical nucleus	GCST90475030
rs201081507	3.00E-08	BANK1	Volume of right cortical nucleus	GCST90475032
rs75088572	3.00E-08	SLC39A8,BANK1	Volume of right medial nucleus	GCST90475042
rs2851245	3.00E-08	BANK1,SLC39A8	Volume of left central nucleus	GCST90474957
rs75088572	3.00E-08	SLC39A8,BANK1	Volume of right cortical nucleus	GCST90474918

rs17249745	3.00E-08	BANK1	Volume of right cortical nucleus	GCST90474919
rs13135092	3.00E-08	SLC39A8	Multi-trait sex score	GCST90270116
rs6532988	3.00E-08	SLC39A8,BANK1	Cannabis use disorder (MTAG)	GCST90296427
rs112519623	3.00E-08	SLC39A8	Diastolic blood pressure	GCST90310295
rs34592089	3.00E-08	BANK1	Monounsaturated fatty acids to total fatty acids percentage	GCST90502043
rs13135092	4.00E-08	SLC39A8	HDL cholesterol levels	GCST008070
rs13126505	4.00E-08	BANK1	Crohn's disease	GCST004132
rs13101632	4.00E-08	BANK1,SLC39A8	General cognitive ability	GCST006269
rs4698969	4.00E-08	BANK1	Metabolic syndrome	GCST90444487
rs371681307	4.00E-08	MANBA,NFKB1	Chronotype	GCST004696
rs116445911	4.00E-08	NFKB1,SLC39A8	Theta EEG coherence	GCST010464
rs13107325	4.00E-08	SLC39A8	Shoulder impingement or rotator cuff tear	GCST011377
rs10006327	4.00E-08	SLC9B1	Atrial fibrillation	GCST006414
rs13107325	4.00E-08	SLC39A8	Childhood body mass index	GCST90002409
rs9884397	4.00E-08	SLC9B1	Triglycerides to total lipids ratio in medium VLDL	GCST90092927
rs11722779	4.00E-08	SLC9B1	Schizophrenia	GCST007201
rs12511373	4.00E-08	BANK1	Dorsolateral prefrontal thickness	GCST90572705
rs223423	4.00E-08	UBE2D3	Type 2 diabetes	GCST90132184
rs71621627	4.00E-08	NFKB1,SLC39A8	Duration to complete alphanumeric path task (baseline)	GCST90565839
rs7659556	4.00E-08	LINC02428,CENPE	Testicular cancer	GCST90011805
rs3960788	4.00E-08	SLC9B1	Atrial fibrillation (MTAG)	GCST90132229
rs12511373	4.00E-08	BANK1	Height (baseline)	GCST90565843
rs233829	4.00E-08	SLC39A8	Insomnia	GCST90131903
rs13135092	4.00E-08	SLC39A8	Chronic obstructive pulmonary disease or gastroesophageal reflux disease (MTAG)	GCST90570623
rs223480	4.00E-08	UBE2D3,MANBA	Creatinine levels	GCST90239628
rs13107325	4.00E-08	SLC39A8	Gastroesophageal reflux disease or posttraumatic stress disorder (pleiotropy)	GCST90271338
rs13135092	4.00E-08	SLC39A8	Schizophrenia vs ADHD (ordinary least squares (OLS))	GCST90016618
rs114601774	4.00E-08	LINC02428	Volume of left medial nucleus	GCST90474892
rs2851245	4.00E-08	BANK1,SLC39A8	Volume of right central nucleus	GCST90475023
rs768027456	4.00E-08	BANK1	Volume of right cortical nucleus	GCST90475032
rs768027456	4.00E-08	BANK1	Volume of right medial nucleus	GCST90475044
rs13119968	4.00E-08	SLC39A8	Volume of left central nucleus	GCST90474957
rs223486	4.00E-08	UBE2D3,MANBA	Fasting glucose	GCST012078
rs6849365	4.00E-08	NFKB1,SLC39A8	Ventromedial occipital thickness (unadjusted for global measures)	GCST90271813
rs57957741	4.00E-08	BANK1,SLC39A8	Multi-trait sex score	GCST90270118
rs13140486	4.00E-08	SLC39A8,NFKB1	emisphere salience/ventral attention network to hippocampus white-matter structural conn	GCST90302821
rs13135092	4.00E-08	SLC39A8	Right-hemisphere limbic network to caudate white-matter structural connectivity	GCST90302828
rs6855246	4.00E-08	SLC39A8,BANK1	Short sleep duration (<5 hours)	GCST90428611

Supplemental Table 13. Genome-wide significant associations reported in the GWAS catalog within hg19 chr19:51,259,179-51,903,804 (hg38 chr19:50,755,921-51,400,550).

Variant	P Value	Mapped Genes	Reported Trait	Study Accession
rs2075803	4.00E-303	SIGLEC9	Sialic acid-binding Ig-like lectin 9 levels	GCST90161587
rs12983058	3.00E-298	SIGLEC9	SIGLEC7 protein levels	GCST90470635
rs12459419	1.00E-291	CD33	Myeloid cell surface antigen CD33 levels	GCST90161642
rs117268623	4.00E-289	KLK11	kallikrein-11 levels	GCST90012012
rs2075803	2.00E-280	SIGLEC9	Serum uromodulin levels (aptamer-based assay)	GCST90129632
rs62113212	2.00E-272	KLK3	Elevated prostate specific antigen [PSA] (PheCode 796)	GCST90480590
rs1048328	1.00E-261	KLK11	Blood protein levels	GCST006585
rs1654535	6.00E-258	KLK6,KLK7	KLK6/MOG protein level ratio	GCST90315255
rs62115757	1.00E-250	KLK10	Kallikrein-10 levels	GCST90426597
rs34093024	8.00E-231	KLK14,CTU1	KLK14 protein levels	GCST90469700
rs33978622	1.00E-223	CD33	Myeloid cell surface antigen CD33 levels	GCST90137742
rs2075803	3.00E-223	SIGLEC9	Serum levels of protein UMOD	GCST90090697
rs148028486	2.00E-221	KLK15,KLK3	KLK15 protein levels	GCST90469701
rs117533019	4.00E-214	SIGLEC22P,CD33	CD33 protein levels	GCST90468625
rs1654535	5.00E-214	KLK6,KLK7	KLK6/PTPRN2 protein level ratio	GCST90315256
rs3745540	6.00E-212	KLK12	Kallikrein-12 levels	GCST90161667
rs2075803	5.00E-208	SIGLEC9	Protein quantitative trait loci	GCST010900
rs140185670	4.00E-204	SIGLEC7	CD33 protein levels	GCST90468625
rs2411332	1.00E-199	KLK15,KLK3	KLK15 protein levels	GCST90469701
rs3865444	2.00E-191	SIGLEC22P,CD33	CD33 on CD33dim HLA DR+ CD11b+	GCST90001948
rs268891	5.00E-191	KLK6	KLK6 protein levels	GCST90469705
rs3865444	1.00E-190	SIGLEC22P,CD33	CD33 on CD14+ monocyte	GCST90001946
rs2569459	3.00E-189	KLK13,KLK12	Kallikrein-13 levels	GCST90248158
rs1035454	4.00E-189	VSIG10L-AS1	VSIG10L protein levels	GCST90471049
rs3865444	9.00E-189	SIGLEC22P,CD33	CD33 on CD33+ HLA DR+ CD14-	GCST90001957
rs3865444	3.00E-187	SIGLEC22P,CD33	CD33 on CD33+ HLA DR+	GCST90001956
rs2075803	8.00E-187	SIGLEC9	Uromodulin levels	GCST90250141
rs1048328	6.00E-186	KLK11	Kallikrein-11 levels (KLK11.2831.29.1)	GCST90241670
rs61752561,rs2668	9.00E-186	KLK3; KLK15 - KLK3; KLK15 - KLK3; KLK3; KLK15 - KLK3; KLK2; KLK2	Prostate-specific antigen levels	GCST004093
rs3865444	8.00E-184	SIGLEC22P,CD33	CD33 on CD33+ HLA DR+ CD14dim	GCST90001947
rs266881	9.00E-184	KLK3	KLK15 protein levels	GCST90469701
rs3865444	2.00E-183	SIGLEC22P,CD33	CD33 on CD33dim HLA DR+ CD11b-	GCST90001949
rs77342236	2.00E-182	KLK12,KLK13	KLK12 protein levels	GCST90469698
rs2075803	2.00E-180	SIGLEC9	Sialic acid-binding Ig-like lectin 9 level in Chronic kidney disease with hypertension and no diabetes (3007_7)	GCST90237195
rs62113212	9.00E-179	KLK3	Prostate cancer	GCST90274713
rs62117662	2.00E-178	KLK11	KLK11 protein levels	GCST90469697
rs1810020	8.00E-173	KLK3	Prostate-specific antigen levels	GCST90461907
rs62113212	1.00E-170	KLK3	Prostate cancer	GCST90274714
rs148022792	3.00E-168	KLK7	KLK7 protein levels	GCST90469706
rs12459419	3.00E-165	CD33	Myeloid cell surface antigen CD33 levels	GCST90101334
rs148089918	3.00E-165	SIGLEC22P,CD33	CD33 protein levels	GCST90468625
rs78018472	3.00E-165	KLK12	KLK12 protein levels	GCST90469698
rs112258520	3.00E-158	KLK3	KLK15 protein levels	GCST90469701
rs2075803	6.00E-158	SIGLEC9	Sialic acid-binding Ig-like lectin 9 levels	GCST90060056
rs577416273	3.00E-157	PPIAP59,KLK4	KLK4 protein levels	GCST90469704
rs17716134	2.00E-156	SIGLEC24P	CD33 protein levels	GCST90468625
rs2569454	3.00E-154	KLK10	Kallikrein-10 levels	GCST90179343
rs145472676	4.00E-154	KLK10	KLK10 protein levels	GCST90469696
rs2659067	5.00E-154	KLK7	Kallikrein-7 levels	GCST90248163
rs3865444	2.00E-153	SIGLEC22P,CD33	CD33 on Monocytic Myeloid-Derived Suppressor Cells	GCST90001952
rs1048328	6.00E-153	KLK11	Serum levels of protein KLK11	GCST90089823
rs7245846	2.00E-150	CD33	CD33 on basophil	GCST90001954
rs74705037	4.00E-150	KLK8	Kallikrein-8 levels	GCST90248164
rs266114	6.00E-148	KLK15	KLK1 protein levels	GCST90469702
rs2075803	3.00E-146	SIGLEC9	Sialic acid-binding Ig-like lectin 9 levels	GCST90137745
rs61729813	4.00E-146	KLK3	KLK4 protein levels	GCST90469704
rs3865444	3.00E-145	SIGLEC22P,CD33	CD33 on CD33dim HLA DR-	GCST90001953
rs7245846	6.00E-143	CD33	CD33 on Immature Myeloid-Derived Suppressor Cells	GCST90001955
rs117648753	8.00E-143	SIGLEC21P	CD33 protein levels	GCST90468625
rs11673588	2.00E-139	KLK9	KLK8 protein levels	GCST90469707
rs1048328	3.00E-136	KLK11	kallikrein-11 levels	GCST90161500
rs7255834	1.00E-133	KLK10	Kallikrein-10 levels	GCST90248155
rs149201050	1.00E-130	CD33	CD33 protein levels	GCST90468625
rs12983058	3.00E-129	SIGLEC9	T-cell surface glycoprotein CD5:Extracellular domain levels	GCST90426389
rs140185670	1.00E-126	SIGLEC7	Sialic acid-binding Ig-like lectin 7 levels	GCST90249551
rs11665748	4.00E-124	KLK15,KLK3	Prostate cancer	GCST90274713
rs1039405	3.00E-123	SIGLEC18P,SIGLEC9	Blood protein levels	GCST006585
rs117025461	5.00E-122	KLK10	KLK12 protein levels	GCST90469698
rs2075803	7.00E-122	SIGLEC9	Sialic acid-binding Ig-like lectin 9 levels	GCST90101552
rs1048328	2.00E-121	KLK11	Blood protein levels	GCST006585
rs2569491	4.00E-119	KLK14	Kallikrein-14 levels (KLK14.8620.56.3)	GCST90241672
rs3760738	2.00E-117	KLK11,KLK10	Serum levels of protein KLK10	GCST90089306
rs183055678	1.00E-116	LINC01872	CD33 protein levels	GCST90468625
rs201074739	5.00E-114	CD33	CD33 on CD14+ monocyte	GCST90001946
rs2569491	9.00E-114	KLK14	Serum levels of protein KLK14	GCST90090240
rs62115997	2.00E-113	SIGLECL1	Myeloid cell surface antigen CD33 levels	GCST90248431
rs61752561,rs2668	2.00E-111	KLK3; KLK15 - KLK3; KLK15 - KLK3; KLK3; KLK15 - KLK3; KLK2; KLK2	Prostate-specific antigen levels	GCST004093
rs7253072	2.00E-110	KLK13,KLK14	KLK13 protein levels	GCST90469699
rs3212815	3.00E-109	KLK1,KLK15	Kallikrein-15 levels	GCST90426727
rs3865444	2.00E-108	SIGLEC22P,CD33	CD33 on CD66b++ myeloid cell	GCST90001951
rs144350395	4.00E-107	KLK4	KLK4 protein levels	GCST90469704
rs76673459	5.00E-107	KLK14,CTU1	KLK14 protein levels	GCST90469700
rs142760794	6.00E-107	MIR8074,SIGLEC21P	CD33 on CD33dim HLA DR+ CD11b+	GCST90001948
rs867191	2.00E-106	KLK14	CD33 protein levels	GCST90468625
rs112343212	1.00E-103	KLK15,KLK3	Prostate-specific antigen levels	GCST90461907
rs142760794	1.00E-102	MIR8074,SIGLEC21P	CD33 on CD33+ HLA DR+	GCST90001956
rs62623429	2.00E-102	SIGLEC22P	CD33 protein levels	GCST90468625
rs142760794	5.00E-102	MIR8074,SIGLEC21P	CD33 on CD33+ HLA DR+ CD14-	GCST90001957
rs35996455	6.00E-102	KLK12	KLK10 protein levels	GCST90469696
rs142760794	1.00E-101	MIR8074,SIGLEC21P	CD33 on CD33+ HLA DR+ CD14dim	GCST90001947
rs11665748	2.00E-100	KLK15,KLK3	Prostate cancer	GCST90274714
rs142760794	1.00E-99	MIR8074,SIGLEC21P	CD33 on CD33dim HLA DR+ CD11b-	GCST90001949
rs268891	3.00E-99	KLK6	Kallikrein-6 levels	GCST90012008
rs112431762	2.00E-98	SIGLECL1	CD33 protein levels	GCST90468625
rs1710354	6.00E-98	SIGLEC24P	Serum levels of protein CD33	GCST90088239
rs2569459	2.00E-96	KLK13,KLK12	KLK14 protein levels	GCST90469700
rs12983058	2.00E-96	SIGLEC9	Sialic acid-binding Ig-like lectin 7 levels	GCST90249551
rs16988910	8.00E-96	SIGLEC9	Uromodulin level in Chronic kidney disease with hypertension and no diabetes (9451_20)	GCST90239359
rs142760794	2.00E-95	MIR8074,SIGLEC21P	CD33 on basophil	GCST90001954
rs1048328	2.00E-95	KLK11	kallikrein-11 levels	GCST90137609
rs2659053	5.00E-94	KLK3,KLK15	Prostate-specific antigen levels	GCST90301330
rs11669237	8.00E-92	KLK12	Kallikrein-12 levels	GCST90137650

rs142760794	2.00E-91	MIR8074,SIGLEC21P	CD33 on Monocytic Myeloid-Derived Suppressor Cells	GCST90001952
rs142760794	1.00E-90	MIR8074,SIGLEC21P	CD33 on CD33dim HLA DR-	GCST90001953
rs77136875	8.00E-90	KLK5,PPIAP59	KLK15 protein levels	GCST90469701
rs2075803	2.00E-89	SIGLEC9	Serum levels of protein CD5	GCST90089081
rs144628077	2.00E-88	KLKP1	KLK4 protein levels	GCST90469704
rs11549920	1.00E-86	KLK2	KLK4 protein levels	GCST90469704
rs2569491	3.00E-86	KLK14	Blood protein levels	GCST006585
rs61752561,rs2668	1.00E-85	KLK3; KLK15 - KLK3; KLK15 - KLK3; KLK3; KLK15 - KLK3; KLK2; KLK2	Prostate-specific antigen levels	GCST004093
rs1122466	5.00E-85	KLK8,KLK7	Kallikrein-8 levels	GCST90248164
rs190686274	1.00E-84	KLK2	Prostate-specific antigen levels	GCST90461907
rs8109338	4.00E-83	SIGLEC9	SIGLEC9 protein levels	GCST90470637
rs142760794	2.00E-81	MIR8074,SIGLEC21P	CD33 on Immature Myeloid-Derived Suppressor Cells	GCST90001955
rs62113212	4.00E-81	KLK3	Cancer of prostate (PheCode 185)	GCST90475591
rs56031098	1.00E-80	KLK6,KLK5	KLK7 protein levels	GCST90469706
rs2569459	1.00E-79	KLK13,KLK12	Serum levels of protein KLK13	GCST90086572
rs2691254	8.00E-79	CTU1	Blood protein levels	GCST006585
rs3760719	5.00E-78	KLK15,KLK3	KLK4 protein levels	GCST90469704
rs12459419	2.00E-77	CD33	Protein quantitative trait loci	GCST010900
rs76921020	6.00E-76	SIGLEC7	CD33 protein levels	GCST90468625
rs62115757	7.00E-76	KLK10	Blood protein levels in cardiovascular risk	GCST009731
rs273688	2.00E-74	SIGLEC9	T-cell surface glycoprotein CD5 level in Chronic kidney disease with hypertension and no diabetes (5596_75)	GCST90237975
rs67621164	1.00E-73	SIGLEC9	SIGLEC7 protein levels	GCST90470635
rs2691273	3.00E-73	CTU1,KLK14	Sialic acid-binding Ig-like lectin 9 levels	GCST90101552
rs198965	1.00E-72	KLK4	Prostate-specific antigen levels	GCST90461907
rs112736390	3.00E-71	KLK7	Kallikrein-7 levels	GCST90137528
rs76975163	5.00E-70	KLK13,KLK12	KLK12 protein levels	GCST90469698
rs189223569	1.00E-69	SIGLEC24P	CD33 protein levels	GCST90468625
rs183578707	3.00E-69	KLK5	KLK8 protein levels	GCST90469707
rs2569495	1.00E-68	CTU1,KLK14	KLK12 protein levels	GCST90469698
rs183185223	4.00E-67	SIGLECL1	CD33 protein levels	GCST90468625
rs2739419	1.00E-66	KLK7	Kallikrein-7 levels (KLK7.3378.49.2)	GCST90241679
rs12985029	2.00E-66	SIGLEC22P,MIR8074	Myeloid cell surface antigen CD33 levels	GCST90101334
rs201074739	7.00E-65	CD33	CD33 on CD66b++ myeloid cell	GCST90001951
rs147992404	2.00E-64	KLK6	KLK6 protein levels	GCST90469705
rs77067043	2.00E-64	SIGLEC7,SIGLEC9	SIGLEC7 protein levels	GCST90470635
rs76765083	2.00E-62	KLK3	Prostate cancer	GCST011049
rs142611321	2.00E-62	CD33	CD33 protein levels	GCST90468625
rs7259451	3.00E-62	KLK10	KLK14 protein levels	GCST90469700
rs34542531	3.00E-61	KLK8	Kallikrein-8 levels	GCST90161502
rs118185380	1.00E-60	ETFB	CD33 protein levels	GCST90468625
rs181266376	3.00E-60	SIGLEC22P,CD33	CD33 protein levels	GCST90468625
rs2547319	1.00E-59	LIM2,NKG7	CD33 protein levels	GCST90468625
rs60850416	2.00E-59	KLK6	KLK10 protein levels	GCST90469696
rs17632542	8.00E-59	KLK3	Cancer of prostate (PheCode 185)	GCST90479803
rs7245846	1.00E-58	CD33	CD33 on Granulocytic Myeloid-Derived Suppressor Cells	GCST90001950
rs116953977	5.00E-58	SIGLECL1,CD33	Myeloid cell surface antigen CD33 levels	GCST90248431
rs74705037	6.00E-58	KLK8	Kallikrein-8 levels (KLK8.13708.56.3)	GCST90241680
rs143108505	7.00E-58	IGLON5	CD33 protein levels	GCST90468625
rs2735837	7.00E-58	KLK2	Prostate-specific antigen levels	GCST90301330
rs117774219	8.00E-58	KLKP1,KLK2	KLK4 protein levels	GCST90469704
rs62113212	8.00E-58	KLK3	Prostate cancer	GCST90475185
rs57392237	2.00E-57	KLK6,KLK7	Serum levels of protein KLK10	GCST90089306
rs149842697	7.00E-57	CD33	CD33 protein levels	GCST90468625
rs2569523	7.00E-57	PPIAP59,KLK5	KLK8 protein levels	GCST90469707
rs1048328	7.00E-56	KLK11	Protein S100-P levels	GCST90427997
rs11665748	3.00E-54	KLK15,KLK3	Prostate-specific antigen levels (conditioned on lead SNPs)	GCST004094
rs550268035	3.00E-54	CTU1,KLK14	KLK14 protein levels	GCST90469700
rs2569454	4.00E-54	KLK10	Blood protein levels	GCST006585
rs3760739	4.00E-54	KLK12,KLK13	Blood protein levels	GCST006585
rs112237160	8.00E-54	KLK13	Kallikrein-13 levels	GCST90248158
rs273687	2.00E-53	SIGLEC9	Sialic acid-binding Ig-like lectin 9 levels	GCST90161587
rs73051055	3.00E-53	CTU1,KLK14	SIGLEC9 protein levels	GCST90470637
rs2659067	5.00E-53	KLK7	Kallikrein-7 levels	GCST90161750
rs66717916	1.00E-52	CTU1	Sialic acid-binding Ig-like lectin 9 levels	GCST90161587
rs141110039	1.00E-52	CD33	CD33 protein levels	GCST90468625
rs1039405	2.00E-52	SIGLEC18P,SIGLEC9	Blood protein levels	GCST006585
rs2659091	3.00E-52	KLK5,PPIAP59	Kallikrein-8 (analyte X2834.54) levels	GCST90425492
rs141372791	3.00E-52	MIR8074,SIGLEC21P	CD33 protein levels	GCST90468625
rs546388120	4.00E-52	KLK15,KLK3	KLK4 protein levels	GCST90469704
rs45623731	7.00E-52	KLK4	KLK4 protein levels	GCST90469704
rs3745535	8.00E-52	KLK10	CDSN/KLK8 protein level ratio	GCST90313996
rs273640	1.00E-50	SIGLEC22P,CD33	CD45 on Immature Myeloid-Derived Suppressor Cells	GCST90002052
rs1122466	2.00E-50	KLK8,KLK7	kallikrein-11 levels	GCST90012012
rs113836077	2.00E-50	KLK3,KLK15	KLK4 protein levels	GCST90469704
rs56915529	2.00E-50	SIGLEC9	KLK10 protein levels	GCST90469696
rs62113212	2.00E-50	KLK3	Prostate cancer	GCST90479536
rs8103941	5.00E-50	KLK12,KLK13	Kallikrein-12 levels	GCST90101221
rs1722546	8.00E-50	KLK7,KLK8	Kallikrein-8 (analyte X13708.56) levels	GCST90422316
rs10407079	5.00E-49	KLK13,KLK12	KLK12 protein levels	GCST90469698
rs34089525	2.00E-48	KLK13	Kallikrein-13 (analyte X11152.46) levels	GCST90421334
rs114591592	2.00E-48	SIGLEC7,SIGLEC17P	Sialic acid-binding Ig-like lectin 7 levels	GCST90137744
rs12459419	4.00E-48	CD33	white blood cell count (WBC, mean, inv-norm transformed)	GCST90476454
rs2735839	3.00E-47	KLK3,KLK2	Prostate cancer	GCST006085
rs140350753	6.00E-47	KLK4	KLK4 protein levels	GCST90469704
rs190813930	1.00E-46	SIGLEC24P	CD33 protein levels	GCST90468625
rs17632542	3.00E-46	KLK3	Serum prostate-specific antigen levels	GCST000919
rs17715394	9.00E-46	KLK14,CTU1	Sialic acid-binding Ig-like lectin 9 levels	GCST90161587
rs1275221	1.00E-45	KLK7	Kallikrein-7 levels (KLK7.3378.49.2)	GCST90241679
rs35945487	1.00E-45	KLK4	KLK4 protein levels	GCST90469704
rs112103380	1.00E-45	KLK2	Prostate-specific antigen levels	GCST90461907
rs61752561,rs2668	2.00E-45	KLK3; KLK15 - KLK3; KLK15 - KLK3; KLK3; KLK15 - KLK3; KLK2; KLK2	Prostate-specific antigen levels	GCST004093
rs112561158	3.00E-45	KLK4,PPIAP59	KLK4 protein levels	GCST90469704
rs17658926	8.00E-45	KLK14	Kallikrein-14 levels	GCST90248159
rs1275221	2.00E-44	KLK7	Serum levels of protein KLK7	GCST90088351
rs2673908	4.00E-44	SIGLEC18P,SIGLEC9	Cerebrospinal fluid biomarker levels	GCST004000
rs12459419	3.00E-43	CD33	White blood cell count	GCST90002374
rs201074739	1.00E-42	CD33	CD33 on Granulocytic Myeloid-Derived Suppressor Cells	GCST90001950
rs117702791	1.00E-42	LINC01872	SIGLEC10 protein levels	GCST90470630
rs12459419	4.00E-42	CD33	White blood cell count	GCST90002378
rs75950648	1.00E-41	KLKP1	KLK4 protein levels	GCST90469704
rs2411329	3.00E-41	CD33,SIGLEC22P	SIGLEC9 protein levels	GCST90470637
rs3865444	1.00E-40	SIGLEC22P,CD33	Basophil %CD33dim HLA DR- CD66b-	GCST90001533
rs113920094	3.00E-40	KLK15,KLK3	Prostate-specific antigen levels	GCST90461907
rs1710369	4.00E-40	SIGLECL1	Myeloid cell surface antigen CD33 levels	GCST90161642
rs149957772	4.00E-40	KLKP1	Prostate-specific antigen levels	GCST90461907

rs2569459	3.00E-39	KLK13,KLK12	Serum levels of protein KLK13	GCST90088265
rs76334147	3.00E-39	SIGLEC18P,CTU1	Sialic acid-binding Ig-like lectin 9 levels	GCST90161587
rs151335303	3.00E-39	SIGLEC21P,SIGLEC20P	CD33 protein levels	GCST90468625
rs140023174	3.00E-39	ETFB	SIGLEC10 protein levels	GCST90470630
rs201074739	4.00E-39	CD33	Myeloid cell surface antigen CD33 levels (CD33.3166.92.1)	GCST90241989
rs138585047	6.00E-39	CD33	CD33 protein levels	GCST90468625
rs144092860	2.00E-38	SIGLEC20P	CD33 protein levels	GCST90468625
rs113529853	5.00E-38	KLK15	KLK15 protein levels	GCST90469701
rs73050317	6.00E-38	KLK15,KLK3	KLK15 protein levels	GCST90469701
rs139528466	8.00E-38	SIGLEC9	CD33 protein levels	GCST90468625
rs3865444	1.00E-37	SIGLEC22P,CD33	Platelet count	GCST90002357
rs188268307	1.00E-37	SIGLEC21P,SIGLEC20P	CD33 protein levels	GCST90468625
rs3865444	2.00E-37	SIGLEC22P,CD33	Platelet count	GCST90002361
rs76913418	2.00E-37	KLK14,CTU1	Sialic acid-binding Ig-like lectin 9 levels	GCST90161587
rs10415306	2.00E-37	KLK5	KLK8 protein levels	GCST90469707
rs12461147	3.00E-37	KLK6	KLK12 protein levels	GCST90469698
rs2735839	6.00E-37	KLK3,KLK2	Prostate-specific antigen levels	GCST001796
rs139431213	9.00E-37	PPIAP59,KLK4	KLK4 protein levels	GCST90469704
rs74415796	1.00E-36	LIM2	CD33 protein levels	GCST90468625
rs142828509	1.00E-36	KLK12,KLK13	KLK14 protein levels	GCST90469700
rs140185670	2.00E-36	SIGLEC7	Sialic acid-binding Ig-like lectin 7 levels (SIGLEC7.2742.68.2)	GCST90242811
rs111362352	3.00E-36	KLK2	Prostate cancer	GCST90011808
rs73054374	5.00E-36	KLKP1	KLK15 protein levels	GCST90469701
rs117658654	6.00E-36	SIGLEC17P,SIGLEC7	CD33 protein levels	GCST90468625
rs12459419	1.00E-35	CD33	Platelet crit (UKB data field 30090)	GCST90468096
rs150891637	1.00E-35	KLK2	KLK15 protein levels	GCST90469701
rs35379909	1.00E-35	KLK14,KLK13	KLK12 protein levels	GCST90469698
rs1697573	2.00E-35	CD33,SIGLEC22P	CD33- HLA DR+ Absolute Count	GCST90001523
rs149067793	2.00E-35	GPR32,SMIM47	KLK15 protein levels	GCST90469701
rs2569429	3.00E-35	CTU1	Sialic acid-binding Ig-like lectin 9 levels	GCST90161587
rs74705037	5.00E-35	KLK8	KLK12 protein levels	GCST90469698
rs12459419	5.00E-35	CD33	platelet count (mean, inv-norm transformed)	GCST90476299
rs12459419	5.00E-35	CD33	white blood cell count (WBC, minimum, inv-norm transformed)	GCST90476457
rs3865444	8.00E-35	SIGLEC22P,CD33	Basophil Absolute Count	GCST90001532
rs2691254	1.00E-34	CTU1	Blood protein levels	GCST006585
rs2569735	1.00E-34	KLK2,KLK3	Prostate cancer	GCST90018905
rs2691237	1.00E-34	KLK12,KLK13	KLK10 protein levels	GCST90469696
rs1122466	1.00E-34	KLK8,KLK7	KLK12 protein levels	GCST90469698
rs144363592	1.00E-34	SIGLEC7	SIGLEC7 protein levels	GCST90470635
rs34093024	1.00E-34	KLK14,CTU1	Kallikrein-14 levels	GCST90248159
rs12459419	2.00E-34	CD33	Glycated haemoglobin HbA1c levels (UKB data field 30750)	GCST90468072
rs79082347	2.00E-34	KLK14,CTU1	KLK12 protein levels	GCST90469698
rs3745540	3.00E-34	KLK12	Kallikrein-12 levels (KLK12.3199.54.2)	GCST90241671
rs146775340	3.00E-34	KLK13,KLK12	KLK12 protein levels	GCST90469698
rs268883	5.00E-34	KLK7	Kallikrein-7 levels	GCST90248163
rs56349210	8.00E-34	KLK1	KLK1 protein levels	GCST90469702
rs73596517	1.00E-33	KLK14	KLK14 protein levels	GCST90469700
rs3745539	2.00E-33	KLK11	Kallikrein-10 levels	GCST90179343
rs140185670	2.00E-33	SIGLEC7	Sialic acid-binding Ig-like lectin 7 levels	GCST90179421
rs33978622	4.00E-33	CD33	CD33dim HLA DR- Absolute Count	GCST90001531
rs111362352	5.00E-33	KLK2	Cancer (pleiotropy)	GCST90011822
rs2232534	6.00E-33	KLK5	KLK12 protein levels	GCST90469698
rs1624358	7.00E-33	KLK7	Serum levels of protein KLK7	GCST90088351
rs1722547	7.00E-33	KLK8,KLK7	KLK7 protein levels	GCST90469706
rs12459419	8.00E-33	CD33	Lymphocyte count	GCST90002316
rs7249762	8.00E-33	KLK12,KLK13	SIGLEC9 protein levels	GCST90470637
rs12459419	1.00E-32	CD33	White blood cell count	GCST007070
rs2455069	1.00E-32	CD33	Immature Myeloid-Derived Suppressor Cells Absolute Count	GCST90001515
rs2691247	1.00E-32	KLK14,KLK13	Serum levels of protein KLK13	GCST90086572
rs112091145	1.00E-32	TMEM277P	Prostate-specific antigen levels	GCST90461907
rs151144652	2.00E-32	IGLON5	CD33 protein levels	GCST90468625
rs77792593	3.00E-32	KLK2	Prostate-specific antigen levels	GCST90461907
rs1568003	4.00E-32	SIGLEC9	Sialic acid-binding Ig-like lectin 9 levels	GCST90101552
rs35413640	4.00E-32	SIGLEC20P	Sialic acid-binding Ig-like lectin 7 levels	GCST90161452
rs3760739	4.00E-32	KLK12,KLK13	Kallikrein-13 levels	GCST90161668
rs189759947	7.00E-32	LIM2	CD33 protein levels	GCST90468625
rs116993226	8.00E-32	SIGLEC9	SIGLEC9 protein levels	GCST90470637
rs198978	8.00E-31	KLK2	Prostate-specific antigen levels	GCST90301330
rs34542531	1.00E-30	KLK8	Kallikrein-8 levels (KLK8.13708.56.3)	GCST90241680
rs148974810	1.00E-30	KLK10,KLK9	Kallikrein-8 levels	GCST90137649
rs118089616	1.00E-30	KLK14,CTU1	KLK14 protein levels	GCST90469700
rs2569491	2.00E-30	KLK14	ELN protein levels	GCST90469103
rs145855656	2.00E-30	GPR32P1,CLEC11A	KLK15 protein levels	GCST90469701
rs71333926	2.00E-30	KLK13,KLK12	SIGLEC9 protein levels	GCST90470637
rs12459419	4.00E-30	CD33	Lymphocyte count	GCST90002320
rs12459419	6.00E-30	CD33	Platelet count (UKB data field 30080)	GCST90468095
rs148022792	6.00E-30	KLK7	Kallikrein-7 levels	GCST90248163
rs12459419	7.00E-30	CD33	White blood cell count	GCST90002407
rs3826656	9.00E-30	CD33,SIGLEC22P	CD33 on Immature Myeloid-Derived Suppressor Cells	GCST90001955
rs145396720	1.00E-29	KLK14,KLK13	KLK12 protein levels	GCST90469698
rs146242395	1.00E-29	KLK13,KLK12	KLK12 protein levels	GCST90469698
rs148022792	2.00E-29	KLK7	Kallikrein-7 levels	GCST90161750
rs57392237	2.00E-29	KLK6,KLK7	KLK4 protein levels	GCST90469704
rs2659051	3.00E-29	KLK15,KLK3	Prostate cancer	GCST011049
rs3810091	3.00E-29	KLK14,KLK13	Kallikrein-14 (analyte X8620.56) levels	GCST90427428
rs76765083	3.00E-29	KLK3	Prostate cancer	GCST90428117
rs10409348	4.00E-29	CD33	CD33 on CD33dim HLA DR+ CD11b+	GCST90001948
rs3745540	8.00E-29	KLK12	Protein levels in obesity	GCST010196
rs143406762	9.00E-29	KLK11	KLK7 protein levels	GCST90469706
rs74705037	1.00E-28	KLK8	Kallikrein-8 levels (KLK8.13708.56.3)	GCST90241680
rs62113212	1.00E-28	KLK3	KLK3 protein levels	GCST90469703
rs17632542	2.00E-28	KLK3	Prostate cancer	GCST002421
rs1354106	2.00E-28	CD33	Monocytic Myeloid-Derived Suppressor Cells Absolute Count	GCST90001530
rs192688056	2.00E-28	KLK13	KLK8 protein levels	GCST90469707
rs3826656	3.00E-28	CD33,SIGLEC22P	CD33 on basophil	GCST90001954
rs12983058	3.00E-28	SIGLEC9	Sialic acid-binding Ig-like lectin 7 levels (SIGLEC7.2742.68.2)	GCST90242811
rs198968	5.00E-28	KLK4	KLK15 protein levels	GCST90469701
rs567511285	6.00E-28	CTU1,KLK14	KLK14 protein levels	GCST90469700
rs268904	7.00E-28	KLK5	KLK8 protein levels	GCST90469707
rs141544900	1.00E-27	SIGLEC7	SIGLEC9 protein levels	GCST90470637
rs79449301	1.00E-27	KLK2	Prostate-specific antigen levels	GCST90461907
rs117278328	2.00E-27	SIGLECL1	CD33 protein levels	GCST90468625
rs12459419	2.00E-27	CD33	white blood cell count (WBC, maximum, inv-norm transformed)	GCST90476451
rs3760744	3.00E-27	KLK13,KLK12	Blood protein levels	GCST006585
rs3810091	3.00E-27	KLK14,KLK13	KLK13 protein levels	GCST90469699

rs112156686	5.00E-27	KLK13,KLK12	KLK12 protein levels	GCST90469698
rs10426	8.00E-27	KLK10	Serum 25-Hydroxyvitamin D levels (conditioned on BMI)	GCST90000616
rs3865444	1.00E-26	SIGLEC22P,CD33	Plateletcrit	GCST90002400
rs200590126	1.00E-26	VSIG10L	VSIG10L protein levels	GCST90471049
rs2691227	2.00E-26	KLK13,KLK12	Kallikrein-12 levels	GCST90161667
rs10426	3.00E-26	KLK10	Serum 25-Hydroxyvitamin D levels	GCST010144
rs147740402	3.00E-26	NKG7,CLDND2	CD33 protein levels	GCST90468625
rs3865444	3.00E-26	SIGLEC22P,CD33	white blood cell count (WBC, mean, inv-norm transformed)	GCST90480724
rs150618116	4.00E-26	ETFB	CD33 protein levels	GCST90468625
rs3865444	6.00E-26	SIGLEC22P,CD33	Myeloid cell surface antigen CD33 level in Chronic kidney disease with hypertension and no diabetes (3166_92)	GCST90237245
rs12983058	7.00E-26	SIGLEC9	Sialic acid-binding Ig-like lectin 7 levels	GCST90179421
rs143810424	1.00E-25	GPR32,GPR32P1	KLK15 protein levels	GCST90469701
rs61752561	2.00E-25	KLK3	Prostate-specific antigen levels (conditioned on lead SNPs)	GCST004094
rs149671592	2.00E-25	IGLON5	CD33 protein levels	GCST90468625
rs138813375	2.00E-25	KLK2	KLK4 protein levels	GCST90469704
rs2691262	2.00E-25	CTU1	KLK13 protein levels	GCST90469699
rs10409348	3.00E-25	CD33	CD33 on CD33dim HLA DR+ CD11b-	GCST90001949
rs17632542	3.00E-25	KLK3	Prostate cancer	GCST90027060
rs2455070	3.00E-25	LINC01872	SIGLEC7 protein levels	GCST90470635
rs577568020	3.00E-25	KLK2	Prostate-specific antigen levels	GCST90461907
rs10426	5.00E-25	KLK10	Serum 25-Hydroxyvitamin D levels	GCST90000618
rs3865444	1.00E-24	SIGLEC22P,CD33	Lymphocyte count	GCST90002388
rs116016879	1.00E-24	SIGLEC17P	Sialic acid-binding Ig-like lectin 7 level in Chronic kidney disease with hypertension and no diabetes (2742_68)	GCST90237070
rs150601072	1.00E-24	SIGLEC9	Sialic acid-binding Ig-like lectin 9 levels	GCST90161587
rs144406548	1.00E-24	LINC01872	Myeloid cell surface antigen CD33 levels	GCST90161642
rs12975233	1.00E-24	LINC01872	Myeloid cell surface antigen CD33 levels	GCST90161642
rs12459419	1.00E-24	CD33	platelet count (maximum, inv-norm transformed)	GCST90476296
rs756155792	2.00E-24	SIGLEC17P,SIGLEC20P	CD33 protein levels	GCST90468625
rs1058205	2.00E-24	KLK3	KLK1 protein levels	GCST90469702
rs77342236	2.00E-24	KLK12,KLK13	KLK13 protein levels	GCST90469699
rs181163991	2.00E-24	KLK9	PSAPL1 protein levels	GCST90470350
rs117229324	2.00E-24	KLK14	Kallikrein-14 levels	GCST90248159
rs3826656	3.00E-24	CD33,SIGLEC22P	CD33 on CD33dim HLA DR-	GCST90001953
rs117296079	3.00E-24	KLK13,KLK14	KLK14 protein levels	GCST90469700
rs10426	3.00E-24	KLK10	25-hydroxyvitamin D levels (skin colour stratified)	GCST90448682
rs1722558	4.00E-24	KLK7	Blood protein levels	GCST006585
rs3865444	5.00E-24	SIGLEC22P,CD33	Lymphocyte count (UKB data field 30120)	GCST90468082
rs2569491	6.00E-24	KLK14	LECT2 protein levels	GCST90469751
rs111946867	9.00E-24	SIGLECL1	CD33 protein levels	GCST90468625
rs12459419	1.00E-23	CD33	Hemoglobin A1c levels	GCST90018958
rs147441988	1.00E-23	KLK14,CTU1	CD33 protein levels	GCST90468625
rs3865444	2.00E-23	SIGLEC22P,CD33	Platelet count	GCST90002402
rs140254627	4.00E-23	KLK6	KLK7 protein levels	GCST90469706
rs3865444	4.00E-23	SIGLEC22P,CD33	red cell diameter width (RDW, mean, inv-norm transformed)	GCST90476361
rs10424744	6.00E-23	KLK10	kallikrein-11 levels	GCST90161500
rs2459143	6.00E-23	SIGLEC9	SIGLEC9 protein levels	GCST90470637
rs3865444	6.00E-23	SIGLEC22P,CD33	white blood cell count (WBC, minimum, inv-norm transformed)	GCST90480725
rs3760737	7.00E-23	KLK11,KLK10	KLK11 protein levels	GCST90469697
rs10410942	9.00E-23	KLK8	Serum levels of protein KLK8	GCST90088093
rs11084060	9.00E-23	CD33,SIGLEC22P	Myeloid cell surface antigen CD33 levels	GCST90161642
rs3793436	9.00E-23	SIGLEC7	KLK13 protein levels	GCST90469699
rs147530298	1.00E-22	CTU1,SIGLEC18P	KLK14 protein levels	GCST90469700
rs137991548	2.00E-22	CTU1,SIGLEC18P	KLK12 protein levels	GCST90469698
rs62117666	3.00E-22	KLK13,KLK12	Kallikrein-12 levels	GCST90161667
rs184997153	4.00E-22	KLK14	KLK14 protein levels	GCST90469700
rs268890	4.00E-22	KLK7,KLK6	Erythematosquamous dermatosis (PheCode 690)	GCST90480446
rs201009355	5.00E-22	KLK14	CD33 protein levels	GCST90468625
rs1054713	5.00E-22	KLK1	Prostate-specific antigen levels	GCST90301330
rs73053016	7.00E-22	SIGLEC18P,CTU1	Sialic acid-binding Ig-like lectin 9 levels	GCST90161587
rs111504285	8.00E-22	PPIAP59,KLK5	KLK10 protein levels	GCST90469696
rs2735839	1.00E-21	KLK3,KLK2	Prostate cancer	GCST011957
rs566774530	1.00E-21	SIGLECL1	CD33 protein levels	GCST90468625
rs117206276	1.00E-21	IGLON5	CD33 protein levels	GCST90468625
rs538022630	1.00E-21	KLK4,PPIAP59	KLK4 protein levels	GCST90469704
rs59352575	1.00E-21	KLK2	Prostate-specific antigen levels	GCST90461907
rs268890	1.00E-21	KLK7,KLK6	Seborrheic dermatitis (PheCode 690.1)	GCST90480445
rs2673908	2.00E-21	SIGLEC18P,SIGLEC9	Monocyte percentage of white cells	GCST90002394
rs10409348	2.00E-21	CD33	CD33 on CD14+ monocyte	GCST90001946
rs3865444	2.00E-21	SIGLEC22P,CD33	CD33+ HLA DR+ CD14- Absolute Count	GCST90001518
rs141554252	2.00E-21	KLK6,KLK5	KLK10 protein levels	GCST90469696
rs118001207	2.00E-21	KLK6,KLK7	KLK12 protein levels	GCST90469698
rs142212375	2.00E-21	KLK15	Calculus of kidney (PheCode 594.1)	GCST90476145
rs142212375	2.00E-21	KLK15	Urinary calculus (PheCode 594)	GCST90476143
rs2673908	3.00E-21	SIGLEC18P,SIGLEC9	Neurological blood protein biomarker levels	GCST008478
rs576056416	3.00E-21	KLK14	KLK14 protein levels	GCST90469700
rs17715394	3.00E-21	KLK14,CTU1	Kallikrein-14 levels	GCST90248159
rs3865444	4.00E-21	SIGLEC22P,CD33	CD33+ HLA DR+ Absolute Count	GCST90001517
rs62117661	5.00E-21	KLK11	kallikrein-11 levels	GCST90161500
rs266121	5.00E-21	ACP4,C19orf48P	KLK15 protein levels	GCST90469701
rs3865444	5.00E-21	SIGLEC22P,CD33	red cell diameter width (RDW, minimum, inv-norm transformed)	GCST90476365
rs531711120	6.00E-21	LINC01872	CD33 protein levels	GCST90468625
rs12459419	7.00E-21	CD33	Neutrophil count	GCST90002351
rs186312026	7.00E-21	KLK4,KLKP1	Prostate-specific antigen levels	GCST90461907
rs1058205	8.00E-21	KLK3	Prostate-specific antigen levels	GCST002476
rs12459419	8.00E-21	CD33	C-reactive protein levels	GCST90029070
rs2659051	9.00E-21	KLK15,KLK3	Prostate-specific antigen levels	GCST002703
rs12459419	9.00E-21	CD33	White blood cell count	GCST90018978
rs12983058	1.00E-20	SIGLEC9	Cerebrospinal fluid biomarker levels	GCST004000
rs3865444	1.00E-20	SIGLEC22P,CD33	Platelet count	GCST90018969
rs2569459	1.00E-20	KLK13,KLK12	Kallikrein-13 levels	GCST90137943
rs561859027	1.00E-20	C19orf48P	KLK1 protein levels	GCST90469702
rs117837287	1.00E-20	KLK4,PPIAP59	KLK4 protein levels	GCST90469704
rs543694487	1.00E-20	LIM2-AS1,C19orf84	SIGLEC10 protein levels	GCST90470630
rs1654527	1.00E-20	KLK7	Kallikrein-10 levels	GCST90179343
rs76765083	2.00E-20	KLK3	Prostate cancer	GCST011048
rs3212852	2.00E-20	KLK15	Prostate-specific antigen levels	GCST90461907
rs66613646	2.00E-20	KLK13,KLK14	Kallikrein-13 levels	GCST90248158
rs112177318	3.00E-20	SIGLECL1	Myeloid cell surface antigen CD33 levels	GCST90161642
rs2569454	3.00E-20	KLK10	KLK13 protein levels	GCST90469699
rs142212375	3.00E-20	KLK15	Calculus of kidney (PheCode 594.1)	GCST90480395
rs2569735	4.00E-20	KLK2,KLK3	Prostate cancer	GCST90651052
rs12983058	4.00E-20	SIGLEC9	Sialic acid-binding Ig-like lectin 7 levels	GCST90161452
rs11084043	4.00E-20	KLK7,KLK6	Kallikrein-7 levels	GCST90161750
rs35889412	4.00E-20	SMIM47	KLK1 protein levels	GCST90469702
rs74761463	4.00E-20	KLK2	KLK15 protein levels	GCST90469701

rs12459419	4.00E-20	CD33	platelet count (minimum, inv-norm transformed)	GCST90476302
rs11665748	5.00E-20	KLK15,KLK3	Prostate cancer	GCST90274716
rs1354774	6.00E-20	KLKP1	Prostate-specific antigen levels	GCST001799
rs118001207	6.00E-20	KLK6,KLK7	KLK10 protein levels	GCST90469696
rs3810091	7.00E-20	KLK14,KLK13	Kallikrein-14 (analyte X15544.25) levels	GCST90422724
rs186620214	7.00E-20	SIGLEC9	Sialic acid-binding Ig-like lectin 7 levels	GCST90161452
rs2735839	8.00E-20	KLK3,KLK2	Elevated serum prostate-specific antigen levels in healthy men	GCST004345
rs2691270	9.00E-20	KLK14,CTU1	SIGLEC7 protein levels	GCST90470635
rs142212375	9.00E-20	KLK15	Urinary calculus (PheCode 594)	GCST90480398
rs189680912	1.00E-19	ETFB	CD33 protein levels	GCST90468625
rs12459419	1.00E-19	CD33	C-reactive protein levels (MTAG)	GCST90179146
rs67621164	1.00E-19	SIGLEC9	Sialic acid-binding Ig-like lectin 7 levels	GCST90249551
rs62113214	3.00E-19	KLK3	Prostate cancer	GCST90651052
rs268890	3.00E-19	KLK7,KLK6	Seborrheic dermatitis (PheCode 690.1)	GCST90476171
rs12459419	4.00E-19	CD33	Neutrophil count	GCST90002355
rs145323276	4.00E-19	SIGLECL1	SIGLEC9 protein levels	GCST90470637
rs117268623	4.00E-19	KLK11	kallikrein-11 levels	GCST90274899
rs268890	4.00E-19	KLK7,KLK6	Erythematosquamous dermatosis (PheCode 690)	GCST90476170
rs117268623	5.00E-19	KLK11	KLK11 protein levels	GCST90277891
rs2075803	8.00E-19	SIGLEC9	Monocyte percentage (UKB data field 30190)	GCST90468091
rs3865444	8.00E-19	SIGLEC22P,CD33	Monocyte count (UKB data field 30130)	GCST90468090
rs10409348	8.00E-19	CD33	CD33 on CD33+ HLA DR+ CD14-	GCST90001957
rs2673908	8.00E-19	SIGLEC18P,SIGLEC9	Sialic acid-binding Ig-like lectin 7 levels	GCST90161452
rs545076978	1.00E-18	PPIAP59,KLK5	KLK4 protein levels	GCST90469704
rs2075803	1.00E-18	SIGLEC9	T-cell surface glycoprotein CD5 levels	GCST90249781
rs2735839	2.00E-18	KLK3,KLK2	Prostate cancer	GCST000152
rs1710354	2.00E-18	SIGLEC24P	Myeloid cell surface antigen CD33 levels	GCST90101334
rs2569491	2.00E-18	KLK14	CCL27 protein levels	GCST90468579
rs144363592	2.00E-18	SIGLEC7	Sialic acid-binding Ig-like lectin 7 levels	GCST90161452
rs547578487	2.00E-18	SIGLEC24P,IGLON5	CD33 protein levels	GCST90468625
rs146690810	2.00E-18	IGLON5,SIGLEC24P	CD33 protein levels	GCST90468625
rs191123336	2.00E-18	KLK4,PPIAP59	KLK15 protein levels	GCST90469701
rs578220231	2.00E-18	KLK13,KLK14	KLK12 protein levels	GCST90469698
rs113788004	2.00E-18	SIGLEC9	SIGLEC9 protein levels	GCST90470637
rs112103380	2.00E-18	KLK2	Prostate-specific antigen levels	GCST90301330
rs10635364	2.00E-18	KLK12	Kallikrein-12 levels	GCST90248157
rs33978622	2.00E-18	CD33	platelet count (mean, inv-norm transformed)	GCST90480651
rs11667946	3.00E-18	KLK7,KLK6	Blood protein levels in cardiovascular risk	GCST009731
rs10421385	3.00E-18	SIGLEC20P,SIGLEC21P	Myeloid cell surface antigen CD33 levels	GCST90101334
rs117268623	3.00E-18	KLK11	kallikrein-11 levels	GCST90161500
rs146181856	3.00E-18	CD33	CD33 protein levels	GCST90468625
rs117285162	3.00E-18	KLK3,KLK15	KLK15 protein levels	GCST90469701
rs3865444	4.00E-18	SIGLEC22P,CD33	C-reactive protein levels	GCST009777
rs2569488	4.00E-18	KLK14,KLK13	KLK14 protein levels	GCST90277889
rs573547411	4.00E-18	SMIM47	KLK4 protein levels	GCST90469704
rs12461866	4.00E-18	SIGLEC20P,SIGLEC21P	SIGLEC7 protein levels	GCST90470635
rs118147512	4.00E-18	SIGLEC9	SIGLEC9 protein levels	GCST90470637
rs62117662	4.00E-18	KLK11	kallikrein-11 levels	GCST90248156
rs3865444	4.00E-18	SIGLEC22P,CD33	Hemoglobin A1c (HbA1c, minimum, inv-norm transformed)	GCST90475097
rs117268623	5.00E-18	KLK11	kallikrein-11 levels	GCST009567
rs80237306	6.00E-18	SIGLEC20P	Myeloid cell surface antigen CD33 levels	GCST90161642
rs139528466	6.00E-18	SIGLEC9	SIGLEC9 protein levels	GCST90470637
rs11668576	6.00E-18	VSIG10L	SIGLEC8 protein levels	GCST90470636
rs10409348	7.00E-18	CD33	CD33 on CD33+ HLA DR+	GCST90001956
rs117110414	8.00E-18	SIGLEC17P	CD33 protein levels	GCST90468625
rs12610961	8.00E-18	ETFB	SIGLEC8 protein levels	GCST90470636
rs1474520	1.00E-17	KLK11	KLK13 protein levels	GCST90469699
rs12983994	1.00E-17	KLK2	Prostate-specific antigen levels	GCST90301330
rs33978622	1.00E-17	CD33	white blood cell count (WBC, maximum, inv-norm transformed)	GCST90480723
rs10410942	2.00E-17	KLK8	Blood protein levels	GCST006585
rs3865444	2.00E-17	SIGLEC22P,CD33	C-reactive protein levels (UKB data field 30710)	GCST90468064
rs34912427	3.00E-17	CD33	CD45 on CD33- HLA DR+	GCST90002046
rs12983058	3.00E-17	SIGLEC9	AGR3 protein levels	GCST90468254
rs117268623	3.00E-17	KLK11	GPR15L protein levels	GCST90469396
rs144178553	3.00E-17	SIGLEC9	SIGLEC9 protein levels	GCST90470637
rs266849	4.00E-17	KLK3,KLK15	Prostate-specific antigen levels (conditioned on lead SNPs)	GCST004094
rs138894449	5.00E-17	SIGLEC20P,SIGLEC17P	KLK12 protein levels	GCST90469698
rs187388451	6.00E-17	CD33	CD33 protein levels	GCST90468625
rs571270254	6.00E-17	KLK13,KLK12	KLK12 protein levels	GCST90469698
rs151253920	6.00E-17	SIGLEC17P,SIGLEC7	SIGLEC9 protein levels	GCST90470637
rs113697264	6.00E-17	KLK15,KLK3	Prostate-specific antigen levels	GCST90461907
rs12459419	6.00E-17	CD33	lymphocyte (absolute count, maximum, inv-norm transformed)	GCST90475423
rs61752561,rs2668	8.00E-17	KLK3; KLK15 - KLK3; KLK15 - KLK3; KLK3; KLK15 - KLK3; KLK2; KLK2	Prostate-specific antigen levels	GCST004093
rs10425377	9.00E-17	KLK10	Protein quantitative trait loci	GCST010900
rs140989696	9.00E-17	KLK8,KLK7	KLK10 protein levels	GCST90469696
rs186627912	9.00E-17	KLK14	KLK12 protein levels	GCST90469698
rs3865444	1.00E-16	SIGLEC22P,CD33	CD33+ HLA DR+ CD14dim Absolute Count	GCST90001520
rs2569491	1.00E-16	KLK14	Serum levels of protein DKK2	GCST90089488
rs201452734	1.00E-16	VSIG10L	VSIG10L protein levels	GCST90471049
rs266851	1.00E-16	KLK15	KLK1 protein levels	GCST90469702
rs76673459	1.00E-16	KLK14,CTU1	KLK13 protein levels	GCST90469699
rs10424797	1.00E-16	IGLON5	KLK14 protein levels	GCST90469700
rs137953543	2.00E-16	SIGLEC17P,SIGLEC7	Serum levels of protein SIGLEC7	GCST90088042
rs563123318	2.00E-16	SIGLEC24P	CD33 protein levels	GCST90468625
rs73066858	2.00E-16	LIM2-AS1,NIFKP6	CD33 protein levels	GCST90468625
rs145396720	2.00E-16	KLK14,KLK13	KLK13 protein levels	GCST90469699
rs3865444	2.00E-16	SIGLEC22P,CD33	mean corpuscular hemoglobin concentration (MCHC, mean, inv-norm transformed)	GCST90475458
rs193165893	3.00E-16	SIGLEC17P,SIGLEC7	CD33 protein levels	GCST90468625
rs78347527	3.00E-16	CD33	Myeloid cell surface antigen CD33 levels	GCST90161642
rs75773078	3.00E-16	CD33	SIGLEC5 protein levels	GCST90470633
rs11553092	3.00E-16	KLK5	Kallikrein 5 plasma levels	GCST90085747
rs16982527	3.00E-16	SIGLEC20P,SIGLEC17P	SIGLEC7 protein levels	GCST90470635
rs145445360	3.00E-16	SIGLEC18P,CTU1	SIGLEC9 protein levels	GCST90470637
rs6070	4.00E-16	KLK2	Prostate-specific antigen levels (conditioned on lead SNPs)	GCST004094
rs56035820	4.00E-16	KLK10	CDSN protein levels	GCST90468688
rs2569451	5.00E-16	KLK10	kallikrein-11 levels	GCST90161500
rs181821157	5.00E-16	LIM2,NKG7	CD33 protein levels	GCST90468625
rs66544068	6.00E-16	SIGLEC9	Sialic acid-binding Ig-like lectin 9 levels	GCST90161587
rs184997153	7.00E-16	KLK14	KLK12 protein levels	GCST90469698
rs1124952,rs66787	7.00E-16	BCAS4 x VN1R92P - ZNF725P	Total PHF-tau (SNP x SNP interaction)	GCST010340
rs3865444	8.00E-16	SIGLEC22P,CD33	Lymphocyte count	GCST90018962
rs180684409	9.00E-16	IGLON5	CD33 protein levels	GCST90468625
rs149671592	9.00E-16	IGLON5	Myeloid cell surface antigen CD33 levels	GCST90161642
rs77485145	9.00E-16	KLK13,KLK12	KLK10 protein levels	GCST90469696
rs2659067	1.00E-15	KLK7	Kallikrein-7 levels	GCST90101227

rs192688056	1.00E-15	KLK13	CD33 protein levels	GCST90468625
rs186627912	1.00E-15	KLK14	KLK14 protein levels	GCST90469700
rs76195063	2.00E-15	SIGLEC9	Sialic acid-binding Ig-like lectin 9 levels	GCST90161587
rs79324425	2.00E-15	KLK6	Kallikrein-7 levels (KLK7.3378.49.2)	GCST90241679
rs73591965	2.00E-15	KLK11	Kallikrein-12 levels	GCST90161667
rs192662882	2.00E-15	KLK13,KLK12	KLK12 protein levels	GCST90469698
rs2569446	2.00E-15	SIGLEC9	SIGLEC9 protein levels	GCST90470637
rs33978622	2.00E-15	CD33	monocyte (absolute count, maximum, inv-norm transformed)	GCST90475499
rs2232534	2.00E-15	KLK5	Prostate-specific antigen levels	GCST90461907
rs7245846	3.00E-15	CD33	CD33+ HLA DR+ CD14- %CD33+ HLA DR+	GCST90001519
rs12459419	3.00E-15	CD33	Neutrophill count (UKB data field 30140)	GCST90468092
rs141548226	3.00E-15	KLK14,CTU1	SIGLEC9 protein levels	GCST90470637
rs61752561	3.00E-15	KLK3	Prostate cancer	GCST90274714
rs12459419	3.00E-15	CD33	neutrophil (absolute count, mean, inv-norm transformed)	GCST90475529
rs139204023	4.00E-15	VSIG10L	SIGLEC8 protein levels	GCST90470636
rs148108087	4.00E-15	LINC01872	Body mass index	GCST90255621
rs10419464	5.00E-15	IGLON5	Myeloid cell surface antigen CD33 levels	GCST90161642
rs191123336	5.00E-15	KLK4,PPIAP59	KLK4 protein levels	GCST90469704
rs10409348	6.00E-15	CD33	CD33 on CD33+ HLA DR+ CD14dim	GCST90001947
rs62113214	6.00E-15	KLK3	KLK4 protein levels	GCST90469704
rs9304708	7.00E-15	KLK14	RSPO1 protein levels	GCST90470503
rs77067043	7.00E-15	SIGLEC7,SIGLEC9	Sialic acid-binding Ig-like lectin 7 levels	GCST90179421
rs2569735	8.00E-15	KLK2,KLK3	Prostate cancer	GCST90503487
rs3865444	9.00E-15	SIGLEC22P,CD33	Monocyte count	GCST90002393
rs3865444	9.00E-15	SIGLEC22P,CD33	red cell diameter width (RDW, mean, inv-norm transformed)	GCST90480672
rs266868	1.00E-14	KLK3,KLK15	Prostate-specific antigen levels (conditioned on lead SNPs)	GCST004094
rs36120499	1.00E-14	KLK14,CTU1	Kallikrein-14 levels (KLK14.8620.56.3)	GCST90241672
rs2569475	1.00E-14	KLK13	KLK11 protein levels	GCST90469697
rs11670134	1.00E-14	KLK4,PPIAP59	KLK12 protein levels	GCST90469698
rs80253708	1.00E-14	KLK9	KLK12 protein levels	GCST90469698
rs34967380	1.00E-14	SIGLEC20P,SIGLEC21P	SIGLEC7 protein levels	GCST90470635
rs34912427	1.00E-14	CD33	SIGLEC6 protein levels	GCST90470634
rs112658494	1.00E-14	KLK14	SIGLEC9 protein levels	GCST90470637
rs3865444	1.00E-14	SIGLEC22P,CD33	red cell diameter width (RDW, minimum, inv-norm transformed)	GCST90480673
rs3865444	2.00E-14	SIGLEC22P,CD33	Eosinophil counts	GCST007065
rs12459419	2.00E-14	CD33	Basophil count	GCST90002292
rs34912427	2.00E-14	CD33	C-reactive protein	GCST90018950
rs2455069	2.00E-14	CD33	Immature Myeloid-Derived Suppressor Cells %CD33dim HLA DR- CD66b-	GCST90001516
rs273665	2.00E-14	SIGLEC7,SIGLEC17P	Sialic acid-binding Ig-like lectin 7 levels	GCST90161452
rs113294466	2.00E-14	CTU1	CD33 protein levels	GCST90468625
rs61752561	2.00E-14	KLK3	Prostate cancer	GCST90274713
rs77067043	2.00E-14	SIGLEC7,SIGLEC9	Sialic acid-binding Ig-like lectin 7 levels	GCST90249551
rs3865444	2.00E-14	SIGLEC22P,CD33	platelet count (maximum, inv-norm transformed)	GCST90480650
rs3865444	3.00E-14	SIGLEC22P,CD33	Monocyte count	GCST90018967
rs192204021	3.00E-14	KLK7,KLK8	KLK10 protein levels	GCST90469696
rs3865444	4.00E-14	SIGLEC22P,CD33	Total bilirubin levels (UKB data field 30840)	GCST90468104
rs555362730	4.00E-14	CD33	CD33 protein levels	GCST90468625
rs117268623	4.00E-14	KLK11	LY6D protein levels	GCST90469821
rs188809440	5.00E-14	SIGLEC7	CD33 protein levels	GCST90468625
rs11084051	5.00E-14	KLK12,KLK13	KLK11 protein levels	GCST90469697
rs266849	5.00E-14	KLK3,KLK15	Prostate-specific antigen levels	GCST90249127
rs12459419	5.00E-14	CD33	lymphocyte (absolute count, mean, inv-norm transformed)	GCST90475426
rs2739466	5.00E-14	KLK2	Prostatitis (PheCode 601.1)	GCST90480409
rs3760744	6.00E-14	KLK13,KLK12	KLK13 protein levels	GCST90277893
rs10409348	6.00E-14	CD33	CD33 on Monocytic Myeloid-Derived Suppressor Cells	GCST90001952
rs12459419	6.00E-14	CD33	Lymphocyte count	GCST90085815
rs138684768	6.00E-14	KLK4,PPIAP59	KLK8 protein levels	GCST90469707
rs118165899	6.00E-14	SIGLEC17P,SIGLEC7	SIGLEC9 protein levels	GCST90470637
rs142212375	6.00E-14	KLK15	Calculus of ureter (PheCode 594.3)	GCST90480397
rs2569446	7.00E-14	SIGLEC9	KLK14 protein levels	GCST90469700
rs117268623	8.00E-14	KLK11	SLURP1 protein levels	GCST90470674
rs2691244	9.00E-14	KLK14	Sialic acid-binding Ig-like lectin 9 levels	GCST90161587
rs12459419	9.00E-14	CD33	mean corpuscular hemoglobin concentration (MCHC, minimum, inv-norm transformed)	GCST90475462
rs2659124	1.00E-13	KLK3,KLK15	Prostate cancer	GCST002944
rs12459419	1.00E-13	CD33	Basophil count	GCST90002296
rs1058205	1.00E-13	KLK3	Prostate cancer	GCST90503487
rs62113214	1.00E-13	KLK3	Prostate cancer	GCST90503487
rs111774908	1.00E-13	SIGLEC7	SIGLEC9 protein levels	GCST90470637
rs118089616	1.00E-13	KLK14,CTU1	SIGLEC9 protein levels	GCST90470637
rs528134634	2.00E-13	SIGLEC7	CD33 protein levels	GCST90468625
rs547724741	2.00E-13	SIGLEC22P,CD33	CD33 protein levels	GCST90468625
rs144861939	2.00E-13	SIGLEC22P,CD33	Myeloid cell surface antigen CD33 levels	GCST90161642
rs190041860	2.00E-13	PPIAP59,KLK4	KLK4 protein levels	GCST90469704
rs1048328	2.00E-13	KLK11	KLK15 protein levels	GCST90469701
rs2691235	2.00E-13	KLK12,KLK13	KLK8 protein levels	GCST90469707
rs180674410	2.00E-13	C19orf48P	Prostate-specific antigen levels	GCST90461907
rs3826656	3.00E-13	CD33,SIGLEC22P	Cerebrospinal fluid biomarker levels	GCST004000
rs2659051	3.00E-13	KLK15,KLK3	Prostate cancer	GCST011046
rs201074739	3.00E-13	CD33	CD45 on Immature Myeloid-Derived Suppressor Cells	GCST90002052
rs2739437	3.00E-13	KLK11,KLK12	Kallikrein-12 levels	GCST90161667
rs746701618	3.00E-13	KLK4,KLKP1	KLK1 protein levels	GCST90469702
rs145560168	3.00E-13	KLK4	KLK4 protein levels	GCST90469704
rs554122041	3.00E-13	KLK10	KLK10 protein levels	GCST90469696
rs2411329	3.00E-13	CD33,SIGLEC22P	KLK14 protein levels	GCST90469700
rs62113212	3.00E-13	KLK3	Elevated prostate specific antigen [PSA] (PheCode 796)	GCST90476276
rs112749278	4.00E-13	KLK5,PPIAP59	KLK15 protein levels	GCST90469701
rs33978622	4.00E-13	CD33	neutrophil (absolute count, mean, inv-norm transformed)	GCST90479711
rs62113214	5.00E-13	KLK3	Prostate cancer (late onset)	GCST90503489
rs2569735	5.00E-13	KLK2,KLK3	Prostate cancer (late onset)	GCST90503489
rs12459419	5.00E-13	CD33	platelet count (minimum, inv-norm transformed)	GCST90480652
rs111335308	6.00E-13	NKG7,LIM2	Sialic acid-binding Ig-like lectin 7 levels	GCST90161452
rs3865444	7.00E-13	SIGLEC22P,CD33	Reticulocyte fraction of red cells	GCST90002406
rs192872396	7.00E-13	SIGLEC7	CD33 protein levels	GCST90468625
rs12459419	7.00E-13	CD33	lymphocyte (absolute count, maximum, inv-norm transformed)	GCST90479663
rs3865444	8.00E-13	SIGLEC22P,CD33	Eosinophil counts	GCST90002381
rs149079128	8.00E-13	KLK14	KLK14 protein levels	GCST90469700
rs370777932	9.00E-13	ETFB	SIGLEC8 protein levels	GCST90470636
rs17716331	1.00E-12	LIM2,NKG7	Neutrophil count	GCST90002398
rs12459419	1.00E-12	CD33	Neutrophil count	GCST90018968
rs3865444	1.00E-12	SIGLEC22P,CD33	Granulocytic Myeloid-Derived Suppressor Cells Absolute Count	GCST90001524
rs12459419	1.00E-12	CD33	CD45 on Immature Myeloid-Derived Suppressor Cells	GCST90002052
rs545571047	1.00E-12	KLK15	GLIPR1 protein levels	GCST90469357
rs174776	1.00E-12	KLK3	Prostate cancer	GCST90503487
rs11671709	1.00E-12	TMEM277P	Prostate-specific antigen levels	GCST90461907
rs12459419	2.00E-12	CD33	Basophil count	GCST90002379

rs1058205	2.00E-12	KLK3	Prostate cancer	GCST90651067
rs117658654	2.00E-12	SIGLEC17P,SIGLEC7	Myeloid cell surface antigen CD33 levels	GCST90161642
rs2560935	2.00E-12	KLK15	KLK4 protein levels	GCST90469704
rs1058205	2.00E-12	KLK3	Prostate cancer (late onset)	GCST90503489
rs374546878	2.00E-12	KLK3	Prostate-specific antigen levels	GCST90301330
rs56021438	2.00E-12	KLK7	Kallikrein-7 levels	GCST90248163
rs33978622	2.00E-12	CD33	monocyte (absolute count, mean, inv-norm transformed)	GCST90479702
rs564957276	3.00E-12	IGLON5	Body mass index	GCST90435413
rs3745542	3.00E-12	KLK14,CTU1	Sialic acid-binding Ig-like lectin 9 levels	GCST90101552
rs76673459	3.00E-12	KLK14,CTU1	KLK12 protein levels	GCST90469698
rs114693949	3.00E-12	ETFB	SIGLEC6 protein levels	GCST90470634
rs17801525	4.00E-12	CTU1,SIGLEC18P	Sialic acid-binding Ig-like lectin 9 levels	GCST90101552
rs112632468	4.00E-12	KLK8,KLK7	KLK10 protein levels	GCST90469696
rs147395892	4.00E-12	SIGLEC9	SIGLEC7 protein levels	GCST90470635
rs12459419	5.00E-12	CD33	Neutrophil count	GCST90002398
rs2673908	5.00E-12	SIGLEC18P,SIGLEC9	Monocyte percentage of white cells	GCST004609
rs61752561	5.00E-12	KLK3	Prostate cancer	GCST011049
rs8102103	5.00E-12	SIGLECL1	Sialic acid-binding Ig-like lectin 9 levels	GCST90161587
rs111515946	6.00E-12	KLK7,KLK8	KLK12 protein levels	GCST90469698
rs2659091	8.00E-12	KLK5,PPIAP59	KLK7 protein levels	GCST90469706
rs543724886	8.00E-12	CTU1,KLK14	SIGLEC9 protein levels	GCST90470637
rs35112940	8.00E-12	CD33	Smoking initiation	GCST90243968
rs3865444	1.00E-11	SIGLEC22P,CD33	Red cell distribution width	GCST90002369
rs3865444	1.00E-11	SIGLEC22P,CD33	Eosinophill count (UKB data field 30150)	GCST90468068
rs1810020	1.00E-11	KLK3	Prostate cancer	GCST90013706
rs3865444	1.00E-11	SIGLEC22P,CD33	Reticulocyte percentage (UKB data field 30240)	GCST90468101
rs140989696	1.00E-11	KLK8,KLK7	KLK12 protein levels	GCST90469698
rs543724886	1.00E-11	CTU1,KLK14	KLK12 protein levels	GCST90469698
rs2659051	1.00E-11	KLK15,KLK3	Prostate cancer	GCST90503487
rs11668576	1.00E-11	VSIG10L	SIGLEC9 protein levels	GCST90470637
rs3865444	1.00E-11	SIGLEC22P,CD33	Hemoglobin A1c (HbA1c, minimum, inv-norm transformed)	GCST90479502
rs62113214	1.00E-11	KLK3	Chronic prostatitis (PheCode 601.12)	GCST90480408
rs148108087	2.00E-11	LINC01872	Body mass index	GCST009004
rs148108087	2.00E-11	LINC01872	Body mass index	GCST007039
rs35112940	2.00E-11	CD33	Smoking initiation	GCST90243985
rs143534987	2.00E-11	C19orf48P	KLK1 protein levels	GCST90469702
rs33978622	2.00E-11	CD33	monocyte (absolute count, maximum, inv-norm transformed)	GCST90479701
rs17632542	2.00E-11	KLK3	Chronic prostatitis (PheCode 601.12)	GCST90476155
rs142212375	2.00E-11	KLK15	Calculus of ureter (PheCode 594.3)	GCST90476147
rs61752561,rs2668	3.00E-11	KLK3; KLK15 - KLK3; KLK15 - KLK3; KLK3; KLK15 - KLK3; KLK2; KLK2	Prostate-specific antigen levels	GCST004093
rs148108087	3.00E-11	LINC01872	Body mass index	GCST009871
rs2735837	3.00E-11	KLK2	Prostate cancer	GCST90018685
rs150582568	3.00E-11	NKG7,LIM2	Myeloid cell surface antigen CD33 levels	GCST90161642
rs174776	3.00E-11	KLK3	Prostate cancer (late onset)	GCST90503489
rs11665698	3.00E-11	KLK3,KLK15	Prostate cancer	GCST90503487
rs182464120	3.00E-11	KLKP1	Prostate-specific antigen levels	GCST90461907
rs2739466	3.00E-11	KLK2	Prostatitis (PheCode 601.1)	GCST90476154
rs183578707	4.00E-11	KLK5	Prostate-specific antigen levels	GCST90461907
rs2569735	4.00E-11	KLK2,KLK3	Prostate-specific antigen levels	GCST90429172
rs33978622	4.00E-11	CD33	neutrophil (absolute count, maximum, inv-norm transformed)	GCST90479710
rs3865444	5.00E-11	SIGLEC22P,CD33	red cell diameter width (RDW, maximum, inv-norm transformed)	GCST90480671
rs3865444	6.00E-11	SIGLEC22P,CD33	Red cell distribution width	GCST90002372
rs12459419	6.00E-11	CD33	Basophil count	GCST90056179
rs148108087	6.00E-11	LINC01872	Body mass index	GCST90301650
rs148108087	7.00E-11	LINC01872	Body mass index (MTAG)	GCST90179150
rs17716331	8.00E-11	LIM2,NKG7	Neutrophil percentage of white cells	GCST90002399
rs2569735	8.00E-11	KLK2,KLK3	Prostate cancer	GCST90651067
rs62113216	9.00E-11	KLK3,KLK2	Prostate cancer	GCST90503487
rs12459419	1.00E-10	CD33	Plateletcrit	GCST004607
rs4802779	1.00E-10	IGLON5	Body mass index	GCST90018947
rs80122351	1.00E-10	KLK5,PPIAP59	Prostate-specific antigen levels	GCST90301330
rs115458416	1.00E-10	KLK5,PPIAP59	Kallikrein-8 levels	GCST90248164
rs12975233	1.00E-10	LINC01872	Glycated hemoglobin levels	GCST90019509
rs2569491	2.00E-10	KLK14	Blood protein levels	GCST006585
rs2673908	2.00E-10	SIGLEC18P,SIGLEC9	Granulocyte percentage of myeloid white cells	GCST004608
rs3865444	2.00E-10	SIGLEC22P,CD33	White blood cell count	GCST004610
rs2691217	2.00E-10	KLK13,KLK12	kallikrein-11 levels	GCST90012012
rs12459419	2.00E-10	CD33	Neutrophil count	GCST90056178
rs1354106	2.00E-10	CD33	Late-onset Alzheimer's disease	GCST90044699
rs2075803	2.00E-10	SIGLEC9	Body mass index	GCST90255621
rs4802778	2.00E-10	IGLON5,SIGLEC24P	Body mass index	GCST90255621
rs28536511	2.00E-10	SIGLECL1,LINC01872	ICD10 M54: Dorsalgia	GCST90103484
rs190901442	2.00E-10	KLK3,KLK15	Prostate-specific antigen levels	GCST90461907
rs11665698	2.00E-10	KLK3,KLK15	Prostate cancer (late onset)	GCST90503489
rs34681424	2.00E-10	SIGLEC9	Body mass index	GCST90428119
rs3865444	3.00E-10	SIGLEC22P,CD33	Reticulocyte count	GCST90002405
rs17632542	3.00E-10	KLK3	Hormone-sensitive cancer	GCST90102436
rs11084054,rs1014	3.00E-10	CTU1 x LINC02292 - SYNE3	Total PHF-tau (SNP x SNP interaction)	GCST010340
rs35112940	4.00E-10	CD33	High light scatter reticulocyte percentage of red cells	GCST90002386
rs2659051	4.00E-10	KLK15,KLK3	Prostate cancer (late onset)	GCST90503489
rs2075803	5.00E-10	SIGLEC9	C-reactive protein levels	GCST009777
rs2062655	5.00E-10	SIGLEC24P,IGLON5	Body mass index (MTAG)	GCST90179150
rs266849	6.00E-10	KLK3,KLK15	Serum prostate-specific antigen levels	GCST000919
rs12461354	6.00E-10	CD33	Myeloid cell surface antigen CD33 levels	GCST90248431
rs6509525	7.00E-10	IGLON5	Body mass index	GCST009001
rs2739472	8.00E-10	KLK2	Prostate-specific antigen levels (conditioned on lead SNPs)	GCST004094
rs61752561,rs2668	8.00E-10	KLK3; KLK15 - KLK3; KLK15 - KLK3; KLK3; KLK15 - KLK3; KLK2; KLK2	Prostate-specific antigen levels	GCST004093
rs62113216	8.00E-10	KLK3,KLK2	Prostate cancer (late onset)	GCST90503489
rs12459419	8.00E-10	CD33	Glycoprotein acetyls levels	GCST90454488
rs116623489	1.00E-09	CD33,SIGLEC22P	CD33 on CD33+ HLA DR+ CD14-	GCST90001957
rs7255024	1.00E-09	KLK4	Gut microbial network clusters (BlueViolet (at 3 months) x Household furry pet cat or dog (3 Months) interaction	GCST90569241
rs141135092	1.00E-09	PPIAP59,KLK4	Prostate-specific antigen levels	GCST90301330
rs7250000	2.00E-09	LIM2	Lymphocyte percentage of white cells	GCST90002389
rs3865444	2.00E-09	SIGLEC22P,CD33	Alzheimer's disease (late onset)	GCST001026
rs4802779	2.00E-09	IGLON5	Body mass index	GCST009871
rs11668077	2.00E-09	SIGLECL1	CD45 on CD33- HLA DR+	GCST90002046
rs112786111	2.00E-09	KLK15,KLK3	Prostate cancer	GCST90503487
rs56915529	2.00E-09	SIGLEC9	Kallikrein-10 levels	GCST90248155
rs1048328	2.00E-09	KLK11	Vitamin D levels	GCST90019526
rs2735839	3.00E-09	KLK3,KLK2	Prostate cancer	GCST008860
rs12983058	3.00E-09	SIGLEC9	High light scatter reticulocyte percentage of red cells	GCST90002386
rs2569444	3.00E-09	SIGLEC9	Serum alkaline phosphatase levels	GCST90018942
rs10417413	3.00E-09	SIGLEC21P,MIR8074	CD33 on CD66b++ myeloid cell	GCST90001951
rs11666870	3.00E-09	CTU1,KLK14	Serum levels of protein KLK14	GCST90090240
rs200711091	3.00E-09	KLK4,PPIAP59	Prostate-specific antigen levels	GCST90461907

rs112786111	3.00E-09	KLK15,KLK3	Prostate cancer (late onset)	GCST90503489
rs11671711,rs8122	3.00E-09	ECH1 x KCNG1 - RPSAP1	Diffuse plaques (SNP x SNP interaction)	GCST010341
rs61752561,rs2668	4.00E-09	KLK3; KLK15 - KLK3; KLK15 - KLK3; KLK3; KLK15 - KLK3; KLK2; KLK2	Prostate-specific antigen levels	GCST004093
rs10425377	4.00E-09	KLK10	KLK11 protein levels	GCST90277891
rs10410942	5.00E-09	KLK8	Blood protein levels	GCST006585
rs2691254	5.00E-09	CTU1	Blood protein levels	GCST006585
rs62113212	6.00E-09	KLK3	Prostate cancer aggressiveness	GCST002886
rs2735839	6.00E-09	KLK3,KLK2	Prostate cancer	GCST002944
rs3865444	6.00E-09	SIGLEC22P,CD33	Alzheimer's disease or family history of Alzheimer's disease	GCST007320
rs2075690	6.00E-09	KLK10	25-Hydroxyvitamin D levels	GCST90319504
rs2659051	6.00E-09	KLK15,KLK3	Prostate cancer	GCST011829
rs34733715	7.00E-09	NKG7,LIM2	Neutrophil count	GCST004629
rs3865444	7.00E-09	SIGLEC22P,CD33	Platelet count	GCST004603
rs113920094	7.00E-09	KLK15,KLK3	Prostate-specific antigen levels	GCST90301330
rs12983578	8.00E-09	CTU1	Lung function (FEV1/FVC)	GCST007080
rs12983058	8.00E-09	SIGLEC9	Reticulocyte fraction of red cells	GCST90002406
rs266849	8.00E-09	KLK3,KLK15	Prostate cancer	GCST90503487
rs2455069	9.00E-09	CD33	Triacylglyceride levels	GCST008922
rs116623489	9.00E-09	CD33,SIGLEC22P	CD33 on CD33+ HLA DR+	GCST90001956
rs3760731	9.00E-09	KLK2	Prostate-specific antigen levels	GCST90429172
rs12459419	1.00E-08	CD33	Red cell distribution width	GCST007074
rs10424878	1.00E-08	KLK2	PCA3 expression level	GCST001946
rs143398878	1.00E-08	ACP4	Heel bone mineral density x serum urate levels interaction	GCST012489
rs11666508	1.00E-08	KLK15	Kidney stone disease	GCST90652507
rs7250000	1.00E-08	LIM2	Neutrophils and lymphocytes in blood (confirmatory factor analysis Factor 35)	GCST90309369
rs2075803	1.00E-08	SIGLEC9	Body mass index	GCST008129
rs2459143	1.00E-08	SIGLEC9	Gut microbial network clusters (BlueViolet (at 3 months) x Homesmokers interaction	GCST90569276
rs78177998	1.00E-08	KLK15,KLK3	Prostate cancer	GCST90503487
rs2659124	2.00E-08	KLK3,KLK15	Prostate cancer	GCST008860
rs2735839	2.00E-08	KLK3,KLK2	Prostate cancer	GCST002944
rs2659051	2.00E-08	KLK15,KLK3	Prostate cancer	GCST011048
rs3852865	2.00E-08	MIR8074,SIGLEC22P	Alzheimer's disease or peptic ulcer disease	GCST90134604
rs7245846	2.00E-08	CD33	Alzheimer's disease or peptic ulcer disease	GCST90134604
rs2739459	2.00E-08	KLK2	Prostate cancer	GCST011829
rs11665748	2.00E-08	KLK15,KLK3	Prostate cancer	GCST90274717
rs7255024	2.00E-08	KLK4	Gut microbial network clusters (BlueViolet (at 3 months) x Household furry pet dog (3 Months) interaction	GCST90569242
rs11084054,rs1326	2.00E-08	CTU1 x COL22A1	Total PHF-tau (SNP x SNP interaction)	GCST010340
rs3116139	3.00E-08	LIM2,NKG7	Glaucoma	GCST002529
rs2455069	3.00E-08	CD33	Triacylglyceride levels	GCST008922
rs8111074	3.00E-08	LINC01872	Adult body size	GCST010988
rs2659052	3.00E-08	KLK15,KLK3	Cancer (pleiotropy)	GCST90011822
rs11084061	3.00E-08	SIGLECL1	CD33 on CD33dim HLA DR-	GCST90001953
rs266876	3.00E-08	KLK3	Prostate cancer	GCST011829
rs10426620	3.00E-08	KLK13	ut microbiome abundance (class Bifidobacterium catenulatum (at 1 year) x Any household furry Pets (3 Months) interactio	GCST90568715
rs2739430	3.00E-08	KLK10	Serum 25-Hydroxyvitamin D level variance	GCST90000621
rs2673908	3.00E-08	SIGLEC18P,SIGLEC9	Alanine aminotransferase levels	GCST90428729
rs3865444	4.00E-08	SIGLEC22P,CD33	Alzheimer's disease (late onset)	GCST007319
rs3865444	4.00E-08	SIGLEC22P,CD33	Alzheimer's disease	GCST009021
rs74705037	4.00E-08	KLK8	KLK8 protein levels	GCST90277810
rs78353057	4.00E-08	KLK6	Gut microbiome abundance (class Roseburia sp. 7) (at 1 year)	GCST90565206
rs61752567	4.00E-08	KLK11	Gut microbial network clusters (Tan (at 3 months) x Summer Birth (Jun-Aug) interaction	GCST90569205
rs6509525	5.00E-08	IGLON5	Metabolic syndrome	GCST90444487
rs2455069	5.00E-08	CD33	Triacylglyceride levels	GCST008922
rs3909863,rs14219	5.00E-08	TMEM277P x CAST	Total PHF-tau (SNP x SNP interaction)	GCST010340

Supplemental Table 14. PolarMorphism metrics of LD-independent loci with pleiotropic effects on HP, ADHD, and ASD

rsID	rs325485	rs2207286
chr	5	1
hg19 position	103,995,368	88,828,592
Effect Allele	A	A
Other Allele	G	G
HP beta	0.026	0.019
ASD beta	0.081	0.096
ADHD beta	0.067	0.036
HP p-value	2.57E-08	1.70E-04
ASD p-value	4.71E-03	1.45E-03
ADHD p-value	2.74E-12	2.86E-04
r	7.86	5.28
angle	2.46	3.10
z.whitened.HP	4.60	3.11
z.whitened.ASD	2.72	3.09
z.whitened.ADHD	5.76	2.95
r.pval	2.42E-13	3.86E-06
r.qval	1.22E-08	7.14E-03
theta.pval	2.46E-04	3.77E-04
theta.qval	1.71E-03	1.71E-03

Supplemental Table 15. 95% credible sets available from Open Targets Platform including rs325485.

leadVariant	credibleSetSize	reportedTrait	study	P value	Fine-mapping method
5_104596006_G_A	115	Hand grip strength	GCST005830	2.00E-15	PICS fine-mapped credible set based on reported top hit
5_104596006_G_A	127	Hand grip strength	GCST005829	2.00E-13	PICS fine-mapped credible set based on reported top hit
5_104659667_A_G	103	Autism spectrum disorder (MTAG)	GCST012092	4.00E-13	PICS fine-mapped credible set based on reported top hit
5_104612267_T_C	17	Depression medications	FINNGEN_R12_ANTIDEPRESSANTS	5.32E-13	SuSiE fine-mapped credible set with in-sample LD
5_104609477_C_T	125	Waist-hip ratio	GCST008996	2.00E-12	PICS fine-mapped credible set based on reported top hit
5_104604714_T_A	120	Body fat percentage	GCST90020232	9.00E-12	PICS fine-mapped credible set based on reported top hit
5_104608319_G_T	130	Risk-taking tendency (4-domain principal component model)	GCST007323	1.00E-11	PICS fine-mapped credible set based on reported top hit
5_104686538_C_G	130	Asthma and major depressive disorder	GCST008921	2.00E-11	PICS fine-mapped credible set based on reported top hit
5_104628884_G_A	30	Self-reported math ability (MTAG)	GCST006569	2.00E-11	PICS fine-mapped credible set based on reported top hit
5_104606359_AT_A	133	Waist circumference (UKB data field 48)	GCST90310188	2.58E-11	PICS fine-mapped credible set extracted from summary statistics
5_104676602_C_G	149	Autism and major depressive disorder (MTAG)	GCST007553	3.00E-11	PICS fine-mapped credible set based on reported top hit
5_104636656_G_A	31	Lifetime major depressive disorder (MTAG)	GCST90449009	3.00E-11	PICS fine-mapped credible set based on reported top hit
5_104606359_AT_A	135	Trunk fat mass (UKB data field 23128)	GCST90429597	4.86E-11	PICS fine-mapped credible set extracted from summary statistics
5_104604714_T_A	128	High density lipoprotein cholesterol levels	GCST90020233	5.00E-11	PICS fine-mapped credible set based on reported top hit
5_104619660_T_C	135	Urinary potassium excretion	GCST008648	7.00E-11	PICS fine-mapped credible set based on reported top hit
5_104628884_G_A	37	Chronotype	GCST007576	8.00E-11	PICS fine-mapped credible set based on reported top hit
5_104659667_A_G	132	Depression	GCST90014436	8.43E-11	PICS fine-mapped credible set extracted from summary statistics
5_104655775_C_A	127	Autism spectrum disorder (MTAG)	GCST010643	1.00E-10	PICS fine-mapped credible set based on reported top hit
5_104606359_AT_A	137	Whole body fat mass (UKB data field 23100)	GCST90429573	1.34E-10	PICS fine-mapped credible set extracted from summary statistics
5_104665051_G_A	36	HDL cholesterol	GCST90018956	1.76E-10	SuSiE fine-mapped credible set with out-of-sample LD
5_104670966_A_G	128	Depression	GCST90058037	2.00E-10	PICS fine-mapped credible set based on reported top hit
5_104597772_C_T	140	Body mass index	GCST009004	2.00E-10	PICS fine-mapped credible set based on reported top hit
5_104604714_T_A	134	Body mass index	GCST90301650	2.00E-10	PICS fine-mapped credible set based on reported top hit
5_104611504_C_T	138	Body mass index	GCST009871	3.00E-10	PICS fine-mapped credible set based on reported top hit
5_104646025_T_G	39	Urinary sodium excretion	GCST008647	3.00E-10	PICS fine-mapped credible set based on reported top hit
5_104608319_G_T	139	Body mass index (MTAG)	GCST90179150	3.38E-10	PICS fine-mapped credible set extracted from summary statistics
5_104643132_A_G	74	Creatinine enzymatic in urine (UKB data field 30510)	GCST90013987	3.69E-10	SuSiE fine-mapped credible set with out-of-sample LD
5_104676602_C_G	152	Anxiety disorder	GCST90446654	4.00E-10	PICS fine-mapped credible set based on reported top hit
5_104608319_G_T	139	Predicted visceral adipose tissue	GCST008744	4.00E-10	PICS fine-mapped credible set based on reported top hit
5_104703862_A_ATG	93	Hand grip strength left (UKB data field 46)	GCST90014019	4.03E-10	SuSiE fine-mapped credible set with out-of-sample LD
5_104665051_G_A	23	Weight	GCST90018949	4.51E-10	SuSiE fine-mapped credible set with out-of-sample LD
5_104611504_C_T	139	Body mass index	GCST007039	5.00E-10	PICS fine-mapped credible set based on reported top hit
5_104620815_C_G	43	Migraine or type 2 diabetes	GCST90244660	8.00E-10	PICS fine-mapped credible set based on reported top hit
5_104636656_G_A	47	Lifetime major depressive disorder (AutoComplete Impute and observed)	GCST90449002	1.00E-09	PICS fine-mapped credible set based on reported top hit
5_104606359_AT_A	142	Leg fat mass right (UKB data field 23112)	GCST90429586	1.28E-09	PICS fine-mapped credible set extracted from summary statistics
5_104678081_G_A	148	Tea intake (UKB data field 1488)	GCST90132983	1.68E-09	SuSiE fine-mapped credible set with out-of-sample LD
5_104671732_G_C	137	Positive affect	GCST007338	2.00E-09	PICS fine-mapped credible set based on reported top hit
5_104646025_T_G	46	Lifetime major depressive disorder (SoftImpute and observed)	GCST90449000	2.00E-09	PICS fine-mapped credible set based on reported top hit
5_104620815_C_G	46	Neuroticism (MTAG)	GCST005326	2.00E-09	PICS fine-mapped credible set based on reported top hit
5_104604714_T_A	142	Body mass index	GCST90302889	2.27E-09	PICS fine-mapped credible set extracted from summary statistics
5_104604714_T_A	142	Weight	GCST90302891	2.64E-09	PICS fine-mapped credible set extracted from summary statistics
5_104606359_AT_A	144	Leg fat mass left (UKB data field 23116)	GCST90429589	2.87E-09	PICS fine-mapped credible set extracted from summary statistics
5_104578438_T_TCAAACATGA	146	Body mass index	GCST90435413	3.00E-09	PICS fine-mapped credible set based on reported top hit
5_104676602_C_G	102	emphysema, asthma, rhinitis, eczema, allergy diagnosed by doctor: PHESANT recoding	GCST90044359	3.33E-09	SuSiE fine-mapped credible set with out-of-sample LD
5_104606359_AT_A	144	Leg fat percentage right (UKB data field 23111)	GCST90429572	3.95E-09	PICS fine-mapped credible set extracted from summary statistics
5_104604714_T_A	143	Metabolic biomarkers (multivariate analysis)	GCST90038594	4.00E-09	PICS fine-mapped credible set based on reported top hit
5_104678081_G_A	147	Age of onset of adult onset asthma	GCST90255668	5.00E-09	PICS fine-mapped credible set based on reported top hit
5_104620815_C_G	49	Loneliness	GCST009463	5.00E-09	PICS fine-mapped credible set based on reported top hit
5_104646025_T_G	120	Triglyceride levels	GCST90239664	6.67E-09	SuSiE fine-mapped credible set with out-of-sample LD
5_104604714_T_A	145	Body fat percentage (UKB data field 23099)	GCST90429576	6.72E-09	PICS fine-mapped credible set extracted from summary statistics
5_104608319_G_T	68	High density lipoprotein cholesterol levels (UKB data field 30760)	GCST90014007	7.03E-09	SuSiE fine-mapped credible set with out-of-sample LD
5_104636656_G_A	55	Loneliness	GCST006923	8.00E-09	PICS fine-mapped credible set based on reported top hit
5_104659667_A_G	148	Triglyceride levels	GCST010244	9.00E-09	PICS fine-mapped credible set based on reported top hit
5_104671732_G_C	143	Life satisfaction	GCST007337	9.00E-09	PICS fine-mapped credible set based on reported top hit
5_104665051_G_A	35	Body mass index	GCST90018947	9.65E-09	SuSiE fine-mapped credible set with out-of-sample LD
5_104636656_G_A	56	Depression (broad)	GCST90281043	1.00E-08	PICS fine-mapped credible set based on reported top hit
5_104659667_A_G	74	Type 2 diabetes, definitions combined	FINNGEN_R12_T2D	4.13E-07	SuSiE fine-mapped credible set with in-sample LD

Supplemental Table 16. Information regarding expression and splicing quantitative trait loci related to rs325485 available from GTEx v 10.

Gencode Id	Variant Id	SNP Id	NES	P-Value	Tissue
ENSG00000251574.8	chr5_104659667_A_G_b38	rs325485	-0.21	2.10E-15	Testis

Gencode Id	Variant Id	SNP Id	Intron Id	NES	P-Value	Tissue
ENSG00000251574.8	chr5_104659667_A_G_b38	rs325485	104763383:104773613:clu_94270_-	0.37	2.80E-08	Testis
ENSG00000251574.8	chr5_104659667_A_G_b38	rs325485	104630656:104763280:clu_94270_-	0.24	3.70E-07	Testis
ENSG00000251574.8	chr5_104659667_A_G_b38	rs325485	104739412:104739497:clu_94270_-	-0.32	4.50E-07	Testis

Supplemental Table 17. 95% credible sets available from Open Targets Platform including rs2207286.

leadVariant	credibleSetSize	reportedTrait	study	P value	Fine-mapping method
1_88332586_A_C	73	Insomnia	GCST90131901	3.00E-14	PICS fine-mapped credible set based on reported top hit
1_88316098_T_C	62	Diseases of middle ear and mastoid	FINNGEN_R12_H8_MIDDLEMASTOID	3.90E-11	SuSiE fine-mapped credible set with in-sample LD
1_88282464_G_A	81	Hearing loss	GCST90132906	2.00E-10	PICS fine-mapped credible set based on reported top hit
1_88325277_T_C	84	General risk tolerance (MTAG)	GCST007325	2.00E-10	PICS fine-mapped credible set based on reported top hit
1_88353792_C_A	78	Insomnia	GCST90131903	3.00E-10	PICS fine-mapped credible set based on reported top hit
1_88315659_C_T	61	Nonsuppurative otitis media	FINNGEN_R12_H8_NONSUPPNAS	4.65E-10	SuSiE fine-mapped credible set with in-sample LD
1_88309473_G_A	49	F-acquired taste liking (derived food-liking factor)	GCST90094791	3.61E-09	SuSiE fine-mapped credible set with out-of-sample LD
1_88332586_A_C	73	Pain (limb, back, neck, head abdominally)	FINNGEN_R12_PAIN	1.16E-08	SuSiE fine-mapped credible set with in-sample LD
1_88362957_A_C	72	Green olives liking	GCST90094775	2.31E-08	SuSiE fine-mapped credible set with out-of-sample LD
1_88332586_A_C	63	Allergic rhinitis	FINNGEN_R12_ALLERG_RHINITIS	2.67E-08	SuSiE fine-mapped credible set with in-sample LD
1_88324468_C_T	95	Hearing difficulty problems background noise (UKB data field 2257)	GCST90042885	5.06E-08	SuSiE fine-mapped credible set with out-of-sample LD

Supplemental Table 18. Bonferroni-significant terms shared between HP, ASD, TS, and ADHD

GO ID	GO description	HP		ASD		ADHD		TS	
		Enrichment	P	Enrichment	P	Enrichment	P	Enrichment	P
GO:0005478	transporter activity	0.90	1.85E-57	1.17	6.42E-45	1.00	2.08E-23	1.11	2.47E-45
GO:0070161	anchoring junction	0.90	4.72E-64	0.87	2.84E-29	0.85	1.40E-30	0.88	4.70E-41
GO:0099080	supramolecular complex	1.02	1.51E-64	0.87	7.25E-26	0.91	3.25E-37	0.99	1.48E-27
GO:0070727	cellular macromolecule localization	1.00	1.25E-30	0.94	2.51E-45	1.02	9.07E-26	0.96	1.13E-46
GO:0009719	response to endogenous stimulus	0.97	6.48E-46	1.04	2.14E-43	1.05	7.11E-23	1.08	1.13E-24
GO:1901135	carbohydrate derivative metabolic process	0.87	9.98E-39	1.22	1.82E-33	0.86	2.36E-26	1.08	8.96E-34
GO:0098590	plasma membrane region	1.06	1.23E-07	0.87	9.87E-52	1.03	1.47E-24	0.89	3.94E-47
GO:0040011	locomotion	0.96	3.42E-31	0.93	2.12E-50	1.00	4.50E-19	0.94	9.96E-25
GO:0099081	supramolecular polymer	1.03	2.86E-44	0.88	9.95E-28	0.87	2.26E-28	1.04	2.99E-20
GO:0044281	small molecule metabolic process	1.07	7.67E-13	1.38	4.99E-29	0.96	5.12E-26	1.19	1.11E-41
GO:0060089	molecular transducer activity	1.01	9.78E-21	0.87	9.08E-29	1.09	1.92E-07	0.93	1.27E-46
GO:0006394	RNA processing	1.28	1.57E-32	1.09	4.61E-13	1.20	5.25E-34	1.14	1.30E-23
GO:0048471	perinuclear region of cytoplasm	0.93	7.46E-47	1.06	2.92E-18	0.94	7.47E-12	1.11	1.45E-23
GO:0006629	lipid metabolic process	1.08	1.74E-17	1.17	9.26E-40	1.03	2.08E-23	1.38	4.39E-15
GO:0016477	cell migration	0.96	1.85E-21	0.96	2.23E-38	0.99	1.52E-14	0.92	1.73E-17
GO:0019900	kinase binding	1.09	1.76E-19	1.11	3.97E-16	0.95	3.11E-29	1.05	1.18E-26
GO:0016491	oxidoreductase activity	1.32	1.21E-33	1.28	7.10E-17	1.16	3.44E-24	1.08	5.18E-15
GO:0005198	structural molecule activity	1.22	4.08E-30	0.77	3.68E-21	0.89	3.77E-25	1.03	2.02E-11
GO:0031252	cell leading edge	0.97	2.97E-29	1.01	1.06E-19	0.92	1.34E-22	0.85	4.79E-16
GO:0019725	cellular homeostasis	1.07	4.60E-13	1.17	9.68E-24	1.10	1.07E-07	1.09	1.37E-35
GO:0005543	phospholipid binding	1.02	7.45E-15	1.15	7.49E-18	0.83	1.74E-24	1.00	2.11E-21
GO:0005813	centrosome	0.93	3.98E-28	1.04	8.81E-20	1.07	6.95E-18	1.17	1.07E-12
GO:0044297	cell body	1.04	6.55E-22	1.08	2.23E-16	1.05	1.06E-17	0.94	1.84E-20
GO:0044770	cell cycle phase transition	0.97	1.14E-18	1.40	1.95E-18	0.96	6.47E-26	1.04	5.05E-12
GO:0043235	receptor complex	0.95	2.43E-28	0.88	1.22E-13	1.04	9.65E-15	0.70	4.30E-12
GO:0061919	process utilizing autophagic mechanism	0.93	6.48E-23	1.22	1.24E-11	0.84	1.19E-19	1.09	8.84E-13
GO:0044723	carbohydrate metabolic process	0.87	3.32E-22	1.55	3.87E-12	1.11	9.81E-09	1.25	4.00E-23
GO:0098552	side of membrane	0.96	4.62E-27	1.10	6.43E-12	1.04	9.65E-09	0.95	1.35E-17
GO:0045177	apical part of cell	1.19	1.04E-21	1.07	2.66E-14	1.13	4.45E-11	1.07	1.23E-17
GO:0019001	guanyl nucleotide binding	0.85	5.44E-15	1.26	3.65E-12	1.19	2.22E-20	1.21	6.81E-15
GO:0003682	chromatin binding	1.10	2.06E-26	0.91	4.01E-11	1.20	9.06E-09	0.92	3.81E-15
GO:0030396	extrinsic component of membrane	1.11	1.25E-18	0.92	1.79E-11	1.02	1.24E-16	1.01	4.03E-14
GO:0106310	protein serine kinase activity	0.77	2.33E-15	1.01	1.86E-07	0.85	1.50E-18	0.93	1.14E-15
GO:0022804	active transmembrane transporter activity	1.00	1.39E-13	1.36	8.62E-13	1.18	2.93E-14	1.23	3.61E-15
GO:0048511	rhythmic process	0.99	8.45E-15	0.79	4.09E-07	0.91	1.16E-16	0.84	2.48E-16
GO:0009434	motile cilium	0.94	2.58E-12	1.07	1.82E-07	1.23	5.48E-24	1.51	5.46E-11
GO:0050673	epithelial cell proliferation	0.93	6.50E-19	1.09	1.58E-10	0.98	2.61E-08	0.91	2.18E-14
GO:0050954	sensory perception of mechanical stimulus	2.17	2.96E-19	0.95	3.86E-11	0.64	3.48E-11	0.67	2.43E-08
GO:0030012	establishment or maintenance of cell polarity	0.83	4.68E-16	0.98	2.02E-10	0.84	9.61E-14	0.80	3.75E-09
GO:0016607	nuclear speck	0.82	9.66E-12	1.14	5.34E-12	1.02	6.50E-14	1.18	1.34E-11
GO:0099568	cytoplasmic region	1.15	1.07E-13	0.83	2.68E-10	0.75	2.46E-12	0.93	1.40E-12
GO:0060485	mesenchyme development	1.04	7.88E-18	0.94	1.27E-11	1.09	1.38E-09	0.98	2.38E-09
GO:0030483	site of polarized growth	0.75	4.70E-13	0.95	2.17E-10	1.10	6.57E-14	1.04	6.00E-10
GO:0070085	glycosylation	0.74	9.67E-16	1.26	5.94E-10	0.95	4.44E-11	1.02	4.13E-10
GO:0045178	basal part of cell	0.99	5.37E-12	1.00	5.89E-09	0.91	1.42E-12	1.25	6.31E-13
GO:0004002	ATP hydrolysis activity	0.98	6.40E-13	0.90	8.27E-11	0.99	2.59E-12	0.96	6.40E-10
GO:0006066	alcohol metabolic process	1.09	1.52E-14	1.18	2.30E-08	1.18	1.90E-11	1.16	4.83E-11
GO:1904951	positive regulation of establishment of protein localization	0.86	7.55E-15	1.07	2.49E-08	0.80	1.31E-12	1.04	3.79E-08
GO:0051258	protein polymerization	1.35	1.28E-14	1.10	1.67E-08	0.88	3.06E-10	1.26	1.69E-09
GO:0019207	kinase regulator activity	0.92	1.93E-10	1.34	3.34E-10	0.84	1.57E-09	1.34	6.57E-12
GO:0070382	exocytic vesicle	1.29	4.51E-11	0.76	3.85E-07	1.25	2.58E-13	0.91	4.88E-08
GO:0098562	cytoplasmic side of membrane	1.25	2.45E-12	1.14	2.29E-08	0.99	3.07E-08	1.36	1.85E-10
GO:0019867	outer membrane	0.82	5.86E-14	1.20	2.65E-08	0.70	4.32E-07	1.49	2.69E-08
GO:0008037	cell recognition	0.84	2.08E-08	0.98	1.70E-08	0.73	2.72E-11	0.80	9.24E-09
GO:0051402	neuron apoptotic process	1.08	3.83E-08	1.40	3.57E-09	1.08	4.00E-11	0.77	2.95E-08
GO:0036142	cilium movement	0.81	1.01E-08	1.43	1.87E-07	1.08	3.99E-09	1.38	3.27E-08

Supplemental Table 19. Genome-wide significant CD33 expression quantitative trait loci available from GTEx v10.

Gencode Id	Gene Symbol	Variant Id	SNP Id	NES	P-Value	Tissue
ENSG00000105383.15	CD33	chr19_51225847_CCCGG_C_b38	rs201074739	-0.64	1.9E-26	Whole Blood
ENSG00000105383.15	CD33	chr19_51258097_G_C_b38	rs62115997	-0.53	8.6E-21	Whole Blood
ENSG00000105383.15	CD33	chr19_51258337_G_C_b38	rs62115998	-0.53	8.6E-21	Whole Blood
ENSG00000105383.15	CD33	chr19_51258360_G_A_b38	rs62115999	-0.53	8.6E-21	Whole Blood
ENSG00000105383.15	CD33	chr19_51267038_G_A_b38	rs11668077	-0.57	1.6E-18	Whole Blood
ENSG00000105383.15	CD33	chr19_51222568_G_T_b38	rs273640	0.15	8E-18	Whole Blood
ENSG00000105383.15	CD33	chr19_51223655_A_C_b38	rs1710398	0.14	9.9E-18	Whole Blood
ENSG00000105383.15	CD33	chr19_51224066_A_G_b38	rs1697553	0.14	1.3E-17	Whole Blood
ENSG00000105383.15	CD33	chr19_51225385_A_G_b38	rs2455069	0.14	1.3E-17	Whole Blood
ENSG00000105383.15	CD33	chr19_51217369_A_T_b38	rs1566576	0.14	2E-17	Whole Blood
ENSG00000105383.15	CD33	chr19_51220290_C_G_b38	rs273637	0.14	2.2E-17	Whole Blood
ENSG00000105383.15	CD33	chr19_51220856_A_G_b38	rs273638	0.14	2.6E-17	Whole Blood
ENSG00000105383.15	CD33	chr19_51220906_A_G_b38	rs273639	0.14	2.6E-17	Whole Blood
ENSG00000105383.15	CD33	chr19_51221277_A_ACACAATATTTTAC_b38	rs11273989	0.14	2.6E-17	Whole Blood
ENSG00000105383.15	CD33	chr19_51219326_A_G_b38	rs12609179	0.14	4.8E-17	Whole Blood
ENSG00000105383.15	CD33	chr19_51215061_T_A_b38	rs1697573	0.14	6.5E-17	Whole Blood
ENSG00000105383.15	CD33	chr19_51223099_G_C_b38	rs1399837	0.14	6.5E-17	Whole Blood
ENSG00000105383.15	CD33	chr19_51215581_C_T_b38	rs273634	0.14	9.7E-17	Whole Blood
ENSG00000105383.15	CD33	chr19_51224937_T_C_b38	rs2459141	0.14	1E-16	Whole Blood
ENSG00000105383.15	CD33	chr19_51225847_CCCGG_C_b38	rs201074739	-0.56	7.6E-16	Nerve - Tibial
ENSG00000105383.15	CD33	chr19_51242324_T_G_b38	rs1034521	0.22	1.7E-15	Adipose - Subcutaneous
ENSG00000105383.15	CD33	chr19_51243667_G_A_b38	rs12461354	0.22	1.7E-15	Adipose - Subcutaneous
ENSG00000105383.15	CD33	chr19_51246334_C_T_b38	rs1566578	0.22	2.5E-15	Adipose - Subcutaneous
ENSG00000105383.15	CD33	chr19_51246958_G_A_b38	rs11084061	0.22	2.5E-15	Adipose - Subcutaneous
ENSG00000105383.15	CD33	chr19_51246983_T_C_b38	rs11084062	0.22	2.5E-15	Adipose - Subcutaneous
ENSG00000105383.15	CD33	chr19_51248679_C_A_b38	rs989502	0.22	2.5E-15	Adipose - Subcutaneous
ENSG00000105383.15	CD33	chr19_51248695_G_A_b38	rs989505	0.22	2.5E-15	Adipose - Subcutaneous
ENSG00000105383.15	CD33	chr19_51248696_C_A_b38	rs1319675	0.22	2.5E-15	Adipose - Subcutaneous
ENSG00000105383.15	CD33	chr19_51247502_T_C_b38	rs12461282	0.22	4.7E-15	Adipose - Subcutaneous
ENSG00000105383.15	CD33	chr19_51240790_G_T_b38	rs8103085	0.22	8.1E-15	Adipose - Subcutaneous
ENSG00000105383.15	CD33	chr19_51250704_G_C_b38	rs7253829	0.22	9.2E-15	Adipose - Subcutaneous
ENSG00000105383.15	CD33	chr19_51250705_G_A_b38	rs7253831	0.22	9.2E-15	Adipose - Subcutaneous
ENSG00000105383.15	CD33	chr19_51243997_T_C_b38	rs73612300	0.21	1.3E-14	Adipose - Subcutaneous
ENSG00000105383.15	CD33	chr19_51225847_CCCGG_C_b38	rs201074739	-0.43	6.9E-14	Adipose - Subcutaneous
ENSG00000105383.15	CD33	chr19_51250875_G_T_b38	rs17801843	0.2	1.7E-13	Adipose - Subcutaneous
ENSG00000105383.15	CD33	chr19_51225847_CCCGG_C_b38	rs201074739	-0.53	8.4E-13	Artery - Tibial
ENSG00000105383.15	CD33	chr19_51239889_G_C_b38	rs1803254	0.38	1.8E-12	Spleen
ENSG00000105383.15	CD33	chr19_51239889_G_C_b38	rs1803254	0.2	1.9E-12	Adipose - Subcutaneous
ENSG00000105383.15	CD33	chr19_51247502_T_C_b38	rs12461282	0.39	2.3E-12	Spleen
ENSG00000105383.15	CD33	chr19_51258097_G_C_b38	rs62115997	-0.45	4.4E-12	Nerve - Tibial
ENSG00000105383.15	CD33	chr19_51258337_G_C_b38	rs62115998	-0.45	4.4E-12	Nerve - Tibial
ENSG00000105383.15	CD33	chr19_51258360_G_A_b38	rs62115999	-0.45	4.4E-12	Nerve - Tibial
ENSG00000105383.15	CD33	chr19_51242324_T_G_b38	rs1034521	0.38	5.7E-12	Spleen
ENSG00000105383.15	CD33	chr19_51243667_G_A_b38	rs12461354	0.38	5.7E-12	Spleen
ENSG00000105383.15	CD33	chr19_51246334_C_T_b38	rs1566578	0.38	5.7E-12	Spleen
ENSG00000105383.15	CD33	chr19_51246958_G_A_b38	rs11084061	0.38	5.7E-12	Spleen
ENSG00000105383.15	CD33	chr19_51246983_T_C_b38	rs11084062	0.38	5.7E-12	Spleen
ENSG00000105383.15	CD33	chr19_51248679_C_A_b38	rs989502	0.38	5.7E-12	Spleen
ENSG00000105383.15	CD33	chr19_51248695_G_A_b38	rs989505	0.38	5.7E-12	Spleen
ENSG00000105383.15	CD33	chr19_51248696_C_A_b38	rs1319675	0.38	5.7E-12	Spleen
ENSG00000105383.15	CD33	chr19_51258097_G_C_b38	rs62115997	-0.47	7E-12	Artery - Tibial
ENSG00000105383.15	CD33	chr19_51258337_G_C_b38	rs62115998	-0.47	7E-12	Artery - Tibial
ENSG00000105383.15	CD33	chr19_51258360_G_A_b38	rs62115999	-0.47	7E-12	Artery - Tibial
ENSG00000105383.15	CD33	chr19_51240790_G_T_b38	rs8103085	0.29	8.7E-12	Adipose - Visceral (Omentum)
ENSG00000105383.15	CD33	chr19_51240790_G_T_b38	rs8103085	0.39	8.8E-12	Spleen
ENSG00000105383.15	CD33	chr19_51242324_T_G_b38	rs1034521	0.28	1.1E-11	Adipose - Visceral (Omentum)
ENSG00000105383.15	CD33	chr19_51243667_G_A_b38	rs12461354	0.28	1.1E-11	Adipose - Visceral (Omentum)
ENSG00000105383.15	CD33	chr19_51246334_C_T_b38	rs1566578	0.28	1.1E-11	Adipose - Visceral (Omentum)
ENSG00000105383.15	CD33	chr19_51246958_G_A_b38	rs11084061	0.28	1.1E-11	Adipose - Visceral (Omentum)
ENSG00000105383.15	CD33	chr19_51246983_T_C_b38	rs11084062	0.28	1.1E-11	Adipose - Visceral (Omentum)
ENSG00000105383.15	CD33	chr19_51248679_C_A_b38	rs989502	0.28	1.1E-11	Adipose - Visceral (Omentum)
ENSG00000105383.15	CD33	chr19_51248695_G_A_b38	rs989505	0.28	1.1E-11	Adipose - Visceral (Omentum)
ENSG00000105383.15	CD33	chr19_51248696_C_A_b38	rs1319675	0.28	1.1E-11	Adipose - Visceral (Omentum)
ENSG00000105383.15	CD33	chr19_51258097_G_C_b38	rs62115997	-0.36	1.2E-11	Adipose - Subcutaneous
ENSG00000105383.15	CD33	chr19_51258337_G_C_b38	rs62115998	-0.36	1.2E-11	Adipose - Subcutaneous
ENSG00000105383.15	CD33	chr19_51258360_G_A_b38	rs62115999	-0.36	1.2E-11	Adipose - Subcutaneous
ENSG00000105383.15	CD33	chr19_51267038_G_A_b38	rs11668077	-0.53	1.3E-11	Artery - Tibial
ENSG00000105383.15	CD33	chr19_51225847_CCCGG_C_b38	rs201074739	-0.56	1.4E-11	Skin - Sun Exposed (Lower leg)
ENSG00000105383.15	CD33	chr19_51247502_T_C_b38	rs12461282	0.28	1.4E-11	Adipose - Visceral (Omentum)
ENSG00000105383.15	CD33	chr19_51250704_G_C_b38	rs7253829	0.27	2.9E-11	Adipose - Visceral (Omentum)
ENSG00000105383.15	CD33	chr19_51250705_G_A_b38	rs7253831	0.27	2.9E-11	Adipose - Visceral (Omentum)
ENSG00000105383.15	CD33	chr19_51250704_G_C_b38	rs7253829	0.37	3E-11	Spleen
ENSG00000105383.15	CD33	chr19_51250705_G_A_b38	rs7253831	0.37	3E-11	Spleen
ENSG00000105383.15	CD33	chr19_51256436_A_G_b38	rs1501447	0.18	4.2E-11	Adipose - Subcutaneous
ENSG00000105383.15	CD33	chr19_51250875_G_T_b38	rs17801843	0.35	7.5E-11	Spleen
ENSG00000105383.15	CD33	chr19_51225847_CCCGG_C_b38	rs201074739	-0.58	8.3E-11	Breast - Mammary Tissue
ENSG00000105383.15	CD33	chr19_51267038_G_A_b38	rs11668077	-0.49	8.3E-11	Nerve - Tibial

ENSG00000105383.15	CD33	chr19_51247868_C_T_b38	rs11084063	0.19	1.8E-10	Adipose - Subcutaneous
ENSG00000105383.15	CD33	chr19_51248395_G_T_b38	rs989504	0.19	1.8E-10	Adipose - Subcutaneous
ENSG00000105383.15	CD33	chr19_51334106_T_G_b38	rs11668576	-0.45	2E-10	Whole Blood
ENSG00000105383.15	CD33	chr19_51217988_G_A_b38	rs79254320	0.26	2.5E-10	Whole Blood
ENSG00000105383.15	CD33	chr19_51215061_T_A_b38	rs1697573	0.16	2.7E-10	Lung
ENSG00000105383.15	CD33	chr19_51223655_A_C_b38	rs1710398	0.15	3.5E-10	Lung
ENSG00000105383.15	CD33	chr19_51222568_G_T_b38	rs273640	0.14	3.8E-10	Nerve - Tibial
ENSG00000105383.15	CD33	chr19_51256436_A_G_b38	rs1501447	0.32	4E-10	Spleen
ENSG00000105383.15	CD33	chr19_51223655_A_C_b38	rs1710398	0.14	4.3E-10	Nerve - Tibial
ENSG00000105383.15	CD33	chr19_51224066_A_G_b38	rs1697553	0.14	4.3E-10	Nerve - Tibial
ENSG00000105383.15	CD33	chr19_51255314_A_T_b38	rs7256372	0.19	4.4E-10	Adipose - Subcutaneous
ENSG00000105383.15	CD33	chr19_51217369_A_T_b38	rs1566576	0.14	4.5E-10	Nerve - Tibial
ENSG00000105383.15	CD33	chr19_51224066_A_G_b38	rs1697553	0.15	4.6E-10	Lung
ENSG00000105383.15	CD33	chr19_51224937_T_C_b38	rs2459141	0.14	4.8E-10	Nerve - Tibial
ENSG00000105383.15	CD33	chr19_51255314_A_T_b38	rs7256372	0.37	5E-10	Spleen
ENSG00000105383.15	CD33	chr19_51225385_A_G_b38	rs2455069	0.14	5.8E-10	Nerve - Tibial
ENSG00000105383.15	CD33	chr19_51222568_G_T_b38	rs273640	0.15	6E-10	Lung
ENSG00000105383.15	CD33	chr19_51256448_A_T_b38	rs1501448	0.18	6.5E-10	Adipose - Subcutaneous
ENSG00000105383.15	CD33	chr19_51225847_CCCGG_C_b38	rs201074739	-0.48	6.8E-10	Adipose - Visceral (Omentum)
ENSG00000105383.15	CD33	chr19_51215581_C_T_b38	rs273634	0.15	7.3E-10	Lung
ENSG00000105383.15	CD33	chr19_51243997_T_C_b38	rs73612300	0.24	7.9E-10	Adipose - Visceral (Omentum)
ENSG00000105383.15	CD33	chr19_51223099_G_C_b38	rs1399837	0.13	8.1E-10	Nerve - Tibial
ENSG00000105383.15	CD33	chr19_51217369_A_T_b38	rs1566576	0.15	0.000000001	Lung
ENSG00000105383.15	CD33	chr19_51225385_A_G_b38	rs2455069	0.15	0.000000001	Lung
ENSG00000105383.15	CD33	chr19_51225847_CCCGG_C_b38	rs201074739	-0.5	0.000000001	Lung
ENSG00000105383.15	CD33	chr19_51258097_G_C_b38	rs62115997	-0.45	0.000000001	Adipose - Visceral (Omentum)
ENSG00000105383.15	CD33	chr19_51258337_G_C_b38	rs62115998	-0.45	0.000000001	Adipose - Visceral (Omentum)
ENSG00000105383.15	CD33	chr19_51258360_G_A_b38	rs62115999	-0.45	0.000000001	Adipose - Visceral (Omentum)
ENSG00000105383.15	CD33	chr19_51214320_C_T_b38	rs4802772	0.17	1.1E-09	Adipose - Subcutaneous
ENSG00000105383.15	CD33	chr19_51224937_T_C_b38	rs2459141	0.15	1.1E-09	Lung
ENSG00000105383.15	CD33	chr19_51246242_C_T_b38	rs116953977	0.3	1.1E-09	Whole Blood
ENSG00000105383.15	CD33	chr19_51215061_T_A_b38	rs1697573	0.13	1.2E-09	Nerve - Tibial
ENSG00000105383.15	CD33	chr19_51215581_C_T_b38	rs273634	0.13	1.3E-09	Nerve - Tibial
ENSG00000105383.15	CD33	chr19_51219326_A_G_b38	rs12609179	0.13	1.3E-09	Nerve - Tibial
ENSG00000105383.15	CD33	chr19_51251278_A_C_b38	rs9676731	0.16	1.4E-09	Adipose - Subcutaneous
ENSG00000105383.15	CD33	chr19_51260244_C_T_b38	rs11668174	0.19	1.4E-09	Adipose - Subcutaneous
ENSG00000105383.15	CD33	chr19_51223099_G_C_b38	rs1399837	0.15	1.5E-09	Lung
ENSG00000105383.15	CD33	chr19_51240790_G_T_b38	rs8103085	0.22	1.9E-09	Artery - Tibial
ENSG00000105383.15	CD33	chr19_51206550_G_A_b38	rs142760794	-0.5	2.2E-09	Whole Blood
ENSG00000105383.15	CD33	chr19_51260103_T_C_b38	rs11672446	0.18	2.2E-09	Adipose - Subcutaneous
ENSG00000105383.15	CD33	chr19_51219326_A_G_b38	rs12609179	0.15	2.3E-09	Lung
ENSG00000105383.15	CD33	chr19_51220856_A_G_b38	rs273638	0.15	2.8E-09	Lung
ENSG00000105383.15	CD33	chr19_51221277_A_ACACAATATTTTAC_b38	rs11273989	0.15	2.8E-09	Lung
ENSG00000105383.15	CD33	chr19_51220906_A_G_b38	rs273639	0.15	2.9E-09	Lung
ENSG00000105383.15	CD33	chr19_51220906_A_G_b38	rs273639	0.13	2.9E-09	Nerve - Tibial
ENSG00000105383.15	CD33	chr19_51217369_A_T_b38	rs1566576	0.12	0.000000003	Artery - Tibial
ENSG00000105383.15	CD33	chr19_51223655_A_C_b38	rs1710398	0.12	0.000000003	Artery - Tibial
ENSG00000105383.15	CD33	chr19_51222568_G_T_b38	rs273640	0.13	3.2E-09	Artery - Tibial
ENSG00000105383.15	CD33	chr19_51206550_G_A_b38	rs142760794	-0.63	3.5E-09	Skin - Sun Exposed (Lower leg)
ENSG00000105383.15	CD33	chr19_51225385_A_G_b38	rs2455069	0.12	3.5E-09	Artery - Tibial
ENSG00000105383.15	CD33	chr19_51214320_C_T_b38	rs4802772	0.31	3.6E-09	Colon - Sigmoid
ENSG00000105383.15	CD33	chr19_51221277_A_ACACAATATTTTAC_b38	rs11273989	0.13	3.7E-09	Nerve - Tibial
ENSG00000105383.15	CD33	chr19_51220290_C_G_b38	rs273637	0.14	4.3E-09	Lung
ENSG00000105383.15	CD33	chr19_51224066_A_G_b38	rs1697553	0.12	4.3E-09	Artery - Tibial
ENSG00000105383.15	CD33	chr19_51243997_T_C_b38	rs73612300	0.31	4.3E-09	Spleen
ENSG00000105383.15	CD33	chr19_51215061_T_A_b38	rs1697573	0.12	4.4E-09	Artery - Tibial
ENSG00000105383.15	CD33	chr19_51256448_A_T_b38	rs1501448	0.34	4.4E-09	Spleen
ENSG00000105383.15	CD33	chr19_51225847_CCCGG_C_b38	rs201074739	-0.43	4.8E-09	Artery - Aorta
ENSG00000105383.15	CD33	chr19_51220856_A_G_b38	rs273638	0.13	5.5E-09	Nerve - Tibial
ENSG00000105383.15	CD33	chr19_51298886_T_C_b38	rs1710354	0.098	5.8E-09	Adipose - Subcutaneous
ENSG00000105383.15	CD33	chr19_51246334_C_T_b38	rs1566578	0.21	5.9E-09	Artery - Tibial
ENSG00000105383.15	CD33	chr19_51246958_G_A_b38	rs11084061	0.21	5.9E-09	Artery - Tibial
ENSG00000105383.15	CD33	chr19_51246983_T_C_b38	rs11084062	0.21	5.9E-09	Artery - Tibial
ENSG00000105383.15	CD33	chr19_51248679_C_A_b38	rs989502	0.21	5.9E-09	Artery - Tibial
ENSG00000105383.15	CD33	chr19_51248695_G_A_b38	rs989505	0.21	5.9E-09	Artery - Tibial
ENSG00000105383.15	CD33	chr19_51248696_C_A_b38	rs1319675	0.21	5.9E-09	Artery - Tibial
ENSG00000105383.15	CD33	chr19_51258097_G_C_b38	rs62115997	-0.45	6.2E-09	Skin - Sun Exposed (Lower leg)
ENSG00000105383.15	CD33	chr19_51258337_G_C_b38	rs62115998	-0.45	6.2E-09	Skin - Sun Exposed (Lower leg)
ENSG00000105383.15	CD33	chr19_51258360_G_A_b38	rs62115999	-0.45	6.2E-09	Skin - Sun Exposed (Lower leg)
ENSG00000105383.15	CD33	chr19_51213820_A_G_b38	rs2411329	-0.12	6.7E-09	Artery - Tibial
ENSG00000105383.15	CD33	chr19_51242324_T_G_b38	rs1034521	0.21	6.7E-09	Artery - Tibial
ENSG00000105383.15	CD33	chr19_51243667_G_A_b38	rs12461354	0.21	6.7E-09	Artery - Tibial
ENSG00000105383.15	CD33	chr19_51247502_T_C_b38	rs12461282	0.2	7.4E-09	Artery - Tibial
ENSG00000105383.15	CD33	chr19_51220290_C_G_b38	rs273637	0.13	8.1E-09	Nerve - Tibial
ENSG00000105383.15	CD33	chr19_51246242_C_T_b38	rs116953977	0.3	9.1E-09	Adipose - Subcutaneous
ENSG00000105383.15	CD33	chr19_51250875_G_T_b38	rs17801843	0.23	9.6E-09	Adipose - Visceral (Omentum)
ENSG00000105383.15	CD33	chr19_51224937_T_C_b38	rs2459141	0.12	9.7E-09	Artery - Tibial
ENSG00000105383.15	CD33	chr19_51240790_G_T_b38	rs8103085	0.22	9.9E-09	Nerve - Tibial
ENSG00000105383.15	CD33	chr19_51255314_A_T_b38	rs7256372	0.25	0.00000001	Adipose - Visceral (Omentum)
ENSG00000105383.15	CD33	chr19_51260244_C_T_b38	rs11668174	0.35	0.00000001	Spleen

ENSG00000105383.15	CD33	chr19_51219326_A_G_b38	rs12609179	0.12	0.000000011	Artery - Tibial
ENSG00000105383.15	CD33	chr19_51243997_T_C_b38	rs73612300	0.19	0.000000011	Artery - Tibial
ENSG00000105383.15	CD33	chr19_51247868_C_T_b38	rs11084063	0.35	0.000000011	Spleen
ENSG00000105383.15	CD33	chr19_51248395_G_T_b38	rs989504	0.35	0.000000011	Spleen
ENSG00000105383.15	CD33	chr19_51220290_C_G_b38	rs273637	0.12	0.000000012	Artery - Tibial
ENSG00000105383.15	CD33	chr19_51223099_G_C_b38	rs1399837	0.12	0.000000013	Artery - Tibial
ENSG00000105383.15	CD33	chr19_51233128_G_C_b38	rs33978622	-0.1	0.000000015	Whole Blood
ENSG00000105383.15	CD33	chr19_51256448_A_T_b38	rs1501448	0.24	0.000000015	Adipose - Visceral (Omentum)
ENSG00000105383.15	CD33	chr19_51220856_A_G_b38	rs273638	0.12	0.000000016	Artery - Tibial
ENSG00000105383.15	CD33	chr19_51220906_A_G_b38	rs273639	0.12	0.000000016	Artery - Tibial
ENSG00000105383.15	CD33	chr19_51221277_A_ACACAATATTTTAC_b38	rs11273989	0.12	0.000000016	Artery - Tibial
ENSG00000105383.15	CD33	chr19_51261577_C_T_b38	rs11084064	0.17	0.000000018	Adipose - Subcutaneous
ENSG00000105383.15	CD33	chr19_51220906_A_G_b38	rs273639	0.12	0.000000021	Artery - Aorta
ENSG00000105383.15	CD33	chr19_51239889_G_C_b38	rs1803254	0.19	0.000000021	Artery - Tibial
ENSG00000105383.15	CD33	chr19_51239889_G_C_b38	rs1803254	0.23	0.000000023	Adipose - Visceral (Omentum)
ENSG00000105383.15	CD33	chr19_51250704_G_C_b38	rs7253829	0.2	0.000000023	Artery - Tibial
ENSG00000105383.15	CD33	chr19_51250705_G_A_b38	rs7253831	0.2	0.000000023	Artery - Tibial
ENSG00000105383.15	CD33	chr19_51215061_T_A_b38	rs1697573	0.12	0.000000024	Artery - Aorta
ENSG00000105383.15	CD33	chr19_51223655_A_C_b38	rs1710398	0.11	0.000000024	Artery - Aorta
ENSG00000105383.15	CD33	chr19_51224066_A_G_b38	rs1697553	0.11	0.000000024	Artery - Aorta
ENSG00000105383.15	CD33	chr19_51222568_G_T_b38	rs273640	0.11	0.000000027	Artery - Aorta
ENSG00000105383.15	CD33	chr19_51334106_T_G_b38	rs11668576	-0.46	0.000000027	Artery - Tibial
ENSG00000105383.15	CD33	chr19_51215581_C_T_b38	rs273634	0.12	0.000000028	Artery - Tibial
ENSG00000105383.15	CD33	chr19_51220856_A_G_b38	rs273638	0.11	0.000000028	Artery - Aorta
ENSG00000105383.15	CD33	chr19_51227920_G_A_b38	rs7245846	-0.095	0.00000003	Whole Blood
ENSG00000105383.15	CD33	chr19_51221277_A_ACACAATATTTTAC_b38	rs11273989	0.11	0.000000031	Artery - Aorta
ENSG00000105383.15	CD33	chr19_51224706_C_A_b38	rs3865444	-0.1	0.000000031	Whole Blood
ENSG00000105383.15	CD33	chr19_51225221_C_T_b38	rs12459419	-0.1	0.000000031	Whole Blood
ENSG00000105383.15	CD33	chr19_51267038_G_A_b38	rs11668077	-0.34	0.000000031	Adipose - Subcutaneous
ENSG00000105383.15	CD33	chr19_51223099_G_C_b38	rs1399837	0.11	0.000000033	Artery - Aorta
ENSG00000105383.15	CD33	chr19_51260244_C_T_b38	rs11668174	0.25	0.000000033	Adipose - Visceral (Omentum)
ENSG00000105383.15	CD33	chr19_51217369_A_T_b38	rs1566576	0.11	0.000000037	Artery - Aorta
ENSG00000105383.15	CD33	chr19_51219326_A_G_b38	rs12609179	0.11	0.000000046	Artery - Aorta
ENSG00000105383.15	CD33	chr19_51225385_A_G_b38	rs2455069	0.11	0.000000046	Artery - Aorta
ENSG00000105383.15	CD33	chr19_51260103_T_C_b38	rs11672446	0.32	0.000000047	Spleen
ENSG00000105383.15	CD33	chr19_51246334_C_T_b38	rs1566578	0.2	0.000000049	Nerve - Tibial
ENSG00000105383.15	CD33	chr19_51246958_G_A_b38	rs11084061	0.2	0.000000049	Nerve - Tibial
ENSG00000105383.15	CD33	chr19_51246983_T_C_b38	rs11084062	0.2	0.000000049	Nerve - Tibial
ENSG00000105383.15	CD33	chr19_51248679_C_A_b38	rs989502	0.2	0.000000049	Nerve - Tibial
ENSG00000105383.15	CD33	chr19_51248695_G_A_b38	rs989505	0.2	0.000000049	Nerve - Tibial
ENSG00000105383.15	CD33	chr19_51248696_C_A_b38	rs1319675	0.2	0.000000049	Nerve - Tibial
ENSG00000105383.15	CD33	chr19_51220290_C_G_b38	rs273637	0.11	0.000000052	Artery - Aorta
ENSG00000105383.15	CD33	chr19_51250704_G_C_b38	rs7253829	0.2	0.000000053	Nerve - Tibial
ENSG00000105383.15	CD33	chr19_51250705_G_A_b38	rs7253831	0.2	0.000000053	Nerve - Tibial
ENSG00000105383.15	CD33	chr19_51259327_T_C_b38	rs10412123	0.15	0.000000053	Adipose - Subcutaneous
ENSG00000105383.15	CD33	chr19_51215581_C_T_b38	rs273634	0.11	0.000000054	Artery - Aorta
ENSG00000105383.15	CD33	chr19_51242324_T_G_b38	rs1034521	0.2	0.000000058	Nerve - Tibial
ENSG00000105383.15	CD33	chr19_51243667_G_A_b38	rs12461354	0.2	0.000000058	Nerve - Tibial
ENSG00000105383.15	CD33	chr19_51247502_T_C_b38	rs12461282	0.2	0.000000063	Nerve - Tibial
ENSG00000105383.15	CD33	chr19_51250875_G_T_b38	rs17801843	0.18	0.000000063	Artery - Tibial
ENSG00000105383.15	CD33	chr19_51255390_A_T_b38	rs7256512	0.15	0.000000063	Adipose - Subcutaneous
ENSG00000105383.15	CD33	chr19_51247868_C_T_b38	rs11084063	0.24	0.00000007	Adipose - Visceral (Omentum)
ENSG00000105383.15	CD33	chr19_51248395_G_T_b38	rs989504	0.24	0.000000074	Adipose - Visceral (Omentum)
ENSG00000105383.15	CD33	chr19_51224937_T_C_b38	rs2459141	0.11	0.000000075	Artery - Aorta
ENSG00000105383.15	CD33	chr19_51262616_A_T_b38	rs10412214	0.16	0.000000094	Adipose - Subcutaneous
ENSG00000105383.15	CD33	chr19_51246242_C_T_b38	rs116953977	0.34	0.00000011	Adipose - Visceral (Omentum)
ENSG00000105383.15	CD33	chr19_51210109_G_A_b38	rs12985029	-0.12	0.00000012	Nerve - Tibial
ENSG00000105383.15	CD33	chr19_51225847_CCCGG_C_b38	rs201074739	-0.7	0.00000012	Spleen
ENSG00000105383.15	CD33	chr19_51239889_G_C_b38	rs1803254	0.19	0.00000012	Nerve - Tibial
ENSG00000105383.15	CD33	chr19_51261577_C_T_b38	rs11084064	0.31	0.00000012	Spleen
ENSG00000105383.15	CD33	chr19_51223099_G_C_b38	rs1399837	0.12	0.00000013	Adipose - Visceral (Omentum)
ENSG00000105383.15	CD33	chr19_51210809_G_A_b38	rs3852865	-0.12	0.00000014	Nerve - Tibial
ENSG00000105383.15	CD33	chr19_51239889_G_C_b38	rs1803254	0.24	0.00000014	Colon - Transverse
ENSG00000105383.15	CD33	chr19_51217988_G_A_b38	rs79254320	0.23	0.00000015	Adipose - Subcutaneous
ENSG00000105383.15	CD33	chr19_51251278_A_C_b38	rs9676731	0.17	0.00000016	Artery - Tibial
ENSG00000105383.15	CD33	chr19_51334106_T_G_b38	rs11668576	-0.35	0.00000017	Adipose - Subcutaneous
ENSG00000105383.15	CD33	chr19_51225847_CCCGG_C_b38	rs201074739	-0.47	0.00000023	Skin - Not Sun Exposed (Suprapubic)
ENSG00000105383.15	CD33	chr19_51246242_C_T_b38	rs116953977	0.31	0.00000023	Artery - Tibial
ENSG00000105383.15	CD33	chr19_51262272_G_A_b38	rs10406897	0.16	0.00000023	Adipose - Subcutaneous
ENSG00000105383.15	CD33	chr19_51233866_A_G_b38	rs34813869	-0.091	0.00000024	Whole Blood
ENSG00000105383.15	CD33	chr19_51217988_G_A_b38	rs79254320	0.29	0.00000029	Adipose - Visceral (Omentum)
ENSG00000105383.15	CD33	chr19_51267038_G_A_b38	rs11668077	-0.46	0.0000003	Breast - Mammary Tissue
ENSG00000105383.15	CD33	chr19_51206550_G_A_b38	rs142760794	-0.35	0.00000032	Adipose - Subcutaneous
ENSG00000105383.15	CD33	chr19_51215581_C_T_b38	rs273634	0.12	0.00000032	Adipose - Visceral (Omentum)
ENSG00000105383.15	CD33	chr19_51234736_T_G_b38	rs1354106	-0.088	0.00000035	Whole Blood
ENSG00000105383.15	CD33	chr19_51219326_A_G_b38	rs12609179	0.12	0.00000038	Adipose - Visceral (Omentum)
ENSG00000105383.15	CD33	chr19_51210809_G_A_b38	rs3852865	-0.11	0.0000004	Artery - Tibial
ENSG00000105383.15	CD33	chr19_51258097_G_C_b38	rs62115997	-0.41	0.00000042	Lung
ENSG00000105383.15	CD33	chr19_51258337_G_C_b38	rs62115998	-0.41	0.00000042	Lung
ENSG00000105383.15	CD33	chr19_51258360_G_A_b38	rs62115999	-0.41	0.00000042	Lung

ENSG00000105383.15	CD33	chr19_51262616_A_T_b38	rs10412214	0.29	0.00000047	Spleen
ENSG00000105383.15	CD33	chr19_51298886_T_C_b38	rs1710354	0.1	0.00000047	Artery - Aorta
ENSG00000105383.15	CD33	chr19_51215061_T_A_b38	rs1697573	0.12	0.00000054	Adipose - Visceral (Omentum)
ENSG00000105383.15	CD33	chr19_51224937_T_C_b38	rs2459141	0.12	0.00000054	Adipose - Visceral (Omentum)
ENSG00000105383.15	CD33	chr19_51214320_C_T_b38	rs4802772	0.21	0.00000058	Adipose - Visceral (Omentum)
ENSG00000105383.15	CD33	chr19_51210109_G_A_b38	rs12985029	-0.11	0.00000059	Artery - Tibial
ENSG00000105383.15	CD33	chr19_51262272_G_A_b38	rs10406897	0.29	0.00000062	Spleen
ENSG00000105383.15	CD33	chr19_51187836_C_T_b38	rs7255837	0.092	0.00000063	Whole Blood
ENSG00000105383.15	CD33	chr19_51268161_T_C_b38	rs17716016	-0.21	0.00000067	Brain - Caudate (basal ganglia)
ENSG00000105383.15	CD33	chr19_51267038_G_A_b38	rs11668077	-0.43	0.00000069	Adipose - Visceral (Omentum)
ENSG00000105383.15	CD33	chr19_51206550_G_A_b38	rs142760794	-0.42	0.00000073	Nerve - Tibial
ENSG00000105383.15	CD33	chr19_51222568_G_T_b38	rs273640	0.12	0.00000073	Adipose - Visceral (Omentum)
ENSG00000105383.15	CD33	chr19_51262872_C_CTATG_b38		0.14	0.00000073	Adipose - Subcutaneous
ENSG00000105383.15	CD33	chr19_51225385_A_G_b38	rs2455069	0.12	0.00000078	Adipose - Visceral (Omentum)
ENSG00000105383.15	CD33	chr19_51267038_G_A_b38	rs11668077	-0.45	0.00000079	Skin - Sun Exposed (Lower leg)
ENSG00000105383.15	CD33	chr19_51223655_A_C_b38	rs1710398	0.12	0.0000008	Adipose - Visceral (Omentum)
ENSG00000105383.15	CD33	chr19_51224066_A_G_b38	rs1697553	0.12	0.0000008	Adipose - Visceral (Omentum)
ENSG00000105383.15	CD33	chr19_51235662_G_A_b38	rs35112940	-0.13	0.0000008	Nerve - Tibial
ENSG00000105383.15	CD33	chr19_51298607_T_G_b38	rs1710355	0.083	0.00000083	Adipose - Subcutaneous
ENSG00000105383.15	CD33	chr19_51250875_G_T_b38	rs17801843	0.17	0.00000088	Nerve - Tibial
ENSG00000105383.15	CD33	chr19_51270875_C_A_b38	rs12608749	-0.21	0.00000089	Brain - Caudate (basal ganglia)
ENSG00000105383.15	CD33	chr19_51220906_A_G_b38	rs273639	0.12	0.00000092	Adipose - Visceral (Omentum)
ENSG00000105383.15	CD33	chr19_51256870_A_T_b38	rs1501449	0.13	0.00000097	Adipose - Subcutaneous
ENSG00000105383.15	CD33	chr19_51175173_G_A_b38	rs2134068	0.1	0.00000098	Whole Blood
ENSG00000105383.15	CD33	chr19_51225847_CCCGG_C_b38	rs201074739	-0.47	0.00000099	Colon - Transverse
ENSG00000105383.15	CD33	chr19_51183354_A_G_b38	rs11666927	0.09	0.0000011	Whole Blood
ENSG00000105383.15	CD33	chr19_51217369_A_T_b38	rs1566576	0.11	0.0000011	Adipose - Visceral (Omentum)
ENSG00000105383.15	CD33	chr19_51251278_A_C_b38	rs9676731	0.18	0.0000011	Adipose - Visceral (Omentum)
ENSG00000105383.15	CD33	chr19_51256436_A_G_b38	rs1501447	0.19	0.0000011	Adipose - Visceral (Omentum)
ENSG00000105383.15	CD33	chr19_51261577_C_T_b38	rs11084064	0.2	0.0000011	Adipose - Visceral (Omentum)
ENSG00000105383.15	CD33	chr19_51210270_C_G_b38	rs12985109	-0.18	0.0000012	Nerve - Tibial
ENSG00000105383.15	CD33	chr19_51210300_A_T_b38	rs12984892	-0.18	0.0000012	Nerve - Tibial
ENSG00000105383.15	CD33	chr19_51260103_T_C_b38	rs11672446	0.21	0.0000012	Adipose - Visceral (Omentum)
ENSG00000105383.15	CD33	chr19_51185832_T_C_b38	rs10405784	0.09	0.0000013	Whole Blood
ENSG00000105383.15	CD33	chr19_51270938_A_G_b38	rs12608738	-0.21	0.0000013	Brain - Caudate (basal ganglia)
ENSG00000105383.15	CD33	chr19_51256271_T_C_b38	rs10402797	0.13	0.0000013	Adipose - Subcutaneous
ENSG00000105383.15	CD33	chr19_51192887_G_A_b38	rs7254889	0.089	0.0000014	Whole Blood
ENSG00000105383.15	CD33	chr19_51192888_G_T_b38	rs7254890	0.089	0.0000014	Whole Blood
ENSG00000105383.15	CD33	chr19_51213820_A_G_b38	rs2411329	-0.1	0.0000014	Nerve - Tibial
ENSG00000105383.15	CD33	chr19_51243997_T_C_b38	rs73612300	0.18	0.0000014	Breast - Mammary Tissue
ENSG00000105383.15	CD33	chr19_51262272_G_A_b38	rs10406897	0.21	0.0000014	Adipose - Visceral (Omentum)
ENSG00000105383.15	CD33	chr19_51275698_G_C_b38	rs12975233	-0.2	0.0000015	Brain - Caudate (basal ganglia)
ENSG00000105383.15	CD33	chr19_51240790_G_T_b38	rs8103085	0.28	0.0000015	Colon - Sigmoid
ENSG00000105383.15	CD33	chr19_51246242_C_T_b38	rs116953977	0.41	0.0000016	Colon - Sigmoid
ENSG00000105383.15	CD33	chr19_51240790_G_T_b38	rs8103085	0.55	0.0000017	Small Intestine - Terminal Ileum
ENSG00000105383.15	CD33	chr19_51185948_A_C_b38	rs10404245	0.089	0.0000018	Whole Blood
ENSG00000105383.15	CD33	chr19_51240790_G_T_b38	rs8103085	0.23	0.0000018	Colon - Transverse
ENSG00000105383.15	CD33	chr19_51256448_A_T_b38	rs1501448	0.29	0.0000018	Colon - Sigmoid
ENSG00000105383.15	CD33	chr19_51255314_A_T_b38	rs7256372	0.3	0.0000019	Colon - Sigmoid
ENSG00000105383.15	CD33	chr19_51184343_A_G_b38	rs10416576	0.088	0.000002	Whole Blood
ENSG00000105383.15	CD33	chr19_51185586_G_C_b38	rs10404091	0.088	0.000002	Whole Blood
ENSG00000105383.15	CD33	chr19_51220856_A_G_b38	rs273638	0.11	0.000002	Adipose - Visceral (Omentum)
ENSG00000105383.15	CD33	chr19_51221277_A_ACACAATATTTTAC_b38	rs11273989	0.11	0.000002	Adipose - Visceral (Omentum)
ENSG00000105383.15	CD33	chr19_51271552_C_T_b38	rs10408324	-0.2	0.000002	Brain - Caudate (basal ganglia)
ENSG00000105383.15	CD33	chr19_51271359_G_A_b38	rs7249595	-0.2	0.000002	Brain - Caudate (basal ganglia)
ENSG00000105383.15	CD33	chr19_51271142_T_G_b38	rs7249916	-0.2	0.000002	Brain - Caudate (basal ganglia)
ENSG00000105383.15	CD33	chr19_51225847_CCCGG_C_b38	rs201074739	-0.57	0.000002	Brain - Spinal cord (cervical c-1)
ENSG00000105383.15	CD33	chr19_51256436_A_G_b38	rs1501447	0.16	0.000002	Artery - Tibial
ENSG00000105383.15	CD33	chr19_51176765_T_TCA_b38	rs59548084	0.086	0.0000021	Whole Blood
ENSG00000105383.15	CD33	chr19_51272277_G_A_b38	rs12972826	-0.19	0.0000021	Brain - Caudate (basal ganglia)
ENSG00000105383.15	CD33	chr19_51272460_C_G_b38	rs12972888	-0.19	0.0000021	Brain - Caudate (basal ganglia)
ENSG00000105383.15	CD33	chr19_51272484_C_T_b38	rs12972903	-0.19	0.0000021	Brain - Caudate (basal ganglia)
ENSG00000105383.15	CD33	chr19_51272126_T_C_b38	rs34589735	-0.19	0.0000021	Brain - Caudate (basal ganglia)
ENSG00000105383.15	CD33	chr19_51272908_C_T_b38	rs8110859	-0.19	0.0000021	Brain - Caudate (basal ganglia)
ENSG00000105383.15	CD33	chr19_51272863_G_T_b38	rs8111074	-0.19	0.0000021	Brain - Caudate (basal ganglia)
ENSG00000105383.15	CD33	chr19_51272975_G_A_b38	rs8111217	-0.19	0.0000021	Brain - Caudate (basal ganglia)
ENSG00000105383.15	CD33	chr19_51272622_T_C_b38	rs8111393	-0.19	0.0000021	Brain - Caudate (basal ganglia)
ENSG00000105383.15	CD33	chr19_51272883_T_G_b38	rs8111753	-0.19	0.0000021	Brain - Caudate (basal ganglia)
ENSG00000105383.15	CD33	chr19_51272291_GT_G_b38		-0.19	0.0000021	Brain - Caudate (basal ganglia)
ENSG00000105383.15	CD33	chr19_51246242_C_T_b38	rs116953977	0.34	0.0000021	Lung
ENSG00000105383.15	CD33	chr19_51248395_G_T_b38	rs989504	0.18	0.0000021	Artery - Tibial
ENSG00000105383.15	CD33	chr19_51175173_G_A_b38	rs2134068	0.12	0.0000022	Nerve - Tibial
ENSG00000105383.15	CD33	chr19_51225847_CCCGG_C_b38	rs201074739	-0.45	0.0000022	Esophagus - Mucosa
ENSG00000105383.15	CD33	chr19_51243997_T_C_b38	rs73612300	0.17	0.0000022	Nerve - Tibial
ENSG00000105383.15	CD33	chr19_51174413_T_C_b38	rs4802771	0.089	0.0000023	Whole Blood
ENSG00000105383.15	CD33	chr19_51225847_CCCGG_C_b38	rs201074739	-0.57	0.0000023	Pancreas
ENSG00000105383.15	CD33	chr19_51188393_T_C_b38	rs10421852	0.088	0.0000024	Whole Blood
ENSG00000105383.15	CD33	chr19_51246788_T_C_b38	rs145323276	0.18	0.0000024	Adipose - Subcutaneous
ENSG00000105383.15	CD33	chr19_51185059_C_T_b38	rs16982535	0.087	0.0000025	Whole Blood
ENSG00000105383.15	CD33	chr19_51225385_A_G_b38	rs2455069	0.14	0.0000025	Brain - Spinal cord (cervical c-1)

ENSG00000105383.15	CD33	chr19_51184466_C_CG_b38		0.087	0.0000026	Whole Blood
ENSG00000105383.15	CD33	chr19_51193473_G_A_b38	rs7255660	0.089	0.0000026	Whole Blood
ENSG00000105383.15	CD33	chr19_51239889_G_C_b38	rs1803254	0.19	0.0000027	Esophagus - Muscularis
ENSG00000105383.15	CD33	chr19_51242324_T_G_b38	rs1034521	0.19	0.0000027	Esophagus - Muscularis
ENSG00000105383.15	CD33	chr19_51243667_G_A_b38	rs12461354	0.19	0.0000027	Esophagus - Muscularis
ENSG00000105383.15	CD33	chr19_51246334_C_T_b38	rs1566578	0.19	0.0000027	Esophagus - Muscularis
ENSG00000105383.15	CD33	chr19_51246958_G_A_b38	rs11084061	0.19	0.0000027	Esophagus - Muscularis
ENSG00000105383.15	CD33	chr19_51246983_T_C_b38	rs11084062	0.19	0.0000027	Esophagus - Muscularis
ENSG00000105383.15	CD33	chr19_51248679_C_A_b38	rs989502	0.19	0.0000027	Esophagus - Muscularis
ENSG00000105383.15	CD33	chr19_51248695_G_A_b38	rs989505	0.19	0.0000027	Esophagus - Muscularis
ENSG00000105383.15	CD33	chr19_51248696_C_A_b38	rs1319675	0.19	0.0000027	Esophagus - Muscularis
ENSG00000105383.15	CD33	chr19_51250704_G_C_b38	rs7253829	0.19	0.0000027	Esophagus - Muscularis
ENSG00000105383.15	CD33	chr19_51250705_G_A_b38	rs7253831	0.19	0.0000027	Esophagus - Muscularis
ENSG00000105383.15	CD33	chr19_51210809_G_A_b38	rs3852865	-0.087	0.0000028	Whole Blood
ENSG00000105383.15	CD33	chr19_51235662_G_A_b38	rs35112940	-0.097	0.0000029	Whole Blood
ENSG00000105383.15	CD33	chr19_51258097_G_C_b38	rs62115997	-0.34	0.0000029	Thyroid
ENSG00000105383.15	CD33	chr19_51258337_G_C_b38	rs62115998	-0.34	0.0000029	Thyroid
ENSG00000105383.15	CD33	chr19_51258360_G_A_b38	rs62115999	-0.34	0.0000029	Thyroid
ENSG00000105383.15	CD33	chr19_51177310_T_C_b38	rs11670380	0.084	0.000003	Whole Blood
ENSG00000105383.15	CD33	chr19_51186850_T_C_b38	rs34186776	0.11	0.000003	Nerve - Tibial
ENSG00000105383.15	CD33	chr19_51276338_T_C_b38	rs1399838	-0.19	0.0000031	Brain - Caudate (basal ganglia)
ENSG00000105383.15	CD33	chr19_51276433_T_G_b38	rs2898694	-0.19	0.0000031	Brain - Caudate (basal ganglia)
ENSG00000105383.15	CD33	chr19_51235662_G_A_b38	rs35112940	-0.11	0.0000031	Artery - Aorta
ENSG00000105383.15	CD33	chr19_51247868_C_T_b38	rs11084063	0.18	0.0000032	Artery - Tibial
ENSG00000105383.15	CD33	chr19_51194930_C_T_b38	rs10405709	0.086	0.0000033	Whole Blood
ENSG00000105383.15	CD33	chr19_51240790_G_T_b38	rs8103085	0.2	0.0000033	Esophagus - Muscularis
ENSG00000105383.15	CD33	chr19_51183449_G_T_b38	rs11667023	0.084	0.0000035	Adipose - Subcutaneous
ENSG00000105383.15	CD33	chr19_51215061_T_A_b38	rs1697573	0.081	0.0000036	Adipose - Subcutaneous
ENSG00000105383.15	CD33	chr19_51273503_A_G_b38	rs59526361	-0.19	0.0000036	Brain - Caudate (basal ganglia)
ENSG00000105383.15	CD33	chr19_50530131_G_A_b38	rs1150928	0.35	0.0000037	Brain - Putamen (basal ganglia)
ENSG00000105383.15	CD33	chr19_51175173_G_A_b38	rs2134068	0.16	0.0000037	Colon - Sigmoid
ENSG00000105383.15	CD33	chr19_51247502_T_C_b38	rs12461282	0.17	0.0000037	Artery - Aorta
ENSG00000105383.15	CD33	chr19_51298886_T_C_b38	rs1710354	0.082	0.0000037	Whole Blood
ENSG00000105383.15	CD33	chr19_51258097_G_C_b38	rs62115997	-0.62	0.0000038	Brain - Caudate (basal ganglia)
ENSG00000105383.15	CD33	chr19_51258337_G_C_b38	rs62115998	-0.62	0.0000038	Brain - Caudate (basal ganglia)
ENSG00000105383.15	CD33	chr19_51258360_G_A_b38	rs62115999	-0.62	0.0000038	Brain - Caudate (basal ganglia)
ENSG00000105383.15	CD33	chr19_51224937_T_C_b38	rs2459141	0.14	0.0000038	Brain - Spinal cord (cervical c-1)
ENSG00000105383.15	CD33	chr19_51298886_T_C_b38	rs1710354	0.1	0.0000038	Artery - Tibial
ENSG00000105383.15	CD33	chr19_51189214_T_C_b38	rs34528093	0.09	0.0000039	Adipose - Subcutaneous
ENSG00000105383.15	CD33	chr19_51187448_T_C_b38	rs35203095	0.089	0.000004	Whole Blood
ENSG00000105383.15	CD33	chr19_51210109_G_A_b38	rs12985029	-0.085	0.000004	Whole Blood
ENSG00000105383.15	CD33	chr19_51247502_T_C_b38	rs12461282	0.19	0.000004	Esophagus - Muscularis
ENSG00000105383.15	CD33	chr19_51188393_T_C_b38	rs10421852	0.11	0.0000041	Nerve - Tibial
ENSG00000105383.15	CD33	chr19_51190844_A_G_b38	rs7245847	0.11	0.0000041	Nerve - Tibial
ENSG00000105383.15	CD33	chr19_51271284_A_G_b38	rs7249149	-0.19	0.0000041	Brain - Caudate (basal ganglia)
ENSG00000105383.15	CD33	chr19_51255390_A_T_b38	rs7256512	0.15	0.0000041	Artery - Tibial
ENSG00000105383.15	CD33	chr19_51256870_A_T_b38	rs1501449	0.18	0.0000041	Adipose - Visceral (Omentum)
ENSG00000105383.15	CD33	chr19_51258097_G_C_b38	rs62115997	-0.49	0.0000041	Pancreas
ENSG00000105383.15	CD33	chr19_51258337_G_C_b38	rs62115998	-0.49	0.0000041	Pancreas
ENSG00000105383.15	CD33	chr19_51258360_G_A_b38	rs62115999	-0.49	0.0000041	Pancreas
ENSG00000105383.15	CD33	chr19_51255314_A_T_b38	rs7256372	0.18	0.0000042	Artery - Tibial
ENSG00000105383.15	CD33	chr19_51178709_C_A_b38	rs17715691	0.087	0.0000044	Adipose - Subcutaneous
ENSG00000105383.15	CD33	chr19_51185559_T_G_b38	rs59266985	0.09	0.0000044	Whole Blood
ENSG00000105383.15	CD33	chr19_51192221_T_C_b38	rs10411928	0.085	0.0000044	Whole Blood
ENSG00000105383.15	CD33	chr19_51192221_T_C_b38	rs10411928	0.1	0.0000045	Nerve - Tibial
ENSG00000105383.15	CD33	chr19_51214320_C_T_b38	rs4802772	0.16	0.0000047	Artery - Tibial
ENSG00000105383.15	CD33	chr19_51250704_G_C_b38	rs7253829	0.16	0.0000047	Artery - Aorta
ENSG00000105383.15	CD33	chr19_51250705_G_A_b38	rs7253831	0.16	0.0000047	Artery - Aorta
ENSG00000105383.15	CD33	chr19_51216221_C_CGT_b38	rs1491213119	0.096	0.0000048	Whole Blood
ENSG00000105383.15	CD33	chr19_51192887_G_A_b38	rs7254889	0.1	0.0000049	Nerve - Tibial
ENSG00000105383.15	CD33	chr19_51192888_G_T_b38	rs7254890	0.1	0.0000049	Nerve - Tibial
ENSG00000105383.15	CD33	chr19_51201783_T_C_b38	rs7256201	-0.1	0.0000049	Artery - Tibial
ENSG00000105383.15	CD33	chr19_51219326_A_G_b38	rs12609179	0.14	0.0000049	Brain - Spinal cord (cervical c-1)
ENSG00000105383.15	CD33	chr19_51215581_C_T_b38	rs273634	0.14	0.000005	Brain - Spinal cord (cervical c-1)
ENSG00000105383.15	CD33	chr19_51193216_A_G_b38	rs11084055	0.087	0.0000051	Whole Blood
ENSG00000105383.15	CD33	chr19_51213820_A_G_b38	rs2411329	-0.081	0.0000051	Whole Blood
ENSG00000105383.15	CD33	chr19_51189214_T_C_b38	rs34528093	0.092	0.0000052	Whole Blood
ENSG00000105383.15	CD33	chr19_51206550_G_A_b38	rs142760794	-0.44	0.0000052	Breast - Mammary Tissue
ENSG00000105383.15	CD33	chr19_51217369_A_T_b38	rs1566576	0.14	0.0000052	Brain - Spinal cord (cervical c-1)
ENSG00000105383.15	CD33	chr19_51258097_G_C_b38	rs62115997	-0.37	0.0000053	Breast - Mammary Tissue
ENSG00000105383.15	CD33	chr19_51258337_G_C_b38	rs62115998	-0.37	0.0000053	Breast - Mammary Tissue
ENSG00000105383.15	CD33	chr19_51258360_G_A_b38	rs62115999	-0.37	0.0000053	Breast - Mammary Tissue
ENSG00000105383.15	CD33	chr19_51190234_G_A_b38	rs9973300	0.087	0.0000054	Whole Blood
ENSG00000105383.15	CD33	chr19_51277180_C_A_b38	rs10425711	-0.19	0.0000054	Brain - Caudate (basal ganglia)
ENSG00000105383.15	CD33	chr19_51276807_T_G_b38	rs8113446	-0.18	0.0000054	Brain - Caudate (basal ganglia)
ENSG00000105383.15	CD33	chr19_51247868_C_T_b38	rs11084063	0.29	0.0000054	Colon - Sigmoid
ENSG00000105383.15	CD33	chr19_51258097_G_C_b38	rs62115997	-0.31	0.0000054	Artery - Aorta
ENSG00000105383.15	CD33	chr19_51258337_G_C_b38	rs62115998	-0.31	0.0000054	Artery - Aorta
ENSG00000105383.15	CD33	chr19_51258360_G_A_b38	rs62115999	-0.31	0.0000054	Artery - Aorta
ENSG00000105383.15	CD33	chr19_51186703_C_T_b38	rs67836621	0.11	0.0000055	Nerve - Tibial

ENSG00000105383.15	CD33	chr19_51186912_T_A_b38	rs35469086	0.11	0.0000055	Nerve - Tibial
ENSG00000105383.15	CD33	chr19_51187448_T_C_b38	rs35203095	0.11	0.0000055	Nerve - Tibial
ENSG00000105383.15	CD33	chr19_51220290_C_G_b38	rs273637	0.11	0.0000055	Adipose - Visceral (Omentum)
ENSG00000105383.15	CD33	chr19_51233128_G_C_b38	rs33978622	-0.11	0.0000055	Nerve - Tibial
ENSG00000105383.15	CD33	chr19_51250875_G_T_b38	rs17801843	0.2	0.0000056	Colon - Transverse
ENSG00000105383.15	CD33	chr19_51174413_T_C_b38	rs4802771	0.1	0.0000058	Nerve - Tibial
ENSG00000105383.15	CD33	chr19_51266878_G_C_b38	rs10416845	-0.18	0.0000058	Brain - Caudate (basal ganglia)
ENSG00000105383.15	CD33	chr19_51268030_G_A_b38	rs7258998	-0.18	0.0000058	Brain - Caudate (basal ganglia)
ENSG00000105383.15	CD33	chr19_51194930_C_T_b38	rs10405709	0.1	0.0000059	Nerve - Tibial
ENSG00000105383.15	CD33	chr19_51198635_A_G_b38	rs10421385	0.1	0.0000059	Nerve - Tibial
ENSG00000105383.15	CD33	chr19_51177310_T_C_b38	rs11670380	0.1	0.000006	Nerve - Tibial
ENSG00000105383.15	CD33	chr19_51215061_T_A_b38	rs1697573	0.14	0.0000061	Brain - Spinal cord (cervical c-1)
ENSG00000105383.15	CD33	chr19_51235662_G_A_b38	rs35112940	-0.21	0.0000061	Brain - Caudate (basal ganglia)
ENSG00000105383.15	CD33	chr19_51243997_T_C_b38	rs73612300	0.18	0.0000061	Esophagus - Muscularis
ENSG00000105383.15	CD33	chr19_51242324_T_G_b38	rs1034521	0.16	0.0000062	Artery - Aorta
ENSG00000105383.15	CD33	chr19_51243667_G_A_b38	rs12461354	0.16	0.0000062	Artery - Aorta
ENSG00000105383.15	CD33	chr19_51246334_C_T_b38	rs1566578	0.16	0.0000062	Artery - Aorta
ENSG00000105383.15	CD33	chr19_51246958_G_A_b38	rs11084061	0.16	0.0000062	Artery - Aorta
ENSG00000105383.15	CD33	chr19_51246983_T_C_b38	rs11084062	0.16	0.0000062	Artery - Aorta
ENSG00000105383.15	CD33	chr19_51248679_C_A_b38	rs989502	0.16	0.0000062	Artery - Aorta
ENSG00000105383.15	CD33	chr19_51248695_G_A_b38	rs989505	0.16	0.0000062	Artery - Aorta
ENSG00000105383.15	CD33	chr19_51248696_C_A_b38	rs1319675	0.16	0.0000062	Artery - Aorta
ENSG00000105383.15	CD33	chr19_51221277_A_ACACAATATTTTAC_b38	rs11273989	0.079	0.0000064	Adipose - Subcutaneous
ENSG00000105383.15	CD33	chr19_51242324_T_G_b38	rs1034521	0.26	0.0000064	Colon - Sigmoid
ENSG00000105383.15	CD33	chr19_51243667_G_A_b38	rs12461354	0.26	0.0000064	Colon - Sigmoid
ENSG00000105383.15	CD33	chr19_51267038_G_A_b38	rs11668077	-0.42	0.0000064	Lung
ENSG00000105383.15	CD33	chr19_51183449_G_T_b38	rs11667023	0.085	0.0000065	Whole Blood
ENSG00000105383.15	CD33	chr19_51186703_C_T_b38	rs67836621	0.087	0.0000065	Whole Blood
ENSG00000105383.15	CD33	chr19_51186850_T_C_b38	rs34186776	0.086	0.0000065	Whole Blood
ENSG00000105383.15	CD33	chr19_51186912_T_A_b38	rs35469086	0.087	0.0000065	Whole Blood
ENSG00000105383.15	CD33	chr19_51190844_A_G_b38	rs7245847	0.084	0.0000065	Whole Blood
ENSG00000105383.15	CD33	chr19_51270257_C_A_b38	rs28536511	-0.19	0.0000065	Brain - Caudate (basal ganglia)
ENSG00000105383.15	CD33	chr19_51240790_G_T_b38	rs8103085	0.16	0.0000065	Artery - Aorta
ENSG00000105383.15	CD33	chr19_51246334_C_T_b38	rs1566578	0.26	0.0000065	Colon - Sigmoid
ENSG00000105383.15	CD33	chr19_51246958_G_A_b38	rs11084061	0.26	0.0000065	Colon - Sigmoid
ENSG00000105383.15	CD33	chr19_51246983_T_C_b38	rs11084062	0.26	0.0000065	Colon - Sigmoid
ENSG00000105383.15	CD33	chr19_51248679_C_A_b38	rs989502	0.26	0.0000065	Colon - Sigmoid
ENSG00000105383.15	CD33	chr19_51248695_G_A_b38	rs989505	0.26	0.0000065	Colon - Sigmoid
ENSG00000105383.15	CD33	chr19_51248696_C_A_b38	rs1319675	0.26	0.0000065	Colon - Sigmoid
ENSG00000105383.15	CD33	chr19_51190234_G_A_b38	rs9973300	0.11	0.0000066	Nerve - Tibial
ENSG00000105383.15	CD33	chr19_51239889_G_C_b38	rs1803254	0.25	0.0000066	Colon - Sigmoid
ENSG00000105383.15	CD33	chr19_51187836_C_T_b38	rs7255837	0.1	0.0000067	Nerve - Tibial
ENSG00000105383.15	CD33	chr19_51225847_CCCGG_C_b38	rs201074739	-0.36	0.0000067	Thyroid
ENSG00000105383.15	CD33	chr19_51222568_G_T_b38	rs273640	0.078	0.0000068	Adipose - Subcutaneous
ENSG00000105383.15	CD33	chr19_51222568_G_T_b38	rs273640	0.13	0.0000071	Brain - Spinal cord (cervical c-1)
ENSG00000105383.15	CD33	chr19_51223655_A_C_b38	rs1710398	0.13	0.0000071	Brain - Spinal cord (cervical c-1)
ENSG00000105383.15	CD33	chr19_51224066_A_G_b38	rs1697553	0.13	0.0000071	Brain - Spinal cord (cervical c-1)
ENSG00000105383.15	CD33	chr19_51224706_C_A_b38	rs3865444	-0.11	0.0000072	Nerve - Tibial
ENSG00000105383.15	CD33	chr19_51225221_C_T_b38	rs12459419	-0.11	0.0000072	Nerve - Tibial
ENSG00000105383.15	CD33	chr19_51178709_C_A_b38	rs17715691	0.11	0.0000073	Nerve - Tibial
ENSG00000105383.15	CD33	chr19_51298607_T_G_b38	rs1710355	0.079	0.0000073	Whole Blood
ENSG00000105383.15	CD33	chr19_51242324_T_G_b38	rs1034521	0.5	0.0000074	Small Intestine - Terminal Ileum
ENSG00000105383.15	CD33	chr19_51243667_G_A_b38	rs12461354	0.5	0.0000074	Small Intestine - Terminal Ileum
ENSG00000105383.15	CD33	chr19_51246334_C_T_b38	rs1566578	0.5	0.0000074	Small Intestine - Terminal Ileum
ENSG00000105383.15	CD33	chr19_51246958_G_A_b38	rs11084061	0.5	0.0000074	Small Intestine - Terminal Ileum
ENSG00000105383.15	CD33	chr19_51246983_T_C_b38	rs11084062	0.5	0.0000074	Small Intestine - Terminal Ileum
ENSG00000105383.15	CD33	chr19_51248679_C_A_b38	rs989502	0.5	0.0000074	Small Intestine - Terminal Ileum
ENSG00000105383.15	CD33	chr19_51248695_G_A_b38	rs989505	0.5	0.0000074	Small Intestine - Terminal Ileum
ENSG00000105383.15	CD33	chr19_51248696_C_A_b38	rs1319675	0.5	0.0000074	Small Intestine - Terminal Ileum
ENSG00000105383.15	CD33	chr19_51250704_G_C_b38	rs7253829	0.5	0.0000074	Small Intestine - Terminal Ileum
ENSG00000105383.15	CD33	chr19_51250705_G_A_b38	rs7253831	0.5	0.0000074	Small Intestine - Terminal Ileum
ENSG00000105383.15	CD33	chr19_51213820_A_G_b38	rs2411329	-0.11	0.0000077	Lung
ENSG00000105383.15	CD33	chr19_51220290_C_G_b38	rs273637	0.14	0.0000077	Adrenal Gland
ENSG00000105383.15	CD33	chr19_51242324_T_G_b38	rs1034521	0.17	0.0000077	Breast - Mammary Tissue
ENSG00000105383.15	CD33	chr19_51243667_G_A_b38	rs12461354	0.17	0.0000077	Breast - Mammary Tissue
ENSG00000105383.15	CD33	chr19_51243997_T_C_b38	rs73612300	0.25	0.0000077	Colon - Sigmoid
ENSG00000105383.15	CD33	chr19_51246334_C_T_b38	rs1566578	0.17	0.0000077	Breast - Mammary Tissue
ENSG00000105383.15	CD33	chr19_51246958_G_A_b38	rs11084061	0.17	0.0000077	Breast - Mammary Tissue
ENSG00000105383.15	CD33	chr19_51246983_T_C_b38	rs11084062	0.17	0.0000077	Breast - Mammary Tissue
ENSG00000105383.15	CD33	chr19_51247502_T_C_b38	rs12461282	0.17	0.0000077	Breast - Mammary Tissue
ENSG00000105383.15	CD33	chr19_51248679_C_A_b38	rs989502	0.17	0.0000077	Breast - Mammary Tissue
ENSG00000105383.15	CD33	chr19_51248695_G_A_b38	rs989505	0.17	0.0000077	Breast - Mammary Tissue
ENSG00000105383.15	CD33	chr19_51248696_C_A_b38	rs1319675	0.17	0.0000077	Breast - Mammary Tissue
ENSG00000105383.15	CD33	chr19_51251278_A_C_b38	rs9676731	0.15	0.0000077	Nerve - Tibial
ENSG00000105383.15	CD33	chr19_51176765_T_TCA_b38	rs59548084	0.1	0.0000078	Nerve - Tibial
ENSG00000105383.15	CD33	chr19_51192653_G_A_b38	rs11672116	0.087	0.0000079	Whole Blood
ENSG00000105383.15	CD33	chr19_51242324_T_G_b38	rs1034521	0.21	0.000008	Colon - Transverse
ENSG00000105383.15	CD33	chr19_51243667_G_A_b38	rs12461354	0.21	0.000008	Colon - Transverse
ENSG00000105383.15	CD33	chr19_51246334_C_T_b38	rs1566578	0.21	0.000008	Colon - Transverse
ENSG00000105383.15	CD33	chr19_51246958_G_A_b38	rs11084061	0.21	0.000008	Colon - Transverse

ENSG00000105383.15	CD33	chr19_51246983_T_C_b38	rs11084062	0.21	0.000008	Colon - Transverse
ENSG00000105383.15	CD33	chr19_51248679_C_A_b38	rs989502	0.21	0.000008	Colon - Transverse
ENSG00000105383.15	CD33	chr19_51248695_G_A_b38	rs989505	0.21	0.000008	Colon - Transverse
ENSG00000105383.15	CD33	chr19_51248696_C_A_b38	rs1319675	0.21	0.000008	Colon - Transverse
ENSG00000105383.15	CD33	chr19_51198493_T_G_b38	rs10404590	0.1	0.0000081	Nerve - Tibial
ENSG00000105383.15	CD33	chr19_51242324_T_G_b38	rs1034521	0.23	0.0000081	Adrenal Gland
ENSG00000105383.15	CD33	chr19_51243667_G_A_b38	rs12461354	0.23	0.0000081	Adrenal Gland
ENSG00000105383.15	CD33	chr19_51246334_C_T_b38	rs1566578	0.23	0.0000081	Adrenal Gland
ENSG00000105383.15	CD33	chr19_51246958_G_A_b38	rs11084061	0.23	0.0000081	Adrenal Gland
ENSG00000105383.15	CD33	chr19_51246983_T_C_b38	rs11084062	0.23	0.0000081	Adrenal Gland
ENSG00000105383.15	CD33	chr19_51247502_T_C_b38	rs12461282	0.23	0.0000081	Adrenal Gland
ENSG00000105383.15	CD33	chr19_51248679_C_A_b38	rs989502	0.23	0.0000081	Adrenal Gland
ENSG00000105383.15	CD33	chr19_51248695_G_A_b38	rs989505	0.23	0.0000081	Adrenal Gland
ENSG00000105383.15	CD33	chr19_51248696_C_A_b38	rs1319675	0.23	0.0000081	Adrenal Gland
ENSG00000105383.15	CD33	chr19_51276877_T_C_b38	rs8113548	-0.18	0.0000083	Brain - Caudate (basal ganglia)
ENSG00000105383.15	CD33	chr19_51247502_T_C_b38	rs12461282	0.26	0.0000083	Colon - Sigmoid
ENSG00000105383.15	CD33	chr19_51256448_A_T_b38	rs1501448	0.17	0.0000083	Artery - Tibial
ENSG00000105383.15	CD33	chr19_51258097_G_C_b38	rs62115997	-0.47	0.0000085	Brain - Spinal cord (cervical c-1)
ENSG00000105383.15	CD33	chr19_51258337_G_C_b38	rs62115998	-0.47	0.0000085	Brain - Spinal cord (cervical c-1)
ENSG00000105383.15	CD33	chr19_51258360_G_A_b38	rs62115999	-0.47	0.0000085	Brain - Spinal cord (cervical c-1)
ENSG00000105383.15	CD33	chr19_51199836_ACTCTTCTTGGGGAC_A_b38	rs147374894	0.083	0.0000087	Adipose - Subcutaneous
ENSG00000105383.15	CD33	chr19_51262616_A_T_b38	rs10412214	0.19	0.0000087	Adipose - Visceral (Omentum)
ENSG00000105383.15	CD33	chr19_51193473_G_A_b38	rs7255660	0.1	0.0000089	Artery - Tibial
ENSG00000105383.15	CD33	chr19_51220856_A_G_b38	rs273638	0.078	0.000009	Adipose - Subcutaneous
ENSG00000105383.15	CD33	chr19_51259327_T_C_b38	rs10412123	0.14	0.000009	Artery - Tibial
ENSG00000105383.15	CD33	chr19_51260244_C_T_b38	rs11668174	0.28	0.0000091	Colon - Sigmoid
ENSG00000105383.15	CD33	chr19_51250704_G_C_b38	rs7253829	0.21	0.0000095	Colon - Transverse
ENSG00000105383.15	CD33	chr19_51250705_G_A_b38	rs7253831	0.21	0.0000095	Colon - Transverse
ENSG00000105383.15	CD33	chr19_51248395_G_T_b38	rs989504	0.27	0.00001	Colon - Sigmoid
ENSG00000105383.15	CD33	chr19_51256271_T_C_b38	rs10402797	0.17	0.00001	Adipose - Visceral (Omentum)
ENSG00000105383.15	CD33	chr19_51256870_A_T_b38	rs1501449	0.17	0.00001	Breast - Mammary Tissue
ENSG00000105383.15	CD33	chr19_51332295_G_A_b38	rs35525343	0.24	0.00001	Colon - Sigmoid
ENSG00000105383.15	CD33	chr19_50565889_G_A_b38	rs60246192	-0.12	0.000011	Lung
ENSG00000105383.15	CD33	chr19_51175173_G_A_b38	rs2134068	0.088	0.000011	Adipose - Subcutaneous
ENSG00000105383.15	CD33	chr19_51185559_T_G_b38	rs59266985	0.083	0.000011	Adipose - Subcutaneous
ENSG00000105383.15	CD33	chr19_51185832_T_C_b38	rs10405784	0.1	0.000011	Nerve - Tibial
ENSG00000105383.15	CD33	chr19_51220906_A_G_b38	rs273639	0.077	0.000011	Adipose - Subcutaneous
ENSG00000105383.15	CD33	chr19_51224066_A_G_b38	rs1697553	0.076	0.000011	Adipose - Subcutaneous
ENSG00000105383.15	CD33	chr19_51225847_CCCGG_C_b38	rs201074739	-0.61	0.000011	Brain - Caudate (basal ganglia)
ENSG00000105383.15	CD33	chr19_51225847_CCCGG_C_b38	rs201074739	-0.37	0.000011	Esophagus - Muscularis
ENSG00000105383.15	CD33	chr19_51247502_T_C_b38	rs12461282	0.5	0.000011	Small Intestine - Terminal Ileum
ENSG00000105383.15	CD33	chr19_51187448_T_C_b38	rs35203095	0.081	0.000012	Adipose - Subcutaneous
ENSG00000105383.15	CD33	chr19_51193216_A_G_b38	rs11084055	0.1	0.000012	Nerve - Tibial
ENSG00000105383.15	CD33	chr19_51201783_T_C_b38	rs7256201	-0.079	0.000012	Adipose - Subcutaneous
ENSG00000105383.15	CD33	chr19_51214320_C_T_b38	rs4802772	0.19	0.000012	Lung
ENSG00000105383.15	CD33	chr19_51240790_G_T_b38	rs8103085	0.18	0.000012	Breast - Mammary Tissue
ENSG00000105383.15	CD33	chr19_51255390_A_T_b38	rs7256512	0.16	0.000012	Adipose - Visceral (Omentum)
ENSG00000105383.15	CD33	chr19_51256870_A_T_b38	rs1501449	0.23	0.000012	Colon - Sigmoid
ENSG00000105383.15	CD33	chr19_51192653_G_A_b38	rs11672116	0.082	0.000013	Adipose - Subcutaneous
ENSG00000105383.15	CD33	chr19_51193473_G_A_b38	rs7255660	0.1	0.000013	Nerve - Tibial
ENSG00000105383.15	CD33	chr19_51215061_T_A_b38	rs1697573	0.14	0.000013	Adrenal Gland
ENSG00000105383.15	CD33	chr19_51223655_A_C_b38	rs1710398	0.076	0.000013	Adipose - Subcutaneous
ENSG00000105383.15	CD33	chr19_51246788_T_C_b38	rs145323276	0.24	0.000013	Adipose - Visceral (Omentum)
ENSG00000105383.15	CD33	chr19_51250704_G_C_b38	rs7253829	0.17	0.000013	Breast - Mammary Tissue
ENSG00000105383.15	CD33	chr19_51250705_G_A_b38	rs7253831	0.17	0.000013	Breast - Mammary Tissue
ENSG00000105383.15	CD33	chr19_51251278_A_C_b38	rs9676731	0.22	0.000013	Spleen
ENSG00000105383.15	CD33	chr19_51184343_A_G_b38	rs10416576	0.098	0.000014	Nerve - Tibial
ENSG00000105383.15	CD33	chr19_51185586_G_C_b38	rs10404091	0.098	0.000014	Nerve - Tibial
ENSG00000105383.15	CD33	chr19_51185948_A_C_b38	rs10404245	0.099	0.000014	Nerve - Tibial
ENSG00000105383.15	CD33	chr19_51190234_G_A_b38	rs9973300	0.08	0.000014	Adipose - Subcutaneous
ENSG00000105383.15	CD33	chr19_51201503_A_G_b38	rs7251705	-0.26	0.000014	Muscle - Skeletal
ENSG00000105383.15	CD33	chr19_51220290_C_G_b38	rs273637	0.076	0.000014	Adipose - Subcutaneous
ENSG00000105383.15	CD33	chr19_51223099_G_C_b38	rs1399837	0.13	0.000014	Brain - Spinal cord (cervical c-1)
ENSG00000105383.15	CD33	chr19_51256271_T_C_b38	rs10402797	0.17	0.000014	Breast - Mammary Tissue
ENSG00000105383.15	CD33	chr19_51256271_T_C_b38	rs10402797	0.24	0.000014	Colon - Sigmoid
ENSG00000105383.15	CD33	chr19_51192887_G_A_b38	rs7254889	0.077	0.000015	Adipose - Subcutaneous
ENSG00000105383.15	CD33	chr19_51192888_G_T_b38	rs7254890	0.077	0.000015	Adipose - Subcutaneous
ENSG00000105383.15	CD33	chr19_51199836_ACTCTTCTTGGGGAC_A_b38	rs147374894	0.1	0.000015	Artery - Tibial
ENSG00000105383.15	CD33	chr19_51240790_G_T_b38	rs8103085	0.23	0.000015	Adrenal Gland
ENSG00000105383.15	CD33	chr19_51182450_G_T_b38	rs7246268	0.15	0.000016	Colon - Sigmoid
ENSG00000105383.15	CD33	chr19_51220856_A_G_b38	rs273638	0.13	0.000016	Adrenal Gland
ENSG00000105383.15	CD33	chr19_51220906_A_G_b38	rs273639	0.13	0.000016	Adrenal Gland
ENSG00000105383.15	CD33	chr19_51221277_A_ACACAATATTTTAC_b38	rs11273989	0.13	0.000016	Adrenal Gland
ENSG00000105383.15	CD33	chr19_51441435_T_G_b38	rs118152930	-0.75	0.000016	Brain - Caudate (basal ganglia)
ENSG00000105383.15	CD33	chr19_51247502_T_C_b38	rs12461282	0.2	0.000016	Colon - Transverse
ENSG00000105383.15	CD33	chr19_51184466_C_CG_b38		0.098	0.000017	Nerve - Tibial
ENSG00000105383.15	CD33	chr19_51217369_A_T_b38	rs1566576	0.074	0.000017	Adipose - Subcutaneous
ENSG00000105383.15	CD33	chr19_51250704_G_C_b38	rs7253829	0.22	0.000017	Adrenal Gland
ENSG00000105383.15	CD33	chr19_51250705_G_A_b38	rs7253831	0.22	0.000017	Adrenal Gland
ENSG00000105383.15	CD33	chr19_51256436_A_G_b38	rs1501447	0.31	0.000017	Brain - Putamen (basal ganglia)

ENSG00000105383.15	CD33	chr19_51258097_G_C_b38	rs62115997	-0.38	0.000017	Colon - Transverse
ENSG00000105383.15	CD33	chr19_51258337_G_C_b38	rs62115998	-0.38	0.000017	Colon - Transverse
ENSG00000105383.15	CD33	chr19_51258360_G_A_b38	rs62115999	-0.38	0.000017	Colon - Transverse
ENSG00000105383.15	CD33	chr19_51331683_C_A_b38	rs36054492	0.23	0.000017	Colon - Sigmoid
ENSG00000105383.15	CD33	chr19_51334106_T_G_b38	rs11668576	-0.34	0.000017	Nerve - Tibial
ENSG00000105383.15	CD33	chr19_50901931_C_A_b38	rs806022	-0.099	0.000018	Esophagus - Muscularis
ENSG00000105383.15	CD33	chr19_51185059_C_T_b38	rs16982535	0.097	0.000018	Nerve - Tibial
ENSG00000105383.15	CD33	chr19_51186850_T_C_b38	rs34186776	0.079	0.000018	Adipose - Subcutaneous
ENSG00000105383.15	CD33	chr19_51188393_T_C_b38	rs10421852	0.077	0.000018	Adipose - Subcutaneous
ENSG00000105383.15	CD33	chr19_51194930_C_T_b38	rs10405709	0.076	0.000018	Adipose - Subcutaneous
ENSG00000105383.15	CD33	chr19_51196555_C_T_b38	rs10419722	0.085	0.000018	Whole Blood
ENSG00000105383.15	CD33	chr19_51255390_A_T_b38	rs7256512	0.22	0.000018	Spleen
ENSG00000105383.15	CD33	chr19_51185559_T_G_b38	rs59266985	0.1	0.000019	Nerve - Tibial
ENSG00000105383.15	CD33	chr19_51187836_C_T_b38	rs7255837	0.076	0.000019	Adipose - Subcutaneous
ENSG00000105383.15	CD33	chr19_51196555_C_T_b38	rs10419722	0.081	0.000019	Adipose - Subcutaneous
ENSG00000105383.15	CD33	chr19_50228154_C_T_b38	rs56066137	0.079	0.00002	Adipose - Subcutaneous
ENSG00000105383.15	CD33	chr19_50780534_T_G_b38	rs12609769	0.12	0.00002	Muscle - Skeletal
ENSG00000105383.15	CD33	chr19_51186703_C_T_b38	rs67836621	0.079	0.00002	Adipose - Subcutaneous
ENSG00000105383.15	CD33	chr19_51186912_T_A_b38	rs35469086	0.079	0.00002	Adipose - Subcutaneous
ENSG00000105383.15	CD33	chr19_51225385_A_G_b38	rs2455069	0.1	0.00002	Skin - Sun Exposed (Lower leg)
ENSG00000105383.15	CD33	chr19_51235662_G_A_b38	rs35112940	-0.11	0.00002	Artery - Tibial
ENSG00000105383.15	CD33	chr19_50903435_C_T_b38	rs1090647	-0.097	0.000021	Esophagus - Muscularis
ENSG00000105383.15	CD33	chr19_51185832_T_C_b38	rs10405784	0.076	0.000021	Adipose - Subcutaneous
ENSG00000105383.15	CD33	chr19_51194930_C_T_b38	rs10405709	0.098	0.000021	Artery - Tibial
ENSG00000105383.15	CD33	chr19_51198493_T_G_b38	rs10404590	0.08	0.000021	Whole Blood
ENSG00000105383.15	CD33	chr19_51199836_ACTCTTCTTGGGGAC_A_b38	rs147374894	0.082	0.000021	Whole Blood
ENSG00000105383.15	CD33	chr19_51216221_C_CGT_b38	rs1491213119	0.11	0.000021	Artery - Aorta
ENSG00000105383.15	CD33	chr19_51220290_C_G_b38	rs273637	0.13	0.000021	Brain - Spinal cord (cervical c-1)
ENSG00000105383.15	CD33	chr19_51235934_G_A_b38	rs142802794	0.47	0.000021	Spleen
ENSG00000105383.15	CD33	chr19_51193473_G_A_b38	rs7255660	0.078	0.000022	Adipose - Subcutaneous
ENSG00000105383.15	CD33	chr19_51198635_A_G_b38	rs10421385	0.079	0.000022	Whole Blood
ENSG00000105383.15	CD33	chr19_51216221_C_CGT_b38	rs1491213119	0.11	0.000022	Artery - Tibial
ENSG00000105383.15	CD33	chr19_51235662_G_A_b38	rs35112940	-0.088	0.000022	Adipose - Subcutaneous
ENSG00000105383.15	CD33	chr19_51183354_A_G_b38	rs11666927	0.095	0.000023	Nerve - Tibial
ENSG00000105383.15	CD33	chr19_51265713_G_T_b38	rs1875425	0.11	0.000023	Adipose - Subcutaneous
ENSG00000105383.15	CD33	chr19_51174413_T_C_b38	rs4802771	0.076	0.000024	Adipose - Subcutaneous
ENSG00000105383.15	CD33	chr19_51192653_G_A_b38	rs11672116	0.1	0.000024	Nerve - Tibial
ENSG00000105383.15	CD33	chr19_51243997_T_C_b38	rs73612300	0.15	0.000024	Artery - Aorta
ENSG00000105383.15	CD33	chr19_51192221_T_C_b38	rs10411928	0.076	0.000025	Adipose - Subcutaneous
ENSG00000105383.15	CD33	chr19_51225385_A_G_b38	rs2455069	0.073	0.000026	Adipose - Subcutaneous
ENSG00000105383.15	CD33	chr19_51251278_A_C_b38	rs9676731	0.15	0.000026	Breast - Mammary Tissue
ENSG00000105383.15	CD33	chr19_51250875_G_T_b38	rs17801843	0.17	0.000027	Esophagus - Muscularis
ENSG00000105383.15	CD33	chr19_51178709_C_A_b38	rs17715691	0.083	0.000028	Whole Blood
ENSG00000105383.15	CD33	chr19_51184343_A_G_b38	rs10416576	0.075	0.000028	Adipose - Subcutaneous
ENSG00000105383.15	CD33	chr19_51185586_G_C_b38	rs10404091	0.075	0.000028	Adipose - Subcutaneous
ENSG00000105383.15	CD33	chr19_51185948_A_C_b38	rs10404245	0.075	0.000028	Adipose - Subcutaneous
ENSG00000105383.15	CD33	chr19_51193216_A_G_b38	rs11084055	0.077	0.000028	Adipose - Subcutaneous
ENSG00000105383.15	CD33	chr19_51250875_G_T_b38	rs17801843	0.22	0.000028	Adrenal Gland
ENSG00000105383.15	CD33	chr19_51260103_T_C_b38	rs11672446	0.26	0.000028	Colon - Sigmoid
ENSG00000105383.15	CD33	chr19_51190844_A_G_b38	rs7245847	0.076	0.000029	Adipose - Subcutaneous
ENSG00000105383.15	CD33	chr19_51246788_T_C_b38	rs145323276	0.2	0.000029	Artery - Tibial
ENSG00000105383.15	CD33	chr19_51259327_T_C_b38	rs10412123	0.15	0.000029	Breast - Mammary Tissue
ENSG00000105383.15	CD33	chr19_51196555_C_T_b38	rs10419722	0.1	0.000031	Artery - Tibial
ENSG00000105383.15	CD33	chr19_51187113_G_C_b38	rs73055111	-0.55	0.000031	Brain - Caudate (basal ganglia)
ENSG00000105383.15	CD33	chr19_51259327_T_C_b38	rs10412123	0.15	0.000031	Adipose - Visceral (Omentum)
ENSG00000105383.15	CD33	chr19_51259327_T_C_b38	rs10412123	0.21	0.000031	Spleen
ENSG00000105383.15	CD33	chr19_51261577_C_T_b38	rs11084064	0.25	0.000033	Colon - Sigmoid
ENSG00000105383.15	CD33	chr19_51184466_C_CG_b38		0.074	0.000034	Adipose - Subcutaneous
ENSG00000105383.15	CD33	chr19_51185059_C_T_b38	rs16982535	0.074	0.000034	Adipose - Subcutaneous
ENSG00000105383.15	CD33	chr19_51213820_A_G_b38	rs2411329	-0.11	0.000034	Colon - Transverse
ENSG00000105383.15	CD33	chr19_51198635_A_G_b38	rs10421385	0.074	0.000035	Adipose - Subcutaneous
ENSG00000105383.15	CD33	chr19_51223357_G_A_b38	rs3826656	0.084	0.000035	Whole Blood
ENSG00000105383.15	CD33	chr19_51217369_A_T_b38	rs1566576	0.13	0.000036	Adrenal Gland
ENSG00000105383.15	CD33	chr19_51222568_G_T_b38	rs273640	0.097	0.000036	Skin - Sun Exposed (Lower leg)
ENSG00000105383.15	CD33	chr19_51461137_T_TGTGTG_b38	rs1568518027	-0.42	0.000036	Muscle - Skeletal
ENSG00000105383.15	CD33	chr19_51189214_T_C_b38	rs34528093	0.1	0.000037	Artery - Tibial
ENSG00000105383.15	CD33	chr19_51217988_G_A_b38	rs79254320	0.21	0.000038	Artery - Tibial
ENSG00000105383.15	CD33	chr19_51175173_G_A_b38	rs2134068	0.1	0.000039	Artery - Tibial
ENSG00000105383.15	CD33	chr19_51176765_T_TCA_b38	rs59548084	0.072	0.000039	Adipose - Subcutaneous
ENSG00000105383.15	CD33	chr19_50901828_T_C_b38	rs806023	-0.096	0.00004	Esophagus - Muscularis
ENSG00000105383.15	CD33	chr19_51251278_A_C_b38	rs9676731	0.15	0.00004	Esophagus - Muscularis
ENSG00000105383.15	CD33	chr19_51183449_G_T_b38	rs11667023	0.096	0.000041	Artery - Tibial
ENSG00000105383.15	CD33	chr19_51223099_G_C_b38	rs1399837	0.071	0.000041	Adipose - Subcutaneous
ENSG00000105383.15	CD33	chr19_51298886_T_C_b38	rs1710354	0.1	0.000041	Lung
ENSG00000105383.15	CD33	chr19_51178709_C_A_b38	rs17715691	0.099	0.000042	Artery - Tibial
ENSG00000105383.15	CD33	chr19_51183449_G_T_b38	rs11667023	0.094	0.000042	Nerve - Tibial
ENSG00000105383.15	CD33	chr19_51189434_C_A_b38	rs71358815	-0.16	0.000042	Nerve - Tibial
ENSG00000105383.15	CD33	chr19_51256870_A_T_b38	rs1501449	0.14	0.000042	Artery - Tibial
ENSG00000105383.15	CD33	chr19_51215581_C_T_b38	rs273634	0.071	0.000043	Adipose - Subcutaneous
ENSG00000105383.15	CD33	chr19_51258097_G_C_b38	rs62115997	-0.35	0.000043	Skin - Not Sun Exposed (Suprapubic)

ENSG00000105383.15	CD33	chr19_51258337_G_C_b38	rs62115998	-0.35	0.000043	Skin - Not Sun Exposed (Suprapubic)
ENSG00000105383.15	CD33	chr19_51258360_G_A_b38	rs62115999	-0.35	0.000043	Skin - Not Sun Exposed (Suprapubic)
ENSG00000105383.15	CD33	chr19_51218889_A_G_b38	rs1710403	0.081	0.000044	Whole Blood
ENSG00000105383.15	CD33	chr19_51255390_A_T_b38	rs7256512	0.15	0.000045	Breast - Mammary Tissue
ENSG00000105383.15	CD33	chr19_51256448_A_T_b38	rs1501448	0.17	0.000045	Breast - Mammary Tissue
ENSG00000105383.15	CD33	chr19_51177310_T_C_b38	rs11670380	0.071	0.000048	Adipose - Subcutaneous
ENSG00000105383.15	CD33	chr19_51224937_T_C_b38	rs2459141	0.095	0.000048	Skin - Sun Exposed (Lower leg)
ENSG00000105383.15	CD33	chr19_51206550_G_A_b38	rs142760794	-0.45	0.000049	Artery - Aorta
ENSG00000105383.15	CD33	chr19_51262616_A_T_b38	rs10412214	0.16	0.00005	Nerve - Tibial
ENSG00000105383.15	CD33	chr19_50905352_C_A_b38	rs2569530	-0.093	0.000051	Esophagus - Muscularis
ENSG00000105383.15	CD33	chr19_51198635_A_G_b38	rs10421385	0.093	0.000051	Artery - Tibial
ENSG00000105383.15	CD33	chr19_51183354_A_G_b38	rs11666927	0.072	0.000052	Adipose - Subcutaneous
ENSG00000105383.15	CD33	chr19_51214320_C_T_b38	rs4802772	0.15	0.000052	Nerve - Tibial
ENSG00000105383.15	CD33	chr19_51188505_G_C_b38	rs35413640	-0.17	0.000053	Nerve - Tibial
ENSG00000105383.15	CD33	chr19_51224937_T_C_b38	rs2459141	0.07	0.000053	Adipose - Subcutaneous
ENSG00000105383.15	CD33	chr19_50903067_C_CGGGTTCAAGGGATTCTCCTGTCTCA_b38	rs149757666	-0.091	0.000054	Esophagus - Muscularis
ENSG00000105383.15	CD33	chr19_50909410_C_A_b38	rs1654552	-0.094	0.000054	Esophagus - Muscularis
ENSG00000105383.15	CD33	chr19_51221070_T_C_b38	rs200656	0.081	0.000054	Whole Blood
ENSG00000105383.15	CD33	chr19_51198493_T_G_b38	rs10404590	0.073	0.000055	Adipose - Subcutaneous
ENSG00000105383.15	CD33	chr19_51217988_G_A_b38	rs79254320	0.23	0.000055	Skin - Sun Exposed (Lower leg)
ENSG00000105383.15	CD33	chr19_51235960_T_C_b38	rs147493755	0.32	0.000056	Nerve - Tibial
ENSG00000105383.15	CD33	chr19_51259327_T_C_b38	rs10412123	0.15	0.000056	Esophagus - Muscularis
ENSG00000105383.15	CD33	chr19_50903357_A_G_b38	rs1090648	-0.093	0.000057	Esophagus - Muscularis
ENSG00000105383.15	CD33	chr19_50903522_A_C_b38	rs806018	-0.092	0.000057	Esophagus - Muscularis
ENSG00000105383.15	CD33	chr19_51189214_T_C_b38	rs34528093	0.1	0.000057	Nerve - Tibial
ENSG00000105383.15	CD33	chr19_51198493_T_G_b38	rs10404590	0.093	0.000057	Artery - Tibial
ENSG00000105383.15	CD33	chr19_51223655_A_C_b38	rs1710398	0.094	0.000057	Skin - Sun Exposed (Lower leg)
ENSG00000105383.15	CD33	chr19_51224066_A_G_b38	rs1697553	0.094	0.000057	Skin - Sun Exposed (Lower leg)
ENSG00000105383.15	CD33	chr19_51255390_A_T_b38	rs7256512	0.15	0.000058	Esophagus - Muscularis
ENSG00000105383.15	CD33	chr19_51255314_A_T_b38	rs7256372	0.18	0.000059	Esophagus - Muscularis
ENSG00000105383.15	CD33	chr19_51256448_A_T_b38	rs1501448	0.17	0.000059	Esophagus - Muscularis
ENSG00000105383.15	CD33	chr19_51299396_A_G_b38	rs1710353	0.085	0.000059	Artery - Aorta
ENSG00000105383.15	CD33	chr19_51334078_A_G_b38	rs10418551	0.09	0.000061	Artery - Aorta
ENSG00000105383.15	CD33	chr19_51334846_C_CAAG_b38	rs34501324	0.09	0.000061	Artery - Aorta
ENSG00000105383.15	CD33	chr19_51298886_T_C_b38	rs1710354	0.088	0.000062	Nerve - Tibial
ENSG00000105383.15	CD33	chr19_51196555_C_T_b38	rs10419722	0.098	0.000063	Nerve - Tibial
ENSG00000105383.15	CD33	chr19_51215611_G_A_b38	rs273635	0.08	0.000064	Whole Blood
ENSG00000105383.15	CD33	chr19_51247868_C_T_b38	rs11084063	0.16	0.000064	Nerve - Tibial
ENSG00000105383.15	CD33	chr19_51248395_G_T_b38	rs989504	0.16	0.000064	Nerve - Tibial
ENSG00000105383.15	CD33	chr19_50902552_C_T_b38	rs806020	-0.091	0.000065	Esophagus - Muscularis
ENSG00000105383.15	CD33	chr19_51296334_G_T_b38	rs28429628	-0.34	0.000065	Muscle - Skeletal
ENSG00000105383.15	CD33	chr19_51296609_T_C_b38	rs28585037	-0.34	0.000065	Muscle - Skeletal
ENSG00000105383.15	CD33	chr19_51296851_C_T_b38	rs10416135	-0.34	0.000065	Muscle - Skeletal
ENSG00000105383.15	CD33	chr19_51297087_A_G_b38	rs7254340	-0.34	0.000065	Muscle - Skeletal
ENSG00000105383.15	CD33	chr19_51297387_G_A_b38	rs7255071	-0.34	0.000065	Muscle - Skeletal
ENSG00000105383.15	CD33	chr19_51221277_A_ACACAATATTTTAC_b38	rs11273989	0.094	0.000066	Skin - Sun Exposed (Lower leg)
ENSG00000105383.15	CD33	chr19_51177310_T_C_b38	rs11670380	0.088	0.000067	Artery - Tibial
ENSG00000105383.15	CD33	chr19_51217369_A_T_b38	rs1566576	0.093	0.000067	Skin - Sun Exposed (Lower leg)
ENSG00000105383.15	CD33	chr19_51176765_T_TCA_b38	rs59548084	0.088	0.00007	Artery - Tibial
ENSG00000105383.15	CD33	chr19_51223099_G_C_b38	rs1399837	0.093	0.00007	Skin - Sun Exposed (Lower leg)
ENSG00000105383.15	CD33	chr19_52134402_C_T_b38	rs79598869	0.49	0.000072	Skin - Not Sun Exposed (Suprapubic)
ENSG00000105383.15	CD33	chr19_51220856_A_G_b38	rs273638	0.093	0.000073	Skin - Sun Exposed (Lower leg)
ENSG00000105383.15	CD33	chr19_51256271_T_C_b38	rs10402797	0.13	0.000073	Artery - Tibial
ENSG00000105383.15	CD33	chr19_51277766_G_A_b38	rs12459452	0.1	0.000073	Adipose - Subcutaneous
ENSG00000105383.15	CD33	chr19_50900964_A_T_b38	rs1701941	-0.093	0.000074	Esophagus - Muscularis
ENSG00000105383.15	CD33	chr19_51185559_T_G_b38	rs59266985	0.096	0.000075	Artery - Tibial
ENSG00000105383.15	CD33	chr19_51215581_C_T_b38	rs273634	0.093	0.000075	Skin - Sun Exposed (Lower leg)
ENSG00000105383.15	CD33	chr19_51183354_A_G_b38	rs11666927	0.089	0.000078	Artery - Tibial
ENSG00000105383.15	CD33	chr19_51192653_G_A_b38	rs11672116	0.095	0.000078	Artery - Tibial
ENSG00000105383.15	CD33	chr19_51220290_C_G_b38	rs273637	0.093	0.000078	Skin - Sun Exposed (Lower leg)
ENSG00000105383.15	CD33	chr19_51175173_G_A_b38	rs2134068	0.11	0.000079	Skin - Sun Exposed (Lower leg)
ENSG00000105383.15	CD33	chr19_51220906_A_G_b38	rs273639	0.094	0.000079	Skin - Sun Exposed (Lower leg)
ENSG00000105383.15	CD33	chr19_51298370_T_C_b38	rs7260113	0.063	0.00008	Adipose - Subcutaneous
ENSG00000105383.15	CD33	chr19_51219326_A_G_b38	rs12609179	0.092	0.000084	Skin - Sun Exposed (Lower leg)
ENSG00000105383.15	CD33	chr19_51260244_C_T_b38	rs11668174	0.16	0.000085	Artery - Tibial
ENSG00000105383.15	CD33	chr19_51219326_A_G_b38	rs12609179	0.068	0.000087	Adipose - Subcutaneous
ENSG00000105383.15	CD33	chr19_51174413_T_C_b38	rs4802771	0.09	0.000089	Artery - Tibial
ENSG00000105383.15	CD33	chr19_51187836_C_T_b38	rs7255837	0.089	0.000089	Artery - Tibial
ENSG00000105383.15	CD33	chr19_51215061_T_A_b38	rs1697573	0.092	0.0001	Skin - Sun Exposed (Lower leg)
ENSG00000105383.15	CD33	chr19_51255314_A_T_b38	rs7256372	0.16	0.0001	Nerve - Tibial
ENSG00000105383.15	CD33	chr19_51256436_A_G_b38	rs1501447	0.14	0.0001	Nerve - Tibial
ENSG00000105383.15	CD33	chr19_51199836_ACTCTTCTTGGGGAC_A_b38	rs147374894	0.091	0.00012	Nerve - Tibial
